# A long-lived impact-generated hydrothermal system at the Chicxulub impact structure

**DOI:** 10.1038/s43247-026-03618-5

**Published:** 2026-06-09

**Authors:** Annemarie E. Pickersgill, Evangelos Christou, Marissa M. Tremblay, Dan N. Barfod, Cornelia Rasmussen, Martin R. Lee, Martin Schmieder, Gareth S. Collins, Ross Dymock, Sean P. S. Gulick, David A. Kring, Joanna V. Morgan, Gordon R. Osinski, Timothy Swindle, Sonia M. Tikoo, Axel Wittmann, the Expedition 364 Scientists, Timothy Bralower, Elise Chenot, Gail Christeson, Philippe Claeys, Charles Cockell, Marco Coolen, Ludovic Ferrière, Catalina Gebhardt, Kazuhisa Goto, Heather Jones, Xiao Long, Chris Lowery, Rubén Ocampo-Torres, Ligia Perez-Cruz, Michael Poelchau, Auriol S. P. Rae, Mario Rebolledo-Vieyra, Ulrich Riller, Honami Sato, Jan Smit, Naotaka Tomioka, Jaime Urrutia Fucugauchi, Michael Whalen, Kosei Yamagachi, William Zylberman, Darren F. Mark

**Affiliations:** 1 SUERC: Centre for the Isotope Sciences, East Kilbride, UK; 2 School of Geographical & Earth Sciences, University of Glasgow, Molema Building, Glasgow, UK; 3 Department of Earth, Atmospheric, and Planetary Sciences, Purdue University, West Lafayette, IN USA; 4 Institute for Geophysics & Department of Earth and Planetary Sciences, Jackson School of Geosciences, University of Texas at Austin, Austin, Texas USA; 5 Center for Lunar Science and Exploration, Lunar and Planetary Institute, Universities Space Research Association, Houston, TX USA; 6 HNU Neu-Ulm University of Applied Sciences, Neu-Ulm, Germany; 7 Department of Earth Science and Engineering, Imperial College London, London, UK; 8 Center for Planetary Systems Habitability, University of Texas at Austin, Austin, TX USA; 9 Department of Earth Sciences, Institute for Earth and Space Exploration, University of Western Ontario, London, ON Canada; 10 Lunar and Planetary Laboratory, University of Arizona, Tucson, AZ USA; 11 Department of Geophysics, Stanford University, Stanford, CA USA; 12 Eyring Materials Center, Arizona State University, Tempe, AZ USA; 13 Department of Earth & Environmental Science, University of St Andrews, St Andrews, UK; 14 Pennsylvania State University, University Park, PA USA; 15 Institut Polytechnique Lasalle Beauvais, Beauvais, France; 16 Directorate for Geosciences, U.S. National Science Foundation, Alexandra, USA; 17 Analytical-, Environmental-, and Geochemistry (AMGC), Vrije Universiteit Brussel (VUB), Brussels, Belgium; 18 UK Centre for Astrobiology School of Physics and Astronomy, University of Edinburgh, James Clerk Maxwell Building, Edinburgh, UK; 19 Department of Chemistry, Faculty of Science and Engineering, Curtin University, Perth, WA Australia; 20 Natural History Museum (Naturhistorisches Museum), Vienna, Austria; 21 Alfred Wegener Institute Helmholtz Centre of Polar and Marine Research, Bremerhaven, Germany; 22 Department of Earth and Planetary Science, The University of Tokyo, Tokyo, Japan; 23 School of Earth Sciences, Planetary Science Institute, China University of Geosciences (Wuhan), Wuhan, China; 24 Institute for Geophysics, University of Texas, J.J. Pickle Research Campus, Austin, TX USA; 25 Groupe de Physico-Chimie de l’Atmosphère, National Center of Scientific Research (CNRS), Institut de Chimie et Procédés pour l’Energie, l’Environnement et la Santé ICPEES, Université de Strasbourg, Strasbourg, France; 26 Geomagnetismo y Exploración, Instituto de Geofísica, Universidad Nacional Autónoma De México, Cd. Universitaria, Ciudad de México, Mexico; 27 Institute of Earth and Environmental Sciences – Geology, University of Freiburg, Freiburg, Germany; 28 School of Geosciences & Edinburgh Centre for Planetary Sciences, Grant Institute, University of Edinburgh, Edinburgh, UK; 29 Geosoluciones aplicadas, Cancun, Mexico; 30 Institut für Geologie, Universität Hamburg, Hamburg, Germany; 31 Department of Earth and Planetary Sciences, Kyushu University, Fukuoka, Japan; 32 Faculty of Earth and Life Sciences (FALW), Amsterdam, The Netherlands; 33 Japan Agency for Marine-Earth Science and Technology, Kochi Institute for Core Sample Research, Nankoku, Japan; 34 Institute of Geophysics at the National University of Mexico, Investigación Científica, Cd. Universitaria, Ciudad de México, Mexico; 35 Geosciences Department, University of Alaska Fairbanks, Fairbanks, AK USA; 36 Department of Chemistry, Tohu University, Funabashi, Japan; 37 Ginger SOFRECO, Clichy, France

**Keywords:** Astrobiology, Geochemistry, Geology

## Abstract

Hydrothermal systems likely played an essential role in the origin of life, both on Earth and potentially on other planets. They form anywhere that heat and aqueous fluids interact, including within cooling hypervelocity impact craters. Longer periods of hydrothermal activity will generate extended windows of opportunity for prebiotic chemical reactions to occur, life to develop, and micro-organisms to thrive and propagate beyond their point of origin. Here, we present radioisotopic age constraints and numerical simulations for the duration of post-impact hydrothermal activity in and around the peak ring of the ~200 km diameter 66 Ma Chicxulub impact structure. We find that hydrothermal activity persisted for at least 8 million years (Myr), which is approximately four times longer than previously estimated by numerical simulations, palaeomagnetic records, and petrographic interpretations at Chicxulub, making it the longest-lived impact generated hydrothermal system documented on Earth.

## Abstract (web summary)

Radioisotopic age constraints and numerical simulations on the duration of hydrothermal activity associated with the Chicxulub impact structure indicate the system was active for 8 million years, four times longer than previous estimates

## Introduction

The impact of asteroids and comets with planetary bodies is one of the most fundamental and widespread geological processes in the solar system^1,2^. Once thought of as purely destructive events, interest in large ancient meteorite impact structures has risen due to their potential to have created habitable environments in the first few hundred million years (Myr) of solar system history^3–15^. Hydrothermal systems form anywhere that heat and aqueous fluids interact^16^, including within cooling hypervelocity impact craters^9–11,17^. This has been proposed as a mechanism for creating habitable environments for thermophilic and hyperthermophilic micro-organisms^6^ and generating cradles for prebiotic chemical reactions^5,12^.

Impact-generated hydrothermal systems are widespread, with evidence of hydrothermal activity found at over 70 of the ~200 impact structures on Earth^18^. While it has been demonstrated that these environments are habitable via increased porosity/permeability, chemistry, and nutrient availability, explicit evidence of microbial colonisation in impact craters is harder to find (only 8 of the ~200 impact structures on Earth), and even more difficult to chronologically link with specific impact-generated processes^19^. We therefore approach the question of whether ancient impacts on Earth could have contributed to the origin of life by examining the windows of opportunity for habitability. We cannot say with certainty that these early impact environments were inhabited because so little of the rock record from Early Earth still exists, and therefore, the physical properties of the early Earth’s crust are poorly constrained. We can, however, examine whether impact craters may have provided the right temperature and fluid flux conditions, for sufficiently long durations, for life to emerge. In this work, we address the constraint of longevity by examining the duration of the impact-generated hydrothermal system in the Chicxulub peak-ring basin as an analogue for large impact basins in general.

The duration of hydrothermal activity in impact craters is a key parameter to understand their potential as incubators for life. Longer periods of hydrothermal activity and therefore post-impact habitability will generate extended windows of opportunity for prebiotic chemical reactions to occur, life to develop, and micro-organisms to thrive and propagate beyond their point of origin. However, the duration of impact-generated hydrothermal activity is poorly constrained. Here, we combine radio-isotopic (^40^Ar/^39^Ar) measurements and numerical simulations to explore the duration of hydrothermal activity at one of the largest and best-preserved impact structures on Earth, the Chicxulub peak-ring basin, México.

**Fig. 1:**
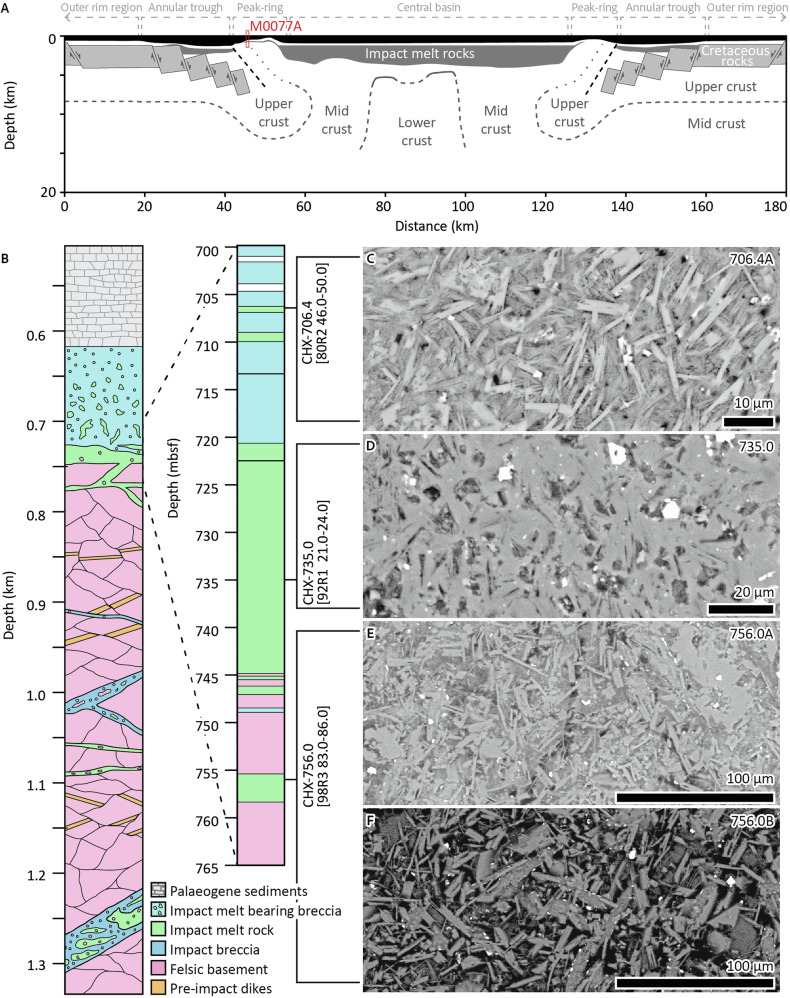
Geologic overview of Core M0077A and SEM images of impact melt rocks used in this study. **A** Simplified cross-section through the Chicxulub impact structure showing the location of the IODP-ICDP Expedition 364 site M0077A in the peak ring, modified from refs. ^92,93^ used with permission. **B** Lithologies recovered from site M0077A^22^. The inset shows an expanded lithologic column for samples used in this study, modified from ref. ^21^ used with permission, all of which are impact melt rocks. Backscattered electron images of CHX 706.4 (**C**), CHX 735.0 (**D**), CHX 756.0A (**E**), and CHX 756.0B (**F**).

Very large impact structures are preserved on other planets, asteroids, and moons, but direct investigation of them is limited because sample return from these bodies is technically demanding and prohibitively expensive. On Earth, these large impact structures characteristic of early solar system evolution have been lost due to the process of plate tectonics recycling the planet’s crust. However, we can investigate some of the key attributes of these structures by studying younger analogues of those very large craters—termed peak-ring basins—that are preserved. The 66 Ma Chicxulub impact structure, Yucatán, México (e.g. refs. ^20, 21^) is the best-preserved peak-ring basin on Earth, making it a good physical model of Hadean impact sites. The target rocks on early Earth are more likely to have been mafic, so post-impact hydrothermal chemical reactions and mineral products will have differed from those observed at the more felsic Chicxulub. However, differences in rock composition (i.e. mafic vs. felsic target rocks) are predicted to be less consequential for hydrothermal system duration than the overall physical properties of the structure, such as impact-induced fracturing and modifications to porosity and permeability observed at Chicxulub. Additionally, Chicxulub provides a unique sample suite recovered through drill cores and has not undergone significant post-impact heating or modification unrelated to the impact event. Although buried on land and submerged offshore, International Ocean Discovery Program (IODP) and International Continental Scientific Drilling Program (ICDP) Expedition 364 at Site M0077 (21.45° N, 89.95° W)^21,22^ has provided access to previously unavailable samples for study. One of the main objectives of Expedition 364 was to increase understanding of the impact generated hydrothermal system^21^. The recovered Core M0077A revealed intensely altered rocks demonstrating significant post-impact hydrothermal activity (Fig. 1)^20,22,23–27^.

An extensive hydrothermal system at Chicxulub has been known for decades from chemical and mineral alteration detected in rocks of previous drilling programmes^17,28–32^. In Core M0077A, hydrothermal alteration is evident from the presence of Na-dachiardite, heulandite, analcime zeolites, secondary clay, calcite, alkali felspar overgrowths, andradite garnet, chlorite, and other phases as described in previous work^20,24^. These hydrothermal minerals cross-cut shock deformation features and overprint impact lithologies^20,24^. These findings support previous observations of an extensive and long-lived hydrothermal system in the Chicxulub structure, interpreted to be a result of the impact.

Rowe et al.^31^ determined a minimum hydrothermal lifetime of 300 thousand years (kyr) based on palaeomagnetic and stratigraphic constraints on the age of hydrothermal deposits in the Yaxcopoil-1 drill core. Numerical simulations of hydrothermal activity at Chicxulub^33^ suggested that it took 1.5–2.3 Myr to cool to 90 °C at 1 km below the surface, and it would have taken at least twice as long to cool below the thermophilic window (50–100 °C). The authors considered this to be a conservative estimate, and one of the main goals of Expedition 364 was to search for evidence that could empirically ground truth those simulations^21^.

Studies of hydrothermal minerals from site M0077A^20^ show that the Chicxulub hydrothermal system maintained temperatures of ≥250 °C for between 150 and 500 kyr. This conclusion is based on the normal-polarity magnetic signal preserved in the impact-melt breccias. Chicxulub formed during a reversed-polarity interval (Chron 29r), and the first switch to normal polarity occurred ~200 kyr later^20,34^. In order for the rocks to record that normal polarity, they must still have been hot enough to reset their magnetic signal—much hotter than the ~50 °C predicted at that time by the thermal model of Abramov and Kring^33^. This result implies that the hydrothermal system lasted substantially longer than the ~2 Myr previously estimated, supporting the authors’ view that their model was conservative^33^. Our new ^40^Ar/^39^Ar ages presented here further strengthen this conclusion, indicating a minimum lifetime of ~8 Myr.

The longevity of impact-induced hydrothermal systems in general is poorly constrained, and has been investigated using a variety of techniques: numerical simulations (e.g. Haughton^35^, Kärdla^36^, Sudbury^33^, Vargeão^37^, Vista Alegre^37^, Vredefort^38^, and various model craters^39^), estimates of conductive cooling of the crater (e.g. Manicouagan^40^, Manson^41^, Ries^42^), estimates based on organic geochemical biomarkers (Haughton^43–45^), stratigraphic correlation (Ries^46^), and comparison with other structures of similar size (Kärdla^47^). Only two systems have thus far been constrained by radio-isotopic measurements: Sudbury^48^, and Lappajärvi^49,50^.

At Sudbury (~250 km diameter, similar in size to Chicxulub), ^207^Pb/^206^Pb titanite ages indicate temperatures of ~350 °C up to ~4 Myr post-impact^48^; however, the data lack the precision to differentiate an apparent hydrothermal age (1848.4 + 3.8/−1.8 Ma, titanite ^207^Pb/^206^Pb^48^) from an impact age (1849.53 ± 0.21, zircon U-Pb^51^). The same paper estimates a likely duration of hydrothermal activity to be on the order of tens to thousands of years, based on calculations of conductive cooling^48^. Subsequent numerical simulations suggest that the hydrothermal system could have remained active for hundreds of thousands to several million years, depending on the permeability of the host rock^3^.

At Lappajärvi (~25 km diameter), ^40^Ar/^39^Ar K-rich feldspar and U-Pb zircon measurements suggest a hydrothermal duration of ~600 kyr to ~1.6 Myr^49,50^. This contrasts with theoretical estimates of the duration of the hydrothermal system at the similar-size Nördlinger Ries and Haughton impact structures of up to tens of thousands of years^42,52^. However, these latter estimates were based on calculations of conductive cooling of a melt sheet only, not accounting for convection of fluids or residual heat from the displaced rocks in the central uplift. This order of magnitude difference between measured and estimated duration is on a par with the lifetime measured in this work compared to previously simulated durations at Chicxulub. No previous models or measurements have suggested hydrothermal activity lasting longer than ~2 Myrs at any impact structure, though several authors have stated they consider their estimated durations to be conservative.

These results cover a range of possible durations from 10s kyrs to several million years for the ~200 km diameter Sudbury structure and 10s kyrs to ~1.6 Myr for the ~25 km diameter Lappajärvi structure. Whilst size is not the only factor in duration, one would expect the order of magnitude larger Sudbury structure to have a substantially longer-lived system, further indicating a substantive discrepancy in the current state of knowledge. These discrepancies serve to highlight how poorly constrained hydrothermal durations at impact structures are and emphasise the need for more robust empirical data.

In this work, we investigate the duration of hydrothermal activity at the Chicxulub impact structure, where impact melt rocks contain K-rich feldspar overgrowths mantling quench crystallised plagioclase feldspar. The textural relationship of these two feldspar phases indicates that crystallisation of the K-rich feldspars is related to the hydrothermal system. K-rich feldspars are ideal targets for ^40^Ar/^39^Ar geochronology of hydrothermal activity because their argon systematics are well understood in comparison to other K-bearing hydrothermal phases such as clays.

^40^Ar/^39^Ar geochronology of Chicxulub impact melt rocks yielded dates ranging from 58 to 66 Ma, which we interpret to represent a protracted but discrete period of mineral formation over ~8 Myr after the impact event. Numerical simulations were used to explore the parameter space that could yield ~8 Myr of hydrothermal activity. Together, these data are taken to indicate that hydrothermal activity persisted for a minimum of ~8 Myr, which is approximately four times longer than previously estimated by numerical simulations, palaeomagnetism, and petrographic interpretations at Chicxulub, and is the longest documented duration for an impact structure on Earth.

## Results and discussion

### Sample descriptions

We analysed four samples of impact melt rock from three core depths: 706.4, 735.0, and 756.0 mbsf (metres below sea floor) (Fig. 1, Supplementary Table A-1). All three samples contain plagioclase crystals with skeletal and swallowtail textures. Samples CHX 706.4 (*IODP sample name: 364_77_A_ 80R2_W_ 46.0-50.0*) and CHX 735.0 (*364_77_A_ 92R1_W_ 21.0-24*.0) are dominated by quench-crystallised plagioclase crystals mantled by K-rich feldspar overgrowths (Fig. 1D). Sample CHX 756.0 (*364_77_A_ 98R3_W_ 83.0-86.0*) comprises two distinct lithologies (CHX 756.0A and CHX 756.0B). Sample CHX 756.0A is similar to CHX 735.0: quench-crystallised plagioclase mantled by K-rich feldspar overgrowths (Fig. 1E). Sample CHX 756.0B is dominantly quench-crystallised plagioclase surrounded by pore space (Fig. 1F).

Petrography reveals the order of crystallisation of K-bearing phases: plagioclase formed by quenching of the impact melt and K-rich feldspar formed later as  overgrowths. Swallowtail and skeletal textures were visible in all plagioclase, indicating rapid crystal formation and cooling below ~800 °C^53^ (See Supplementary Note A for further descriptions). We interpret the plagioclase formation mechanism to be quenching of impact melt, and as such, the age of the plagioclase should record the age of the Chicxulub impact event.

We interpret the K-rich feldspar overgrowths as authigenic, which is supported by their non-luminescence in cold cathodoluminescence imaging, which is characteristic of authigenic feldspar^54^. The K-rich feldspar overgrowths observed here are similar to secondary mineral assemblages formed during post-impact alkali metasomatism in impactites observed at other impact structures, including Ries, Rochechouart, and in other locations at Chicxulub (e.g. refs. ^24,32^). For example, authigenic (<200 °C) feldspar growth concurrent with, or shortly after, zeolite formation was reported by Simpson et al.^24^ higher up in the M0077A core, in the breccias overlying the melt rock (with alteration increasing towards the melt rock contact). Additionally, veins of K-rich feldspar and albite were reported from the M0077A core, indicating temperatures of >250 °C^20^ and relatively high temperature K-metasomatism in the form of veins with biotite, chlorite, and quartz have been described in samples from Yaxcopoil-1^32^.

### ^40^Ar/^39^Ar geochronology

Due to the small crystal size and complex texture of intergrown plagioclase and K-rich feldspar, separating the two phases during sample preparation was not possible, such that all analysed samples were a mixture of plagioclase and K-rich feldspar in varying proportions. Based on visual inspection, we interpret the argon budget of samples CHX 706.4, 735.0, and 756.0A to be dominated by the authigenic K-rich feldspar overgrowths. The argon budget of sample CHX 756.0B is dominated by plagioclase feldspar, though we cannot exclude the possibility that some authigenic feldspar was included in these analyses. We attempted to separate sample CHX 756.0 into its two lithologies (A and B) prior to crushing for ^40^Ar/^39^Ar analyses. However, due to the heterogeneous nature of the sample and the irregularity of the contact(s) between these lithologies, we did not achieve a perfect separation, so small portions of subsample CHX 756.0A were likely included in subsample CHX 756.0B and vice versa (see Supplement A).

^40^Ar/^39^Ar step heating analyses yielded the following results. Sample CHX 706.4 did not yield any plateau ages (Supplementary Figs. B-7 and B-8); however, the four integrated ages range from 47.3 to 50.6 Ma. The discordant age spectra and poor statistical metrics indicate that the argon isotopic system has been disturbed and is therefore not yielding robust ages.

**Fig. 2:**
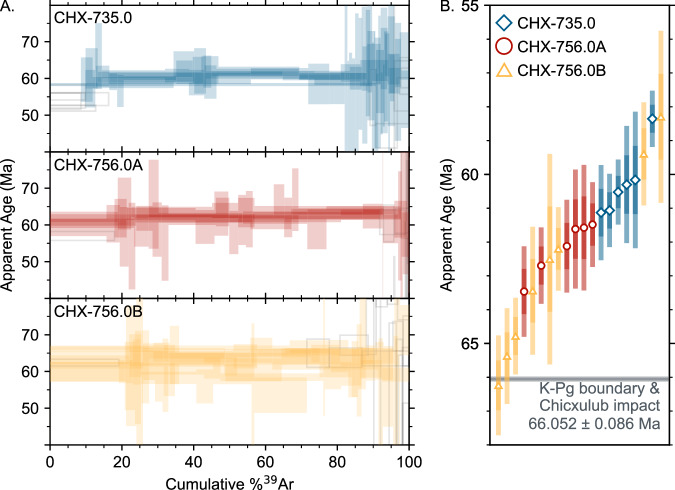
^40^Ar/^39^Ar results. **A** Age spectra of 22 step-heating analyses from 3 impact melt rocks (CHX 735.0, 756.0A, and 756.0B) that yielded plateau ages. Steps included in the plateau are filled; steps not included in the plateaus are unfilled grey outlines. **B** Ranked age plot. Individual plateau ages are represented by error bars, symbol and colour-coded to the sample name. 1*σ* bars are solid, 2*σ* are semi-transparent. The plot shows that the age span from time of impact (66 Ma) to ~58 Ma comprises all three samples, and is not 3 samples with 3 distinct ages, nor a spread within a single sample. Individual age spectra and isotope correlation plots can be found in Supplementary Figs. B-1 and B-6.

**Fig. 3:**
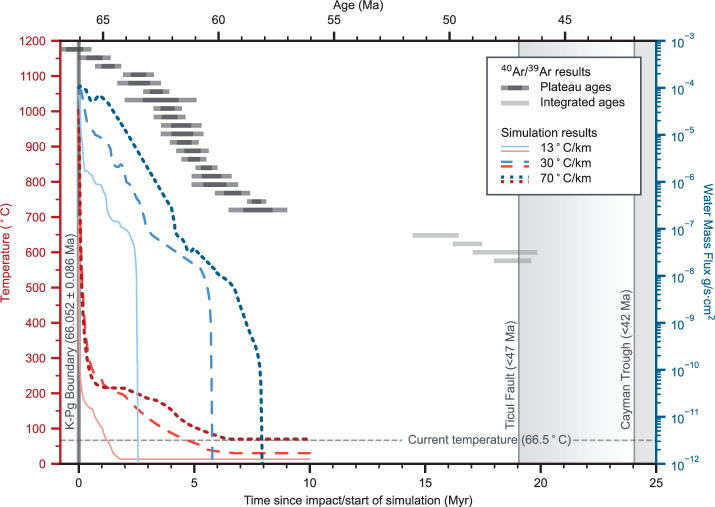
Summary plot of results from numerical simulations of the hydrothermal system, and ^40^Ar/^39^Ar age data from authigenic feldspar and impact melt rocks from Chicxulub. Vertical lines display the age of the K-Pg boundary and Chicxulub impact (66.052 ± 0.086 Ma, 2*σ*^55^), the onset of the Ticul Fault zone in the Middle Eocene (<47 Ma, McClain 1997 as reported by refs. ^63,64^), and the opening of the Cayman Trough in the late Eocene (<42 Ma)^66^. Dashed horizontal line shows the current temperature at the base of Hole M0077A, which was ~700 m under the surface 66 Ma (66.5 °C^68^). Temperature vs. time (red, left axis) and water mass flux vs. time (blue, right axis) for three HYDROTHERM simulations with three ending geothermal gradients (13 °C/km, 30 °C/km, and 70 °C/km). Simulations ran for 10 Myr post-impact. Temperature and water mass flux displayed here were taken from an observation point at a depth of ~1 km below the sea floor, similar to the base of Hole M0077A. The 13 °C/km simulation is a reproduction of the Abramov and Kring (2007) simulation outputs^33^. Temperatures in the new simulations (30 °C/km and 70 °C/km) do not return to background levels until 6–7 Myr after the impact, whereas the 13 °C/km model returns to background levels after ~2 Myr. Similarly, in the new simulations, water mass flux is negligible after 6–8 Myr, in contrast with the 13 °C/km model, where water mass flux is negligible after 2.5 Myr. The plateau ages generated from impact melt rocks in this work (black horizontal bars) define a spread of ages from 66 to 58 Ma. The youngest ages in the cluster of plateau ages are coincident with the time when temperature and water mass flux return to background levels in the 70 °C/km simulation, which is also the simulation that returns a temperature at 1 km depth closest to the temperature measured during Expedition 364. The integrated ages (grey horizontal bars) spread down to ~47 Ma. The integrated ages are not statistically significant (no plateaus, not internally reproducible); however, they approach the age of the Ticul Fault zone, suggesting that there may be a relationship between those disrupted ages and the opening of the Ticul Fault; however, the data are not robust enough to define a causative relationship.

Samples CHX 735.0, 756.0A, and 756.0B yielded 22 plateau ages ranging from 58.3 ± 1.3 Ma (2*σ*) to 66.24 ± 0.74 Ma (2*σ*) (Figs. 2 and 3 Supplementary Figs. B-1 and B-6, and Supplementary Table B-3). The relatively large plateau age uncertainties are due to low K content and the small volume of materials analysed (each aliquot consisted of one or three rock fragments, each 250–500 μm in diameter). Sample CHX 756.0B (quench-crystallised plagioclase surrounded by pore space) yielded eight plateaus, ranging from 58 to 66 Ma. The oldest ages measured in this sample are indistinguishable from the K-Pg boundary and the Chicxulub impact (66.052 ± 0.086 Ma, 2*σ*^55^, Fig. 2). Samples CHX 735.0 and 756.0A (quench-crystallised plagioclase with K-rich feldspar overgrowths) yielded 12 plateaus ranging from 58-64 Ma, all younger than the Chicxulub impact (Fig. 2). To establish a minimum duration of hydrothermal activity we calculate the difference between the age of the impact (66.052 ± 0.086 Ma) and the youngest analysis (Run ID 93303-10: 58.3 ± 1.3 Ma), yielding 7.8 ± 1.3 Myr, which we report as ~8 Myr.

Sample CHX 756.0B defined the oldest and two of the youngest ages in this study (Fig. 2). Due to the uneven boundary between samples CHX 756.0A and B, and challenges in visually differentiating them in hand sample, we could not attain perfect physical separation during sample preparation. As a result of that challenge and in light of the data, we interpret the two youngest ages from 756.0B to contain more authigenic K-rich feldspar than the rest of sample 756.0B, and therefore to have their radiogenic ^40^Ar budget dominated by younger hydrothermal phases rather than impact-melt generated plagioclase. The imperfect separation implies mixing between the age groups, and we can see mixing in the overlap between the two youngest CHX 756.0B ages and the CHX 735.0 and CHX 756.0A ages. This observation is unsurprising given the complex intermixing of textures and phases observed in these samples.

Because it was not possible to physically isolate plagioclase from K-rich feldspar during sample preparation, resulting in aliquots with a range of modal proportions of K-bearing phases, we conducted diffusion experiments to constrain the thermal sensitivity of these multi-phase samples. Argon diffusion experiments were conducted on impact melt rock fragments from the same population of fragments utilised for ^40^Ar/^39^Ar analysis. Models of diffusion data demonstrate that >99% of argon is retained below sustained temperatures of 150–175 °C in sample CHX 735.0 and 165–225 °C in sample CHX 756.0A (Supplement C). Due to limited sample availability, we could not perform argon diffusion experiments on CHX 756.0B, but the large range of plateau ages suggests that the quantitative retention likely occurs across the range of temperatures defined by the other two samples.

A key observation from modelling our diffusion experiment results is that the more retentive mineral phase also dominates the budget of radiogenic Ar in our samples (87% Ar in CHX 735.0, 85% in CHX 756.0A). We infer that this retentive phase is the K-rich feldspar overgrowths, consistent with previous studies of occurrences of authigenic K-rich feldspar in orogenic samples^56,57^ as well as diffusion studies on pure mineral phases^58^. The plagioclase cores are less retentive to Ar but also constitute a much smaller portion of the radiogenic Ar budget (≤15%) due to their lower K content. This observation has two important implications. First, while there may be some diffusive loss from the older plagioclase cores during hydrothermal activity, the low overall radiogenic Ar budget of plagioclase means that any diffusive Ar loss may not be resolved in our data, particularly considering the coarse step resolution of our age spectra at low cumulative ^39^Ar releases. As a result, argon loss from the plagioclase cores has a minimal impact on plateau ages. Second, the observed range of plateau ages spanning ~8 Myr post-impact indicates that K-rich feldspar overgrowths formed throughout a protracted period of hydrothermal activity. Moreover, the formation of the K-rich feldspar overgrowths must have been both spatially and temporally heterogeneous and would have occurred over a range of temperatures during this hydrothermal activity, consistent with previous studies of authigenic K-rich feldspar growth in hydrothermally active settings^54^.

Intriguingly, the integrated ages from CHX 706.4 are similar to previously unpublished ^40^Ar/^39^Ar ages—collected in 1990—from the Y-6 drill core (~65 km away from hole M0077A): impact melt rocks yielded 5 plateau ages covering an age range of ~50–55 Ma. These ages were previously difficult to interpret, but in light of the current work, they likely indicate that hydrothermal activity was occurring over a geographically broad region, which is a known characteristic of impact-generated hydrothermal systems. This suggests a potentially much longer duration, up to 16 Myrs; however, without corroboration and possibly re-analysis, it remains difficult to fully interpret these Y-6 drill core results. The data do, however, point towards the need to further investigate hydrothermal duration at locations beyond the peak ring.

### Numerical simulations

Previous numerical simulations of hydrothermal activity at Chicxulub^33^ suggested that it took 1.5–2.3 Myr to cool to 90 °C at 1 km below the surface, and it would have taken at least twice as long to cool below 50 °C, thereby exiting the thermophilic window of 50–100 °C. We therefore need to further explore the sensitivity of numerical simulations to changes in physical parameters (e.g. permeability, initial heat flow, final geothermal gradient, etc.) that allow at a minimum of ~8 Myr of hydrothermal activity.

We conducted new numerical simulations to examine the different boundary constraints required for at least ~8 Myr duration of hydrothermal activity at Chicxulub. Here, we consider Chicxulub hydrothermal activity to cease when temperatures approach ambient ground temperatures, and the water flux decreases by 5 orders of magnitude. We used version 3.2.0 of the U.S. Geological Survey (USGS) HYDROTHERM programme^59,60^. All input parameters and boundary conditions of the simulations are included in Supplement D. Our simulations followed a similar methodology to previous work^3,4,33^, but are different in that we include measured physical properties from Hole M0077A^23,61^, and explore a range of initial geothermal gradients. Seismic reflection and refraction data provide gross constraints on the overall structure of the crater interior^62^, but may not reveal fine-scale heterogeneities (tens of metres across or less) that may affect fluid flow (see Supplement D for discussion). To approximate the inherent complexity of a natural system, we utilised a more heterogeneous juxtaposition of subsurface crater lithologies in our simulations than what is inferred from seismic measurements. This approach is not intended to reflect the true crater structure; however, our goal is not to infer exactly where subsurface flow occurred, but rather to broadly investigate how changes in physical parameters modify the overall hydrothermal system lifetime at the order-of-magnitude level.

The results of three simulations that return to final geothermal gradients of 13 °C/km (in agreement with previous simulations^33^), 30 °C/km (global crustal average), and 70 °C/km (approximate current temperature at base of Hole M0077A) are presented in Fig. 3 alongside the ^40^Ar/^39^Ar ages and regional post-impact geological events such as activity within the Ticul Fault Zone (<47 Ma^63–65^) and the Cayman Trough (<42 Ma^66^). The 13 °C/km model yielded a duration of hydrothermal activity of ~1–2.5 Myr, which matches that determined by the only previous numerical simulations of hydrothermal activity at Chicxulub^33^. Those initial simulations by Abramov and Kring used, as input, the post-impact thermal state generated by a previously published impact simulation^67^. Abramov and Kring^33^ noted that a gradient of 13 °C/km was a low value, producing a conservative estimate for the duration of hydrothermal activity, and that, in practice, hydrothermal activity may have persisted longer. We considered it important to replicate those results in this study in order to bracket our interpretations. As in the previous work, the duration(s) based on current modelling can be considered conservative; natural post-impact hydrothermal systems may have persisted for longer.

The 70 °C/km model that uses measured temperature from Hole M0077A agrees best with the argon age data. In this model, from 20 kyr to 3 Myr post-impact, the hydrothermal fluid is frequently recharged by overlying seawater that flows down through the impact breccia. Recharge ceases after ~4 Myr. After 6.1 Myr, the water mass flux in the system is four orders of magnitude lower than at the start of the simulation (~10^−4^ to 10^−8^ g/s cm^2^), and by 8 Myr fluid flow has effectively ceased (~10^−10^ g/s cm^2^). Temperatures at 1 km depth in the peak ring are 90 °C after 5.5 Myr, and after 6.4 Myr have returned to 70 °C, close to the modern-day temperature measured at the bottom of Hole M0077A (66.5 °C^68^, Fig. 3). Detailed results of the simulations are in Supplement D.

Previous work has demonstrated that permeability is a key constraint in numerical simulations of hydrothermal systems^3,4,33^. Permeability constraints used in our simulations are therefore underpinned by permeability of analogous materials^69–73^ and the porosity values measured during Expedition 364^23^ (20–35% in impact breccia (suevite), 19–22% in impact melt rock, and 8–13% in granite).

### Linking numerical simulations with age data

The ^40^Ar/^39^Ar data demonstrate that impact-generated hydrothermal activity persisted for ~8 Myr. This duration coincides with fluid flow in the numerical simulation ending after ~8 Myr. The argon diffusion experiments reveal the presence of multiple diffusion domains (MDDs) within each mineral phase, characterised by different degrees of retentivity. The minimum closure temperatures for the lowest retentivity domains in the K-rich feldspar overgrowths, which represent a minor portion of the total argon budget, are 175 and 210 °C in CHX 735.0 and 756.0A, respectively. Together with the hydrothermal simulations, which indicate that temperatures were lower than these closure temperatures over most of the duration of hydrothermal activity, the argon diffusion results suggest that the K-rich feldspar overgrowths formed and retained their radiogenic argon throughout the duration of hydrothermal circulation. We hypothesise that authigenic K-rich feldspar ceased growing as the water flux through the system declined, changing from flushing to stagnant. This is evidenced by a series of discrete plateau ages that span a period of ~8 Myr, composed of internally reproducible steps, and is supported by the results of numerical simulations, which suggest negligible fluid flow after ~8 Myr.

### Other nearby geological events

We interpret the ^40^Ar/^39^Ar data reported here to record post-impact hydrothermal processes related to the Chicxulub impact, rather than any subsequent regional geologic process that could have induced post-impact argon loss in the samples. The chronologically and geographically nearest events occur at least 20 Myr after the Chicxulub impact.

**Fig. 4:**
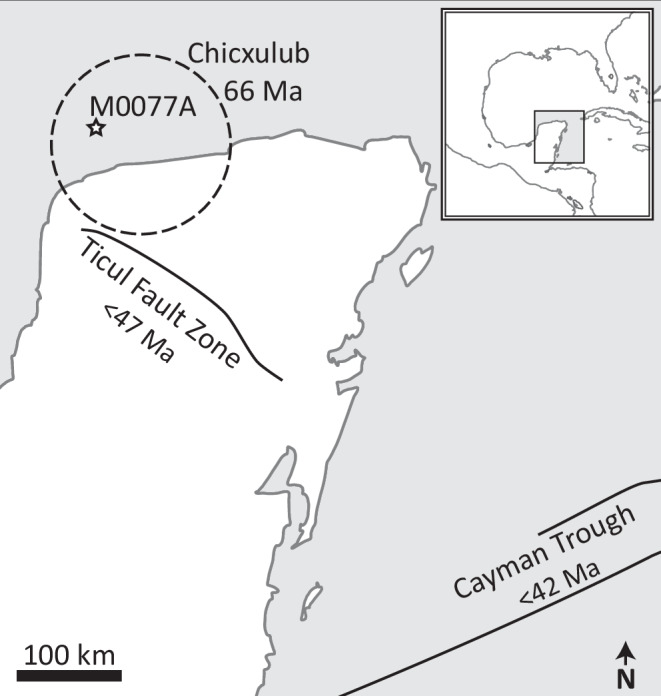
Map of the area surrounding the Chicxulub impact structure showing the two nearest tectonic events—the Ticul Fault zone^63–65^ and the Cayman Trough^66^. Samples in this study originate from hole M0077A as indicated by a star. Basemap and inset from Wikimedia (https://w.wiki/M2DR).

Only two major Paleogene (post-impact) tectonic/thermal events are known within ~600 km of the Chicxulub impact structure: the Ticul Fault Zone—first active during or after the Middle to early Late Eocene^63–65^ (<47 Ma); and the Cayman Trough, first active in the late Eocene^66^ (<42 Ma, Fig. 4). Because these events occurred >10 Myr after the plateau ages measured in this study, they can be safely ruled out as a cause of the post-impact plateau ages. Despite relative proximity (within ~100 km of hole M0077A), we do not consider activation of the Ticul Fault zone to have reset the plateau ages from a Chicxulub age (66 Ma) to the ~8 Myr spread measured, because we obtained discrete plateau ages. If the samples had experienced partial resetting of the argon system, they would show young apparent ages at the early/low temperature release steps in age spectra plots rather than concordant age steps as define a plateau. Therefore, the plateau ages we recovered demonstrate within-sample reproducibility, not a range of individual step ages across a window from 66 to 58 Ma. The Ticul Fault Zone cross-cuts Middle to Early Late Eocene deposits, giving it an approximate age of <47 Ma (McClain 1997 as reported by refs. ^63,64^), which is close in age to the discordant data produced by sample CHX 706.4 (youngest integrated age of 47.3 ± 0.8 Ma). Unfortunately, given the poor quality of the data from sample CHX 706.4 and the large age window of the Ticul fault, nothing can currently be concluded from the coincidence of those two apparent ages.

Additionally, neither diffusion nor step-heating experiments indicate substantial diffusive loss of ^40^Ar from low-retentivity domains, which would manifest as younger apparent step ages during the initial stages of step degassing, with ages increasing with increasing ^39^Ar release fraction. Diffusion experiment results indicate quantitative argon retention below 150–225 °C on geologic timescales (e.g. Supplementary Figs. C-3 and C-4), temperatures which we do not expect the samples to have exceeded during their geologic history after hydrothermal activity at Chicxulub ceased.

### Broader implications of this work

Impact-generated hydrothermal systems are known to be heterogeneous in their extent, composition, fluid evolution, and duration^10,20,24,32,33,35,52^. This inherent heterogeneity, coupled with the limited availability of Chicxulub samples and the targeted analyses required for radio-isotopic age determinations, means that we have empirically characterised hydrothermal activity and longevity for only a limited portion of the crater (in this case, a small portion of the peak ring). The duration reported here may therefore be somewhat local, governed by the crater structure and local lithological/petrophysical properties. Hydrothermal activity may have lasted longer in the peak ring relative to the rest of the crater, due to the high porosity and permeability, and proximity to hot uplifted rocks. This suggests that peak rings in particular may be more suitable for the creation of habitable environments, but this remains speculative without more in-depth characterisations of the extent, continuity and duration of the hydrothermal system at different locations throughout the structure.

Despite the local nature of our sampling and analyses, our results raise important questions about our understanding of post-impact processes: if prior models underestimated the duration of hydrothermal activity, which of the geologic processes involved were underappreciated? Our simulations suggest that accounting for complex geology and incorporating a wider range and better constrained physical properties in the model—such as the higher porosity measured in the Expedition 364 samples compared to previous model estimates and a geothermal gradient representative of the global average or the gradient at the site—is critical.

The cooling of large impact structures is clearly more complex and perhaps more heterogeneous than previously believed, indicating that more thermochronometric data are necessary to unravel the thermal history at Chicxulub and other impact structures. The previous results from other impact structures couple with previously published (U-Th)/He data from Chicxulub^74,75^, together with our new ^40^Ar/^39^Ar results from Chicxulub, lead to the conclusion that we have been underestimating the duration of post-impact hydrothermal activity at impact craters on Earth. To accurately characterise impact-generated hydrothermal systems requires coupling of detailed numerical simulations with precise and accurate geochemical, isotopic, petrologic, and petrophysical observations. This integrated approach has revealed an extended duration of hydrothermal activity at Chicxulub that is at a minimum of ~8 Myr, four times longer than previously estimated. Chicxulub is still relatively small (200 km) compared to the impact basins expected on early Earth and observed on other planetary bodies (1000s km), it is therefore possible that these larger impacts could have created even longer-lived hydrothermal systems and, hence, could have been able to maintain the temperatures and fluid flux required for habitable environments for a minimum of several million years.

## Methods

### Optical microscopy

Transmitted light microscopy of polished thin sections was conducted on Zeiss Axioplan and Olympus BX41 petrographic microscopes. Image capture for the Zeiss Axioplan used a Nikon DS-Fi1 camera (2/3 inch, 5.24 megapixel CCD, 2560 × 1920 recording pixels) with Nikon NIS-Elements F3.0 software. Image capture for the Olympus BX41 used an Olympus DP25 camera (2/3 inch, 5.24 megapixel CCD, 2560 × 1920 recording pixels) with cell^B v2.8 software.

### Scanning electron microscopy

Backscatter electron imaging and qualitative electron dispersive X-ray spectroscopy (EDS) were conducted on a field emission Zeiss Sigma SEM at the Geoanalytical Electron Microscopy (GEMS) facility, University of Glasgow. EDS used the Oxford Instruments AZTEC software and a silicon drift X-ray detector. Operating conditions for imaging and analysis of carbon-coated thin sections were: high vacuum mode, probe current = 1 nA, accelerating voltage = 20 kV, working distance = 8.5 mm.

### ^40^Ar/^39^Ar analyses

Samples were prepared by crushing cut portions of individual pieces (roughly 1–2 cm^3^) of impact melt rock in a disc mill. The crushed material was sieved, and the 250–500 µm size fractions were rinsed until clear in water in an ultrasonic bath, and were then subjected to acid leaching, magnetic separation, and hand-picking.

All impact melt rocks from Chicxulub were leached ultrasonically for 5 min in 25% HNO_3_ (nitric acid), and subsequently rinsed in de-ionised water and dried overnight in an oven (~70 °C). The magnetic portions were then handpicked under a binocular microscope, looking for unaltered grains with few or no inclusions (Supplementary Fig. A-1).

After hand-picking, all samples were packaged into a 21-well aluminium irradiation disc, wrapped in aluminium foil, and sealed in a glass cylinder for irradiation.

International standard Fish Canyon Sanidine (FCs, 28.294 ± 0.072 Ma, 2*σ*^76^) was loaded symmetrically into the discs, adjacent to samples from Chicxulub. Additionally, crystals of IrZ sanidine were loaded symmetrically into the discs to act as a fluence monitor (IrZs, 66.043 ± 0.086 Ma, 2*σ*^77^). Samples were irradiated by fast neutrons for 50 h at 1000 kW in the Cadmium-Lined in-Core Irradiation Tube (CLICIT) facility of the Oregon State University TRIGA Reactor. The 50-h irradiation took place over 9 separate irradiation sessions, with each session lasting between 4 and 6 h (see Supplement B for full irradiation schedule).

Samples were heated to a maximum of 225 °C during irradiation due to interaction with gamma rays. This temperature was confirmed with measurements conducted in 2009 and 2024 by OSU TRIGA staff, bracketing the time of our irradiations. Though our irradiations were 50 h in total duration, this was broken up into 9 separate irradiation periods of varying lengths, such that the amount of time spent at 225 °C would be substantially shorter—we estimate a maximum of 30 h. To assess the potential for radiogenic ^40^Ar loss during neutron irradiation, we calculated the expected fractional loss of ^40^Ar given the diffusion kinetics we obtained from the reactor-produced ^39^Ar. If we consider a 30-h-long heating event to 225 °C (a worst-case scenario, as the cooling between the 9 irradiation periods would further inhibit ^40^Ar loss), we predict 0.06% loss from the least retentive K-rich feldspar domain in CHX-7350 and 0.01% loss from the least retentive K-rich feldspar domain in CHX-7560A. As these are the least retentive domains and constitute a minority of the authigenic K-rich feldspar ^40^Ar budget, the total potential fractional loss of ^40^Ar across all domains is substantially lower. Therefore, loss of ^40^Ar during the neutron irradiation is insignificant and cannot explain the observed young apparent ^40^Ar/^39^Ar dates that we interpret to reflect continued hydrothermal activity for several million years after the Chicxulub impact.

After irradiation, samples were left for 8 months to allow short-lived radioisotopes produced during irradiation to decay.

All argon analyses took place at the NERC Argon Isotope Facility (AIF), which is hosted by SUERC - Centre for the Isotope Sciences) in East Kilbride, Scotland.

Aliquots of single grains were each loaded into individual wells in a 208-well steel laser pan. After loading the pan into the argon extraction line attached to a MAP 215-50 noble gas mass spectrometer, it was baked for 24–36 h at 100 °C to remove atmospheric contamination. Fish Canyon sanidine (FCs) and IrZ sanidine (IrZs) were analysed by total fusion using a CO_2_ laser. FCs and IrZs were used for *J* value determinations.

Following laser heating, extracted gas fractions were subjected to 300 s of purification with two SAES GP50 getters (one at room temperature and one at 450 °C). Ion beams were measured using a MAP 215-50 noble gas mass spectrometer in peak-jumping mode with a measured sensitivity of 1.13 × 10^−13^ mol/V (e.g. ref. ^78^). Gas extraction, purification, extraction line operation, and mass spectrometry were fully automated. Backgrounds were measured after every two unknowns, and mass discrimination was monitored by analysis of air pipettes after every five measurements.

Plateau ages were defined as containing a minimum of three contiguous steps overlapping at 2*σ* uncertainty, and comprising >50% ^39^Ar released^79^. Plateau ages were calculated using mean weighted by inverse variance, with plateau uncertainties calculated by standard error of the mean (*sem*), but if the MSWD was >1, then the uncertainty was calculated by: *σ* = sem*sqrt(MSWD).

All data were regressed and handled using the Berkeley Geochronology Centre software, MassSpec. ^40^Ar/^39^Ar ages were calculated using the decay constants and monitor ages (FCs) from the optimisation model of Renne et al.^76,80^. Mass discrimination values were determined using the atmospheric argon ratios of Lee et al.^81^, which have been independently verified by Mark et al.^82^.

### Argon diffusion experiments and thermal history constraints

In order to estimate the kinetics of argon diffusion in our samples, we performed a second set of step degassing experiments on samples CHX 735.0 and CHX 756.0A. For each experiment, we placed 5-6 irradiated sample fragments into a pure Pt packet and sequentially heated the packet using a 70 W diode laser in a PID feedback control loop with a calibrated optical pyrometer (accurate to within ± 6 °C for temperatures between 470 and 1100 °C). We purified the gas and measured argon isotopes released during each heating step on the ARGUS V multi-collector sector-field mass spectrometer in the AIF at SUERC^83^. Because we performed this second set of step degassing experiments more than 1 year after the samples were irradiated, we were unable to correct ^40^Ar measurements for trapped atmosphere. We therefore do not report step ages for these experiments, and instead use the reactor-induced ^39^Ar to calculate diffusivities^84^ (Supplementary Tables C-1 and C-3). We considered a step heating experiment completed when ^39^Ar signal intensities were comparable to those measured in subsequent blank measurements.

Because the fragments we analysed were whole rock chips, rather than individual mineral separates, concurrent degassing of multiple K-bearing phases with distinct argon diffusion kinetics manifested as nonlinearity in Arrhenius plots (Supplementary Figs. C-1 and C-3). Given this, we utilised the MDD modelling framework^85,86^, as implemented by Boehnke et al.^87^, which allows for multiple activation energies (a requirement when more than one K-bearing phase is present) to interpret the observed argon diffusion behaviour. We allowed model activation energies (*E*_*a*_) to vary between 125 and 250 kJ/mol, consistent with the range of activation energies observed for most feldspars^58^, and frequency factor (*ln*(*D*_*0*_/*a*^2^)) to vary between 0 and 45. We performed model searches with two activation energies representing argon diffusion from two phases (plagioclase and K-rich feldspar), with at most ten diffusion domains per model, and found the models that minimised the reduced *χ*^2^ misfit statistic between observed and predicted diffusivities (Supplementary Table C-4). Because plagioclase undergoes a structural transformation to monoclinic symmetry above 800 °C, we only calculate the model misfits for temperature steps of 800 °C and lower.

With these diffusion parameters, we explored the effects of simple, isothermal histories on the retention of argon in our samples^88^. Supplementary Figs. C-4 and C-6 show temperatures at which 1% and 99% argon loss will occur as a function of time for the best-fit 2-phase MDD models. Two important observations can be made from these fractional loss curves. First, even for hydrothermal circulation lasting more than 10 Myr post-impact, temperatures would need to be exceedingly high over this duration for complete ^40^Ar diffusive loss (>99% loss from all diffusion domains) and age resetting to occur (>350 °C for CHX 735.0, >430 °C for CHX 756.0A and CHX 706.4A). Even greater constant temperatures are required for a shorter-lived hydrothermal system (Supplementary Figs. C-4 and C-6). Second, these models can be used to constrain the maximum temperatures at which the samples would have experienced complete argon retention (<1% loss), either during or after heating associated with hydrothermal circulation. In the first 10 Myr after the Chicxulub impact, we predict maximum temperatures below which complete argon retention occurs in all diffusion domains of ~150, 165, and 190 °C for 7350, 7560A, and 7064A, respectively. Over the last 66 Ma, we predict maximum temperatures below which complete argon retention occurs of ~130, 150, and 175 °C for 7350, 7560A, and 7064A, respectively.

### Numerical simulations

For this study, we operated the updated version of the U.S. Geological Survey (USGS) HYDROTHERM 3 (HT3) software^59,60^ that provides the coupled equations and solutions for mass and energy conservation in a thermodynamic system. Further details on the equations of state used in this work are provided in the HT3 (version 3.2.0) user manual (USGS^59^). The updated version of the code can offer an innovative approach to the evolution of permeability and porosity of lithologies in the Chicxulub crater by incorporating governing boundary conditions for the fractures observed in Core M0077A. In our models, permeability and porosity decrease exponentially with depth, but the fractures observed within the sampling of the peak-ring and their assumed hydraulic conductivity exert a secondary control on hydrothermal flow. In parallel, we also follow the approach of Hayba and Ingebritesen^89^ in which permeability is also described as a function of temperature.

Algorithms addressing hydrothermal convection solutions treat rocks as continuous media. In these sets of simulations, we have included local changes in permeability and a higher level of discretisation to increase the complexity of the simulated domains. We incorporated fracturing (after Yang et al.^90^), sealing of fractures, fluid viscosity-density and fluid heat exchange fluxes as control input parameters discretely through numerical coding. We adopted the methodology of Yang et al.^90^ and resolved the coupled, time-dependent heat and fluid transport differential equations of the HT3 code by using the finite element method for fractured areas in the setting. Resulting input parameters were then introduced to the HT3 source code and its interactive version 3.2.0 to simulate the evolution of fluid flow in several impact-induced hydrothermal and volcanic settings. The numerical tests allowed us to proceed with the simulations by using the Corey-Cooley and the Corey functions of HT3. Several feasibility tests were performed to benchmark the simulation tests. For this reason, the Abramov and Kring^33^ Chicxulub model was also reproduced to validate the code. Multiple sets of simulations were produced for the specified grid in the presence and absence of fractures to assess the efficiency of the HT3 algorithm versus previous versions of the HT code. In addition, simulations of varying resolution (from 4000 cells to 20000 cells) were performed to compare the resulting durations of hydrothermal circulation. The validation results proved that fracturing can be successfully described through the permeability and porosity equations of the HT3 numerical code, as the newest version of HYDROTHERM (HT3.2) includes these modifications.

## Supplementary information

Transparent Peer Review file

Supplemental Materials

## Data Availability

The sample descriptions, images, ^40^Ar/^39^Ar step-heating data, argon diffusion data, and full HYDROTHERM parameters and results are available and preserved at Enlighten, University of Glasgow Repository via 10.5525/gla.researchdata.2272^91^.

## Code availability

HYDROTHERM is accessible via the USGS website: https://volcanoes.usgs.gov/software/hydrotherm/.
