# Supplementary material for: A long-lived impact-generated hydrothermal system at the Chicxulub impact structure
Source: Commun Earth Environ. 2026 Jun 9;7(1):470. doi: 10.1038/s43247-026-03618-5 (PMC13249567; doi:10.1038/s43247-026-03618-5)
Supplement: Supplementary file 2 — Supplemental Materials [file 43247_2026_3618_MOESM2_ESM.pdf]

Supplementary Materials

Supplementary Note A – Sample Descriptions

Materials

Samples of the Chicxulub impact melt rocks were collected from the IODP-ICDP Expedition 364 core, which was drilled in 2016. Samples were chosen based on apparent lack of alteration in hand sample, selecting for dark, well consolidated impact melt rocks. Polished thin sections were examined by optical and scanning electron microscopy to determine which samples would be best suited for $^{40}Ar/^{39}Ar$ analysis. Samples that were chosen had minimal alteration and inclusions/clasts in the groundmass. Table A-1 summarizes the selected samples, Figure A-1 shows images of the samples after sample preparation and picking.

Table A-1: List of samples from Core M0077A used in this project.

<table>
<tr><th>Location</th><th>Sample</th><th>Description</th><th>Depth (mbsf)</th><th>IODP sample name (364_77_A_ …)</th><th>Run ID</th></tr>
<tr><td rowspan="4">Chicxulub, Mexico (IODP-ICDP Expedition 364 Site M077A)</td><td>CHX-7064A</td><td>Dark grey microcrystalline impact melt rock</td><td>706.4</td><td>80R2_W_ 46.0-50.0</td><td>93294</td></tr>
<tr><td>CHX-7350</td><td>Dark grey microcrystalline impact melt rock</td><td>735.0</td><td>92R1_W_ 21.0-24.0</td><td>93302</td></tr>
<tr><td>CHX-7560A</td><td>Black microcrystalline impact melt rock</td><td rowspan="2">756.0</td><td rowspan="2">98R3_W_ 83.0-86.0</td><td>93292</td></tr>
<tr><td>CHX-7560B</td><td>Grey/brown microcrystalline impact melt rock</td><td>93303</td></tr>
</table>

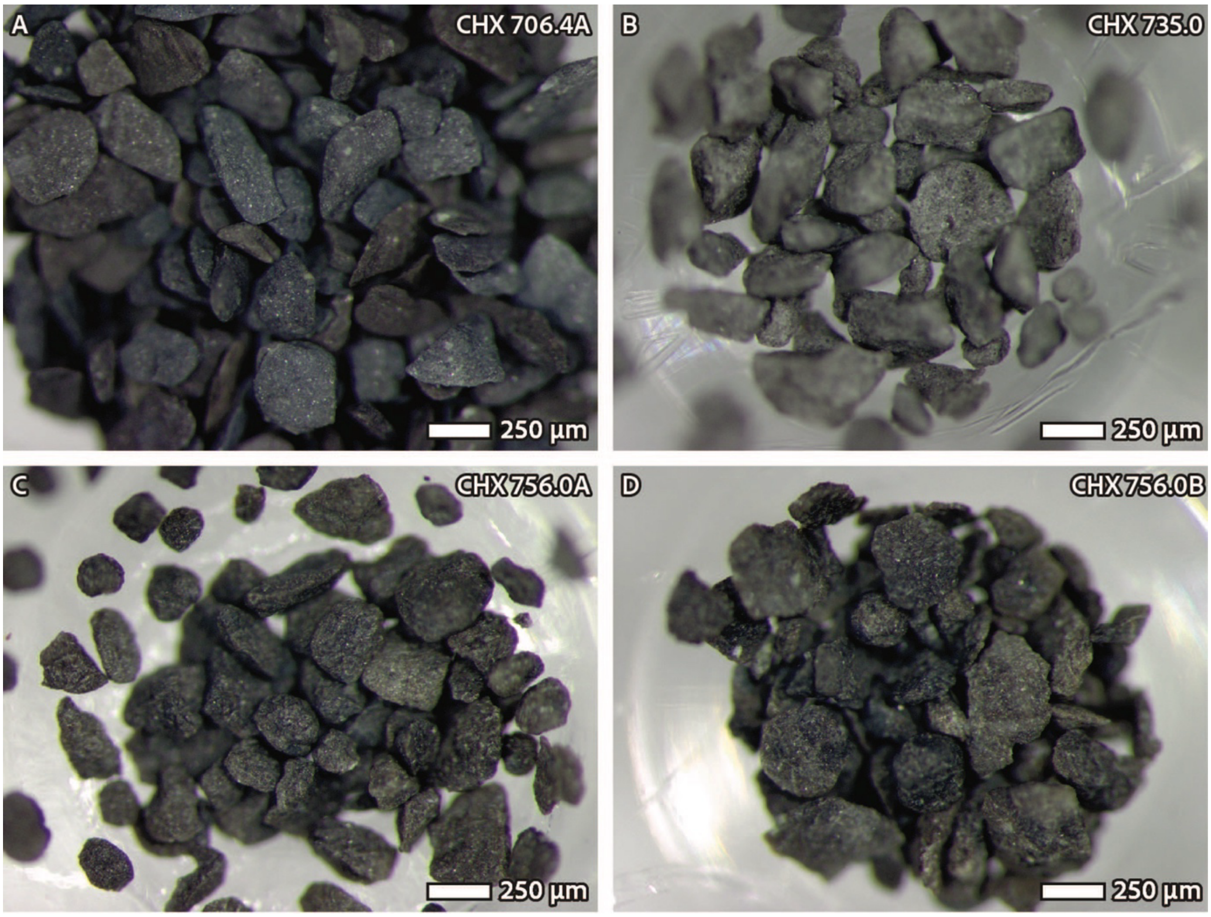

Figure A- 1: Impact melt rock separates. Sample separates for $^{40}Ar/^{39}Ar$ analyses after acid-leaching, magnetic separation, and hand-picking.

Petrography

Sample CHX 706.4

(IODP sample ID: 364_77_A_80_R_2_W_46.0-50.0)

CHX 706.4 samples the contact between an impact melt rock (CHX 706.4A, Run ID 93294) and a mafic lithology (either a large clast or an intrusion; CHX 706.4B; Fig. A-2).

The impact melt rock is dark grey to black in colour with numerous quartz clasts (~15-20% clasts; Fig. A-2A). In thin section, partial digestion of clasts is evidenced by rounded edges and alteration rims. Impact melt shows signs of flow as shown by variation in shades of brown in plane polarized light (Fig. A-2C, D) and variations in mean atomic number as revealed by BSE imaging (Fig. A-2E).

A microcrystalline texture is evident at high magnification and with the SEM. The groundmass is composed of lath-shaped plagioclase crystallites occasionally with swallow-tail terminations and/or skeletal texture (Fig. A-2F). The areas between the crystallites are either voids, or contain K-rich clays (Fig. A-2F). Clast content and evidence of flow are variable throughout the thin section, as is clay content. The contact between the mafic lithology and impact melt rock is lined by a zeolite (Fig. A-2G). Crystallites in the impact melt rock appear to be concentrated near this contact.

The mafic lithology (CHX 706.4B) is composed of plagioclase, pyroxene, and biotite. There is no evidence of flow, and crystal size is consistent throughout the sample with no variation between the contact with the melt rock and the interior of the clast (Fig. A-2H).

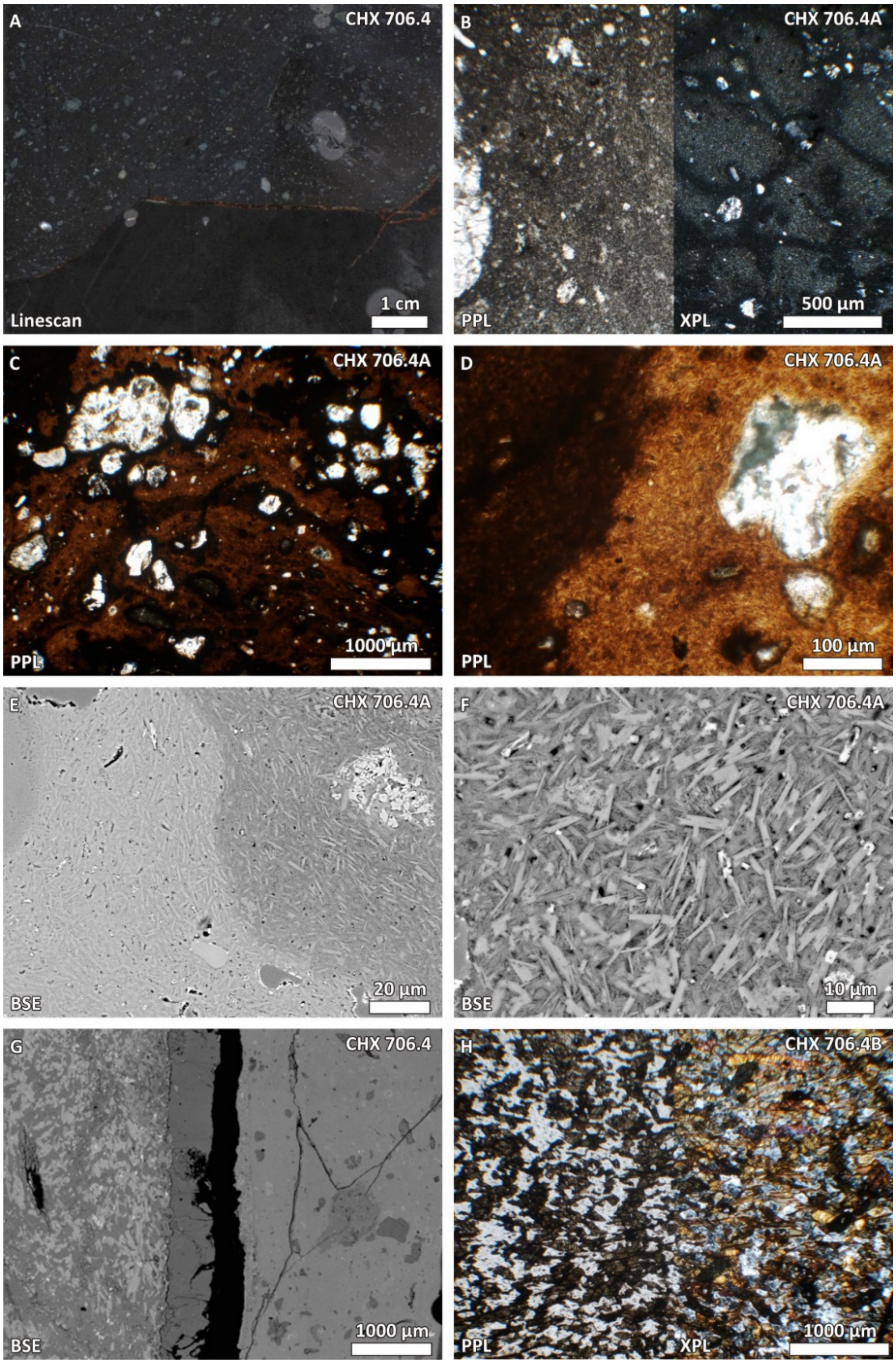

Figure A- 2: Linescan, transmitted light, and BSE images of impact melt rock sample CHX 706.4. A) Linescan image (photograph) taken of the core section from which sample 706.4 originates. The upper grey unit containing clasts is the impact melt rock (subsample 706.4A); the lower black, clast-free unit is a large basalt clast within the melt-rock unit (subsample 706.4B). B) Transmitted light photomicrograph of a representative portion of CHX 706.4A (melt rock). C) Transmitted light photomicrograph of flow textures in 706.4A (melt rock), highlighted by variable brightness. D) Magnification of an area with flow textures; microcrystallites are difficult to pick out. E) BSE image of an area showing flow textures; microcrystallites of plagioclase feldspar are visible as small lath-like bright areas; the colour difference is a result of higher porosity and clay content in the darker part of the image. F) BSE image of a magnified part of the groundmass highlighting plagioclase crystallites with some swallow-tail terminations indicative of quench

cooling. G) BSE image of the contact between the basalt clast (left) and the impact melt rock (right); the black band between them is a fracture filled with resin (formed during thin sectioning); the material between the basalt and the fracture appears to be zeolite. H) Transmitted light photomicrograph of the basalt clast. PPL = plane polarised light; XPL = between crossed-polarisers, BSE = backscatter electron image.

Sample CHX 735.0

(IODP sample ID: 364_77_A_92_R_1_W_21.0-24.0)

Sample CHX 735.0 (Run ID 93302) is a clast-poor impact melt rock. In hand sample the impact melt rock is black with areas of green highlighting alteration; it contains ~10% clasts (Fig. A-3A). In thin section, partial digestion of clasts is evidenced by rounded edges and alteration rims (Fig. A-3B).

A microcrystalline texture is evident at high magnification and with an SEM. The groundmass is composed of densely packed crystals of plagioclase with Mg-rich clay pseudomorphs of an acicular-to-lath-like mineral (Fig. A-3C, D).

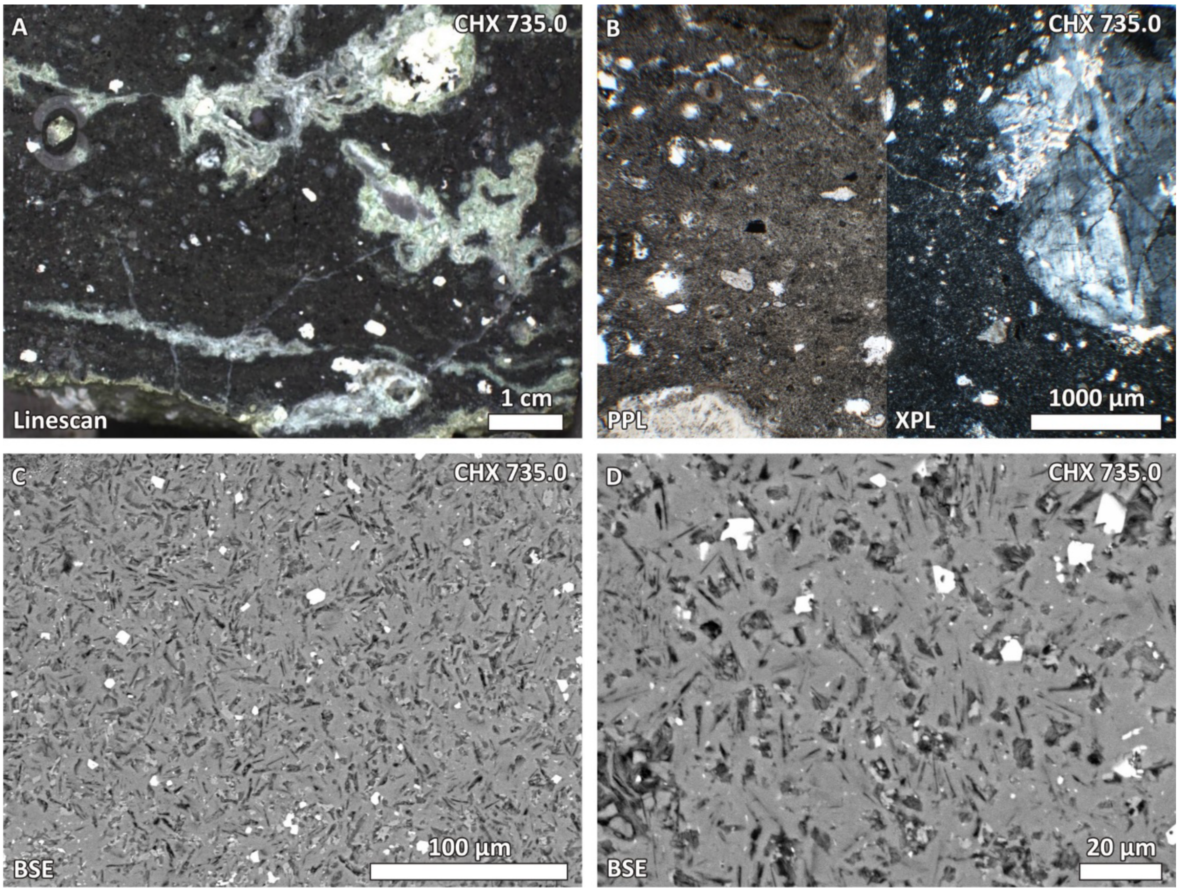

Figure A- 3: Linescan, transmitted light, and BSE images of impact melt rock sample CHX 735.0. A) Linescan image (photograph) taken of the core section from which sample CHX 735.0 originates. The light green

portions of the sample are carbonates, the black portion is silicate impact melt rock. B) Transmitted light photomicrograph of a representative portion of CHX 735.0. C) BSE image of the groundmass showing microcrystalline texture. D) BSE magnification of the groundmass showing dark areas to be pyroxene-shaped voids, either completely empty or with infilling clays, and the grey material to be consolidated plagioclase. PPL = plane polarised light; XPL = between crossed-polarisers, BSE = backscatter electron image.

Sample CHX 756.0

(IODP sample ID: 364_77_A_98_R_3_W_83.0-86.0)

CHX 756.0 samples an area of impact melt rock with two differently coloured components: one black (CHX 756.0A, Run ID 93292) and the other light grey/brown (CHX 756.0B, Run ID 93303). The two different coloured components were cut and prepared for argon analyses separately in order to account for any effects of alteration on the argon isotope compositions.

In hand sample the two components are interlayered but distinct (Fig. A-4A). In thin section they are separated by a dark brown layer, but they share a similar microcrystalline texture and clast content (Fig. A-4B), which suggests that the colour difference is a result of alteration rather than a result of different original compositions. The groundmass of the grey/brown component is composed of lath-like plagioclase crystallites, which occasionally have swallow-tail terminations and/or skeletal texture (Fig. A-4D). The areas between the crystallites are virtually empty in 756.0B (Fig. A-4D), but in 756.0A they are filled with Mg-rich clays (Fig. A-4C). Texturally, parts of 756.0A are more densely packed with plagioclase crystallites, and exhibit overgrowths of K-rich feldspar. The contact between the two is diffuse at high magnification, but is highlighted by chemical differences and an increase in the interstitial clay content (Fig. A-4E, F).

Sample CHX 756.0A (black) is enriched relative to sample CHX 756.0B (grey/brown) in Al, K, O, Si, and (slightly) in Na. 756.0B is enriched relative to 756.0A in C and Cl, the result of more pore space and therefore more resin. The contact between the two is enriched in Mg, but otherwise Mg is distributed uniformly in both lithologies.

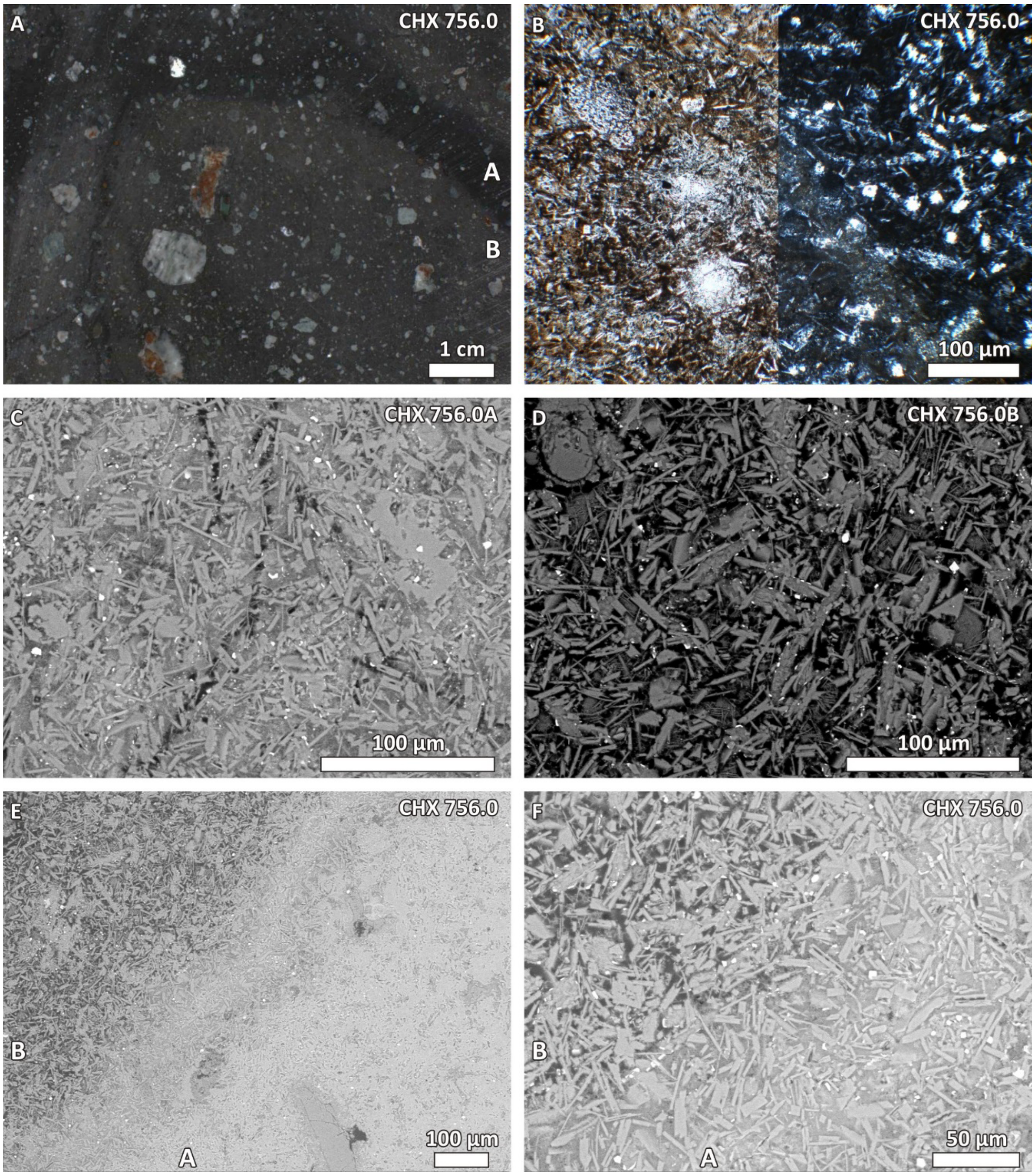

Figure A- 4: Linescan, transmitted light, and BSE images of impact melt rock sample CHX 756.0. A) Linescan image (photograph) taken of the core section from which sample CHX 756.0 originates. The black unit labelled "A" is subsample CHX 756.0A; the grey unit (labelled "B") is subsample CHX 756.0B. B) Transmitted light photomicrograph of a representative portion of CHX 756.0. C) BSE image of a representative part of CHX 756.0A, plagioclase laths make up the majority of the area, interstitial material (darker grey) is Mg-rich clay. D) BSE image of a representative part of 756.0B, plagioclase laths make up the majority of the area, interstitial material is small amounts of relict clay, but mostly pore space. Swallow tail terminations on plagioclase laths are visible in both samples CHX 756.0A and 756.0B. E) BSE image of the contact between units 756.0A and 756.0B. CHX 756.0A is relatively enriched in Al, K, O, Si, and slightly in Na. CHX 756.0B appears relatively enriched in C and Cl due to the higher volume of pore space which is infilled by resin. The boundary between the two zones is enriched in Mg. F) BSE magnification of the contact between 756.0A and 756.0B showing little textural difference between the two except for volume of pore space. PPL = plane polarised light; XPL = between crossed-polarisers, BSE = backscatter electron image.

Supplementary Materials

Supplementary Materials B – Argon Data Figures and Tables

Table of Figures

Figure B- 1: Age spectra of Chicxulub impact melt rock sample CHX 735.0. 9

Figure B- 2: Isotope correlation plots for Chicxulub impact melt rock sample CHX 735.0. 10

Figure B- 3: Age spectra of Chicxulub impact melt rock sample CHX 756.0A. 11

Figure B- 4: Isotope correlation plots for Chicxulub impact melt rock sample CHX 756.0A. 12

Figure B- 5: Age spectra of Chicxulub impact melt rock sample CHX 756.0B. 13

Figure B- 6: Isotope correlation plots for Chicxulub impact melt rock sample CHX 756.0B. 14

Figure B- 7: Age spectra for Chicxulub impact melt rock sample CHX 706.4A. 15

Figure B- 8: Isotope correlation plots for Chicxulub impact melt rock sample CHX 706.4A. 16

Table of Tables

Table B- 1: Age data summary table for Chicxulub sample CHX 706.4A (no plateau ages for this sample). 17

Table B- 2: Age data summary table for Chicxulub impact melt rock samples CHX 735.0, CHX 756.0A, and CHX 756.0B 18

Table B- 3: Summary of weighted mean ages of all Chicxulub samples. 20

Table B- 4: Nucleogenic production ratios and conversion factors, OSU TRIGA reactor 21

Table B- 5: Isotopic constants and decay rates 21

Table B- 6: Irradiation Schedule 21

Table B- 7: Data table for Chicxulub step heating experiments 22

Table B- 8: IrZ data 28

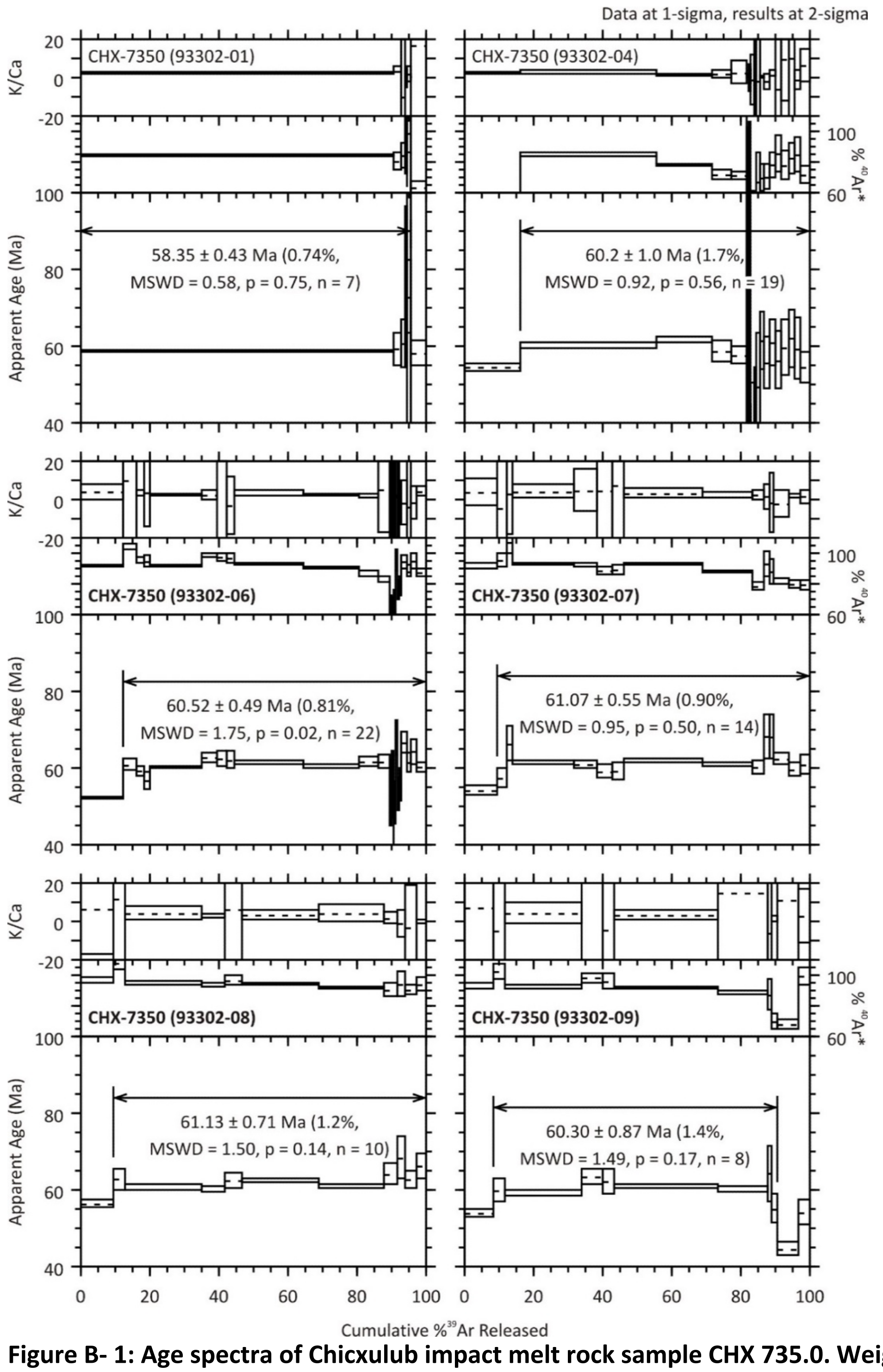

Figure B- 1: Age spectra of Chicxulub impact melt rock sample CHX 735.0. Weighted mean of plateau ages is 60.72 ± 0.35 Ma (2σ, 0.57%, MSWD = 1.4, p = 0.228, n = 5; 93302-01 excluded due to large % ^{39}Ar released in first step). Top two panels (Run IDs 93302-01 and 93302-04) are from single grain aliquots; all others are from 4-grain aliquots.

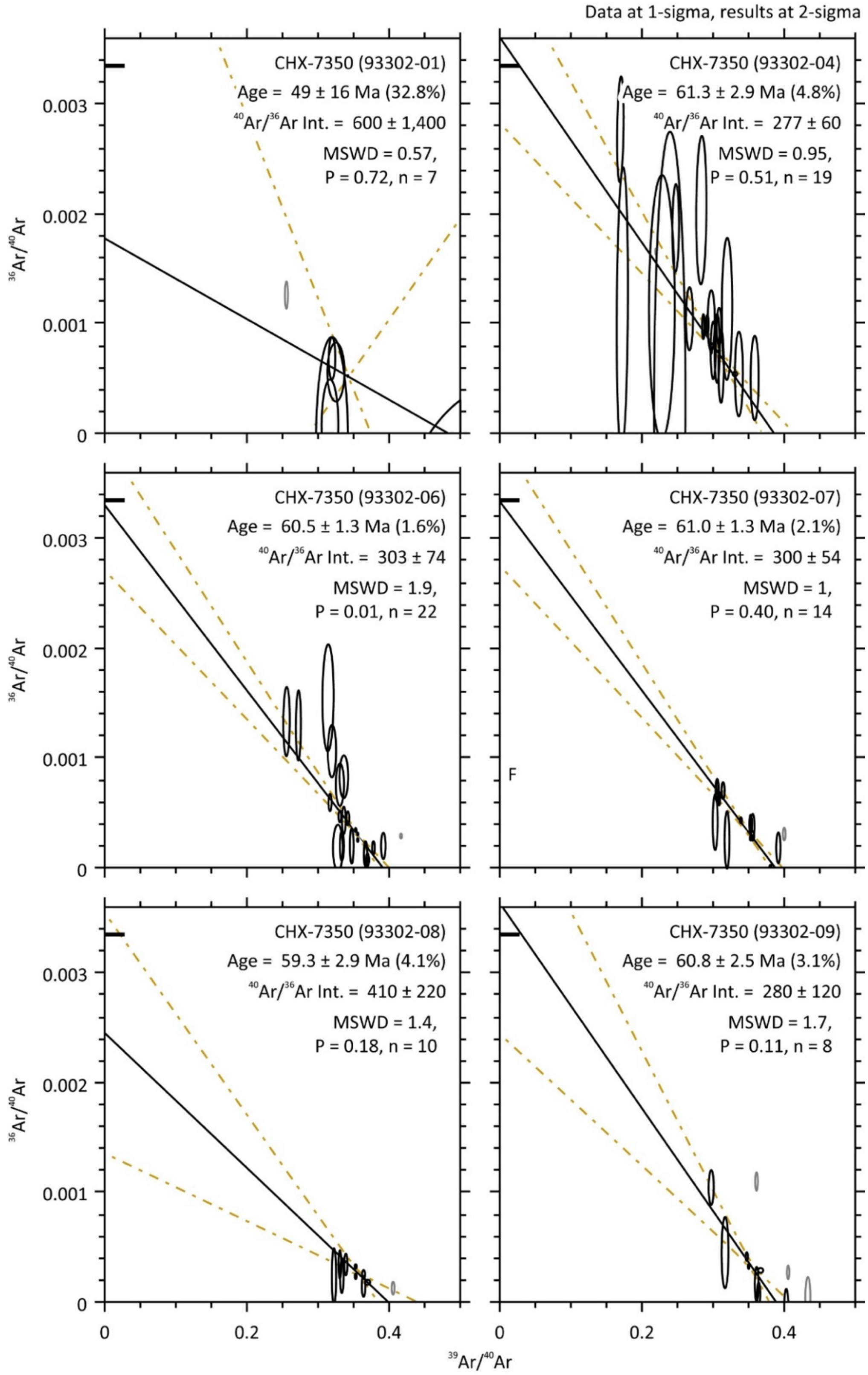

Figure B- 2: Isotope correlation plots for Chicxulub impact melt rock sample CHX 735.0. Top two panels (Run IDs 93302-01 and 93302-04) are from single grain aliquots; all others are from 4-grain aliquots. Inverse isochrons are defined by plateau steps only. Grey ellipses are from analyses not included in the plateau.

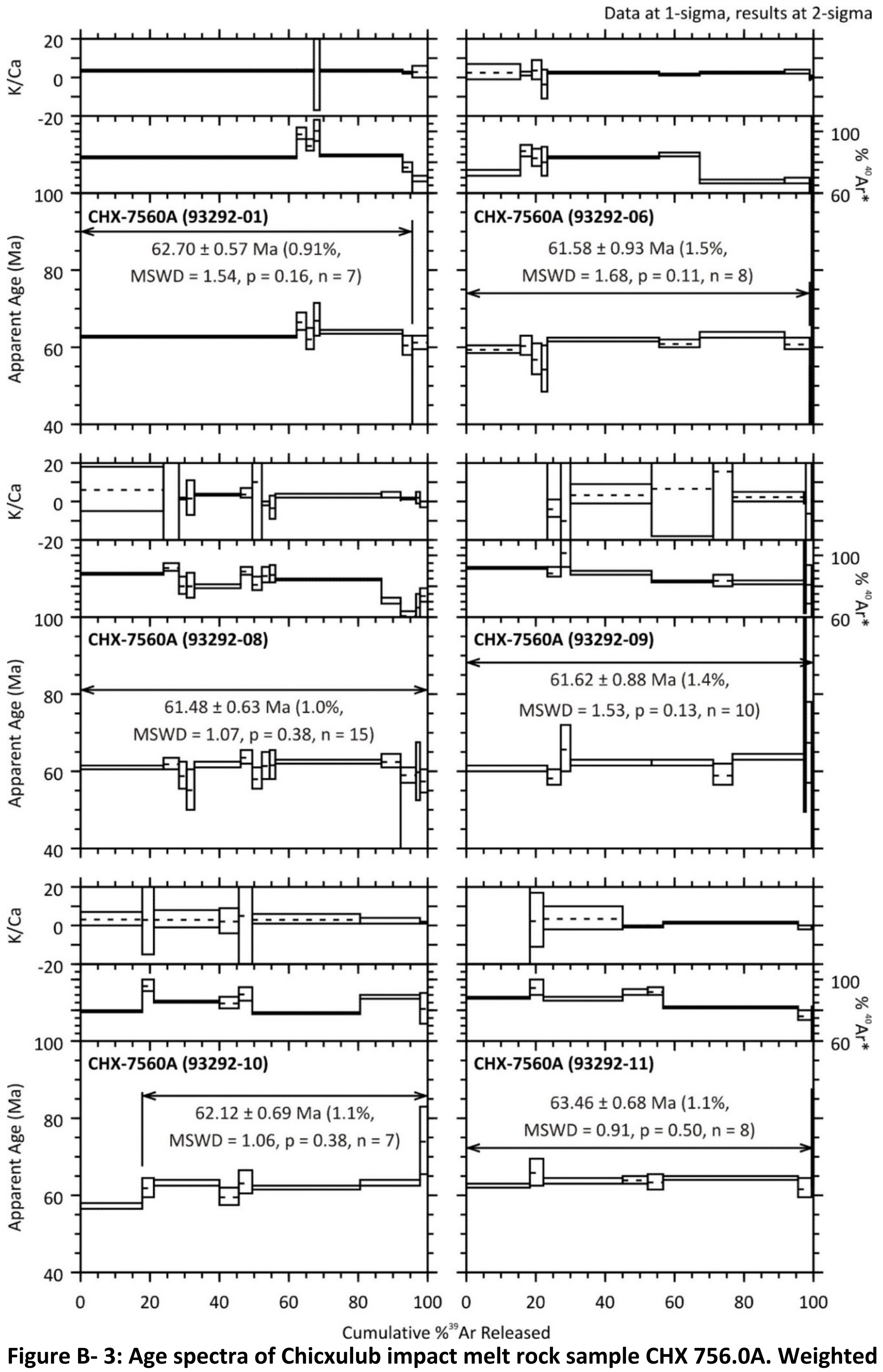

Figure B- 3: Age spectra of Chicxulub impact melt rock sample CHX 756.0A. Weighted mean of plateau ages is 62.27 ± 0.64 Ma (2σ, 1.03%, MSWD = 5.1, p = 0.000, n = 6). Top two panels (Run IDs 93292-01 and 93296-06) are from single grain aliquots; all others are from 4-grain aliquots.

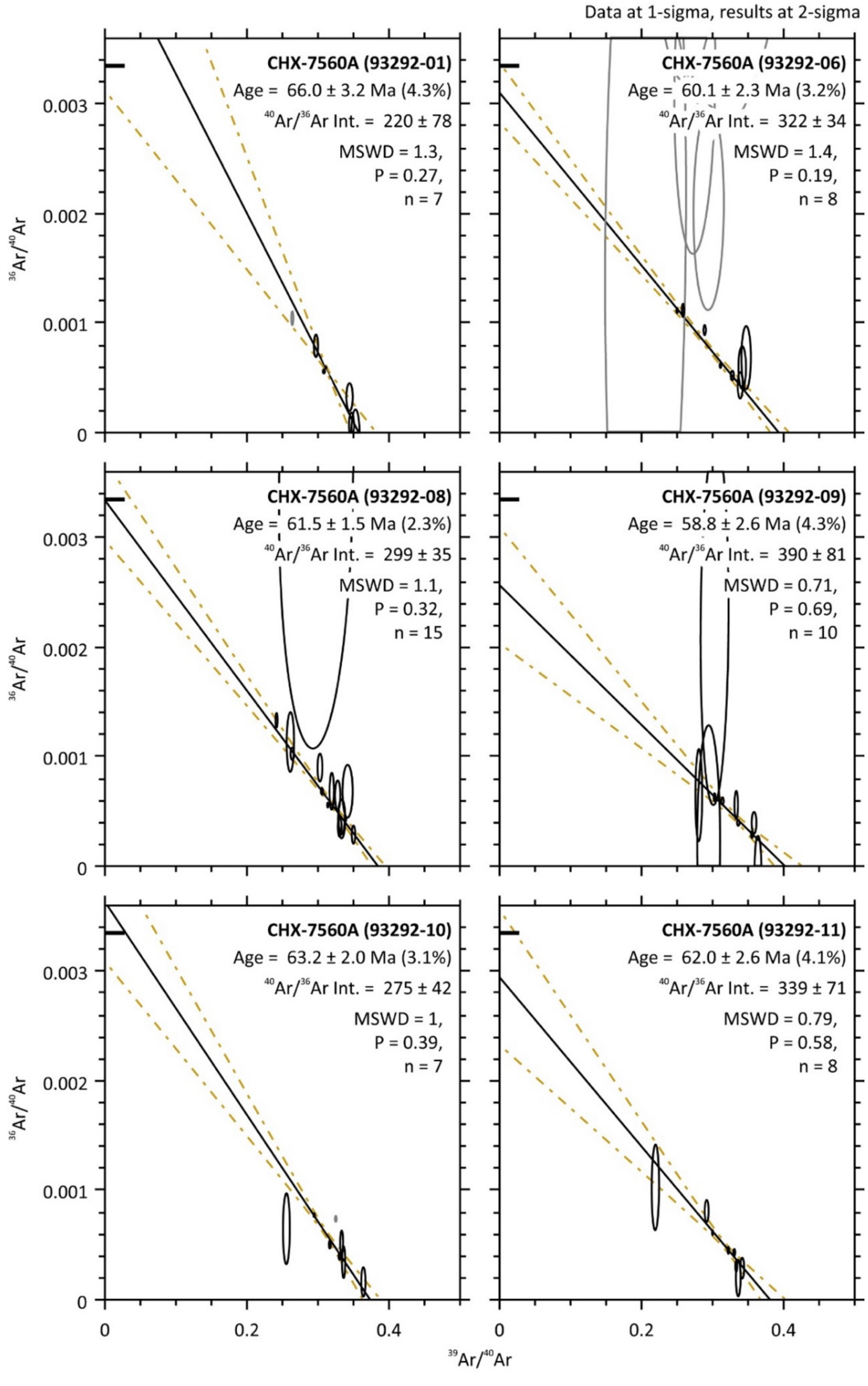

Figure B- 4: Isotope correlation plots for Chicxulub impact melt rock sample CHX 756.0A. Top two panels (Run IDs 93302-01 and 93302-04) are from single grain aliquots; all others are from 4-grain aliquots. Inverse isochrons are defined by plateau steps only. Grey ellipses are from analyses not included in the plateau.

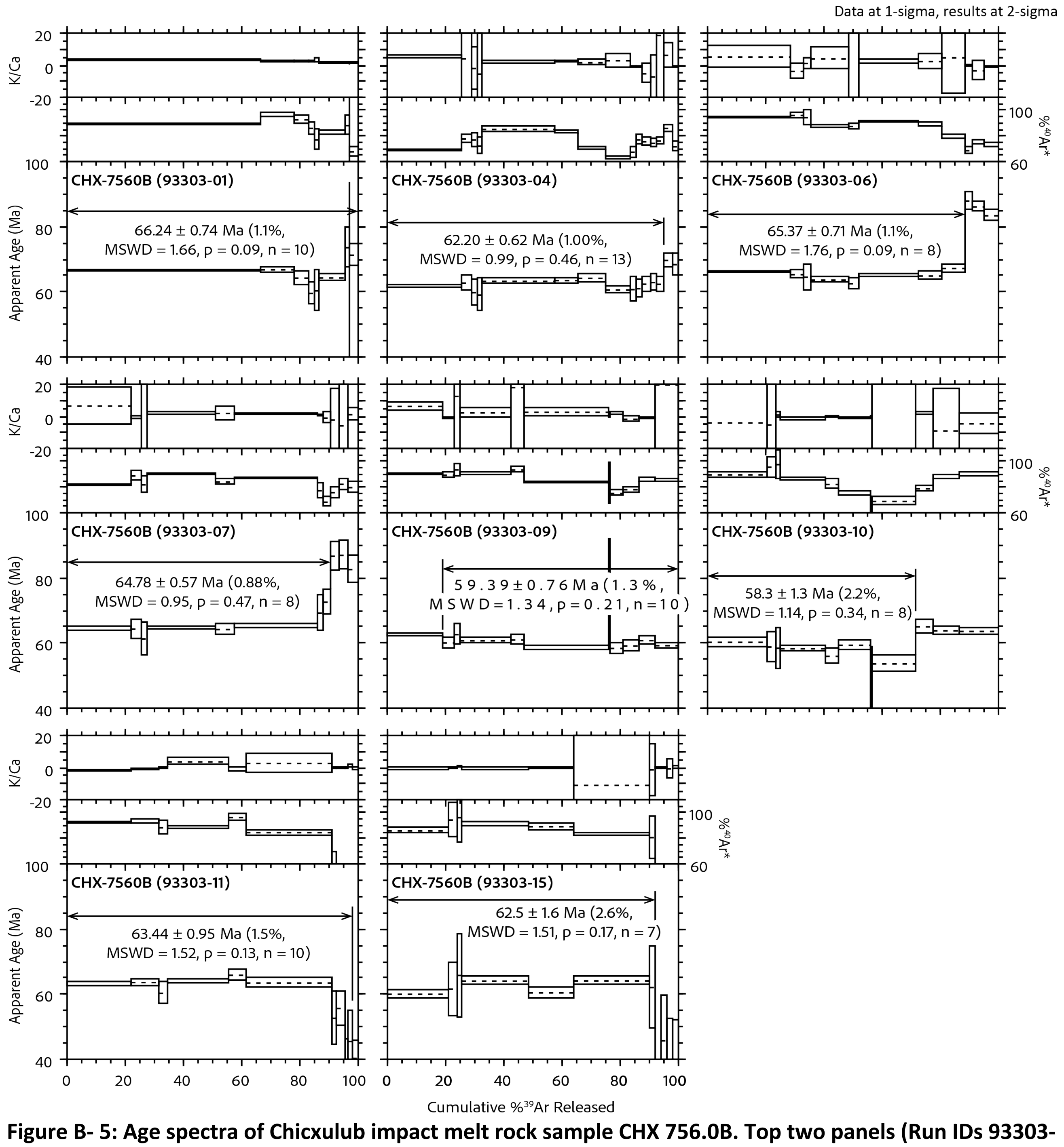

Figure B- 5: Age spectra of Chicxulub impact melt rock sample CHX 756.0B. Top two panels (Run IDs 93303-01 and 93303-04) are from single grain aliquots; all others are from 3-grain aliquots.

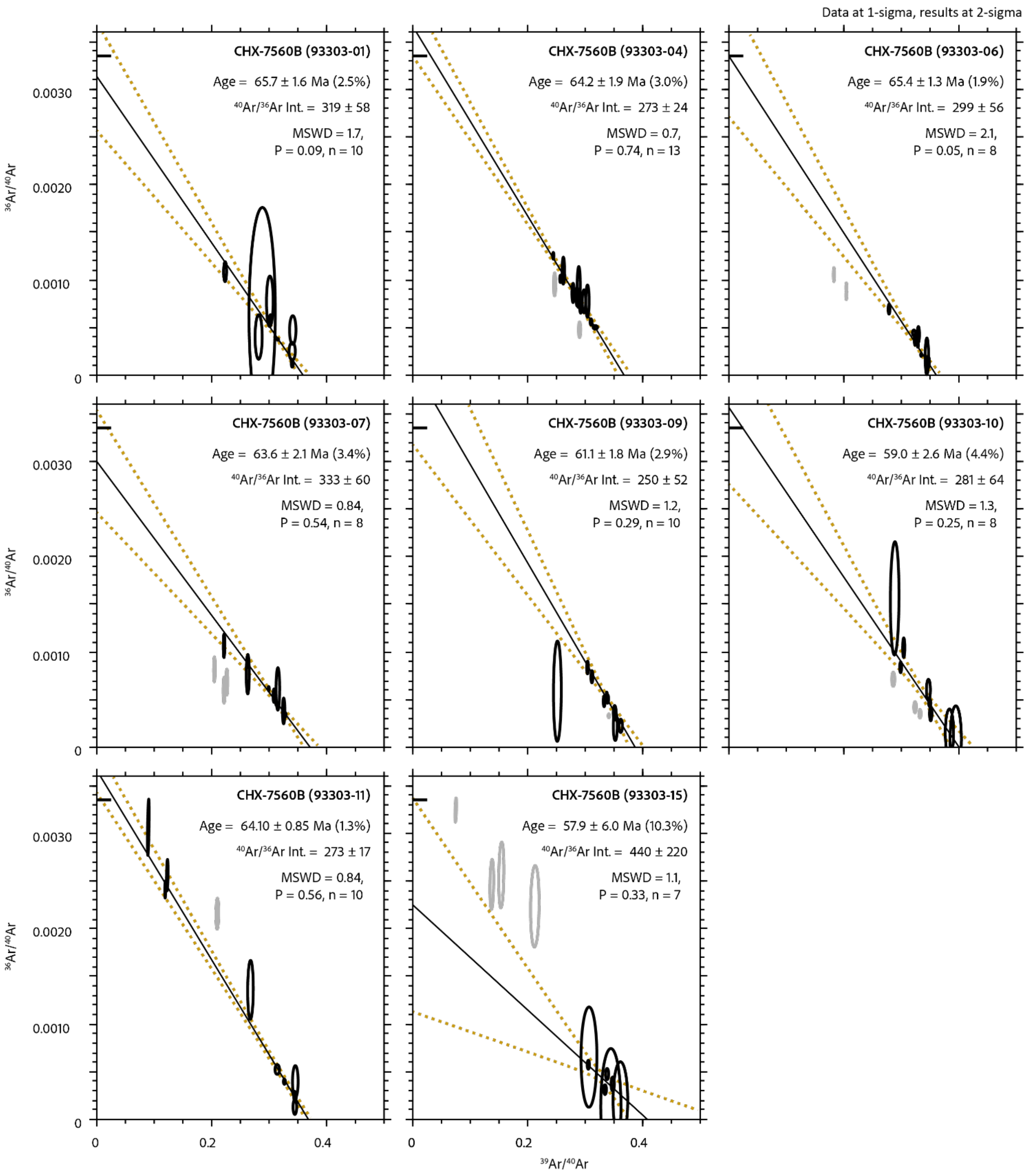

Figure B- 6: Isotope correlation plots for Chicxulub impact melt rock sample CHX 756.0B. Top two panels (Run IDs 93303-01 and 93303-04) are from single grain aliquots; all others are from 3-grain aliquots. Inverse isochrons are defined by plateau steps only. Grey ellipses are from analyses not included in the plateau.

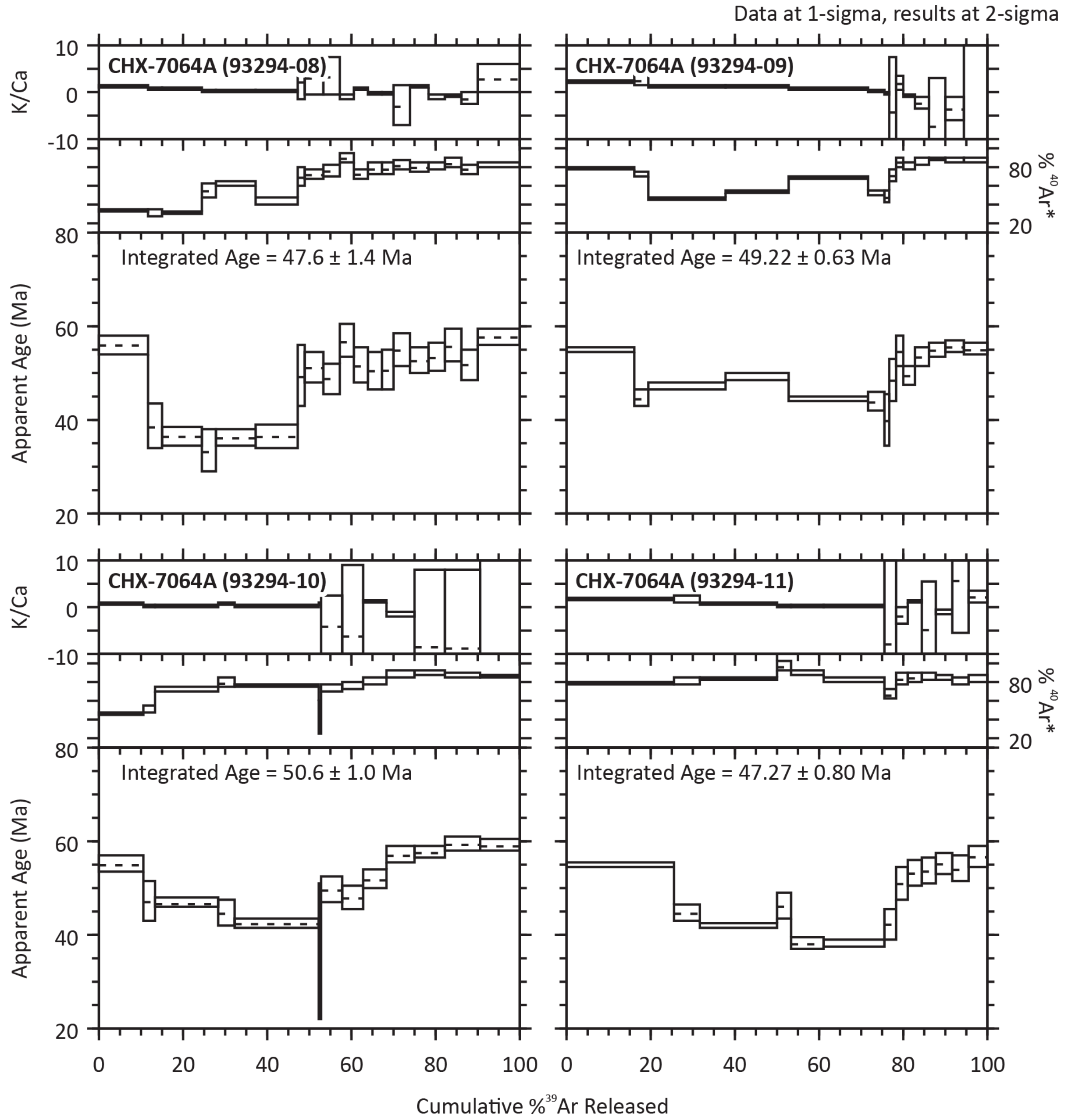

Figure B- 7: Age spectra for Chicxulub impact melt rock sample CHX 706.4A. Weighted mean of integrated ages is 48.8 ± 1.2 Ma (2σ, 2.5%, MSWD = 8.2, p = 0.000, n = 6). Top two panels (Run IDs 93294-05 and 93294-06) are from single grain aliquots; all others are from 4-grain aliquots.

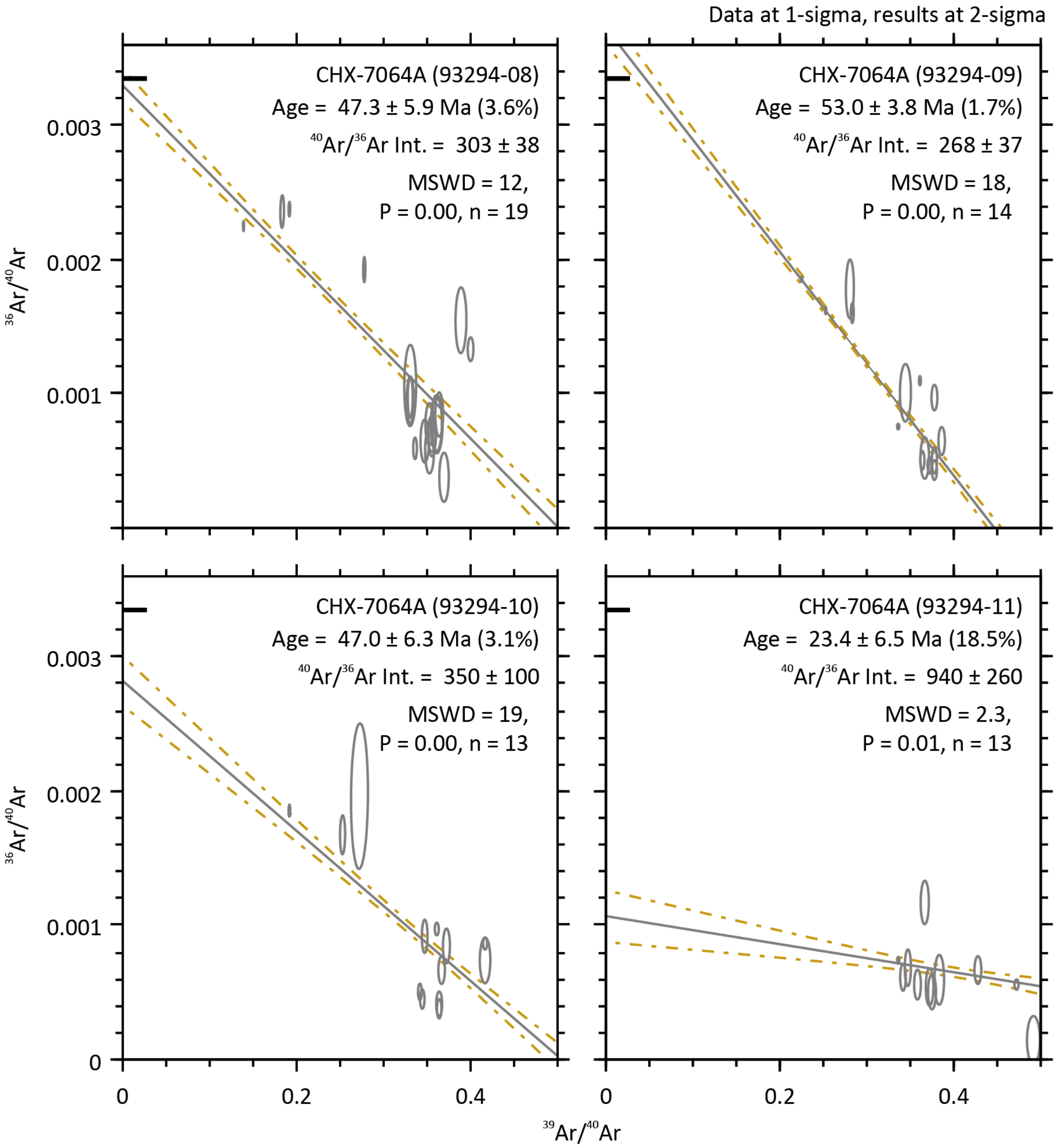

Figure B- 8: Isotope correlation plots for Chicxulub impact melt rock sample CHX 706.4A. Top two panels (Run IDs 93294-05 and 93294-06) are from single grain aliquots; all others are from 4-grain aliquots.

$^{40}Ar/^{39}Ar$ summary tables

Table B- 1: Age data summary table for Chicxulub sample CHX 706.4A (no plateau ages for this sample).

Run ID	Integrated ages<br>Age (Ma)	± 2σ	± %	MSWD	P	n
93294-08	47.6	1.4	2.9			19
93294-09	49.2	0.63	1.3			14
93294-10	50.6	1.0	2.0			13
93294-11	47.3	0.80	1.7			13
**Weighted mean**	**48.8**	**1.4**	**2.8**	**11**	**0.000**	**4**

Run ID	Isochron ages<br>Age (Ma)	± 2σ	± %	MSWD	P	n	$^{40}Ar/^{36}Ar$	± 2σ
93294-08	47.3	5.9	12	12	0.000	19	303	38
93294-09	53.0	3.8	7.2	18	0.000	14	268	37
93294-10	47.0	6.3	13	19	0.000	13	350	100
93294-11	23.4	6.5	28	2.3	0.010	13	940	260
**Weighted mean**	**46.1**	**11.9**	**25.8**	**21**	**0.000**	**4**		

Table B- 2: Age data summary table for Chicxulub impact melt rock samples CHX 735.0, CHX 756.0A, and CHX 756.0B

CHX 735.0

	Plateaus							Integrated ages					
**Run ID**	**Age (Ma)**	**± 2σ**	**± %**	**MSWD**	**P**	**n**	**%^{39}Ar**	**Age (Ma)**	**± 2σ**	**± %**	**MSWD**	**P**	**n**
93302-01*	58.35	0.43	0.74	0.58	0.750	7	95.8	58.6	0.66	1.1			10
93302-04	60.20	1.0	1.7	0.92	0.560	19	83.7	58.4	1.3	2.2			20
93302-06	60.52	0.49	0.81	1.8	0.020	22	87.3	59.6	0.47	0.79			23
93302-07	61.07	0.55	0.90	0.95	0.500	14	90.2	60.4	0.66	1.1			15
93302-08	61.13	0.71	1.2	1.5	0.140	10	90.1	61.0	0.72	1.2			11
93302-09	60.30	0.87	1.4	1.5	0.170	8	82.1	58.6	0.78	1.3			11
**Weighted mean**	60.72	0.35	0.57	1.4	0.228	5		59.6	0.80	1.34	8.5	0.000	6

*this run omitted from weighted mean

	Isochron ages (plateau steps only)								Isochron ages (all steps)							
**Run ID**	**Age (Ma)**	**± 2σ**	**± %**	**MSWD**	**P**	**n**	**^{40}Ar/^{36}Ar**	**±**	**Age (Ma)**	**± 2σ**	**± %**	**MSWD**	**P**	**n**	**^{40}Ar/^{36}Ar**	**±**
*93302-01**	*49*	16	33	*0.57*	*0.720*	*7*	*600*	*1400*	*58.4*	2.9	*5.0*	*0.81*	*0.590*	*10*	*298*	*87*
93302-04	61.3	2.9	4.7	1.0	0.510	19	277	60	62.6	1.4	2.2	0.94	0.530	20	251	14
93302-06	60.5	1.3	2.1	1.9	0.010	22	303	74	44.3	5.9	13	5.8	0.000	23	1100	750
93302-07	61.0	1.3	2.1	1.0	0.400	14	300	54	58.2	2.8	4.8	3.3	0.000	15	410	140
93302-08	59.3	2.9	4.9	1.4	0.180	10	410	220	56.7	3.5	6.2	1.6	0.110	11	560	270
93302-09	60.8	2.5	4.1	1.7	0.110	8	280	120	53.3	6.9	13	12	0.000	11	570	740
**Combined isochron**	**61.0**	**0.7**	**1.1**	**2.3**	**0.000**	**80**	**265**	**26**	**59.1**	**0.94**	**1.6**	**8.4**	**0.000**	**90**	**312**	**31**

CHX756.0A

	Plateaus	Integrated ages

Run ID	Age (Ma)	± 2σ	± %	MSWD	P	n	%^{39}Ar	Age (Ma)	± 2σ	± %	MSWD	P	n
93292-01	62.70	0.57	0.91	1.5	0.160	7	95.7	62.6	0.56	0.89			9
93292-06	61.58	0.93	1.5	1.7	0.110	8	99.0	60.4	1.0	1.7			14
93292-08	61.48	0.63	1.0	1.1	0.380	15	100.0	61.2	0.76	1.2			15
93292-09	61.62	0.88	1.4	1.5	0.130	10	100.0	61.8	0.99	1.6			10
93292-10	62.12	0.69	1.1	1.1	0.380	7	82.0	61.5	0.80	1.3			8
93292-11	63.46	0.68	1.1	0.91	0.500	8	100.0	63.6	0.78	1.2			8
**Weighted mean**	**62.27**	**0.64**	**1.03**	**5.1**	**0.000**	**6**		**62.1**	**0.87**	**1.40**	**7.6**	**0.000**	**6**

Run ID	Isochron ages (plateau steps only) Age (Ma)	± 2σ	± %	MSWD	P	n	^{40}Ar/^{36}Ar	±	Isochron ages (all steps) Age (Ma)	± 2σ	± %	MSWD	P	n	^{40}Ar/^{36}Ar	±
93292-01	66.0	3.2	4.8	1.3	0.270	7	220	78	64.7	2.6	4.0	1.8	0.090	9	251	62
93292-06	60.1	2.3	3.8	1.4	0.190	8	322	34	60.1	2.4	4.0	1.6	0.090	14	322	36
93292-08	61.5	1.5	2.4	1.1	0.320	15	299	35	61.5	1.5	2.4	1.1	0.320	15	299	35
93292-09	58.8	2.6	4.4	0.71	0.690	10	390	81	58.8	2.6	4.4	0.71	0.690	10	390	81
93292-10	63.2	2.0	3.2	1.0	0.390	7	275	42	59.4	6.8	11.4	9.8	0.000	8	330	170
93292-11	62.0	2.6	4.2	0.79	0.580	8	339	71	62.0	2.6	4.2	0.79	0.580	8	339	71
**Combined isochron**	**61.8**	**0.89**	**1.4**	**1.7**	**0.000**	**55**	**309**	**20**	**61.5**	**1.1**	**1.8**	**2.8**	**0.000**	**64**	**312**	**25**

CHX 756.0B

Run ID	Plateaus Age (Ma)	± 2σ	± %	MSWD	P	n	%^{39}Ar	Integrated ages Age (Ma)	± 2σ	± %	MSWD	P	n
93303-01	66.24	0.74	1.1	1.7	0.09	10	100	66.2	0.71	1.1			10
93303-04	62.20	0.63	1.0	0.99	0.46	13	95.5	62.6	0.79	1.3			15
93303-06	65.37	0.71	1.1	1.8	0.09	8	88.6	67.5	0.66	1.0			11
93303-07	64.78	0.57	0.88	0.95	0.47	8	90.8	66.8	0.87	1.3			11
93303-09	59.39	0.76	1.3	1.3	0.21	10	80.9	60.2	0.58	0.96			11
93303-10	58.30	1.3	2.2	1.1	0.34	8	71.5	59.5	1.2	2.0			11

93303-11	63.44	1.0	1.5	1.5	0.13	10	98.2		62.0	1.3	2.1			11
93303-15	62.50	1.6	2.6	1.5	0.17	7	92		59.9	1.9	3.2			11
**Weighted mean**	**63.07**	**2.2**	**3.4**	**63**	**0.00**	**9**			**63.2**	**2.8**	**4.5**	**98**	**0.00**	**9**

	**Isochron ages (plateau steps only)**								**Isochron ages (all steps)**							
**Run ID**	**Age (Ma)**	**± 2σ**	**± %**	**MSWD**	**P**	**n**	**$^{40}Ar/^{36}Ar$**	**±**	**Age (Ma)**	**± 2σ**	**± %**	**MSWD**	**P**	**n**	**$^{40}Ar/^{36}Ar$**	**±**
93303-01	65.7	2.2	3.3	1.9	0.06	9	319	80	65.7	2.1	3.2	1.7	0.09	10	319	75
93303-04	64.2	1.9	3.0	0.70	0.74	13	273	23	64.6	2.4	3.7	1.8	0.04	15	271	31
93303-06	65.4	1.8	2.8	2.1	0.05	8	299	82	61.2	2.6	4.2	4.2	0.00	11	507	82
93303-07	63.6	2.1	3.3	0.84	0.54	8	333	60	58.9	4.7	8.0	3.3	0.00	11	470	150
93303-09	61.1	1.8	2.9	1.2	0.29	10	250	52	62.7	1.2	1.9	3.6	0.00	11	223	41
93303-10	59	2.6	4.4	1.3	0.25	8	281	64	58.7	2.4	4.1	4.5	0.00	11	349	77
93303-11	64.1	0.85	1.3	0.84	0.56	10	273	17	64.2	1.2	1.9	2.0	0.04	11	266	23
93303-15	57.9	6.4	11	1.1	0.33	7	440	240	64.0	1.7	2.7	1.8	0.07	11	250	25
**Combined isochron**	**62.5**	**2.8**	**4.4**	**24**	**0.00**	**9**			**61.3**	**3.0**	**4.9**	**28**	**0.00**	**9**		

Table B- 3: Summary of weighted mean ages of all Chicxulub samples.

Sample	Run ID	Age (Ma)	±2σ (Ma)	±2σ %	MSWD	p	n	Weighted mean of:
CHX-756.0B	93303-01, 04, 06, 07, 09, 10, 11, 14, 15	63.07	2.2	3.4	63	0.000	9	plateaus
CHX-756.0A	93292-01, 06, 08, 09, 10, 11	62.27	0.64	1.03	5.1	0.000	6	plateaus
CHX-735.0	93302-04, 06, 07, 08, 09	60.72	0.35	0.57	1.4	0.228	5	plateaus
CHX-706.4A	93294-08, 09, 10, 11	48.8	1.4	2.8	11	0.000	4	integrated ages

Table B- 4: Nucleogenic production ratios and conversion factors, OSU TRIGA reactor

Production ratios				
$(^{36}Ar/^{38}Ar)_{Cl}$	263	± 2		1
$(^{38}Ar/^{37}Ar)_{Ca}$	1.96	± 0.08	× 10^{-5}	2
$(^{37}Ar/^{39}Ar)_{K}$	2.24	± 0.16	× 10^{-4}	2
$(^{38}Ar/^{39}Ar)_{K}$	1.22	± 0.0027	× 10^{-2}	2
$(^{36}Ar/^{37}Ar)_{Ca}$	2.65	± 0.02	× 10^{-4}	2
$(^{39}Ar/^{37}Ar)_{Ca}$	6.95	± 0.09	× 10^{-4}	2
$(^{40}Ar/^{39}Ar)_{K}$	7.30	± 0.92	× 10^{-4}	2
Conversion factors to molecular ratios				
37Ar/39Ar to Ca/K	1.960			
38ArCl/39Ar to Cl/K	2.900			

Table B- 5: Isotopic constants and decay rates

		±σ		
$\lambda(^{40}K_{\varepsilon})$/yr	5.757	± 0.016	× 10^{-11}	3
$\lambda(^{40}K_{\beta-})$/yr	4.9548	± 0.0134	× 10^{-10}	3
$\lambda(^{40}K_{total})$/yr	5.5305	± 0.0135	× 10^{-10}	3
$\lambda(^{37}Ar)$/d	1.983	± 0.00226	× 10^{-2}	4
$\lambda(^{39}Ar)$/d	7.055	± 0.039	× 10^{-6}	5
$\lambda(^{36}Cl)$/d	6.1817	± 0.04	× 10^{-9}	1
$(^{40}Ar/^{36}Ar)_{atm}$	298.56	± 0.31	--	6
$(^{40}Ar/^{38}Ar)_{atm}$	1584	2		6
$^{40}K/K_{total}$	0.011672	0.0000205	--	7

Table B- 6: Irradiation Schedule

#	Date (M/D/Y)	Start	End	Duration [hh:mm]
1	08/07/2017	09:22	12:07	02:45
2	08/08/2017	09:43	15:12	05:29
3	08/09/2017	08:10	15:34	07:24
4	08/10/2017	09:01	10:54	01:53
5	08/11/2017	09:09	16:09	07:00
6	8/14/2017	08:43	13:43	05:00
7	8/15/2017	08:58	15:27	06:29
8	8/16/2017	08:48	15:48	07:00
9	8/17/2017	09:12	16:12	07:00
			Total:	***50:00***

Table B- 7: Data table for Chicxulub step heating experiments

All data in the following tables has been corrected for blanks, mass discrimination, radioactive decay, and neutron interferences.

Material analysed: crystalline impact melt rock (single and multi-grain aliquots)

Mass spectrometer: MAP 215 noble gas

Decay constants and standard ages: Renne *et al.* (2010, 2011)

J: 0.0132897 ± 0.0000105 (1σ, determined from analyses of IrZ sanidine)

*Plateau steps

Run ID	Power (%)	Run Date & Time	^{39}Ar (moles)	^{40}Ar (V)	± σ	^{39}Ar (V)	± σ	^{38}Ar (V)	± σ	^{37}Ar (V)	± σ	^{36}Ar (V)	± σ	%^{40}Ar*	± σ	^{40}Ar*/ ^{39}Ar	± σ	Age (Ma)	± σ
**Sample: CHX-7560A; Material: Impact melt; D: 1.009502 ± 0.0006813; ngrains: 1**																			
93292-01A*	0.2	16/04/2018 09:26	9.13E-18	-0.0002809	0.0010201	0.0000808	0.0000333	-0.000013	0.0000365	-0.0000125	0.0000085	-0.0000026	0.0000153	-130.895	1735.576	4.476526	57.11814	104.4919	1295.47
93292-01B*	0.3	16/04/2018 10:46	1.24E-13	3.508678	0.0054593	1.096532	0.0021	0.0138682	0.0001102	0.0012956	0.0000331	0.002075	0.0000292	82.72793	0.323124	2.642204	0.011292	62.40243	0.262136
93292-01D*	0.5	16/04/2018 13:54	5.79E-15	0.1474586	0.0013618	0.0512091	0.0001303	0.0006597	0.0000464	0.0000596	0.0000102	0.0000117	0.0000163	98.06324	3.552059	2.818443	0.099013	66.48913	2.293364
93292-01E*	0.6	16/04/2018 15:20	4.40E-15	0.1132643	0.0013617	0.0389413	0.0001204	0.0003971	0.0000444	0.0000498	0.00001	0.0000379	0.0000143	90.46681	4.123776	2.626376	0.115809	62.03495	2.689019
93292-01F*	0.7	16/04/2018 16:11	3.45E-15	0.0865339	0.0012821	0.0305519	0.0001204	0.0004127	0.0000395	0.0000057	0.0000102	-0.0000006	0.0000182	100.2887	6.652888	2.834906	0.183679	66.8704	4.253516
93292-01G*	0.8	16/04/2018 18:19	4.72E-14	1.346853	0.0019705	0.4175201	0.0016	0.00516	0.0000782	0.0005861	0.00015	0.0007582	0.0000232	83.649	0.562867	2.693425	0.020909	63.59111	0.485064
93292-01H*	0.9	16/04/2018 19:51	5.68E-15	0.1682573	0.0015271	0.0502719	0.0001303	0.0006947	0.0000414	0.000083	0.0000112	0.000135	0.0000173	76.56991	3.281079	2.558122	0.107417	60.44942	2.496348
93292-01I	1	16/04/2018 20:41	4.40E-16	0.0130499	0.0010315	0.0038939	0.000044	0.0000306	0.0000395	0.000048	0.000009	0.0000471	0.0000143	-3.85879	33.78913	-0.12922	1.131472	-3.10773	27.23584
93292-01J	5	16/04/2018 22:49	8.04E-15	0.2683031	0.0012809	0.0711729	0.0001603	0.0010039	0.0000444	0.0000985	0.00011	0.0002836	0.0000163	68.83141	1.957247	2.590071	0.073104	61.19178	1.69823
Blanks (mean ± s.d.)			1.31E-17	0.0075515	0.0008005	0.0001157	0.0000093	0.000073	0.000006	0.0003494	0.0000022	0.0000692	0.000003						
Sample: CHX-7560A; Material: Impact melt; D: 1.005686 ± 0.0004619; ngrains: 1																			
93292-06A*	0.3	21/06/2018 16:05	4.50E-14	1.38116	0.0031052	0.3986704	0.002	0.0047641	0.0000921	0.0001721	0.00031	0.0012901	0.0000485	72.58875	1.399373	2.509071	0.050221	59.30914	1.167858
93292-06B*	0.4	21/06/2018 17:01	1.05E-14	0.2728273	0.0026062	0.0929623	0.0002902	0.0010912	0.0000591	0.0000574	0.0000293	0.0001246	0.0000327	87.17698	3.825867	2.552746	0.109691	60.3245	2.549375
93292-06C*	0.5	21/06/2018 19:19	7.15E-15	0.1843047	0.002706	0.0632782	0.0002203	0.0007834	0.0000522	0.0000185	0.0000243	0.0001098	0.0000356	82.59491	6.111232	2.399998	0.174292	56.77092	4.058744
93292-06D*	0.7	21/06/2018 20:54	5.28E-15	0.1341413	0.0024067	0.0467152	0.0002103	0.0005979	0.0000462	-0.0000131	0.0000263	0.0000882	0.0000386	79.9732	8.935319	2.290534	0.252862	54.22003	5.896743
93292-06E*	0.8	21/06/2018 21:46	9.17E-14	2.594105	0.0065025	0.8118103	0.002	0.0101169	0.0001301	0.0003253	0.0000303	0.0016075	0.0000554	81.98621	0.719097	2.613814	0.02304	61.74324	0.535065
93292-06F*	0.9	22/06/2018 00:06	3.28E-14	0.8829518	0.004004	0.2898675	0.0013	0.0034267	0.0000821	0.0001986	0.0000313	0.0004774	0.0000376	84.73405	1.414382	2.575336	0.043068	60.84945	1.000671
93292-06G*	1.6	22/06/2018 01:44	7.03E-14	2.485738	0.0053031	0.6223486	0.0017	0.007991	0.0001301	0.0003231	0.0000313	0.0027959	0.0000584	66.92346	0.750068	2.667094	0.030492	62.98014	0.707632
93292-06H*	12	22/06/2018 02:40	2.05E-14	0.6954525	0.0035046	0.1810879	0.0004201	0.0022031	0.0000711	0.0000775	0.0000253	0.0007772	0.0000415	67.0627	1.893066	2.569688	0.071726	60.71821	1.666636
93292-06I	16	22/06/2018 04:58	4.56E-16	0.0131618	0.0019085	0.0040365	0.000064	0.0000701	0.0000382	-0.000032	0.0000253	0.0000783	0.0000347	-87.3465	81.37874	-2.83339	2.607455	-69.4008	65.10836
93292-06J	20	22/06/2018 06:30	6.51E-16	0.0209768	0.0021077	0.0057605	0.0000917	0.0000805	0.0000352	-0.0000098	0.0000303	0.0000658	0.0000327	4.411021	48.01586	0.160144	1.743206	3.844132	41.79999
93292-06K	24	22/06/2018 07:22	1.14E-15	0.0343974	0.0024067	0.0101272	0.0000897	0.0001319	0.0000362	-0.0000668	0.0000362	0.0000611	0.0000317	39.22604	28.89128	1.326057	0.972592	31.58741	22.96649
93292-06L	28	22/06/2018 09:59	1.97E-16	0.0082927	0.0022074	0.0017454	0.0000581	0.0000998	0.0000333	0.0001072	0.0000283	0.000029	0.0000347	46.91392	129.0624	2.272276	6.223712	53.79421	145.1709
93292-06M	32	22/06/2018 11:32	3.19E-16	0.0101851	0.002307	0.0028208	0.0000709	0.0001361	0.0000372	0.0000335	0.0000263	0.0000749	0.0000376	-106.507	115.6834	-3.8523	4.092247	-95.0237	103.6421
93292-06N	36	22/06/2018 12:23	2.21E-16	0.0057457	0.0021077	0.0019544	0.000062	0.0000198	0.0000333	0.0000156	0.0000243	0.0000557	0.0000376	-178.947	210.2632	-5.26246	5.874736	-131.096	151.7842
Blanks (mean ± s.d.)			5.26E-17	0.018123	0.00018	0.0004658	0.000011	0.0001119	0.0000041	0.0015559	0.0000041	0.0002196	0.0000067						
Sample: CHX-7560A; Material: Impact melt; D: 1.006357 ± 0.0004496; ngrains: 4																			
93292-08A*	0.3	26/06/2018 20:48	1.01E-13	2.621376	0.0039051	0.8896107	0.0021	0.0105329	0.0001301	0.0001382	0.0002701	0.0011139	0.0000473	87.53444	0.730115	2.573068	0.022261	60.79675	0.517247
93292-08B*	0.4	26/06/2018 21:44	1.90E-14	0.4811464	0.0031064	0.1684217	0.0003701	0.0021834	0.0000651	0.0000066	0.0000265	0.000133	0.0000394	91.80416	2.610964	2.616145	0.07273	61.79736	1.688978
93292-08C*	0.5	26/06/2018 23:18	1.05E-14	0.2905828	0.002508	0.0933436	0.0002701	0.0010516	0.0000462	0.0001186	0.0000523	0.0002119	0.0000483	79.98499	5.149314	2.485004	0.158805	58.74939	3.694056
93292-08D*	0.7	27/06/2018 00:11	7.95E-15	0.2052189	0.0041049	0.0703429	0.0002202	0.0007679	0.0000582	0.0000447	0.0002601	0.0001441	0.0000314	79.96009	7.648369	2.327507	0.220304	55.08204	5.135041
93292-08E*	0.8	27/06/2018 02:28	5.54E-14	1.604323	0.0049041	0.4901777	0.0015	0.0059655	0.0001101	0.0001723	0.0000295	0.0011144	0.0000483	79.72086	0.986127	2.60313	0.032333	61.4951	0.750974
93292-08F*	0.9	27/06/2018 04:05	1.57E-14	0.4170645	0.0030067	0.138504	0.0003101	0.0017581	0.0000622	0.000036	0.0000255	0.0001509	0.0000374	89.56893	2.863716	2.690668	0.084072	63.52715	1.950498
93292-08G*	1	27/06/2018 04:58	1.04E-14	0.2791264	0.0030067	0.0919279	0.0002701	0.0011946	0.0000502	0.0000085	0.0000285	0.0001795	0.0000374	80.92515	4.263504	2.45116	0.126672	57.96196	2.94788
93292-08H*	1.1	27/06/2018 07:15	8.96E-15	0.2382957	0.0029069	0.0792484	0.0002501	0.0009267	0.0000512	-0.0000457	0.0000265	0.0001001	0.0000374	86.61756	4.986906	2.597488	0.146435	61.36405	3.401402
93292-08I*	1.2	27/06/2018 08:50	7.78E-15	0.206101	0.0029069	0.0688509	0.0003001	0.0008739	0.0000502	-0.0000179	0.0000295	0.0000852	0.0000364	87.28123	5.640218	2.605946	0.164805	61.56051	3.827686
93292-08J*	1.3	27/06/2018 09:43	1.29E-13	3.603702	0.0045044	1.137249	0.0026	0.0145251	0.0001601	0.0005237	0.0002601	0.0020475	0.0000712	83.67014	0.69064	2.645233	0.022652	62.47275	0.52584
93292-08K*	1.4	27/06/2018 12:03	2.32E-14	0.7783968	0.0068029	0.205176	0.0004401	0.0025834	0.0000811	0.0000712	0.0000423	0.0007975	0.0000433	69.8034	1.999271	2.642095	0.072394	62.3999	1.680593
93292-08L*	1.5	27/06/2018 13:38	4.79E-16	0.0142143	0.0024083	0.0042388	0.0000715	0.0001448	0.0000323	0.0000425	0.0000246	0.000056	0.0000344	-4.49892	74.75576	-0.15108	2.510239	-3.63407	60.44197
93292-08M*	6	27/06/2018 14:30	1.76E-14	0.6402946	0.0034059	0.1555251	0.0003901	0.0021069	0.0000701	0.0001139	0.0000246	0.0008591	0.0000423	60.71556	2.080763	2.494294	0.084794	58.96547	1.9722
93292-08N*	12	27/06/2018 16:47	3.97E-15	0.1345217	0.002508	0.0351245	0.0001702	0.0005215	0.0000482	0.0000168	0.0000265	0.0001549	0.0000364	66.1604	8.438342	2.528133	0.319325	59.75236	7.423892
93292-08O*	24	27/06/2018 18:21	8.78E-15	0.256747	0.0027074	0.0777227	0.0002501	0.0010557	0.0000502	-0.0000374	0.0000255	0.000221	0.0000324	73.65003	4.025547	2.426473	0.130438	57.38736	3.036486
Blanks (mean ± s.d.)			4.32E-17	0.023161	0.0002	0.0003821	0.0000083	0.0001054	0.0000044	0.0015496	0.0000052	0.0002107	0.0000053						
Sample: CHX-7560A; Material: Impact melt; D: 1.006357 ± 0.0004496; ngrains: 4																			
93292-09A*	0.3	02/07/2018 10:36	7.78E-14	1.93105	0.004005	0.6880903	0.0019	0.0079312	0.0001101	0.0000185	0.00029	0.0005576	0.0000364	91.41835	0.966658	2.559065	0.027886	60.47134	0.648046
93292-09B*	0.4	02/07/2018 11:31	1.34E-14	0.3309724	0.0029069	0.1185772	0.0003201	0.0012126	0.0000771	-0.0000245	0.0000285	0.0001252	0.0000324	88.32666	3.187127	2.458886	0.086372	58.14175	2.009828
93292-09C*	0.7	02/07/2018 13:05	9.20E-15	0.223424	0.0027074	0.0813856	0.0003001	0.0009233	0.0000472	-0.0000067	0.00029	-0.0000129	0.0000513	101.5804	9.501566	2.781432	0.261065	65.63168	6.049718
93292-09D*	0.8	02/07/2018 13:58	7.79E-14	2.054352	0.0054037	0.6892725	0.0029	0.0074431	0.00059	0.0001775	0.0002601	0.0008356	0.0000354	88.26423	0.880207	2.624323	0.027986	61.98728	0.649831
93292-09E*	0.9	02/07/2018 16:17	5.96E-14	1.681881	0.0047043	0.5275812	0.0024	0.0060182	0.00046	0.0000668	0.0002501	0.0009934	0.0000413	82.54755	1.097784	2.625073	0.036647	62.00468	0.850928
93292-09F*	1.2	02/07/2018 17:54	1.76E-14	0.4658746	0.0031064	0.1559868	0.0003901	0.0019068	0.0000632	0.0000084	0.0002501	0.0002576	0.0000433	83.57007	3.927604	2.489675	0.117184	58.85803	2.725715
93292-09G*	1.3	02/07/2018 18:48	6.86E-14	2.003111	0.0051039	0.6068013	0.0018	0.0074796	0.0001201	0.0002268	0.0002401	0.0012761	0.0000592	81.52684	1.112525	2.68497	0.037214	63.39494	0.863429
93292-09H*	1.4	02/07/2018 21:07	1.96E-15	0.0586245	0.00201	0.0173471	0.0007	0.0002678	0.0000283	0.0000467	0.0002101	0.0000195	0.0000523	93.99651	32.28654	3.172314	1.101179	74.66706	25.39071
93292-09I*	6	02/07/2018 22:40	4.89E-15	0.1532396	0.0024083	0.0433064	0.0001602	0.0004936	0.0000482	-0.0000057	0.0002001	0.0000968	0.0000553	80.95899	12.70927	2.857434	0.449378	67.39203	10.40342
93292-09J*	24	02/07/2018 23:34	1.02E-15	0.0296502	0.0018111	0.0090486	0.0000834	0.0001235	0.0000353	0.0000618	0.0002501	0.0000756	0.0000265	34.00767	49.85532	1.114818	1.644115	26.59238	38.93103
Blanks (mean ± s.d.)			4.32E-17	0.023161	0.0002	0.0003821	0.0000083	0.0001054	0.0000044	0.0015496	0.0000052	0.0002107	0.0000053						
Sample: CHX-7560A; Material: Impact melt; D: 1.005215 ± 0.0005199; ngrains: 4																			
93292-10A	0.3	06/07/2018 01:57	8.20E-14	2.233084	0.0044037	0.7255172	0.0019	0.0089873	0.0001301	0.0001831	0.0002101	0.0016403	0.0000461	78.48961	0.829479	2.409978	0.026151	57.00333	0.608912
93292-10B*	0.7	06/07/2018 02:52	1.54E-14	0.3734602	0.002706	0.1361413	0.0004501	0.0018759	0.00032	0.0000363	0.0002301	0.0000606	0.0000292	95.67134	4.112415	2.618003	0.112903	61.84053	2.621828
93292-10C*	0.8	06/07/2018 05:09	8.77E-14	2.441152	0.0036045	0.7762925	0.003	0.0087913	0.00054	0.0002052	0.0002901	0.0012647	0.0000711	84.96125	1.088146	2.665253	0.035821	62.93741	0.831327

93292-10D*	0.9	06/07/2018 06:46	2.40E-14	0.6326505	0.0034048	0.2119863	0.0004801	0.0024925	0.0000791	0.0000805	0.0002601	0.0003425	0.0000511	84.49513	3.317232	2.515668	0.098968	59.46254	2.301242
93292-10E*	1.2	06/07/2018 07:42	1.77E-14	0.4645127	0.0028058	0.1566119	0.0003101	0.0022013	0.00031	0.0000243	0.0002301	0.0001552	0.0000531	90.30576	4.374295	2.671827	0.129631	63.08997	3.00819
93292-10F*	1.3	06/07/2018 09:18	1.43E-13	4.296263	0.0049033	1.267479	0.0037	0.0155349	0.00018	0.0003298	0.0002501	0.0033391	0.0000551	77.19031	0.512041	2.610159	0.019096	61.65836	0.443482
93292-10G*	6	06/07/2018 10:14	7.68E-14	2.051275	0.0046035	0.6797596	0.0026	0.0077455	0.00054	0.0003047	0.0002801	0.0008502	0.0000372	88.39552	0.950249	2.661207	0.030275	62.84353	0.70264
93292-10H*	24	06/07/2018 11:51	9.88E-15	0.3402574	0.0059027	0.0874401	0.0005201	0.0118994	0.00018	0.0000692	0.0000256	0.000236	0.0001101	80.8707	9.929033	3.140221	0.38218	73.92693	8.815804
Blanks (mean ± s.d.)			3.99E-17	0.02131	0.00018	0.0003532	0.0000085	0.0000995	0.0000042	0.0014919	0.0000055	0.0002	0.0000037						
Sample: CHX-7560A; Material: Impact melt; D: 1.005215 ± 0.0005199; ngrains: 4																			
93292-11A*	0.3	09/07/2018 12:39	6.25E-14	1.669054	0.0031052	0.553507	0.0016	0.0063436	0.0001001	-0.0000149	0.0001901	0.0006896	0.0000382	87.60604	0.967962	2.634854	0.030088	62.23179	0.698549
93292-11B*	0.7	09/07/2018 13:34	1.23E-14	0.3227269	0.0029056	0.1091041	0.0003101	0.0012068	0.0000611	0.0000363	0.0002301	0.0000658	0.0000322	94.53434	5.142864	2.789543	0.152179	65.81961	3.52612
93292-11C*	0.8	09/07/2018 15:07	7.47E-14	2.048937	0.0039042	0.661162	0.0018	0.0080406	0.0000941	0.0001399	0.0002501	0.0009153	0.0000412	87.03912	0.946242	2.69069	0.030188	63.52765	0.70036
93292-11D*	0.9	09/07/2018 17:26	2.40E-14	0.6349784	0.0022074	0.2127938	0.0004001	0.0025941	0.0000731	-0.0001447	0.0000523	0.0001645	0.0000292	90.98735	1.527047	2.70717	0.044893	63.90995	1.041302
93292-11E*	1.2	09/07/2018 18:21	1.52E-14	0.3932369	0.0030054	0.1341607	0.0003101	0.0016108	0.0000661	-0.0000907	0.0000453	0.0000907	0.0000362	91.8189	3.01306	2.683454	0.085987	63.35978	1.995113
93292-11F*	1.3	09/07/2018 19:56	1.29E-13	3.798843	0.004004	1.142394	0.0024	0.0141809	0.00018	0.0005052	0.0000952	0.0024092	0.0000831	81.80613	0.682398	2.713973	0.023369	64.06774	0.542005
93292-11G*	6	09/07/2018 22:14	1.19E-14	0.3628316	0.0032051	0.1055776	0.0002701	0.0013342	0.0000591	-0.0000423	0.0000217	0.0002829	0.0000362	76.06272	3.202073	2.606827	0.107583	61.58096	2.498644
93292-11H*	24	09/07/2018 23:07	1.81E-15	0.0734709	0.0016101	0.0160358	0.0001103	0.0002714	0.0000333	-0.0001555	0.0000473	0.0000455	0.0000273	69.6323	11.97135	3.16724	0.541973	74.55007	12.49748
Blanks (mean ± s.d.)			3.99E-17	0.02131	0.00018	0.0003532	0.0000085	0.0000995	0.0000042	0.0014919	0.0000055	0.0002	0.0000037						

Run ID	Power (%)	Run Date & Time	^{39}Ar (moles)	^{40}Ar (V)	± σ	^{39}Ar (V)	± σ	^{38}Ar (V)	± σ	^{37}Ar (V)	± σ	^{36}Ar (V)	± σ	%^{40}Ar*	± σ	^{40}Ar*/ ^{39}Ar	± σ	Age (Ma)	± σ
Sample: CHX-7064A; Material: Impact melt; D: 1.009502 ± 0.0006813; ngrains: 1																			
93294-01A	0.5	13/04/2018 19:51	9.69E-15	1.185624	0.0019839	0.0857111	0.0002002	0.0020211	0.0000543	0.002612	0.000075	0.0033353	0.0000331	18.21202	0.85556	2.521495	0.122916	59.59802	2.857883
93294-01B	1	13/04/2018 21:23	2.90E-15	0.0862922	0.0013737	0.0256212	0.0001004	0.0004273	0.0000414	0.0022111	0.000068	0.0002281	0.0000163	46.71511	5.975665	1.582297	0.201087	37.62809	4.732578
93294-01C	1.5	13/04/2018 22:12	3.41E-17	0.0092587	0.0010325	0.0003014	0.0000352	0.0002225	0.0000484	-0.0000051	0.0000095	0.000007	0.0000143	77.22368	48.38894	23.64473	14.85519	494.082	271.6172
93294-01D	2	14/04/2018 00:19	5.22E-17	0.0092826	0.001044	0.0004619	0.0000372	0.0000684	0.0000375	0.0000305	0.0000092	0.0000383	0.0000143	-19.7556	47.5766	-3.98643	9.595681	-98.4242	243.4819
93294-01E	2.5	14/04/2018 01:42	5.00E-17	0.0089182	0.0010284	0.0004422	0.0000343	0.0000896	0.0000335	0.0000114	0.0000112	0.0000271	0.0000133	10.72857	46.28147	2.164592	9.336269	51.28068	218.0757
93294-01F	3	14/04/2018 02:28	1.09E-16	0.0119206	0.0010461	0.0009604	0.0000391	0.0001069	0.0000385	0.0000287	0.0000098	0.0000423	0.0000153	-3.39301	39.39664	-0.42149	4.89402	-10.1569	118.265
93294-01G	3.5	14/04/2018 04:34	1.89E-16	0.0143067	0.0010835	0.0016695	0.000042	-0.0000064	0.0000414	0.0000083	0.0000098	0.0000047	0.0000153	90.79479	33.6013	7.76981	2.821879	177.6838	61.46274
93294-01H	4	14/04/2018 05:57	2.18E-16	0.0152358	0.0010307	0.0019254	0.000046	0.0000855	0.0000454	0.0000203	0.0000122	0.0000001	0.0000143	101.2327	29.73928	8.003535	2.296584	182.7674	49.88097
93294-01I	4.5	14/04/2018 06:45	3.74E-16	0.0207311	0.0010369	0.0033067	0.0000489	0.0000548	0.0000414	0.0000041	0.0000102	0.0000044	0.0000163	93.89639	24.47734	5.876607	1.506171	135.9709	33.57119
93294-01J	5	14/04/2018 08:51	5.64E-16	0.0288672	0.0011455	0.0049955	0.0000528	0.0000762	0.0000404	-0.0000063	0.0000098	0.0000345	0.0000153	64.11558	16.55274	3.697755	0.94421	86.74213	21.62644
Blanks (mean ± s.d.)			1.31E-17	0.0075001	0.0007999	0.0001157	0.0000093	0.000073	0.000006	0.0003494	0.0000022	0.0000692	0.000003						
Sample: CHX-7064A; Material: Impact melt; D: 1.006357 ± 0.0004496; ngrains: 4																			
93294-08A	0.3	29/06/2018 05:32	2.70E-14	1.71381	0.0033061	0.2387954	0.00082	0.0030144	0.0000881	0.0002162	0.0000344	0.0038796	0.0000632	32.97552	1.124814	2.361884	0.081446	55.88315	1.897577
93294-08B	0.7	29/06/2018 06:29	6.37E-15	0.3039263	0.0031064	0.0563936	0.0002301	0.0007887	0.0000442	0.0000877	0.0000285	0.0007257	0.0000364	30.01743	3.763127	1.614881	0.202064	38.39479	4.753535
93294-08C	0.8	29/06/2018 08:05	2.17E-14	1.002037	0.0043046	0.1924354	0.0003701	0.0026183	0.0000761	0.0003491	0.0000443	0.0024226	0.0000533	29.39287	1.664717	1.52796	0.086665	36.34882	2.041099
93294-08D	0.9	29/06/2018 10:23	6.92E-15	0.1576191	0.0024083	0.0612081	0.0002202	0.0007693	0.0000542	0.0001471	0.0000265	0.0002647	0.0000384	54.11266	7.523178	1.391266	0.192366	33.12658	4.538607
93294-08E	1.6	29/06/2018 11:16	2.04E-14	0.4505003	0.0034059	0.1801768	0.0003901	0.0025686	0.0000761	0.0006833	0.0000364	0.0006977	0.0000394	60.68074	2.786118	1.515643	0.068784	36.0587	1.620228
93294-08F	12	29/06/2018 12:51	2.25E-14	0.7118637	0.0027074	0.1993087	0.0003901	0.0030261	0.0000373	0.001533	0.00032	0.0016001	0.0000443	42.73511	2.815063	1.5273	0.101319	36.33328	2.386234
93294-08G	16	29/06/2018 15:08	4.41E-15	0.1181628	0.0023087	0.0389963	0.0001702	0.0003733	0.0000472	0.0000166	0.0000304	0.0001268	0.0000354	68.59167	9.342402	2.073548	0.279751	49.15284	6.542099
93294-08H	20	29/06/2018 16:01	8.62E-15	0.2306753	0.0018111	0.0763163	0.0002301	0.0007814	0.0000532	0.000023	0.0000285	0.0002237	0.0000344	71.47697	4.597579	2.155331	0.137823	51.06435	3.219636
93294-08I	22	29/06/2018 17:35	8.68E-15	0.2107676	0.0024083	0.0768238	0.0002601	0.0008707	0.0000552	0.0000214	0.0000265	0.000179	0.0000344	75.08816	5.121123	2.055079	0.13841	48.72089	3.237538
93294-08J	24	29/06/2018 19:52	8.15E-15	0.1948292	0.0027074	0.0721102	0.0002202	0.0009552	0.0000502	-0.0000602	0.0000295	0.0000638	0.0000354	88.79539	5.787453	2.392215	0.152571	56.58967	3.553297
93294-08K	26	29/06/2018 20:46	7.50E-15	0.2008907	0.0022091	0.0663743	0.0002401	0.0008498	0.0000512	0.0000951	0.0000523	0.0002041	0.0000344	71.84534	5.433369	2.170304	0.16276	51.4141	3.801458
93294-08L	28	29/06/2018 22:21	6.52E-15	0.1590857	0.0024083	0.0576655	0.0002401	0.0007827	0.0000542	-0.0000702	0.0000255	0.0001098	0.0000314	77.35021	6.261079	2.127472	0.169467	50.41342	3.960301
93294-08M	30	30/06/2018 00:38	6.52E-15	0.1595985	0.0028071	0.0577022	0.0002401	0.0007114	0.0000562	-0.0000712	0.0000246	0.0001104	0.0000344	77.2764	6.861749	2.130762	0.185736	50.49031	4.340289
93294-08N	32	30/06/2018 01:32	8.30E-15	0.2106445	0.0027074	0.0734524	0.0002901	0.0007679	0.0000502	-0.0000208	0.0000275	0.0001311	0.0000334	80.95285	5.06439	2.315427	0.142125	54.80044	3.313294
93294-08O	34	30/06/2018 03:07	1.01E-14	0.2501008	0.0031064	0.0890549	0.0002701	0.0010233	0.0000482	0.0000831	0.0000265	0.0001876	0.0000334	79.13591	4.331879	2.217672	0.118476	52.5201	2.765446
93294-08P	36	30/06/2018 05:24	9.26E-15	0.2308496	0.0026077	0.081936	0.0002401	0.0009561	0.0000452	-0.0000548	0.0000265	0.0001462	0.0000344	79.98743	4.718812	2.247296	0.130347	53.21144	3.041381
93294-08Q	38	30/06/2018 06:18	8.18E-15	0.2051984	0.0028071	0.0724251	0.0002202	0.0008993	0.0000522	-0.0000766	0.0000255	0.0001037	0.0000344	83.18155	5.356131	2.349782	0.148078	55.60117	3.450533
93294-08R	40	30/06/2018 07:53	8.61E-15	0.2155881	0.0027074	0.0762011	0.0002301	0.0009826	0.0000552	-0.0000428	0.0000334	0.000157	0.0000334	77.33742	4.956639	2.182006	0.137375	51.6874	3.208062
93294-08S	50	30/06/2018 10:09	2.12E-14	0.5566128	0.0032062	0.1879042	0.0003601	0.0021783	0.0000731	0.0000604	0.0000622	0.0003369	0.0000433	82.42089	2.500671	2.435667	0.07281	57.60137	1.694743
Blanks (mean ± s.d.)			4.32E-17	0.023161	0.0002	0.0003821	0.0000083	0.0001054	0.0000044	0.0015496	0.0000052	0.0002107	0.0000053						
Sample: CHX-7064A; Material: Impact melt; D: 1.006357 ± 0.0004496; ngrains: 4																			
93294-09A	0.3	03/07/2018 03:51	8.26E-14	2.169162	0.0052038	0.7307222	0.0021	0.0080801	0.0001301	0.0003208	0.0000285	0.0016649	0.0000443	77.79588	0.687049	2.303972	0.020837	54.53339	0.485822
93294-09B	0.7	03/07/2018 04:47	1.65E-14	0.3846133	0.0032062	0.1458074	0.0003401	0.0015356	0.0000562	0.000051	0.0000255	0.0003803	0.0000364	71.1127	3.029176	1.871297	0.078343	44.41692	1.836882
93294-09C	0.8	03/07/2018 06:21	9.02E-14	3.525805	0.010002	0.7986192	0.0021	0.0110585	0.0001401	0.0006723	0.0000533	0.0066176	0.0000652	44.89312	0.641646	1.977833	0.028699	46.91312	0.671979
93294-09D	0.9	03/07/2018 08:41	7.42E-14	2.601653	0.0037054	0.6563227	0.0018	0.0086898	0.0001401	0.0004803	0.0000463	0.0042469	0.0000652	52.16359	0.77147	2.063298	0.031225	48.91312	0.730301
93294-09E	1.6	03/07/2018 09:36	9.62E-14	2.348987	0.0036056	0.8510555	0.002	0.0114565	0.0001601	0.0011738	0.00036	0.0027366	0.0000533	67.67823	1.036787	1.864323	0.029122	44.25338	0.682874
93294-09F	12	03/07/2018 11:12	1.91E-14	0.5984896	0.0032062	0.1693808	0.0004301	0.0024539	0.0000711	0.0004999	0.0000453	0.0010411	0.0000433	52.1857	2.279788	1.841683	0.080122	43.72243	1.879329
93294-09G	20	03/07/2018 13:30	5.60E-15	0.1767369	0.0027074	0.0495539	0.0001902	0.0006166	0.0000482	-0.0000889	0.0000255	0.0002985	0.0000384	47.05958	6.748608	1.672919	0.238698	39.7596	5.611125
93294-09H	24	03/07/2018 14:24	6.49E-15	0.1668703	0.0029069	0.0574392	0.0002301	0.0007181	0.0000452	-0.0000108	0.0000285	0.0001635	0.0000354	70.4078	6.75024	2.040097	0.192587	48.3704	4.505681
93294-09I	28	03/07/2018 15:58	8.53E-15	0.2054753	0.0026077	0.0755193	0.0002401	0.0008339	0.0000502	0.0000349	0.0000295	0.0001101	0.0000314	84.82438	4.926037	2.302487	0.130764	54.49876	3.048952
93294-09J	32	03/07/2018 18:14	1.49E-14	0.341598	0.0031064	0.1319721	0.0002901	0.0014476	0.0000622	-0.0000963	0.0000236	0.0002049	0.0000354	80.66907	3.332884	2.082012	0.084075	49.35076	1.96592
93294-09K	36	03/07/2018 19:06	1.64E-14	0.3855261	0.0028071	0.1452978	0.0003301	0.0016169	0.0000642	-0.0000477	0.0000255	0.0001847	0.0000374	85.07134	3.071997	2.251127	0.079828	53.30083	1.862526
93294-09L	40	03/07/2018 20:41	1.90E-14	0.4454644	0.0032062	0.1678704	0.0003701	0.0019969	0.0000651	-0.0000183	0.0000246	0.0001842	0.0000324	87.44527	2.397423	2.314401	0.061486	54.77652	1.433402
93294-09M	45	03/07/2018 22:57	2.18E-14	0.5300077	0.0032062	0.1926352	0.0004101	0.002252	0.0000681	-0.000042	0.0000275	0.0002515	0.0000364	85.42525	2.219253	2.344118	0.059485	55.46917	1.386227
93294-09N	50	03/07/2018 23:51	2.72E-14	0.6475869	0.0034059	0.2407532	0.00076	0.0025783	0.0000771	0.0000145	0.0000295	0.0002965	0.0000394	86.42939	1.960956	2.318911	0.051764	54.88165	1.206683
Blanks (mean ± s.d.)			4.32E-17	0.023161	0.0002	0.0003821	0.0000083	0.0001054	0.0000044	0.0015496	0.0000052	0.0002107	0.0000053						
Sample: CHX-7064A; Material: Impact melt; D: 1.005215 ± 0.0005199; ngrains: 4																			
93294-10A	0.3	06/07/2018 16:12	2.68E-14	1.228994	0.0043038	0.2374968	0.0007201	0.0031719	0.0000871	0.00023	0.0000276	0.0023091	0.0000541	44.88139	1.377158	2.317856	0.071384	54.85706	1.664086
93294-10B	0.7	06/07/2018 17:10	6.49E-15	0.2261202	0.0024067	0.0574087	0.0002002	0.000658	0.0000452	0.0001904	0.0000315	0.0004094	0.0000322	50.37017	4.485099	1.982056	0.175523	47.01199	4.109549
93294-10C	0.8	06/07/2018 19:25	3.64E-14	0.8915344	0.0039042	0.3219661	0.0014	0.0040938	0.0000791	0.0005508	0.0000364	0.0009617	0.0000412	71.04998	1.49888	1.963884	0.041553	46.58649	0.973117
93294-10D	0.9	06/07/2018 21:02	9.56E-15	0.2031375	0.0028058	0.0845636	0.0002901	0.0010109	0.0000452	0.0001246	0.0000276	0.0001703	0.0000342	78.2067	5.382044	1.875036	0.126635	44.50457	2.96904
93294-10E	1.6	06/07/2018 21:55	4.79E-14	1.015381	0.0041039	0.4239941	0.0015	0.0059368	0.0001101	0.0010313	0.0000463	0.0010513	0.0000452	74.46521	1.444331	1.780635	0.034471	42.28994	0.80919
93294-10F	12	07/07/2018 00:13	1.90E-15	0.061852	0.0021077	0.016827	0.0001203	0.0002601	0.0000382	0.000037	0.0000246	0.0001277	0.0000332	41.5438	16.6043	1.52477	0.607462	36.27367	14.3073
93294-10G	24	07/07/2018 01:46	1.16E-14	0.2954969	0.0026062	0.1027702	0.0002701	0.0012803	0.0000522	-0.0000187	0.0000285	0.0002661	0.0000362	72.77275	3.856003	2.086898	0.109248	49.465	2.554359

93294-10H	28	07/07/2018 02:40	1.16E-14	0.2761388	0.0028058	0.1025213	0.0002601	0.0012701	0.0000462	-0.0000124	0.0000295	0.0002288	0.0000352	75.01719	4.058981	2.015233	0.107291	47.7886	2.510934
93294-10I	32	07/07/2018 04:57	1.42E-14	0.3435961	0.0033049	0.1260888	0.0003201	0.0015163	0.0000601	0.0001042	0.0000217	0.0002463	0.0000352	80.20573	3.32183	2.180828	0.088079	51.65987	2.056911
93294-10J	36	07/07/2018 06:33	1.54E-14	0.375441	0.0033049	0.1366238	0.0003101	0.0015382	0.0000621	-0.0000588	0.0000236	0.0001431	0.0000342	87.77734	2.986978	2.405405	0.079307	56.89684	1.846701
93294-10K	40	07/07/2018 07:26	1.81E-14	0.4400496	0.0029056	0.1605813	0.0003601	0.001872	0.0000651	-0.0000141	0.0000266	0.0001609	0.0000312	88.90589	2.322611	2.429946	0.061724	57.4682	1.436821
93294-10L	45	07/07/2018 09:43	1.94E-14	0.496766	0.0034048	0.1718759	0.0004001	0.002049	0.0000711	-0.0000146	0.0000276	0.0002152	0.0000352	86.90356	2.32621	2.505186	0.06515	59.21879	1.515109
93294-10M	50	07/07/2018 11:19	2.24E-14	0.5802223	0.0033049	0.1983382	0.0004001	0.0022636	0.0000691	-0.0000061	0.0000256	0.0002826	0.0000352	85.39348	1.97826	2.49167	0.056244	58.90445	1.30821
Blanks (mean ± s.d.)			3.99E-17	0.02131	0.00018	0.0003532	0.0000085	0.0000995	0.0000042	0.0014919	0.0000055	0.0002	0.0000037						
Sample: CHX-7064A; Material: Impact melt; D: 1.005215 ± 0.0005199; ngrains: 4																			
93294-11A	0.3	10/07/2018 04:07	7.98E-14	2.094841	0.0051032	0.7062899	0.0019	0.007991	0.0001101	0.0003087	0.0000236	0.001583	0.0000521	78.25811	0.809225	2.315627	0.024254	54.8051	0.565417
93294-11B	0.7	10/07/2018 05:02	1.75E-14	0.3621476	0.0031052	0.1552529	0.0003601	0.001944	0.0000542	0.0000666	0.0000266	0.0002479	0.0000372	80.59714	3.290019	1.875452	0.075024	44.51433	1.758972
93294-11C	0.8	10/07/2018 07:19	5.66E-14	1.057783	0.0039042	0.5008629	0.0015	0.0061699	0.0000991	0.0006164	0.0000315	0.0007058	0.0000402	83.37093	1.246224	1.757079	0.026069	41.7369	0.61215
93294-11D	0.9	10/07/2018 08:55	1.04E-14	0.1868016	0.002706	0.0921144	0.0002501	0.0011839	0.0000502	0.0002094	0.0000266	0.0000661	0.0000332	95.79711	5.742035	1.93965	0.112988	46.01887	2.646859
93294-11E	1.6	10/07/2018 09:48	2.38E-14	0.3787694	0.0031052	0.2106283	0.0004901	0.0027959	0.0000781	0.0004817	0.0000315	0.0002309	0.0000372	89.01946	3.172139	1.598249	0.055586	38.00347	1.307947
93294-11F	12	10/07/2018 12:07	4.34E-14	0.7578554	0.0036045	0.3841072	0.0015	0.0053515	0.0000931	0.0008231	0.0000642	0.0006378	0.0000362	81.05182	1.629929	1.596551	0.031918	37.96353	0.751046
93294-11G	24	10/07/2018 13:43	9.37E-15	0.2252523	0.002706	0.0829639	0.0002701	0.0009166	0.0000482	-0.0000074	0.0000246	0.0002583	0.0000362	65.5559	5.054026	1.775274	0.135375	42.16408	3.178047
93294-11H	28	10/07/2018 14:36	8.54E-15	0.1971953	0.002706	0.0755665	0.0002601	0.0007485	0.0000482	-0.0000269	0.0000256	0.0001102	0.0000362	82.52078	5.817637	2.14741	0.148745	50.87929	3.475148
93294-11I	32	10/07/2018 16:51	1.02E-14	0.2425072	0.002706	0.0906782	0.0002701	0.0010957	0.0000382	0.0000746	0.0000285	0.0001439	0.0000332	84.03564	4.400071	2.242477	0.114992	53.099	2.683279
93294-11J	36	10/07/2018 18:25	1.03E-14	0.2434261	0.0028058	0.0914522	0.0002402	0.0010894	0.0000522	-0.0000132	0.0000276	0.0001187	0.0000312	85.12667	4.176735	2.259792	0.108015	53.50299	2.519901
93294-11K	40	10/07/2018 19:18	1.15E-14	0.2847254	0.0028058	0.1021669	0.0003901	0.001164	0.0000611	-0.0000655	0.0000266	0.0001423	0.0000312	83.75479	3.564756	2.327312	0.096855	55.0775	2.257579
93294-11L	45	10/07/2018 21:35	1.18E-14	0.2984987	0.0028058	0.1041932	0.0003101	0.0012868	0.0000542	0.0000132	0.0000266	0.0002058	0.0000402	79.66482	4.230921	2.276504	0.119253	53.89283	2.781475
93294-11M	50	10/07/2018 23:10	1.27E-14	0.3276787	0.0029056	0.1121066	0.0002901	0.0013862	0.0000542	0.000038	0.0000227	0.0002045	0.0000322	82.02942	3.182682	2.391851	0.090626	56.58119	2.110628
Blanks (mean ± s.d.)			3.99E-17	0.02131	0.00018	0.0003532	0.0000085	0.0000995	0.0000042	0.0014919	0.0000055	0.0002	0.0000037						

Run ID	Power (%)	Run Date & Time	^{39}Ar (moles)	^{40}Ar (V)	± σ	^{39}Ar (V)	± σ	^{38}Ar (V)	± σ	^{37}Ar (V)	± σ	^{36}Ar (V)	± σ	%^{40}Ar*	± σ	^{40}Ar*/ ^{39}Ar	± σ	Age (Ma)	± σ
**Sample: CHX-7350; Material: Impact melt; D: 1.009502 ± 0.0006813; ngrains: 1**																			
93302-01A*	0.2	15/04/2018 19:37	-6.17E-18	0.0000122	0.0008541	-0.0000546	0.0000391	-0.0000053	0.0000267	-0.000003	0.000082	-0.00001	0.0000114	24297.95	1.697102e	-54.5749	76.64429	-2336.17	6704.208
93302-01B*	0.3	15/04/2018 20:22	1.46E-13	3.778158	0.0031038	1.293701	0.0019	0.0163567	0.0001201	0.0020031	0.000063	0.0020123	0.0000311	84.64584	0.27033	2.46746	0.008957	58.34124	0.208412
93302-01C*	0.4	15/04/2018 21:55	3.60E-15	0.0997992	0.0013582	0.031851	0.0000964	0.0004156	0.0000414	0.0000283	0.0000097	0.0000677	0.0000192	80.04478	6.027516	2.50332	0.185595	59.17539	4.316221
93302-01D*	0.5	15/04/2018 22:44	1.87E-15	0.0507787	0.0009977	0.0165888	0.0004901	0.0001471	0.0000494	-0.0000062	0.00007	0.0000272	0.0000133	83.87337	8.400119	2.56223	0.263607	60.54491	6.12583
93302-01E*	0.6	16/04/2018 00:50	4.85E-16	0.0134331	0.0008974	0.004295	0.000043	-0.0000921	0.0000553	-0.0000177	0.000088	-0.0000017	0.0000124	102.3174	29.98086	3.192585	0.912884	75.13442	21.0436
93302-01F*	0.7	16/04/2018 02:12	2.40E-16	0.0038793	0.0013595	0.0021274	0.000045	0.0000891	0.0000464	-0.0000205	0.000081	-0.0000127	0.0000133	192.6145	130.099	3.501624	2.025455	82.24432	46.50711
93302-01G*	0.8	16/04/2018 03:00	1.85E-15	0.0513834	0.0009818	0.0163584	0.0004901	0.0002091	0.0000267	0.0000396	0.000095	0.0000043	0.0000212	98.31899	12.77897	3.082911	0.407855	72.60446	9.414953
93302-01H	0.9	16/04/2018 05:05	1.26E-16	0.0025443	0.0009233	0.0011168	0.0000607	0.0000031	0.0000504	0.0000176	0.0001	0.0000352	0.0000124	-305.766	191.1897	-6.96182	3.550961	-175.543	94.02858
93302-01I	1	16/04/2018 06:29	3.35E-17	0.0006068	0.0008704	0.0002963	0.000046	-0.0001034	0.0000464	-0.0000259	0.000076	-0.0000205	0.0000114	1063.147	1637.531	21.55747	12.66925	455.5092	236.6434
93302-01J	5	16/04/2018 07:16	6.62E-15	0.2290963	0.0015272	0.0586274	0.0002502	0.0009176	0.0000464	0.0000138	0.000079	0.0002855	0.0000282	62.85975	3.776744	2.451674	0.146923	57.97392	3.419141
Blanks (mean ± s.d.)			1.31E-17	0.0070776	0.0008015	0.0001157	0.0000093	0.000073	0.000006	0.0003494	0.0000022	0.0000692	0.000003						
Sample: CHX-7350; Material: Impact melt; D: 1.007494 ± 0.0002349; ngrains: 1																			
93302-04A	0.5	03/06/2018 05:12	5.89E-14	2.35354	0.0036036	0.5213685	0.0016	0.0066531	0.00013	0.0003212	0.0000273	0.0038917	0.0000632	50.99104	0.821927	2.297003	0.037649	54.37089	0.877899
93302-04B*	0.9	03/06/2018 06:08	1.43E-13	3.82874	0.0330004	1.26633	0.0039	0.0156978	0.00016	0.0008032	0.00034	0.0021044	0.0000702	84.15491	1.293961	2.538906	0.033354	60.00279	0.775321
93302-04C*	1.3	03/06/2018 08:27	5.79E-14	1.711452	0.0034038	0.511991	0.0016	0.0065831	0.00011	0.0006979	0.0000551	0.0013374	0.0000533	77.77437	0.968384	2.594671	0.032987	61.29862	0.766243
93302-04D*	2.2	03/06/2018 10:04	2.10E-14	0.6468068	0.0034038	0.1861339	0.0003501	0.0021817	0.0000781	0.0001715	0.00025	0.0006363	0.0000692	71.34099	3.430963	2.473941	0.11862	58.49203	2.759685
93302-04E*	3.4	03/06/2018 13:33	1.56E-14	0.4741018	0.0028046	0.1380716	0.00047	0.0016082	0.0000541	0.0000949	0.00026	0.0004717	0.0000344	70.83177	2.743409	2.427006	0.093911	57.39976	2.186154
93302-04F*	4.7	03/06/2018 14:28	5.92E-16	0.0218006	0.0019067	0.0052423	0.0000713	0.0000768	0.0000401	0.0000698	0.00024	0.0000121	0.0000493	92.21101	74.99922	3.838251	3.121739	89.95723	71.37414
93302-04G*	5.9	03/06/2018 16:01	1.25E-15	0.0485363	0.0020064	0.0110453	0.00072	0.0000651	0.0000551	0.0000587	0.00023	0.0000605	0.0000543	66.07642	36.20954	2.900763	1.602195	68.39484	37.07138
93302-04H*	8	03/06/2018 18:17	8.60E-16	0.0439825	0.0018071	0.0076125	0.0000922	0.0000743	0.0000381	-0.0000189	0.00021	0.000048	0.0000533	66.23787	38.82543	3.816287	2.240739	89.45499	51.24556
93302-04I*	10	03/06/2018 19:09	1.74E-15	0.0897473	0.0021061	0.0154171	0.0001202	0.0002615	0.0000441	0.0000385	0.00025	0.0002509	0.0000364	17.71226	14.55992	1.029429	0.848079	24.56933	20.10416
93302-04J*	11.6	03/06/2018 20:42	2.94E-15	0.0917254	0.0021061	0.0260165	0.0002101	0.0005171	0.0000631	-0.000026	0.00023	0.000183	0.0000582	39.67673	20.3535	1.39533	0.71619	33.22247	16.8966
93302-04K*	13.2	03/06/2018 22:59	3.16E-15	0.1127224	0.0018071	0.0280035	0.0002401	0.0003177	0.0000431	0.0000164	0.0003	0.0002105	0.0000354	44.63249	12.0723	1.792779	0.487203	42.57498	11.43496
93302-04L*	14.7	03/06/2018 23:52	3.38E-15	0.0935181	0.0020064	0.0299393	0.0001401	0.000293	0.0000441	-0.0000209	0.00029	0.0001026	0.0000543	66.61184	19.52858	2.075511	0.609109	49.19875	14.24389
93302-04M*	15.9	04/06/2018 01:26	3.76E-15	0.1247457	0.0021061	0.0332874	0.0001601	0.0003352	0.0000501	0.0000844	0.0000551	0.000136	0.0000354	69.30349	8.816263	2.59285	0.327327	61.25633	7.603598
93302-04N*	18.1	04/06/2018 03:44	5.04E-15	0.1501832	0.0022058	0.0445649	0.0002801	0.0004961	0.0000461	-0.0000299	0.0000541	0.0001493	0.0000413	69.75894	8.481456	2.345083	0.283521	55.49167	6.607054
93302-04O*	20	04/06/2018 04:36	7.34E-15	0.2149463	0.0023056	0.0649151	0.0002101	0.0005368	0.0000601	0.0000437	0.0000283	0.0001636	0.0000563	77.81577	7.945861	2.571114	0.261241	60.75134	6.070172
93302-04P*	21.8	04/06/2018 06:11	6.29E-15	0.1554067	0.0022058	0.0556604	0.0003001	0.0006301	0.0000421	-0.0000129	0.00024	0.0000767	0.0000553	85.02881	11.61685	2.368351	0.323026	56.03381	7.525393
93302-04Q*	24	04/06/2018 08:29	6.97E-15	0.2009449	0.0022058	0.0616836	0.0002301	0.0006524	0.0000491	0.0000097	0.0000223	0.0001533	0.0000632	77.34497	9.507413	2.513925	0.307942	59.42202	7.16056
93302-04R*	26.1	04/06/2018 09:23	7.10E-15	0.2010273	0.0021061	0.0628259	0.0003201	0.0005917	0.0000441	0.0000094	0.0000292	0.0001209	0.0000672	82.15815	10.0882	2.622874	0.321193	61.95364	7.458252
93302-04S*	28.8	04/06/2018 10:59	6.36E-15	0.1671003	0.0026049	0.0562678	0.0002301	0.0006497	0.0000441	-0.0000542	0.00018	0.0000825	0.0000622	84.35262	11.70995	2.498623	0.345342	59.06617	8.031817
93302-04T*	32	04/06/2018 15:46	8.09E-15	0.2313895	0.0023056	0.0715591	0.0002301	0.0008244	0.0000571	0.0000172	0.0000243	0.0002251	0.0000394	71.15232	5.239377	2.295558	0.167668	54.33719	3.909755
Blanks (mean ± s.d.)			5.94E-17	0.02027	0.00016	0.000526	0.0000063	0.0001563	0.000003	0.0015878	0.0000038	0.0002559	0.0000053						
Sample: CHX-7350; Material: Impact melt; D: 1.006357 ± 0.0004496; ngrains: 4																			
93302-06A	0.3	30/06/2018 18:29	1.28E-13	2.714761	0.0036056	1.131276	0.0035	0.0131903	0.0001501	0.000266	0.00031	0.0008087	0.0000334	91.55693	0.677411	2.191678	0.017625	51.91325	0.411533
93302-06B*	0.4	30/06/2018 19:24	3.84E-14	0.8414325	0.0033061	0.3394323	0.0022	0.0038257	0.00037	0.0000306	0.0002601	-0.0001	0.0000523	103.7114	2.428981	2.564446	0.062078	60.5964	1.442549
93302-06C*	0.5	30/06/2018 20:59	2.19E-14	0.5098847	0.0023087	0.193476	0.0003601	0.0022387	0.0000731	0.0000526	0.0000226	0.0000915	0.0000305	95.11227	1.905209	2.500606	0.049053	59.11227	1.140824
93302-06D*	0.7	30/06/2018 23:15	1.82E-14	0.4094337	0.0029069	0.160887	0.0005001	0.0020807	0.00027	0.0000426	0.0002301	0.0000858	0.0000324	94.23118	3.695767	2.392172	0.093633	56.58868	2.180663
93302-06E*	0.8	01/07/2018 00:09	1.47E-13	3.650871	0.004005	1.304882	0.0023	0.0157252	0.0001601	0.0004221	0.0000433	0.0011539	0.0000463	91.10355	0.411527	2.542831	0.012153	60.09405	0.282479
93302-06F*	0.9	01/07/2018 01:46	4.63E-14	1.110119	0.0037054	0.4097856	0.002	0.0047199	0.00035	0.0001795	0.0001901	0.0000999	0.0000553	98.0713	1.759195	2.650503	0.048793	62.59508	1.132601
93302-06G*	1	01/07/2018 04:03	2.96E-14	0.714038	0.0030067	0.26222	0.0014	0.0031694	0.00033	0.0000074	0.0002401	0.0000705	0.0000533	97.09661	2.805876	2.637303	0.07734	62.28864	1.79553
93302-06H*	1.2	01/07/2018 05:01	2.26E-14	0.5450847	0.0030067	0.2003207	0.0006301	0.0026458	0.00033	-0.0000499	0.0002201	0.0000568	0.0000513	96.45682	3.491064	2.617708	0.094691	61.83367	2.19892
93302-06I*	1.3	01/07/2018 06:36	2.00E-13	4.972959	0.0044045	1.770663	0.0042	0.0225611	0.00037	0.0004885	0.0002401	0.0013273	0.0000762	92.49547	0.526557	2.59148	0.016	61.22449	0.371667
93302-06J*	2.2	01/07/2018 08:55	1.63E-13	4.093701	0.0041049	1.443815	0.004	0.0183005	0.0001701	0.000579	0.0001601	0.0014296	0.0000742	90.24317	0.588769	2.552646	0.018079	60.32217	0.42017
93302-06K*	3.4	01/07/2018 09:50	5.49E-14	1.4702	0.0043046	0.4854182	0.0024	0.0057741	0.00047	0.0002972	0.0002501	0.0007301	0.0000692	86.12693	1.670479	2.602653	0.051905	61.48403	1.205578
93302-06L*	12	01/07/2018 11:26	3.24E-14	0.9050118	0.0036056	0.286731	0.0016	0.0035927	0.00036	0.0000507	0.0002301	0.0005353	0.0000602	82.60034	2.38857	2.60078	0.076422	61.44053	1.775065

93302-06M*	14	01/07/2018 13:47	5.84E-15	0.1889523	0.0022091	0.0517125	0.0002002	0.0006932	0.0000442	0	0.0003	0.0002425	0.0000364	61.6685	9.641424	2.247727	0.354915	53.22151	8.281173
93302-06N*	16	01/07/2018 14:42	3.38E-15	0.1162038	0.0019105	0.0299196	0.0001402	0.0003933	0.0000422	-0.0000042	0.0000295	0.0001527	0.0000364	60.57576	9.631827	2.34675	0.371457	55.53051	8.656081
93302-06O*	18	01/07/2018 16:16	3.64E-15	0.1021833	0.0024083	0.0321975	0.0001602	0.000435	0.0000452	0.0000155	0.0002301	0.00016	0.0000334	53.94927	14.8261	1.708211	0.471583	40.58903	11.0805
93302-06P*	20	01/07/2018 18:32	4.96E-15	0.1373114	0.0023087	0.0439281	0.0002102	0.0004119	0.0000572	0	0.0000295	0.0001441	0.0000324	68.63181	7.426068	2.139923	0.22907	50.70438	5.352289
93302-06Q*	22	01/07/2018 19:25	5.98E-15	0.1606652	0.0032062	0.052959	0.0002002	0.0005322	0.0000422	0.0000779	0.0000622	0.0000399	0.0000324	94.88049	6.900103	2.872978	0.201627	67.75185	4.666883
93302-06R*	24	01/07/2018 21:00	6.21E-15	0.1633729	0.0028071	0.0549616	0.0002002	0.0006201	0.0000432	0	0.0000295	0.0001326	0.0000314	75.74475	6.21397	2.245824	0.180436	53.1771	4.210198
93302-06S*	26	01/07/2018 23:16	6.16E-15		0.0027074	0.054501	0.0002002	0.00055	0.0000502	-0.000061	0.0000255	0.000112	0.0000314	77.84513	6.147036	2.338643	0.180884	55.34158	4.215584
93302-06T*	30	02/07/2018 00:10	1.26E-14	0.3342955	0.0026077	0.1114766	0.0002601	0.0015936	0.00031	-0.0000429	0.0002201	0.0000587	0.0000275	94.15408	4.165376	2.815911	0.124651	66.43048	2.887281
93302-06U*	34	02/07/2018 01:44	1.55E-14	0.408644	0.0026077	0.1376	0.0005401	0.0019066	0.00023	-0.0000278	0.0002501	0.0001655	0.0000305	87.5887	3.804208	2.594418	0.113531	61.29275	2.637213
93302-06V*	40	02/07/2018 04:01	1.67E-14	0.4257516	0.0028071	0.1475104	0.0004901	0.0020402	0.00032	-0.000065	0.00028	0.0000681	0.0000513	94.49132	4.894902	2.719841	0.141509	64.20385	3.281811
93302-06W*	50	02/07/2018 04:54	2.33E-14	0.6039255	0.0034059	0.2059017	0.0004501	0.0024966	0.0000761	0.0000454	0.0000285	0.0002705	0.0000374	86.98128	2.011156	2.545014	0.057395	60.14477	1.334074
Blanks (mean ± s.d.)		4.32E-17		0.023161	0.0002	0.0003821	0.0000083	0.0001054	0.0000044	0.0015496	0.0000052	0.0002107	0.0000053						
Sample: CHX-7350; Material: Impact melt; D: 1.006357 ± 0.0004496; ngrains: 4																			
93302-07A	0.3	04/07/2018 04:53	6.14E-14	1.358529	0.0044045	0.5431337	0.0017	0.0059453	0.00057	0.0001262	0.0002601	0.0004123	0.0000642	91.39359	1.768148	2.28033	0.044386	53.98206	1.035226
93302-07B*	0.4	04/07/2018 05:48	1.73E-14	0.3895876	0.002508	0.1527357	0.0005401	0.0016413	0.0000542	-0.0000248	0.0002601	0.00006	0.0000324	95.06677	4.286415	2.418468	0.109739	57.20099	2.554883
93302-07C*	0.7	04/07/2018 08:05	1.12E-14	0.2617213	0.0024083	0.09943	0.0003901	0.0015248	0.00028	0.0000306	0.0002401	-0.0000533	0.0000483	106.694	7.334354	2.801574	0.193112	66.09836	4.473877
93302-07D*	0.8	04/07/2018 09:42	1.10E-13	2.726884	0.0037054	0.9705226	0.0032	0.0115072	0.0002	0.0002157	0.0002101	0.0007351	0.0000563	92.3439	0.753202	2.588217	0.022738	61.1487	0.528214
93302-07E*	0.9	04/07/2018 10:37	4.16E-14	1.03497	0.0027074	0.36802	0.0019	0.0041872	0.00045	0.0000698	0.0001801	0.0003002	0.0000334	91.67006	1.356151	2.571635	0.040201	60.76345	0.934097
93302-07F*	1	04/07/2018 12:14	2.66E-14	0.665433	0.0030067	0.2353159	0.0005401	0.0029497	0.00032	0.0000447	0.0002701	0.0002691	0.0000334	88.25955	2.622045	2.489663	0.074368	58.85776	1.729817
93302-07G*	1.2	04/07/2018 13:11	2.12E-14	0.5282642	0.0032062	0.1875271	0.0006201	0.0023329	0.00026	0.0000215	0.0003	0.0002008	0.0000295	88.85068	3.434549	2.496656	0.097212	59.02042	2.260973
93302-07H*	1.3	04/07/2018 15:27	1.43E-13	3.593148	0.0039051	1.264981	0.0041	0.0155986	0.0001601	0.0003566	0.0002701	0.0010165	0.0000672	92.05346	0.693642	2.608404	0.021462	61.61761	0.498449
93302-07I*	1.8	04/07/2018 17:03	8.86E-14	2.309418	0.0051039	0.7836374	0.0027	0.0100448	0.0001101	0.0003445	0.0002701	0.0010173	0.0000394	87.60323	0.83891	2.575643	0.025987	60.85656	0.603789
93302-07J*	2.3	04/07/2018 17:59	2.09E-14	0.603146	0.002508	0.184704	0.0004401	0.0022549	0.0000701	0.0000712	0.0000802	0.0004569	0.0000374	77.97663	2.042794	2.540322	0.066207	60.03572	1.538988
93302-07K*	3.8	04/07/2018 20:16	9.90E-15	0.2734341	0.0026077	0.0875669	0.0003701	0.0014255	0.00029	0.0000517	0.0002501	0.0000759	0.0000572	92.68539	7.918808	2.8876	0.247702	68.09026	5.732253
93302-07L*	12	04/07/2018 21:51	7.92E-15	0.2310752	0.0024083	0.0701167	0.0002301	0.0008952	0.0000472	-0.0000278	0.0002101	0.0000905	0.0000463	87.68654	7.711314	2.881989	0.254226	67.9604	5.883667
93302-07M*	30	04/07/2018 22:44	2.84E-14	0.8130942	0.0032062	0.2509225	0.0007	0.0031735	0.00035	-0.0000791	0.0002201	0.0004913	0.0000344	81.45819	1.946273	2.632554	0.063275	62.17838	1.469089
93302-07N*	40	05/07/2018 01:05	1.97E-14	0.553659	0.0034059	0.17471	0.0004001	0.002017	0.0000661	0.000099	0.0000513	0.0003979	0.0000413	79.44166	2.415875	2.511787	0.075138	59.37229	1.747218
93302-07O*	50	05/07/2018 02:39	1.62E-14	0.4656695	0.0029069	0.1431718	0.0005001	0.0019055	0.00026	0.0000839	0.0002201	0.0003396	0.0000324	79.14174	3.299149	2.568278	0.107604	60.68543	2.500355
Blanks (mean ± s.d.)		4.32E-17		0.023161	0.0002	0.0003821	0.0000083	0.0001054	0.0000044	0.0015496	0.0000052	0.0002107	0.0000053						
Sample: CHX-7350; Material: Impact melt; D: 1.005215 ± 0.0005199; ngrains: 4																			
93302-08A	0.3	07/07/2018 14:16	5.61E-14	1.222623	0.0026062	0.4962515	0.0022	0.0054407	0.00057	0.0000614	0.0002401	0.0001462	0.0000561	96.68984	1.758347	2.376076	0.044573	56.21376	1.038293
93302-08B*	0.7	07/07/2018 15:11	1.90E-14	0.4170262	0.0028058	0.16826	0.0005601	0.0021649	0.0003	0.0000112	0.0002201	-0.0001019	0.0000521	107.4479	4.801354	2.656182	0.118678	62.72689	2.754581
93302-08C*	0.8	07/07/2018 16:45	1.24E-13	2.965271	0.0270006	1.09747	0.0039	0.0131948	0.0001501	0.0002163	0.0001801	0.000566	0.0000601	94.68967	1.443278	2.55205	0.03262	60.30831	0.758133
93302-08D*	0.9	07/07/2018 19:03	3.99E-14	0.9714275	0.004004	0.3526608	0.0013	0.0043584	0.0000771	0.0001074	0.0000305	0.0002587	0.0000382	92.94281	1.315292	2.545679	0.035933	60.16023	0.835211
93302-08E*	1.2	07/07/2018 19:59	2.68E-14	0.652417	0.0035046	0.2368975	0.001	0.0029793	0.00029	0.0000303	0.0002301	0.0000924	0.0000461	96.02312	2.946825	2.637806	0.081316	62.30033	1.887821
93302-08F*	1.3	07/07/2018 21:36	1.28E-13	3.19136	0.0035046	1.130064	0.0035	0.0136727	0.0001401	0.0002756	0.0002301	0.0007584	0.0000691	93.36752	0.770978	2.630254	0.023254	62.12498	0.539917
93302-08G*	1.8	07/07/2018 23:54	1.07E-13	2.672051	0.0033049	0.9430515	0.0033	0.0113568	0.0001301	0.0001851	0.0002201	0.000865	0.0000372	90.70484	0.633951	2.563652	0.020125	60.57795	0.467668
93302-08H*	2.3	08/07/2018 00:50	2.20E-14	0.5884311	0.0028058	0.1948746	0.0005201	0.0026085	0.00031	0.0001187	0.0002901	0.000222	0.0000551	89.83185	3.929931	2.706337	0.119263	63.89064	2.766361
93302-08I*	12	08/07/2018 02:25	1.05E-14	0.2875025	0.002307	0.0927909	0.0004001	0.0010301	0.0000522	-0.0000511	0.0002601	0.0000529	0.0000561	93.53718	7.714761	2.889982	0.240072	68.14539	5.55552
93302-08J*	35	08/07/2018 04:42	1.95E-14	0.5099063	0.002706	0.1726682	0.0005701	0.0022295	0.00027	-0.0000361	0.0002201	0.0001655	0.0000302	89.92714	3.034433	2.648552	0.089996	62.54978	2.089053
93302-08K*	50	08/07/2018 05:36	1.48E-14	0.3950849	0.0025065	0.1313258	0.0004401	0.0019032	0.00028	-0.0001496	0.0002101	0.0000601	0.0000461	93.41205	4.623895	2.801511	0.139239	66.0969	3.225782
Blanks (mean ± s.d.)		3.99E-17		0.02131	0.00018	0.0003532	0.0000085	0.0000995	0.0000042	0.0014919	0.0000055	0.0002	0.0000037						
Sample: CHX-7350; Material: Impact melt; D: 1.005215 ± 0.0005199; ngrains: 4																			
93302-09A	0.3	11/07/2018 02:07	4.52E-14	0.988764	0.0024067	0.4003382	0.0013	0.004749	0.0000961	0.0000413	0.0002701	0.0002651	0.0000322	92.2303	1.884913	2.272033	0.047385	53.78855	1.105273
93302-09B*	0.7	11/07/2018 03:02	1.70E-14	0.372235	0.0025065	0.1501093	0.0003201	0.0019396	0.00025	-0.00002	0.0002201	-0.0000297	0.0000481	102.0805	5.25074	2.524507	0.130098	59.66806	3.024747
93302-09C*	0.8	11/07/2018 04:36	1.19E-13	2.858577	0.0260006	1.049457	0.0034	0.0122793	0.0001301	0.0001891	0.0002701	0.0008355	0.0000342	91.64826	1.409071	2.490081	0.032135	58.86749	0.747456
93302-09D*	0.9	11/07/2018 06:53	3.03E-14	0.7346787	0.0032051	0.2678594	0.0012	0.0031207	0.00028	0.0000092	0.0002901	0.0000519	0.0000531	97.95935	3.209023	2.679842	0.088931	63.27596	2.063495
93302-09E*	1.2	11/07/2018 07:49	1.72E-14	0.4195684	0.0026062	0.1520402	0.0005001	0.0016365	0.0000631	-0.000022	0.0002401	0.0000595	0.0000471	95.45448	4.802522	2.627088	0.132945	62.05148	3.086874
93302-09F*	1.3	11/07/2018 09:33	1.59E-13	4.00581	0.0051032	1.406011	0.0039	0.0171546	0.0001601	0.0003359	0.0003001	0.0013073	0.0000681	90.73762	0.691378	2.578758	0.020947	60.92894	0.486671
93302-09G*	1.8	11/07/2018 10:29	7.54E-14	1.920577	0.0046035	0.666921	0.0026	0.0082558	0.0001101	0.0000323	0.0002201	0.0007668	0.0000631	88.17368	1.229944	2.532661	0.036505	59.85762	0.848641
93302-09H*	2.3	11/07/2018 12:05	6.18E-15	0.1719563	0.002307	0.0546683	0.0003301	0.0006296	0.0000542	-0.0000059	0.0002401	0.0000754	0.0000302	86.70617	9.849977	2.720121	0.3124	64.21034	7.245004
93302-09I*	12	11/07/2018 12:59	8.25E-15	0.2445696	0.002706	0.0729805	0.0002302	0.0009454	0.0000482	0.0000442	0.0000563	0.0002601	0.0000372	69.29242	4.927986	2.316864	0.163223	54.83394	3.805064
93302-09J	35	11/07/2018 15:18	3.17E-14	0.7790921	0.002307	0.2806268	0.0011	0.0033949	0.0000751	0.0000182	0.0002601	0.0008513	0.0000402	67.50065	2.511869	1.869164	0.070482	44.3669	1.652619
93302-09K	50	11/07/2018 16:17	1.73E-14	0.3531016	0.0026062	0.1530417	0.0004601	0.0020921	0.00029	0.0000434	0.0002401	0.000021	0.0000481	98.95023	5.788327	2.277254	0.133611	53.91032	3.116341
Blanks (mean ± s.d.)			3.99E-17	0.02131	0.00018	0.0003532	0.0000085	0.0000995	0.0000042	0.0014919	0.0000055	0.0002	0.0000037						

Run ID	Power (%)	Run Date & Time	^{39}Ar (moles)	^{40}Ar (V)	± σ	^{39}Ar (V)	± σ	^{38}Ar (V)	± σ	^{37}Ar (V)	± σ	^{36}Ar (V)	± σ	%^{40}Ar*	± σ	^{40}Ar*/ ^{39}Ar	± σ	Age (Ma)	± σ
**Sample: CHX-7560B; Material: Impact melt; D: 1.009502 ± 0.0006813; ngrains: 1**																			
93303-01A*	0.2	14/04/2018 23:25	4.72E-18	0.0004779	0.0010254	0.0000418	0.0000352	0.0000373	0.0000345	-0.0000136	0.0000102	-0.0000104	0.0000133	723.0151	1775.501	80.25844	117.3909	1312.557	1364.981
93303-01B*	0.3	15/04/2018 00:48	9.80E-14	2.756542	0.0070456	0.867575	0.0018	0.0107662	0.0001201	0.0011234	0.0000251	0.0010742	0.0000262	88.77929	0.450038	2.815581	0.014011	66.42284	0.324538
93303-01C*	0.4	15/04/2018 01:45	1.68E-14	0.4389137	0.0019698	0.1490745	0.0002502	0.0020793	0.0000593	0.0002635	0.0000122	0.0000636	0.0000173	96.29506	1.335984	2.830016	0.037538	66.75717	0.869323
93303-01D*	0.5	15/04/2018 03:55	7.14E-15	0.1860916	0.0028158	0.0631837	0.0001703	0.0008343	0.0000583	0.0000955	0.0000112	0.0000501	0.0000173	92.49261	3.4799	2.719118	0.093996	64.18707	2.179944
93303-01E*	0.6	15/04/2018 05:23	3.42E-15	0.0887851	0.0013591	0.030233	0.0001104	0.0004747	0.0000444	0.0000568	0.0000089	0.0000441	0.0000133	85.84372	4.940507	2.516403	0.139956	59.47962	3.254281
93303-01F*	0.7	15/04/2018 06:12	1.96E-15	0.0578313	0.0010419	0.0173869	0.0000795	0.0002628	0.0000326	0.0000211	0.0000092	0.0000456	0.0000153	76.84698	8.237142	2.551336	0.26988	60.29173	6.272496
93303-01G*	0.8	15/04/2018 08:20	1.34E-14	0.3934506	0.0019685	0.1187626	0.0002302	0.0016824	0.0000484	0.0002783	0.0000142	0.0002429	0.0000163	82.2992	1.402852	2.721758	0.044814	64.2483	1.039281
93303-01H*	0.9	15/04/2018 09:48	1.96E-15	0.0615693	0.0012549	0.0173182	0.0000875	0.0002341	0.0000316	0.0000484	0.0000112	0.0000264	0.0000143	88.01931	7.481358	3.123979	0.258315	73.55223	5.959826

93303-01I*	1	15/04/2018 10:37	4.20E-16	0.0129253	0.0009732	0.0037149	0.0000479	0.0000836	0.0000326	0.0000131	0.0000102	0.0000089	0.0000143	80.56695	34.54132	2.798629	1.181802	66.03013	27.38016
93303-01J*	5	15/04/2018 12:45	3.70E-15	0.1469514	0.001524	0.0327771	0.0001204	0.0004816	0.0000504	0.0001018	0.0000112	0.0001632	0.0000153	67.56143	3.364762	3.024128	0.147874	71.24699	3.416105
Blanks (mean ± s.d.)			1.31E-17	0.006487	0.0007954	0.0001157	0.0000093	0.000073	0.000006	0.0003494	0.0000022	0.0000692	0.000003						

Sample: CHX-7560B; Material: Impact melt; D: 1.005686 ± 0.0004619; ngrains: 1

93303-04A*	0.3	19/06/2018 11:29	1.32E-13	4.430545	0.0046035	1.166577	0.0026	0.0144577	0.0001601	0.0002513	0.0000283	0.0046454	0.0000673	68.89957	0.472252	2.610689	0.018956	61.67066	0.440225
93303-04B*	0.4	19/06/2018 12:26	1.71E-14	0.5186222	0.0033049	0.151175	0.0004101	0.0018104	0.0000641	0.0000413	0.00029	0.0003954	0.0000376	77.52557	3.131365	2.653446	0.107272	62.66338	2.489925
93303-04C*	0.5	19/06/2018 14:01	1.06E-14	0.309069	0.002706	0.0936763	0.0002802	0.0011411	0.0000462	-0.0000482	0.0003	0.0002342	0.0000356	76.7794	5.152561	2.526667	0.170237	59.71828	3.957871
93303-04D*	0.7	19/06/2018 16:17	8.17E-15	0.2509473	0.0021077	0.0722727	0.0002403	0.0009289	0.0000512	-0.000014	0.00028	0.0002332	0.0000366	72.03501	6.157724	2.495052	0.214659	58.9831	4.99266
93303-04E*	0.8	19/06/2018 17:11	1.25E-13	3.502418	0.0400004	1.109566	0.0027	0.0137518	0.0001501	0.0006216	0.00033	0.0018375	0.0000495	84.99934	1.616626	2.677053	0.041372	63.21124	0.960007
93303-04F*	0.9	19/06/2018 18:47	4.05E-14	1.159782	0.0030054	0.358819	0.0015	0.0046635	0.0001101	0.0001713	0.0000283	0.0006699	0.0000376	83.30812	1.031065	2.686613	0.034545	63.43307	0.801485
93303-04G*	1.6	19/06/2018 21:06	4.84E-14	1.67184	0.0051032	0.4285131	0.0015	0.005634	0.0000981	0.0002938	0.00033	0.001727	0.0000485	69.81625	1.206093	2.718008	0.047777	64.16133	1.108039
93303-04H*	12	19/06/2018 22:01	4.55E-14	1.65085	0.0031052	0.4026677	0.0015	0.0052623	0.0001201	0.000157	0.00023	0.002087	0.0000504	62.60715	1.079016	2.561001	0.04535	60.51634	1.053891
93303-04I*	16	19/06/2018 23:37	8.94E-15	0.3029008	0.0022074	0.0790936	0.0002502	0.0009685	0.0000482	-0.0001442	0.0000263	0.0003139	0.0000425	67.24485	4.300006	2.567548	0.163381	60.66849	3.796477
93303-04J*	20	20/06/2018 01:53	1.11E-14	0.3321049	0.0031052	0.0986328	0.0002602	0.0011673	0.0000601	-0.0000819	0.0000293	0.0002471	0.0000396	76.83845	3.769361	2.580265	0.124499	60.96395	2.892499
93303-04K*	24	20/06/2018 02:47	1.32E-14	0.4111962	0.0021077	0.1172505	0.0003502	0.0013046	0.0000581	-0.0000234	0.0000263	0.0003353	0.0000396	75.42283	2.957106	2.638541	0.102929	62.31738	2.389571
93303-04L*	28	20/06/2018 04:22	1.18E-14	0.3757627	0.0024067	0.1045302	0.0002702	0.0013236	0.0000631	-0.0000151	0.0000273	0.0003223	0.0000376	74.2299	3.108694	2.661873	0.110448	62.85897	2.563358
93303-04M*	32	20/06/2018 06:39	1.34E-14	0.4122601	0.0022074	0.118408	0.0003002	0.0013648	0.0000591	0.0000068	0.0000224	0.000333	0.0000386	75.93336	2.884833	2.637458	0.099484	62.29223	2.309638
93303-04N	36	20/06/2018 07:33	1.44E-14	0.4390091	0.0024067	0.1271055	0.0003302	0.0014973	0.0000561	0.0000222	0.0000283	0.0002125	0.0000356	85.73162	2.544318	2.954125	0.086567	69.62912	2.001615
93303-04O	40	20/06/2018 09:08	8.59E-15	0.3086701	0.0020081	0.0759883	0.0002303	0.0009477	0.0000522	-0.0000815	0.0000273	0.0002837	0.0000366	71.54171	3.650597	2.898132	0.147023	68.33397	3.401902
Blanks (mean ± s.d.)			5.26E-17	0.018123	0.00018	0.0004658	0.000011	0.0001119	0.0000041	0.0015559	0.0000041	0.0002196	0.0000067						

Sample: CHX-7560B; Material: Impact melt; D: 1.005215 ± 0.0005199; ngrains: 3

93303-06A*	0.3	05/07/2018 05:37	1.47E-13	3.905956	0.0046035	1.303516	0.0031	0.0154521	0.0001601	0.0002032	0.0002601	0.0008718	0.0000432	93.59793	0.503056	2.797699	0.01642	66.00859	0.380425
93303-06B*	0.4	05/07/2018 06:32	2.33E-14	0.5968399	0.0033559	0.2063753	0.0004308	0.002521	0.0000684	-0.0000425	0.0000563	0.0000751	0.0000351	95.87448	1.98439	2.765365	0.055597	65.25932	1.288627
93303-06C*	0.7	05/07/2018 08:06	1.31E-14	0.3380214	0.0026062	0.1161765	0.0003201	0.0012172	0.0000522	0.0000926	0.00032	0.0000826	0.0000342	94.10004	5.842403	2.731878	0.17136	64.48299	3.97349
93303-06D*	0.8	05/07/2018 10:23	6.46E-14	1.781262	0.0041039	0.5720133	0.0043	0.0069816	0.0001101	0.0001157	0.0002001	0.0008116	0.0000412	86.72592	0.953272	2.694058	0.03582	63.6058	0.830998
93303-06E*	0.9	05/07/2018 11:18	1.85E-14	0.4980103	0.0023795	0.1637662	0.0004209	0.0020727	0.0000694	0.0000001	0.0000602	0.0002131	0.0000399	87.22108	2.557241	2.645635	0.077091	62.48207	1.789561
93303-06F*	1.3	05/07/2018 12:51	1.05E-13	2.839596	0.0038043	0.9314115	0.0023	0.0118782	0.0001301	0.000365	0.0002801	0.0009621	0.0000442	90.54563	0.714166	2.753935	0.022846	64.99437	0.529609
93303-06G*	1.6	05/07/2018 15:09	4.09E-14	1.120473	0.0025065	0.3621476	0.0013	0.0043824	0.0000911	0.0001268	0.0002801	0.0004348	0.0000362	88.99407	1.644439	2.746889	0.052172	64.83102	1.209528
93303-06H*	20	05/07/2018 16:04	4.01E-14	1.277339	0.0051032	0.3550173	0.0013	0.0043246	0.0000851	0.0000604	0.0002901	0.000896	0.0000432	79.29529	1.635626	2.84608	0.059407	67.12917	1.375508
93303-06I	32	05/07/2018 17:40	1.28E-14	0.6207763	0.0024067	0.1131274	0.0003301	0.0014878	0.0000591	-0.0001187	0.0000523	0.0006348	0.0000442	68.47738	2.220589	3.746694	0.121419	87.8627	2.779288
93303-06J	40	05/07/2018 19:58	2.08E-14	0.9022957	0.0026062	0.1838776	0.0003901	0.0022159	0.0000741	-0.0000407	0.0000712	0.0007499	0.0000392	74.9468	1.408556	3.668281	0.068916	86.06693	1.579055
93303-06K	50	05/07/2018 20:52	2.49E-14	1.083002	0.002706	0.2206716	0.0005901	0.0028482	0.0000771	-0.0002019	0.0000533	0.0009609	0.0000521	72.53931	1.493402	3.549838	0.073392	83.35102	1.684149
Blanks (mean ± s.d.)			3.99E-17	0.02131	0.00018	0.0003532	0.0000085	0.0000995	0.0000042	0.0014919	0.0000055	0.0002	0.0000037						

Sample: CHX-7560B; Material: Impact melt; D: 1.00439 ± 0.0013455; ngrains: 3

93303-07A*	0.3	08/07/2018 10:37	1.08E-13	3.2001	0.004947	0.9553727	0.0022006	0.0114512	0.0001523	0.0001109	0.0002037	0.0019877	0.0000584	81.63671	0.678156	2.727691	0.024313	64.38589	0.563796
93303-07B*	0.4	08/07/2018 11:33	1.71E-14	0.4658521	0.0027088	0.151082	0.0003229	0.0018425	0.0000684	-0.000143	0.0002031	0.0001515	0.0000503	88.61005	4.088915	2.724007	0.125999	64.30045	2.921965
93303-07C*	0.7	08/07/2018 13:10	1.01E-14	0.2837682	0.0025782	0.0892148	0.0002726	0.000978	0.0000593	0	0.0002202	0.0001736	0.0000482	81.72111	6.728754	2.59269	0.214403	61.25261	4.980452
93303-07D*	0.8	08/07/2018 14:03	1.15E-13	3.117597	0.0044244	1.021555	0.0024003	0.0123642	0.0001616	0.0004524	0.0002427	0.001146	0.00005	89.81436	0.670839	2.73452	0.021943	64.54425	0.508798
93303-07E*	0.9	08/07/2018 15:39	3.23E-14	0.927738	0.002958	0.2856176	0.0012007	0.0035672	0.0000858	0.0001197	0.0002652	0.0005241	0.0000434	83.83608	2.14585	2.716886	0.071152	64.13532	1.650195
93303-07F*	1.3	08/07/2018 18:00	1.40E-13	3.971928	0.0048266	1.238316	0.0025003	0.0151275	0.0001338	0.0006947	0.0002435	0.0019477	0.000055	86.31422	0.558121	2.762226	0.019558	65.18655	0.453342
93303-07G*	1.6	08/07/2018 18:55	8.98E-15	0.3026717	0.0026843	0.0794383	0.0002736	0.0009662	0.0000546	0.0001498	0.0002388	0.0002595	0.0000441	77.11132	6.255831	2.933322	0.239933	69.14804	5.549215
93303-07H*	20	08/07/2018 20:30	1.35E-14	0.539687	0.0027028	0.1192	0.0003331	0.0015354	0.0000596	-0.0000789	0.0002483	0.0005602	0.0000484	68.20032	3.744325	3.079157	0.171108	72.5178	3.950055
93303-07I	32	08/07/2018 22:48	1.52E-14	0.6602103	0.0036249	0.1347791	0.0003131	0.0017477	0.000069	-0.0000444	0.0003757	0.0005316	0.0000508	75.58577	3.960825	3.692784	0.196978	86.62828	4.511926
93303-07J	40	08/07/2018 23:42	1.39E-14	0.5554949	0.0037162	0.1228256	0.0003341	0.0015428	0.0000716	-0.0000152	0.0003006	0.0003296	0.0000499	82.13083	4.118112	3.705009	0.187877	86.90828	4.302799
93303-07K	50	09/07/2018 01:17	1.59E-14	0.6225401	0.0037533	0.140733	0.0003738	0.0016046	0.0000763	0.0000992	0.0003634	0.0004435	0.0000501	79.60027	4.10874	3.513582	0.184135	82.51887	4.227347
Blanks (mean ± s.d.)			3.10E-17	0.0208191	0.0015898	0.0002742	0.0000534	0.0001003	0.0000253	0.0014262	0.000219	0.0001393	0.0000349						

Sample: CHX-7560B; Material: Impact melt; D: 1.004482 ± 0.0002617; ngrains: 6

93303-09A	0.3	18/07/2018 14:05	7.98E-14	2.07502	0.0034042	0.705957	0.0021	0.0084404	0.0001201	0.0000686	0.0000285	0.0007003	0.0000414	90.13919	0.64341	2.642608	0.020048	62.4118	0.465413
93303-09B	0.4	18/07/2018 15:01	1.64E-14	0.4146501	0.0027053	0.1451987	0.0003601	0.0016494	0.0000621	-0.0000913	0.0000493	0.0001295	0.0000296	89.1978	2.442498	2.539572	0.068017	60.01827	1.581068
93303-09C	0.7	18/07/2018 16:35	8.83E-15	0.222345	0.0020072	0.078132	0.0002702	0.0009893	0.0000581	0.0000038	0.0000542	0.0000507	0.0000316	93.3101	4.714861	2.648416	0.132383	62.54662	3.072991
93303-09D	0.8	18/07/2018 18:50	7.36E-14	1.858465	0.0032045	0.6509751	0.0016	0.0079601	0.0001201	0.0001786	0.0002501	0.000648	0.0000404	90.22988	1.139106	2.56955	0.033242	60.71499	0.772426
93303-09E	0.9	18/07/2018 19:45	1.94E-14	0.4752226	0.0037039	0.171442	0.0003701	0.0022115	0.0000352	0.0000058	0.0000265	0.0001083	0.0000326	93.28086	2.342382	2.578839	0.061862	60.93082	1.437276
93303-09F	1.3	18/07/2018 21:19	1.19E-13	3.139427	0.0042034	1.056696	0.0022	0.0131926	0.00017	0.0002499	0.0002501	0.0018005	0.0000454	83.40674	0.711439	2.471797	0.021756	58.44214	0.50616
93303-09G	1.6	18/07/2018 23:39	1.79E-15	0.0631563	0.001609	0.0158287	0.0001105	0.0001535	0.0000302	-0.0000251	0.0000453	0.0000315	0.0000316	82.43215	16.04832	3.277484	0.634726	77.09041	14.61574
93303-09H	20	19/07/2018 00:30	1.89E-14	0.5506379	0.0022066	0.1670394	0.0004701	0.0022003	0.0000561	0.0000525	0.0000255	0.0004726	0.0000365	75.01484	2.067272	2.46681	0.067677	58.32612	1.574645
93303-09I	32	19/07/2018 02:05	2.37E-14	0.6753767	0.0032045	0.2098787	0.0005701	0.0026721	0.0000671	-0.000069	0.0000642	0.0004843	0.0000404	77.89829	1.995894	2.499653	0.063478	59.09011	1.476315
93303-09J	40	19/07/2018 04:26	2.38E-14	0.6353173	0.0025058	0.2108901	0.0004601	0.0025354	0.0000641	-0.0001097	0.0000562	0.000282	0.0000335	85.57669	1.766945	2.57044	0.052558	60.73569	1.22125
93303-09K	50	19/07/2018 05:21	3.16E-14	0.8259529	0.002406	0.2792369	0.0011	0.0034258	0.0000781	0.0000086	0.0000493	0.0004208	0.0000345	84.85513	1.368102	2.503328	0.041031	59.17558	0.95421
Blanks (mean ± s.d.)			3.96E-17	0.020152	0.00017	0.00035	0.00001	0.0000956	0.0000038	0.0014712	0.0000051	0.0001654	0.000006						

Sample: CHX-7560B; Material: Impact melt; D: 1.001044 ± 0.0005687; ngrains: 3

93303-10A	0.3	08/08/2018 10:01	4.10E-14	1.035534	0.0038047	0.3625587	0.0016001	0.0044932	0.0001101	-0.0000353	0.0002101	0.0003531	0.0000294	89.47249	2.271842	2.54813	0.066218	60.2172	1.539078
93303-10B	0.4	08/08/2018 10:56	5.69E-15	0.1315834	0.0024075	0.050352	0.0002006	0.0005973	0.0000423	-0.0000037	0.0000259	0.000019	0.0000314	95.40367	7.817945	2.48596	0.199122	58.77162	4.631848
93303-10C	0.7	08/08/2018 12:25	3.19E-15	0.0716842	0.0017106	0.0282289	0.0001708	0.0003066	0.0000343	0.0000131	0.0000259	0.0000105	0.0000235	97.47073	10.97651	2.469171	0.27285	58.38104	6.348234
93303-10D	0.8	08/08/2018 14:39	3.05E-14	0.7737246	0.0031058	0.2696516	0.0009801	0.0033208	0.0000672	-0.0000532	0.0000239	0.0003409	0.0000284	86.14562	1.258778	2.464473	0.035897	58.27174	0.835236
93303-10E	0.9	08/08/2018 15:38	9.14E-15	0.2337986	0.0021086	0.0808873	0.0003104	0.000892	0.0000502	-0.00005	0.0000249	0.0001236	0.0000245	82.03946	3.510952	2.363362	0.099523	55.91758	2.318702
93303-10F	1.3	08/08/2018 17:08	2.11E-14	0.6267633	0.0032056	0.1866686	0.0003803	0.0022899	0.0000672	-0.0001025	0.0000249	0.0004909	0.0000344	74.94918	1.803651	2.508341	0.059367	59.29216	1.380547
93303-10G	1.6	08/08/2018 19:22	1.50E-15	0.0462283	0.0011163	0.0132652	0.0001211	0.0002019	0.0000314	-0.0000353	0.0000297	0.0000602	0.0000255	53.36337	17.92444	1.850163	0.621459	43.92133	14.57519
93303-10H	20	08/08/2018 20:11	2.88E-14	0.8414062	0.0032056	0.2552336	0.0012001	0.003338	0.0000672	-0.0000023	0.0002301	0.0008778	0.0000353	68.81899	3.089089	2.262402	0.103453	53.56387	2.413398
93303-10I	32	08/08/2018 21:48	1.24E-14	0.3841641	0.0031058	0.1095628	0.0003703	0.0015193	0.0000542	0.0000326	0.0000211	0.0002852	0.0000274	78.70237	2.434732	2.752703	0.082904	64.9658	1.921866
93303-10J	40	09/08/2018 00:00	1.79E-14	0.4912396	0.0031058	0.1586381	0.0003703	0.0020017	0.0000622	-0.0000069	0.0000201	0.0002041	0.0000274	87.45029	1.915068	2.700354	0.05709	63.75184	1.324326

93303-10K	50	09/08/2018 00:50	2.58E-14	0.6895244	0.003006	0.2285784	0.0007202	0.0028421	0.0000702	-0.0000197	0.0000278	0.000236	0.0000294	89.48699	1.461754	2.69171	0.043363	63.55133	1.006023
Blanks (mean ± s.d.)			3.77E-17	0.013534	0.00019	0.0003332	0.000016	0.0000652	0.0000048	0.0013613	0.0000066	0.0001077	0.0000049						
Sample: CHX-7560B; Material: Impact melt; D: 1.001044 ± 0.0005687; ngrains: 3																			
93303-11A*	0.3	09/08/2018 05:46	4.73E-14	1.221578	0.0042043	0.4181422	0.0014001	0.0049181	0.0000891	-0.0000881	0.0000268	0.0003006	0.0000284	91.90806	0.868029	2.677022	0.025329	63.21053	0.587729
93303-11B*	0.4	09/08/2018 06:40	2.02E-14	0.5188343	0.003006	0.1784098	0.0004103	0.0020391	0.0000732	-0.0001078	0.0000268	0.0000859	0.0000265	92.91842	1.7974	2.693139	0.050242	63.58448	1.165578
93303-11C*	0.7	09/08/2018 08:53	6.70E-15	0.1721321	0.0023078	0.0592608	0.0002405	0.0007269	0.0000542	-0.0000683	0.0000249	0.0000447	0.0000274	88.16942	5.300688	2.551198	0.150168	60.28852	3.490179
93303-11D*	0.8	09/08/2018 10:23	4.47E-14	1.214187	0.0044041	0.3954514	0.0015001	0.0051031	0.0000961	0.0000441	0.0000259	0.0004954	0.0000314	88.19213	0.93769	2.700562	0.029018	63.75668	0.673141
93303-11E*	0.9	09/08/2018 11:18	1.31E-14	0.3376004	0.0028064	0.116033	0.0004503	0.0013683	0.0000562	-0.0000361	0.0000268	0.0000312	0.0000245	96.13541	2.589113	2.788463	0.072468	65.79459	1.67916
93303-11F*	1.3	09/08/2018 13:30	6.30E-14	1.778464	0.0210009	0.5572064	0.0024001	0.0068603	0.0000951	0.0000878	0.0002101	0.0009602	0.0000542	84.38465	2.172167	2.686248	0.063158	63.4246	1.465369
93303-11G*	1.6	09/08/2018 15:03	2.69E-15	0.0895184	0.001512	0.0237961	0.0001509	0.0001867	0.0000393	-0.0000986	0.000023	0.0000874	0.0000265	59.4459	9.426027	2.221821	0.351162	52.61695	8.196358
93303-11H*	20	09/08/2018 15:56	6.10E-15	0.4541352	0.0024075	0.0539823	0.0002206	0.0012938	0.0000612	-0.0000823	0.0000268	0.0010662	0.0000393	28.04391	2.712889	2.349774	0.227905	55.60098	5.310679
93303-11I*	32	09/08/2018 18:10	2.92E-15	0.2896409	0.0027067	0.0258515	0.0002006	0.0009392	0.0000602	-0.0000884	0.0002201	0.0008567	0.0000373	8.555428	8.818224	0.953087	0.989215	22.75869	23.47335
93303-11J*	40	09/08/2018 19:42	2.70E-15	0.1949369	0.0016112	0.0238944	0.0001509	0.000469	0.0000413	0.0000173	0.0000278	0.0005053	0.0000304	23.53376	4.959957	1.916138	0.404616	45.46799	9.481405
93303-11K	50	09/08/2018 20:36	3.81E-15	0.161056	0.0016112	0.033684	0.0001708	0.000569	0.0000343	-0.0000219	0.0000249	0.0003398	0.0000245	35.59968	4.934028	1.69655	0.235191	40.31502	5.526978
Blanks (mean ± s.d.)			3.77E-17	0.013534	0.00019	0.0003332	0.000016	0.0000652	0.0000048	0.0013613	0.0000066	0.0001077	0.0000049						
Sample: CHX-7560B; Material: Impact melt; D: 1.002918 ± 0.0009517; ngrains: 3																			
93303-15A*	0.3	12/08/2018 08:07	3.57E-14	0.9375306	0.005456	0.3159036	0.00172	0.0036185	0.0001218	-0.0001368	0.0001043	0.0003951	0.000039	85.81657	1.901937	2.538623	0.05671	59.99622	1.31825
93303-15B*	0.4	12/08/2018 09:02	4.89E-15	0.1203544	0.0040759	0.0432531	0.0003601	0.0004766	0.0000808	-0.0000951	0.0000956	-0.000011	0.0000341	94.09906	13.00113	2.605473	0.353506	61.54953	8.210392
93303-15C*	0.7	12/08/2018 10:32	3.01E-15	0.0774725	0.0037869	0.0266722	0.0003127	0.0003311	0.0000729	0.0000522	0.0000916	0.0000294	0.0000331	96.02205	19.42524	2.786426	0.554341	65.74741	12.84505
93303-15D*	0.8	12/08/2018 12:48	3.86E-14	1.02499	0.0058946	0.341695	0.001521	0.0039724	0.000121	-0.000123	0.0000881	0.0002744	0.0000409	90.68599	1.709378	2.711737	0.050687	64.01589	1.175631
93303-15E*	0.9	12/08/2018 13:43	2.65E-14	0.6767765	0.0049636	0.2345149	0.0008861	0.0029327	0.00011	-0.000232	0.0000852	0.0001659	0.000038	88.92422	2.387174	2.556552	0.067363	60.41296	1.56552
93303-15F*	1.3	12/08/2018 15:20	4.33E-14	1.259202	0.0057016	0.3834331	0.0017181	0.0046099	0.0001299	-0.0000127	0.0000847	0.0007158	0.0000488	82.90971	1.495217	2.715078	0.049474	64.09338	1.147437
93303-15G*	1.6	12/08/2018 17:37	3.13E-15	0.0906066	0.0038894	0.027684	0.0003179	0.000408	0.0000724	-0.0000074	0.0000873	0.0000564	0.0000351	80.51691	16.64695	2.627186	0.537466	62.05374	12.47949
93303-15H	20	12/08/2018 18:29	4.08E-15	0.4858137	0.0048522	0.0361321	0.000324	0.0007291	0.0000738	-0.0000554	0.0000934	0.0015552	0.0000497	3.163219	3.855845	0.423568	0.519256	10.14972	12.40778
93303-15I	32	12/08/2018 20:03	3.38E-15	0.2185191	0.0042311	0.0298835	0.0003172	0.0006349	0.000068	-0.0000589	0.0000836	0.0005167	0.0000478	26.44182	8.035297	1.924695	0.587227	45.66849	13.75904
93303-15J	40	12/08/2018 22:20	2.88E-15	0.1661715	0.0041553	0.0254963	0.0003171	0.0004218	0.000073	-0.0000113	0.0000839	0.0004216	0.0000478	23.49176	10.5681	1.526304	0.688726	36.30982	16.22094
93303-15K	50	12/08/2018 23:13	3.12E-15	0.1306449	0.0042279	0.0276401	0.0003171	0.000568	0.0000745	-0.0000321	0.0000813	0.0002801	0.0000468	33.28541	13.18445	1.567227	0.621473	37.27337	14.62921
Blanks (mean ± s.d.)			6.46E-17	0.0205671	0.0028748	0.0005721	0.0002461	0.0001053	0.0000491	0.0020072	0.0000767	0.0001505	0.0000086						

Table B- 8: IrZ data

Run ID	Power (%)	Run Date & Time	^{39}Ar (moles)	^{40}Ar (V)	± σ	^{39}Ar (V)	± σ	^{38}Ar (V)	± σ	^{37}Ar (V)	± σ	^{36}Ar (V)	± σ	%^{40}Ar*	± σ	^{40}Ar*/ ^{39}Ar	± σ	Age (Ma)	± σ
Sample: IRz 14 D1 EK74 A; Material: Sanidine; D: 1.009998 ± 0; ngrains: 1																			
93298-01	12.5	07/04/2018 10:50	8.65E-14	2.159344	0.002003	0.7656906	0.0018	0.0096145	0.0000897	0.0000021	0.0000231	0.0000383	0.0000184	99.47245	0.287569	2.799821	0.010238	66.05776	0.237197
93298-02	12.5	07/04/2018 11:45	7.53E-14	1.889247	0.0022027	0.6662002	0.0023	0.0082309	0.0000946	-0.0000159	0.0000192	0.0000614	0.0000204	99.02252	0.363032	2.8027	0.013954	66.12444	0.323266
93298-03	12.5	07/04/2018 13:18	1.16E-13	2.926422	0.0026023	1.026822	0.0018	0.0125628	0.0001305	0.0000495	0.0000241	0.0001232	0.0000223	98.75745	0.261455	2.809138	0.008662	66.27359	0.200661
93298-04	12.5	07/04/2018 14:12	7.76E-14	1.935341	0.002003	0.686739	0.0018	0.0083824	0.0000897	0.0000159	0.0000202	0.0000758	0.0000194	98.83742	0.334289	2.780011	0.011711	65.59874	0.271388
93298-05	12.5	07/04/2018 16:29	1.11E-13	2.752508	0.0025024	0.9785653	0.0022	0.0123676	0.0001305	0.0000375	0.0000222	-0.0000046	0.0000322	100.0635	0.373767	2.809142	0.012102	66.2737	0.280338
93298-06	12.5	02/08/2018 06:26	1.34E-13	3.347686	0.0039506	1.184697	0.0022027	0.01394	0.0001503	0.0000309	0.0000449	0.0001307	0.000031	98.91297	0.345469	2.787408	0.010602	65.77016	0.245662
93298-07	12.5	02/08/2018 07:21	2.00E-13	5.028168	0.0050395	1.771085	0.0044014	0.0210796	0.0001702	0.0000462	0.0000469	0.0001752	0.0000357	99.0387	0.26934	2.80405	0.010055	66.15572	0.232928
93298-08	12.5	02/08/2018 13:15	1.48E-13	3.709959	0.0040493	1.313726	0.0042014	0.0153097	0.0001503	-0.0000085	0.0000508	0.0000732	0.0000532	99.38598	0.471712	2.798899	0.01582	66.0364	0.366518
93298-09	12.5	02/08/2018 14:09	1.17E-13	2.938433	0.0043459	1.031321	0.0023026	0.0122425	0.0001403	-0.0000065	0.0000313	0.0000924	0.0000348	99.03822	0.422311	2.813996	0.012944	66.38614	0.299823
93298-10	12.5	02/08/2018 15:43	1.40E-13	3.488333	0.0037533	1.240436	0.0037016	0.0146284	0.0001303	-0.0000541	0.0000577	-0.0000457	0.0000502	100.2484	0.480314	2.811289	0.015651	66.32342	0.362545
93298-11	12.5	02/08/2018 16:37	1.81E-13	4.505368	0.0044449	1.597444	0.0044014	0.0187194	0.0001702	0.0001346	0.0000469	-0.0000719	0.0000483	100.7418	0.362015	2.833629	0.012578	66.84083	0.291271
93298-12	12.5	02/08/2018 18:11	1.06E-13	2.630428	0.0037533	0.9383736	0.0033018	0.0108283	0.0001303	0.0000005	0.0000498	-0.0000757	0.0000483	100.8562	0.609084	2.819369	0.019347	66.51058	0.44812
93298-13	12.5	02/08/2018 19:05	1.75E-13	4.339505	0.0041481	1.54988	0.0041015	0.0182352	0.0001603	-0.0000412	0.0000449	0.0000197	0.0000444	99.77526	0.346961	2.785827	0.011909	65.73352	0.275949
93298-14	12.5	02/08/2018 22:01	1.02E-13	2.559682	0.0035562	0.9057079	0.0018034	0.0103753	0.0001603	-0.0000719	0.0000518	0.0000906	0.0000282	98.6835	0.424507	2.781085	0.01266	65.62362	0.293369
93298-15	12.5	02/08/2018 22:54	1.70E-13	4.217767	0.0044449	1.500016	0.0039016	0.0172581	0.0001902	0.0000574	0.0000488	0.0000971	0.0000512	99.43043	0.405703	2.788159	0.013194	65.78755	0.305729
Blanks (mean ± s.d.)			1.12E-16	0.020221	0.00063	0.000992	0.00011	0.0001115	0.000009	0.0013958	0.000009	0.0001232	0.000011						
Sample: IRz 17 D1 EK74 A; Material: Sanidine; D: 1.010594 ± 0; ngrains: 1																			
93301-01	12.5	07/04/2018 18:02	8.86E-14	2.219408	0.0036017	0.7836524	0.002	0.0091305	0.0001604	0.0000122	0.000098	0.0000405	0.0000302	99.45741	0.471068	2.811317	0.014542	66.32407	0.336847
93301-02	12.5	07/04/2018 18:55	6.04E-14	1.504062	0.0018034	0.5348994	0.0018	0.0062782	0.0000817	-0.0000058	0.000082	-0.0000432	0.0000292	100.8525	0.608186	2.830334	0.019397	66.76453	0.449217
93301-03	12.5	07/04/2018 21:08	8.71E-14	2.175018	0.0023026	0.7703657	0.0017	0.0093892	0.0000956	0.0000384	0.0001	0.0000536	0.0000273	99.27947	0.40621	2.797595	0.012752	66.00619	0.295448
93301-04	12.5	07/04/2018 22:40	1.10E-13	2.766987	0.0025024	0.9769861	0.0032	0.0118416	0.0001803	-0.0000243	0.0000152	0.0000291	0.0000342	99.67749	0.391678	2.817558	0.014378	66.46864	0.333028
93301-05	12.5	07/04/2018 23:35	6.46E-14	1.615311	0.0018034	0.5719529	0.0017	0.0069588	0.0000847	0.0000172	0.0000192	0.0000912	0.0000174	98.32311	0.359883	2.771468	0.012878	65.40076	0.298451
93301-06	12.5	03/08/2018 00:29	1.33E-13	3.282743	0.0039506	1.175797	0.0039016	0.014065	0.0001702	0.0000482	0.0002002	0.0000382	0.0000483	99.78324	0.726721	2.778265	0.022264	65.55829	0.515952
93301-07	12.5	03/08/2018 01:23	1.23E-13	3.091898	0.0036547	1.087215	0.0023026	0.0131948	0.0001303	-0.0000389	0.0002002	0.0000438	0.0000282	99.4616	0.66949	2.820669	0.019984	66.5407	0.462868
93301-08	12.5	03/08/2018 02:57	6.08E-14	1.533261	0.0030654	0.5377048	0.0017036	0.0064439	0.0000974	0	0.0000469	0.0001291	0.000031	97.48313	0.721823	2.77205	0.021722	65.41425	0.503425
93301-09	12.5	03/08/2018 03:51	1.48E-13	3.706337	0.0064309	1.312647	0.0035017	0.0159018	0.0001503	-0.0000081	0.000021	0.0000501	0.0000273	99.57517	0.334149	2.803781	0.011034	66.14949	0.255615
93301-10	12.5	03/08/2018 06:46	1.27E-13	3.198712	0.0038519	1.127483	0.0039016	0.0135746	0.0001802	0.0000232	0.0002502	0.0000773	0.0000273	99.34252	0.777	2.810644	0.024251	66.30849	0.561757
93301-11	12.5	03/08/2018 07:40	1.17E-13	2.930655	0.0041481	1.031751	0.0023026	0.011853	0.0001503	-0.000057	0.0000597	0.0000807	0.000031	98.99497	0.417645	2.804027	0.012851	66.1552	0.297701
93301-12	12.5	03/08/2018 09:14	9.93E-14	2.465552	0.0051388	0.8786705	0.003102	0.00984	0.0001303	-0.0000589	0.0002402	-0.0000673	0.0000502	100.5902	1.11775	2.814604	0.032772	66.40022	0.759116
93301-13	12.5	03/08/2018 10:08	1.26E-13	3.148322	0.0062319	1.117655	0.0040015	0.0136742	0.0001303	0.0000719	0.0000449	0.0000235	0.0000492	99.98499	0.560048	2.808833	0.01785	66.26653	0.413504
93301-14	12.5	03/08/2018 11:43	1.40E-13	3.48091	0.0059335	1.241394	0.0022027	0.0146871	0.0001902	-0.0000518	0.0002002	0.0000956	0.0000301	99.04048	0.632748	2.769351	0.01804	65.3517	0.418115
93301-15	12.5	03/08/2018 12:37	1.01E-13	2.515778	0.0038519	0.8935006	0.0023026	0.0103451	0.0001702	0.0000648	0.0000557	0.000145	0.0000348	98.50957	0.507483	2.766164	0.015402	65.27784	0.356981
Blanks (mean ± s.d.)			1.12E-16	0.020221	0.00063	0.000992	0.00011	0.0001115	0.000009	0.0013958	0.000009	0.0001232	0.000011						
Sample: IRz 20 D1 EK74 A; Material: Sanidine; D: 1.011391 ± 0; ngrains: 1																			
93304-01	12.5	08/04/2018 01:49	1.18E-13	2.948028	0.0038016	1.045759	0.0022	0.0127831	0.0001205	0.0000018	0.000077	0.000094	0.0000194	99.04803	0.271381	2.786781	0.009069	65.75562	0.210135
93304-02	12.5	08/04/2018 03:22	1.28E-13	3.19578	0.0026023	1.134	0.0024	0.0139824	0.0001105	0.0000496	0.0000212	0.0000284	0.0000521	99.74864	0.501279	2.805621	0.015203	66.19212	0.352183
93304-03	12.5	08/04/2018 04:16	8.59E-14	2.158216	0.0026023	0.7600044	0.002	0.0091576	0.0001504	0.0000488	0.0000231	0.0000288	0.0000412	99.62089	0.595763	2.823507	0.018224	66.60643	0.422071
93304-04	12.5	08/04/2018 06:31	5.94E-14	1.537093	0.0019032	0.5258231	0.0016	0.006601	0.0000768	-0.0000003	0.0000152	0.0002453	0.0000174	95.23518	0.380861	2.77855	0.013683	65.56489	0.317085
93304-05	12.5	08/04/2018 08:04	9.44E-14	2.355316	0.0026023	0.8350997	0.0021	0.0105674	0.0000966	0.0000411	0.0000212	0.0000203	0.0000292	99.75992	0.403776	2.808179	0.013139	66.25138	0.304368
93304-06	12.5	14/08/2018 13:00	1.53E-13	4.063501	0.0065011	1.358083	0.0032099	0.015644	0.0002159	-0.0000609	0.0001079	0.0009421	0.0000527	92.89973	0.538969	2.771653	0.016934	65.40504	0.392475
93304-07	12.5	14/08/2018 13:56	1.76E-13	4.447452	0.0143083	1.557178	0.004607	0.0187027	0.0002063	0.0001366	0.0003881	0.000138	0.000036	99.42132	1.126533	2.831752	0.032558	66.79737	0.753982
93304-08	12.5	14/08/2018 16:10	2.16E-13	6.908972	0.0114039	1.910044	0.0058057	0.0236252	0.0001968	-0.0000384	0.0003009	0.0050018	0.0000765	78.31537	0.637251	2.824855	0.024501	66.63764	0.567448
93304-09	12.5	14/08/2018 17:47	1.28E-13	3.22165	0.0055239	1.129858	0.0029118	0.013267	0.0001683	0.0000007	0.0003109	0.0001686	0.0000322	98.43563	1.173502	2.798794	0.034544	66.03397	0.800327
93304-10	12.5	14/08/2018 18:40	1.80E-13	4.527582	0.006396	1.589886	0.00351	0.0197998	0.0002068	0.00015	0.000189	0.0017889	0.0000656	88.57885	0.672862	2.515563	0.019806	59.4601	0.460526
93304-11	12.5	14/08/2018 20:57	1.08E-13	3.089494	0.0048065	0.9536489	0.0023002	0.0103107	0.0006901	0.0001876	0.0003303	0.0015125	0.0000865	86.06634	1.498105	2.780961	0.049471	65.62075	1.146403
93304-12	12.5	14/08/2018 22:31	1.29E-13	3.313299	0.0045069	1.145471	0.0046001	0.0141968	0.0002302	0.0000283	0.0003402	0.0002715	0.00004	97.65107	1.24884	2.816627	0.038341	66.44707	0.888092
93304-13	12.5	14/08/2018 23:25	2.05E-13	5.09806	0.0089035	1.811442	0.0055001	0.0223035	0.0002602	0.0001137	0.0003502	0.000002	0.0000736	100.2445	0.933934	2.813392	0.027592	66.37213	0.639144
93304-14	12.5	15/08/2018 01:43	2.34E-13	5.852567	0.0053059	2.074631	0.0054001	0.0248229	0.0002402	0.0000283	0.0003203	0.000219	0.00004	98.93577	0.67589	2.783086	0.020593	65.66999	0.477205
93304-15	12.5	15/08/2018 03:20	1.46E-13	3.643848	0.0077041	1.292186	0.0043001	0.0156719	0.0002302	0.0000283	0.0002703	0.000002	0.0000617	100.0721	1.03955	2.813968	0.030616	66.38549	0.709184
Blanks (mean ± s.d.)			4.78E-17	0.018084	0.00025	0.0004233	0.000031	0.0000991	0.00001	0.0019415	0.000013	0.0001363	0.0000091						

Supplement C –

Argon diffusion kinetics and thermal history constraints

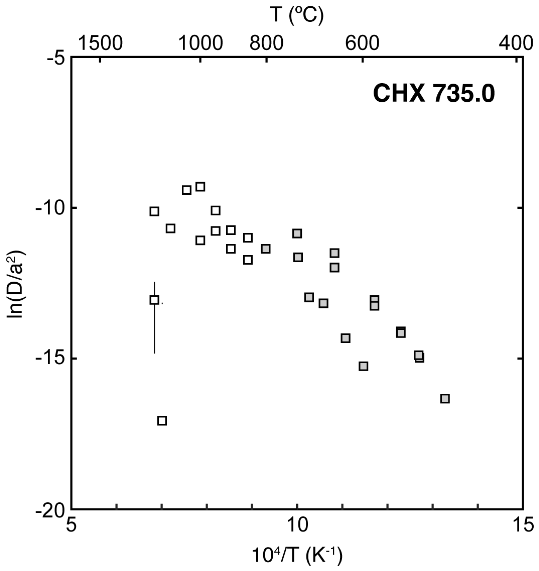

Figure C- 1: Arrhenius plot for diffusion of neutron-induced ^{39}Ar from sample CHX 735.0. *D/a^2* values are normalized to s^{-1}. Uncertainties in *$ln(D/a^2)$,* estimated using a Monte Carlo approach[8], are shown; in most cases the uncertainty is smaller than the symbol plotted. Diffusivities for heating steps of 800 ºC and lower are filled; we use these steps to fit multi-diffusion domain (MDD) models.

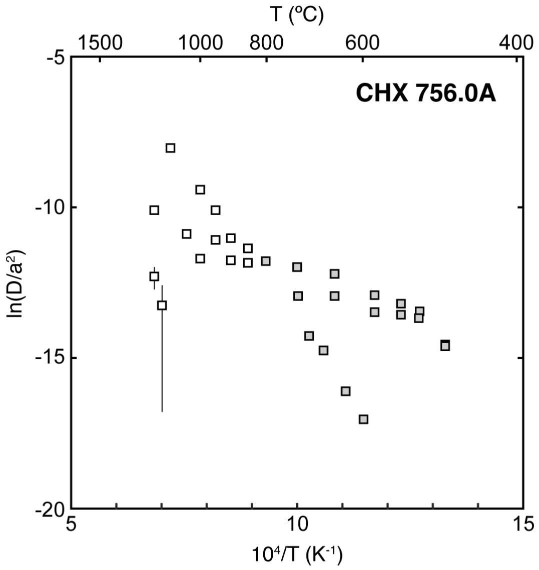

Figure C- 2: Arrhenius plot for diffusion of neutron-induced ^{39}Ar from sample CHX 756.0A. *D/a^2* values are normalized to s^{-1}. Uncertainties in *$ln(D/a^2)$,* estimated using a Monte Carlo approach[8], are shown; in most cases the uncertainty is smaller than the symbol plotted. Diffusivities for heating steps of 800 ºC and lower are filled; we use these steps to fit multi-diffusion domain (MDD) models.

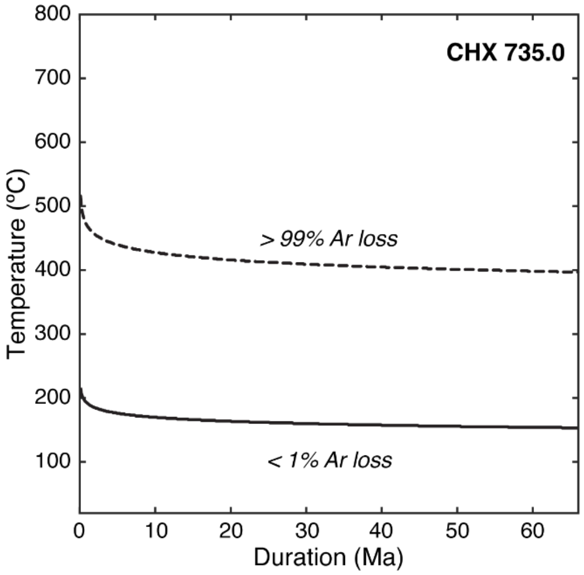

Figure C- 3: Isothermal models for argon diffusive loss, sample CHX 735.0. Curves are shown for complete argon retention (solid) and complete argon loss (dashed) for the best fit multi-diffusion domain (MDD) model that assumes argon is hosted in 2 phases.

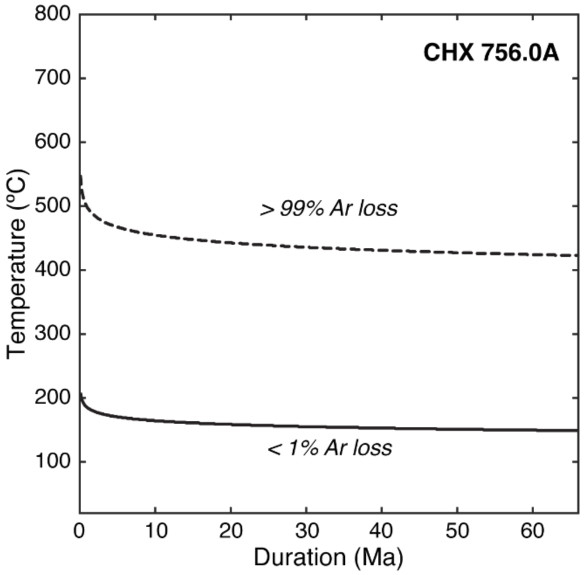

Figure C- 4:. Isothermal models for argon diffusive loss, sample CHX 756.0A. Curves are shown for complete argon retention (solid) and complete argon loss (dashed) for the best fit multi-diffusion domain (MDD) model that assumes argon is hosted in 2 phases.

Table C- 1: ^{39}Ar measurements for the diffusion experiment on CHX-735.0.

Step	Temperature (ºC)	Duration (s)	^{39}Ar (V)	± 1σ (V)	D/a^2 (s^{-1})	± 1σ (s^{-1})
1	480	900	0.035866	0.000080	8.129E-08	3.596E-10
2	480	1800	0.025905	0.000100	7.991E-08	8.044E-10
3	514	900	0.031956	0.000110	3.140E-07	6.788E-10
4	515	1800	0.042889	0.000120	3.373E-07	2.382E-09
5	540	900	0.035015	0.000100	7.573E-07	1.281E-09
6	540	1800	0.050338	0.000120	7.175E-07	4.081E-09
7	580	900	0.056570	0.000120	2.137E-06	2.662E-09
8	580	1800	0.071104	0.000120	1.776E-06	7.258E-09
9	651	600	0.098009	0.000084	1.005E-05	4.529E-09
10	650	600	0.047726	0.000120	6.201E-06	1.661E-08
11	726	600	0.114756	0.000082	1.912E-05	2.359E-08
12	725	600	0.041467	0.000130	8.631E-06	2.760E-08
13	671	1800	0.024901	0.000095	1.899E-06	2.189E-08
14	630	3600	0.014688	0.000079	5.924E-07	6.418E-09
15	599	3600	0.005640	0.000039	2.342E-07	1.628E-09
16	701	3600	0.050851	0.000130	2.292E-06	5.998E-09
17	801	900	0.054918	0.000130	1.156E-05	7.109E-09
18	849	900	0.066834	0.000100	1.696E-05	2.992E-08
19	849	1800	0.052639	0.000140	8.120E-06	4.413E-08
20	900	900	0.057722	0.000140	2.179E-05	2.720E-08
21	900	1800	0.048868	0.000160	1.154E-05	7.447E-08

22	948	900	0.065008	0.000100	4.169E-05	4.166E-08
23	946	1800	0.045262	0.000180	2.105E-05	1.553E-07
24	1000	900	0.055235	0.000110	9.077E-05	1.254E-07
25	1000	1800	0.010689	0.000083	1.544E-05	2.353E-07
26	1051	3600	0.032027	0.000110	8.190E-05	2.209E-06
27	*1118	900	0.000339	0.000022	2.286E-05	4.212E-07
28	*1153	900	^–	^–	–	–
29	*1189	900	0.000028	0.000023	2.111E-06	1.727E-06
30	*1189	1800	0.000761	0.000042	4.068E-05	5.129E-06
31	*1189	1800	0.000718	0.000049	–	–

*Temperature not calibrated.

^Measured ^{39}Ar was below detection limits.

Table C- 2: ^{39}Ar measurements for the diffusion experiment on CHX-756.0A.

Step	Temperature (ºC)	Duration (s)	^{39}Ar (V)	± 1σ (V)	D/a^2 (s^{-1})	± 1σ (s^{-1})
1	480	900	0.060279	0.000089	4.752E-07	1.375E-09
2	480	1800	0.039638	0.000090	4.491E-07	2.692E-09
3	514	900	0.040346	0.000099	1.428E-06	2.132E-09
4	515	1800	0.045673	0.000092	1.150E-06	5.629E-09
5	540	900	0.028834	0.000100	1.860E-06	3.450E-09
6	540	1800	0.033510	0.000083	1.297E-06	7.112E-09
7	580	900	0.027100	0.000079	2.464E-06	3.822E-09
8	580	1800	0.026602	0.000071	1.382E-06	7.949E-09
9	651	600	0.028121	0.000094	4.983E-06	5.768E-09
10	650	600	0.012473	0.000052	2.422E-06	1.033E-08
11	726	600	0.029556	0.000079	6.295E-06	1.753E-08
12	725	600	0.010407	0.000062	2.414E-06	1.457E-08
13	671	1800	0.004872	0.000043	3.891E-07	1.041E-08
14	630	3600	0.002516	0.000044	1.020E-07	3.572E-09
15	599	3600	0.000981	0.000039	4.008E-08	1.589E-09
16	701	3600	0.014869	0.000062	6.281E-07	2.650E-09
17	801	900	0.039675	0.000082	7.514E-06	4.124E-09
18	849	900	0.051402	0.000110	1.175E-05	2.576E-08
19	849	1800	0.051402	0.000110	7.274E-06	3.146E-08
20	900	900	0.046485	0.000094	1.625E-05	1.681E-08
21	900	1800	0.036854	0.000096	7.809E-06	4.032E-08

22	948	900	0.073036	0.000090	4.168E-05	2.986E-08
23	946	1800	0.037346	0.000093	1.536E-05	7.490E-08
24	1000	900	0.061650	0.000079	8.214E-05	7.334E-08
25	1000	1800	0.007878	0.000057	8.312E-06	1.172E-07
26	1051	3600	0.024033	0.000070	1.870E-05	1.226E-07
27	*1153	900	0.000022	0.000021	3.234E-04	9.544E-07
28	*1189	900	0.000057	0.000020	1.732E-06	1.653E-06
29	*1189	1800	0.000712	0.000027	4.616E-06	1.607E-06
30	*1189	1800	0.000650	0.000033	4.164E-05	3.733E-06

*Temperature not calibrated.

Table C- 3: Diffusion parameters for the best fit MDD models.

# of phases	E_a (kJ/mol)	$ln(D_0/a^2)$	f
		CHX 735.0	
2	173.6	6.94, 11.18, 12.86, 8.13	0.34, 0.10, 0.25, 0.18
	243.8	23.72, 26.14	0.09, 0.04
		CHX 756.0A	
2	240.6	26.25, 12.48, 23.98, 16.12	0.18, 0.43, 0.07, 0.17
	165.8	12.20, 19.79, 19.99	0.13, 0.01, 0.01

Supplement D –
Numerical Simulations

Methods

For this study, we used the updated version of the U.S. Geological Survey (USGS) HYDROTHERM program (Version 3.2.0; HT3[9]; firstly developed by Hayba & Ingebritsen[10]) that provides the coupled equations and solutions for the mass and energy conservation in putative thermodynamic systems. A three-dimensional iterative technique based on the method of general conjugate residuals (GMRES) is a new functional characteristic of the HYDROTHERM version 3 code and this new solver is over 10 times faster for solving this problem than the original HYDROTHERM. During the simulations via the older solver, the nonlinear behaviour of the equations led to non-convergent conditions that required several adjustments for the simulations to continue through the entire time specified for the problem. Moreover, impact-induced hydrothermal systems that are mainly represented by the upper heat limit conditions may have been more simplistically parametrized, and thus the complexity and resolution of the thermodynamics that govern impact-induced hydrothermal systems had been previously decreased. Further details on the equations of state used in this work are provided in the HT3 user's manual (USGS[9]). This work modelled the post-impact thermodynamic evolution in the Chicxulub crater as governed by permeability, porosity and fracturing of impactites.

Algorithms addressing hydrothermal convection solutions treat rocks as continuous media. In these sets of simulations, we have included local changes in permeability and a higher level of discretization (resolution) to increase the complexity of the simulated domains. The coupled heat and fluid transport differential equations were independently solved by the Galerkin finite element method and the Picard iterative technique through Fortran coding and were finally used as input parameters in the HT3 (version 3.2.0) code. Several feasibility tests were then performed to check the validity of the modified version. For this reason, the Abramov and Kring (2007)[11] Chicxulub model was also reproduced to validate the code. Multiple sets of simulations were produced for the specified setting in the presence and absence of fractures to assess the efficiency of the modified (version 3.2.0) algorithm versus the original numerical code. The validation results proved that fractures can be successfully described within a simulative geological setting. Assuming that the rocks of the peak ring and central inner basin of a Chicxulub-size impact crater are far more fractured than those of the outer rim regions,

we investigated the effects of the localized higher permeability on the hydrothermal fluid flow.

The simulations showed that fractures initiate and maintain hydrothermal fluid circulation in media which would otherwise behave passively (Figure D-1). Fracture location seems to govern both the convection pattern and vigour of a closed thermodynamic system. In our fluid mechanical models, discrete fractures (described solely by permeability and porosity functions) evidently change an established convection pattern and multiple fractured porous media are comparable with anisotropic media in these numerical solutions. This is also consistent with observations in geological settings of impact craters, where rock units of the peak ring and the central basin are significantly fractured. We found that the lifetime of hydrothermal flow is affected after minor changes to the hydraulic conductivity within the fractures and the spatio-temporal distribution and geometry of fractures and convective cells. Figure D-1 illustrates differences in the post-impact hydraulic response in the absence [A] and presence [B] of fractures (size x,z of fractures in [B]: 1 mm – 2 cm). We additionally performed several other simulations to test how the width and distribution of discontinuities affect the hydrothermal cooling rates. Preliminary results suggest that fractures of a width greater than 4 cm result in a far more rapid cooling of the hydrothermal system and shorten the duration of fluid flow. Moreover, the geometrical characteristics of fractures play an important role in the convection pattern of the setting but more experiments are needed to determine an empirical relationship for the direction of flow and the geometry of fractures[12,13]. The results show that if fractures of a 1 mm – 2 cm width range are present in the impactites, then the duration of hydrothermal flow is increased by a factor of 2 and water flux is 3 orders of magnitude greater at 2 Myr after the impact (Figure D-1). For this reason, the effective permeability of lithological units is by 0.7 – 1.5 orders of magnitude higher than that of the lithologies modelled by Abramov and Kring (2007)[11]. In conclusion, fracturing seems to significantly affect the lifetimes of impact-induced hydrothermal systems, either by increasing the duration of fluid flow as presented in this study, or by resulting in rapid cooling, if the density and width of fractures overcome a yet unknown critical threshold. Future research should focus on establishing an empirical relationship that would adequately describe these phenomena.

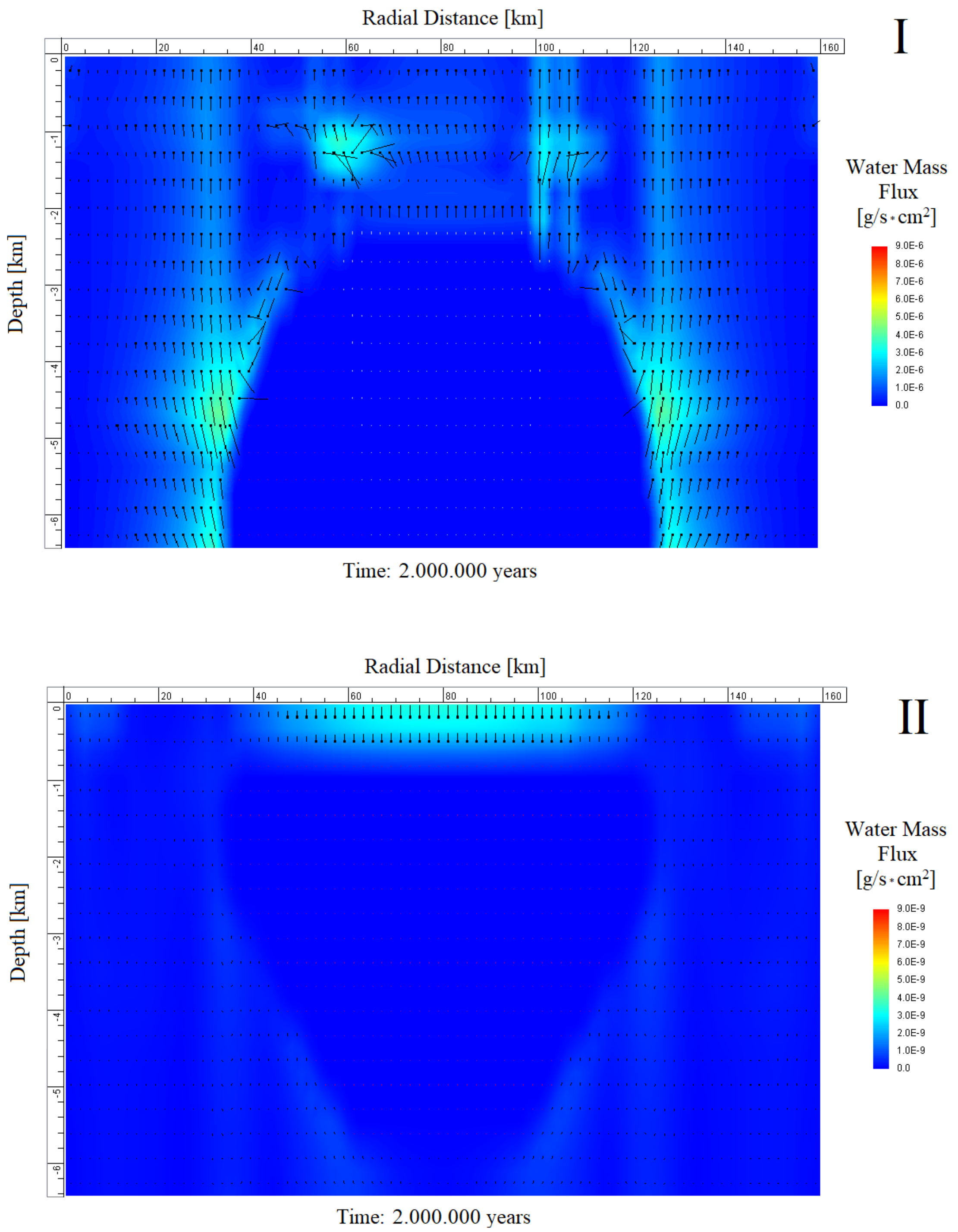

Figure D- 1: [I] Post-impact water flow in a fractured porous impactite setting of a putative Chicxulub-like crater including fractures. [II]: Water flow in the same setting without fractures. At 2 Myr after the impact, the Water Mass Flux max. in [I] = 3.39E-6 g/s·cm^2; the Water Mass Flux max. in [II] = 3.06E-9 g/s·cm^2. Furthermore, the hydrothermal convection pattern is noticeably different. The simulations via the HT3 code were performed to understand how the impact-induced hydrothermal system at Chicxulub evolved over time, with particular attention to the thermodynamic and fluid flow conditions, mechanics and lifetime of the system.

The results presented are highly dependent on the host rock permeability which is the most significant variable affecting fluid circulation and lifetimes of hydrothermal systems[11,14]. Therefore, a goal of Expedition 364 was to measure porosity and permeability in sampled rock[14]. In this research work, permeability was adjusted as accurately as possible for each rock unit through benchmark simulations to better understand its effects on the lifetime of the hydrothermal system, and more significantly, to produce simulations that are more consistent with current studies on impact-induced thermodynamics[15,16]. Porosity and permeability have been measured on the M0077A samples[14]. In general, all peak ring lithologic groups exhibit elevated porosities. Granitoid basement rocks have porosities ranging between 7-13%. Melt-bearing impact breccias (suevites) have porosities ranging between 8-35%. Impact melt rocks have porosities ranging between 9-22%. There is not a consistent relationship between porosity and permeability within M0077A[14]. Fractured granitoids and clast-supported suevites had relatively high permabilities at 10 MPa pressure (~10^{-16} to ~10^{-15} m^2). In contrast, melt-rich lithologies had far lower permeabilities (~10^{-19} to ~10^{-18} m^2). We note that these measured porosities and permeabilities correspond to the “final state” of the drill core materials and may not reflect the initial permeabilities or interim permeabilities as the hydrothermal system evolved. Porosities and permeabilities within the crater immediately after impact may also be estimated from impact hydrocode simulations[17]. Permeabilites from impact simulations (up to 10^{-14} m^2) tend to be larger than what is typically measured from natural core samples because intact cores do not exhibit the large-scale fragmentation and faulting that naturally occurs within craters and that can drive more efficient fluid flow. For our simulations, we utilize permeability values from analagous materials as discussed below. Assuming that the inner basin of the crater was far more fractured than the outer rim region due to the impact, we expect the permeability and porosity of the rocks therein to be appreciably increased. Most of the rock properties of the peak-ring and inner crater basin are determined according to Christeson et al. (2018)[18]. Some of the rocks have similarities with those of the Yaxcopoil-1 drilled core and hence, previously published data[19–26] describing the thermodynamic properties of outer rim rock units at Chicxulub were also used as input parameters for the HT3 numerical simulations. The permeability values of the impact-breccias of the setting that we simulated are taken from [23,24,26–28]. Furthermore, knowing that impact-induced hydrothermal systems are similar to volcanic settings, in this work, physical properties of the fractured rocks and their hydraulic conductivity, reflect those of highly pressurised volcanic settings[26]. Finally, we adopted the thermodynamic models for melt rocks (constructed by [27]) to estimate the post-impact physical characteristics of the melt and to assess the thermodynamic response of the lower crust and asthenospheric mantle. The petrophysical properties of the impact-induced hydrothermal setting are shown in Table D-1

and are simplified for computational efficiency, according to the methodology followed by Abramov & Kring (2007)[11].

In the simulations, the melt in the peak ring is only a few dozens of meters thick and it has been illustrated with a maximum thickness of 1.5 km beneath the centre of the crater. The thickness of the central melt based on petrophysical properties of the central melt sheet and its surrounding units have been adjusted to account for a radial thickness of 1.5 km (Figure D-2.1). Note, the melt sheet thickness has been estimated to average 2.5 km thick based on full waveform data[29], and the width melt sheet depicted here is also wider than what is inferred from seismic reflection and refaction data[30] and well as full waveform inversions[29] but does not include bodies of melt beneath the annular trough. Thus on balance, the duration of the hydrothermal system from this simulation can be considered conservative. The permeability and porosity values of each rock unit were accordingly determined and simply imported as parameters into the HT3 interface. Initial temperature conditions are converted to enthalpy automatically at the starting step of a HT3 simulation. The west and east sides of the grid were specified as insulated (no heat flux) and the base of the grid was assigned a basal heat flux of 45 mW/m^2 after recent observations (including those of the IODP-ICDP 364 expedition) on the geothermal gradient at Chicxulub[31]. Much uncertainty still exists as to the initial temperature conditions at the base of the crater after the impact. One possibility is that a superheated impact melt (up to 2000°C) might have been present. The most reliable scenario, after observations on impact glasses (ejecta)[32–34] suggests that impact-induced melt was partially ejected from the crater. Since the upper temperature limit for HT3 is 1200°C, this value was chosen as an input temperature, given all of the other uncertainties. 1200°C is a reasonable approximation because it is estimated that large melt sheets equilibrated to ~1200°C after 2 minutes[35]. Most of the heat transfer into the crater over time originates from the heated melt sheet and the uplifted lower crustal rocks and granitoids. Our initial temperatures used for uplifted lower crust are likely also conservative when compared to impact simulations, and thus additional heat from this source as well as latent heat of crystallization within the melt sheet likely would have extended the duration of the hydrothermal system beyond our results. In our modelling results (see the results below for temperature and water mass flux distributions from t_0 to t_{final} = 10 Myr), heat transfer at 0.5-1.0 Myr after the crater's formation is mainly governed by a network of vertical heat and degassing pipes that form at greater depths and which evolve as ubiquitous and strong conduits of hydrothermal fluid circulation and heat transfer at depths between 2.5 – 0.5 km beneath the seafloor. Our hydrothermal models are consistent with recent geochemical findings that have thoroughly described and concluded on the impact-induced formation and evolution of such degassing pipes in the Ries impact structure[36]. These hydrothermal pipes

can be easily distinguished as vertical in orientation convection cells (especially at depths between 0 – 2500 mbsf) in our post-impact water mass flux evolution results at $t_{steps} > 0.5$ Myr, and indicate that fluids should have been circulating and escaping from the deeper and hot subsurface realm of the crater to the seafloor. Therefore, the exact temperature of the central melt sheet may not be a critical parameter for understanding the evolution of the Chicxulub hydrothermal system, since highly increased basal heat flux from the lower crust – upper mantle and the impact-induced emplacement of the uplifted granitic domain seem to be the main drives for the heat transfer patterns observed in the numerical models of this research.

The initial temperature distribution was assigned to reflect the impact heating as predicted by earlier studies using the HYDROTHERM code[11,37,38]. HT3 allows to draw temperature and pressure contours within the domain that function as an input for the initial thermodynamic conditions of the setting. Pressure distributions are assigned to the hydrostatic 1.01 bar at the upper boundary of the domain, and to 1000 bars at the lower boundary which amounts to the hydrostatic pressure down to a depth of $D \geq 7$ km for active hydrothermal systems[39]. Figure D-2.1 - 2.2 illustrate the initial thermodynamic and boundary conditions of the simulated grid.

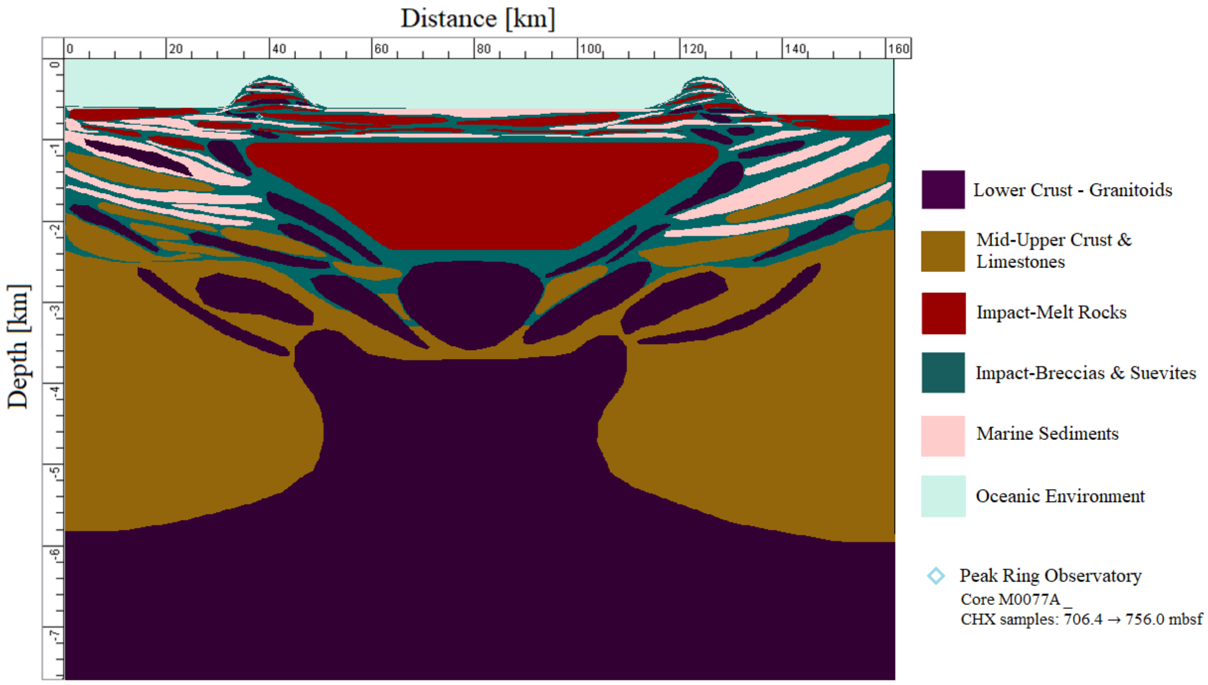

Figure D-2. 1: HYDROTHERM 2D model for the initial petrophysical properties at Chicxulub (not meant to reflect actual structures at depth).

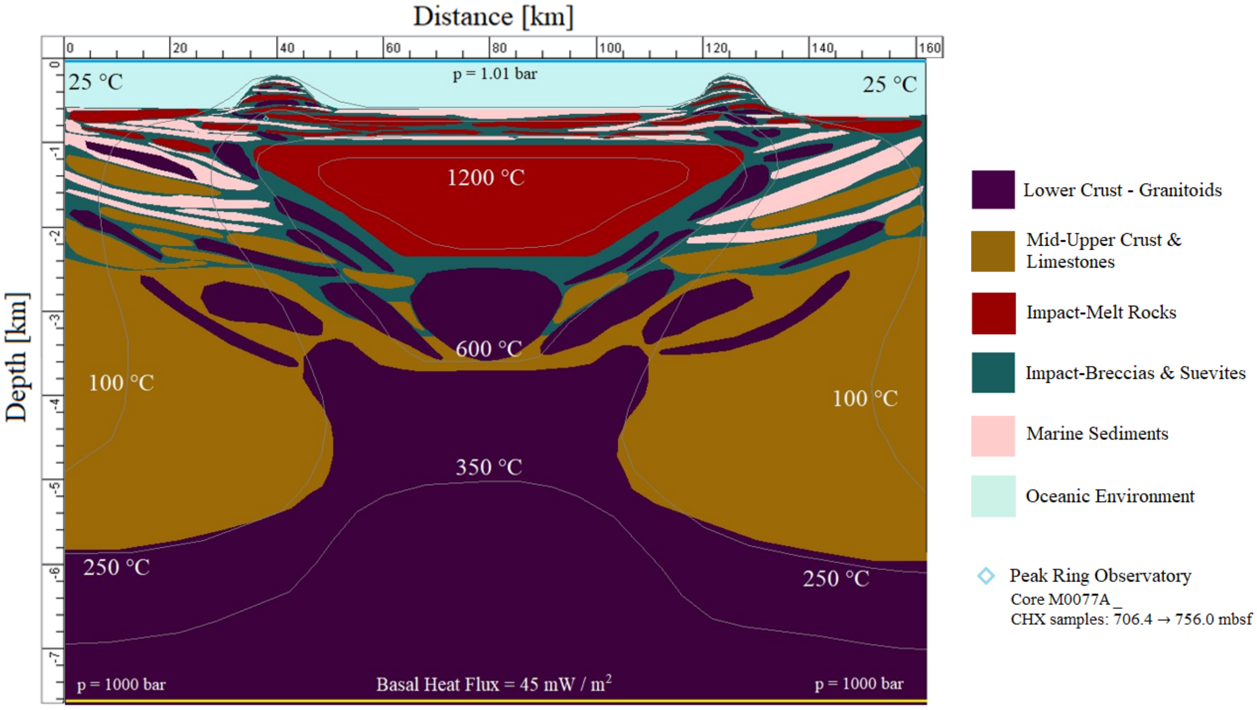

Figure D-2. 2: Chicxulub starting physical conditions assumed for the HT3 numerical simulations.

<u>Petrophysical Properties</u>	Lower Crust - Granitoids	Mid - Upper Crust	Melt Rocks	Breccias	Sediments
**Permeability (k) {m^2}**	k_z = f (d,T), k_x = f (d,T)	k_z = f (d,T), k_x = f (d,T)	k_z = f (d,T), k_x = f (d,T)	k_z = f (d,T), k_x = f (d,T)	k_z = f (d,T), k_x = f (d,T)
**Porosity (φ)** ·	φ = f (d,T)	φ = f (d,T)	φ = f (d,T)	φ = f (d,T)	φ = f (d,T)
**Thermal Conductivity {W/m K}**	2.5	2.5	2.5	2.5	2.5
**Specific Heat Capacity {J/kg K}**	1000	1000	1000	1000	700
**Density {kg/m^3}**	2700	2500	2500	2500	2300
**Surface Permeability {m^2}**	4.00E-18	1.00E-17	4.00E-17	5.00E-15	5.00E-14
**Surface Porosity**	0.5%	2.0%	5.0%	10.0%	20.0%

Table D-1: Rock properties and thermodynamic constraints used in the HT3 simulations. Permeability has been adjusted by 1.5 orders of magnitude greater for the lower crust/granitoids, melt sheet and mid-upper crust, when compared to permeability values used by Abramov and Kring (2007)[11]. The permeability of breccias and sediments (excluding limestone units) is by 0.7 orders of magnitude greater than the initial permeability values modelled by Abramov and Kring (2007)[11].

Results

Temperature distributions from 0 to 10 M years after the impact at the Chicxulub crater: (simulation ends with 70°C/km geotherm)

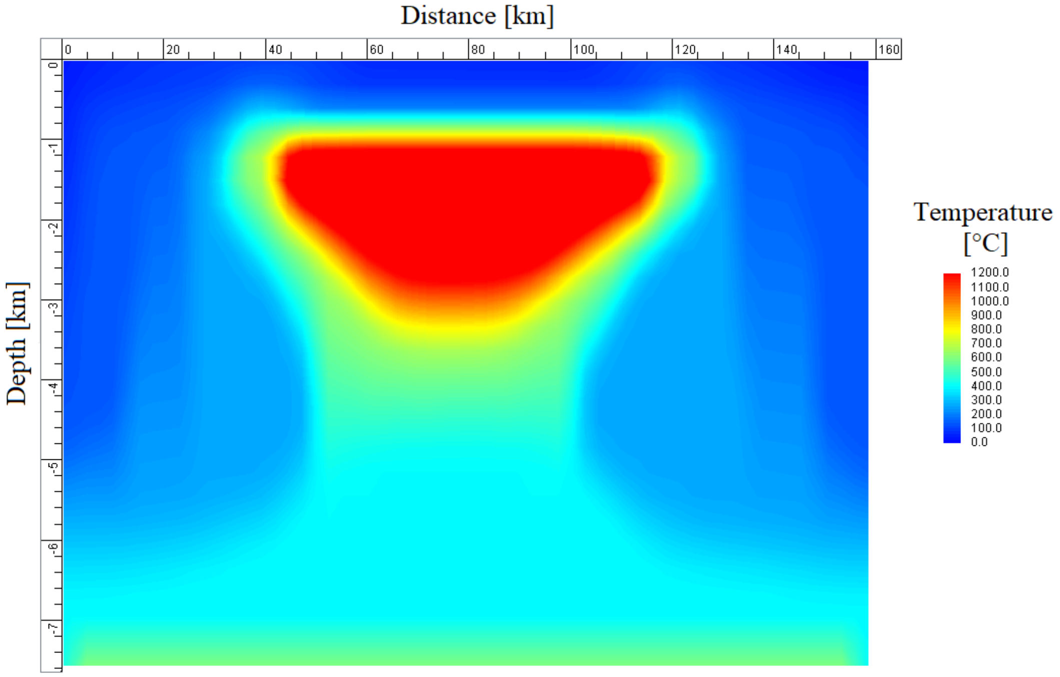

Time: 0 years

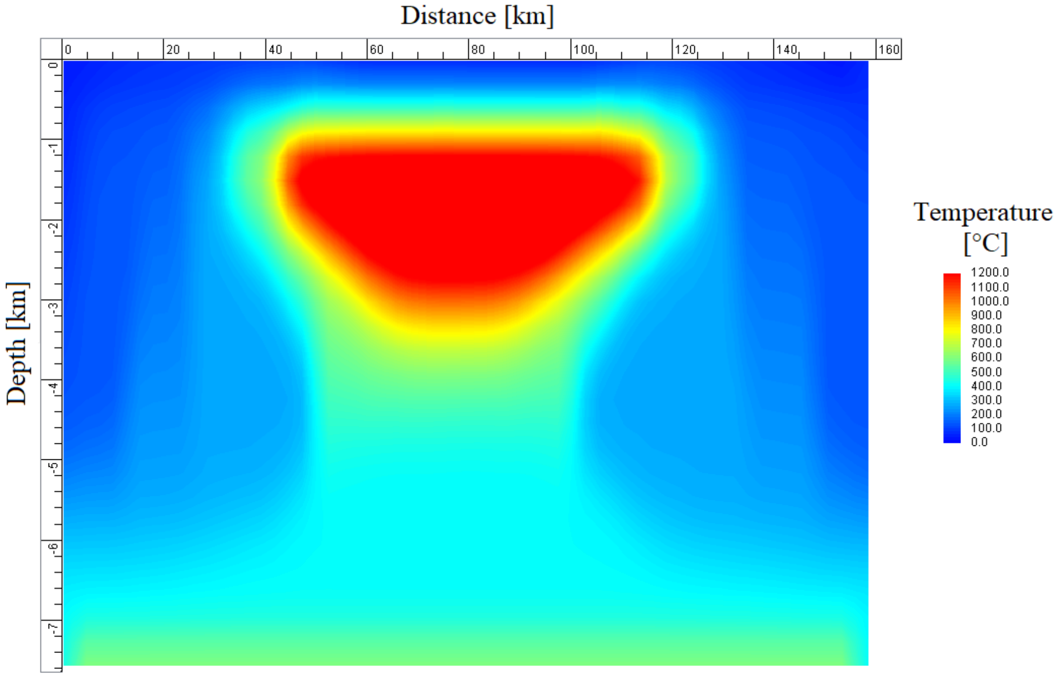

Time: 2.000 years

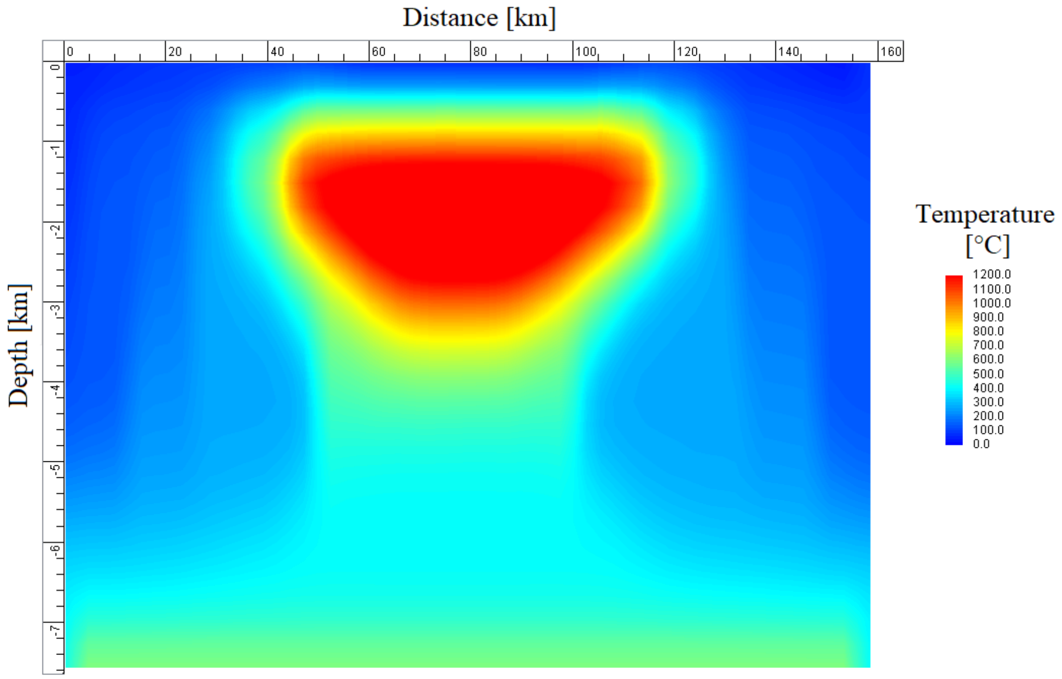

Time: 4.000 years

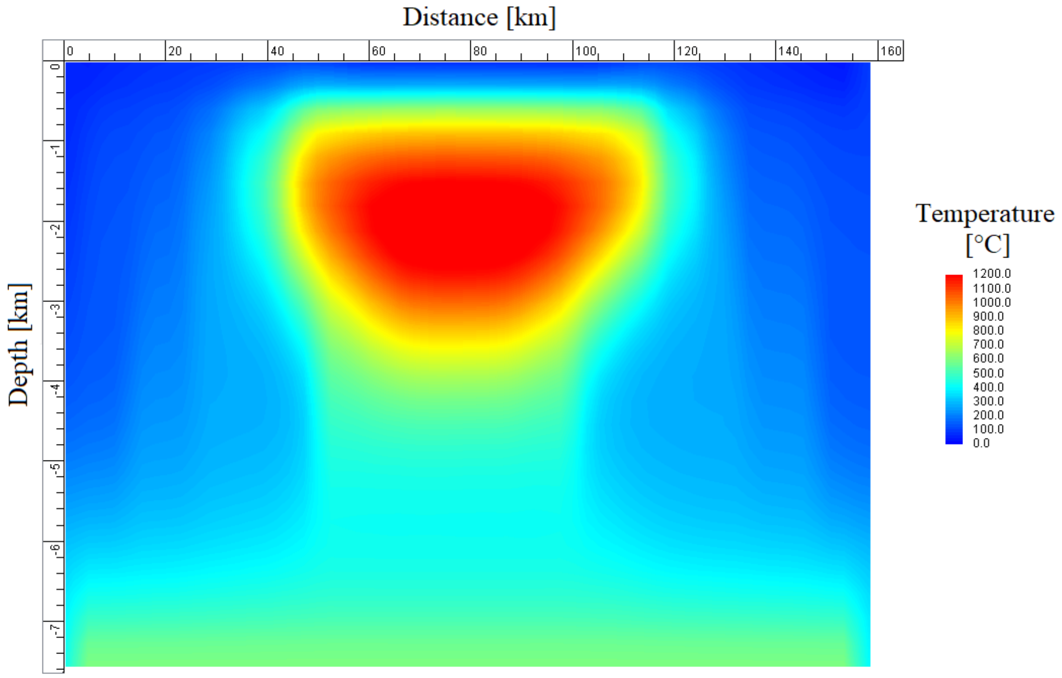

Time: 10.000 years

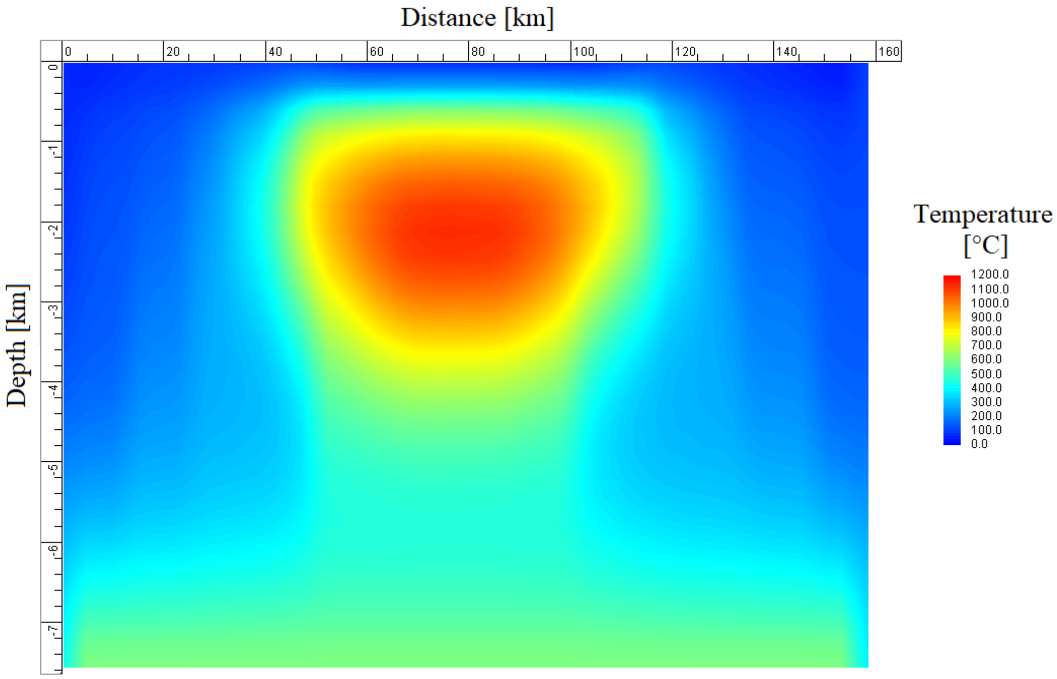

Time: 20.000 years

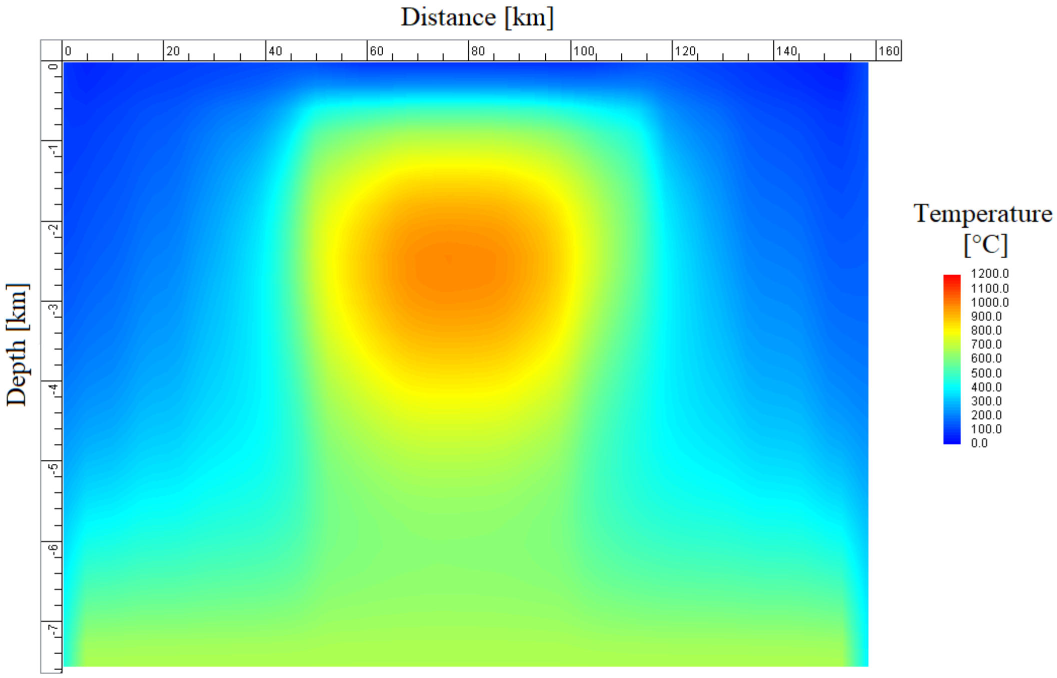

Time: 50.000 years

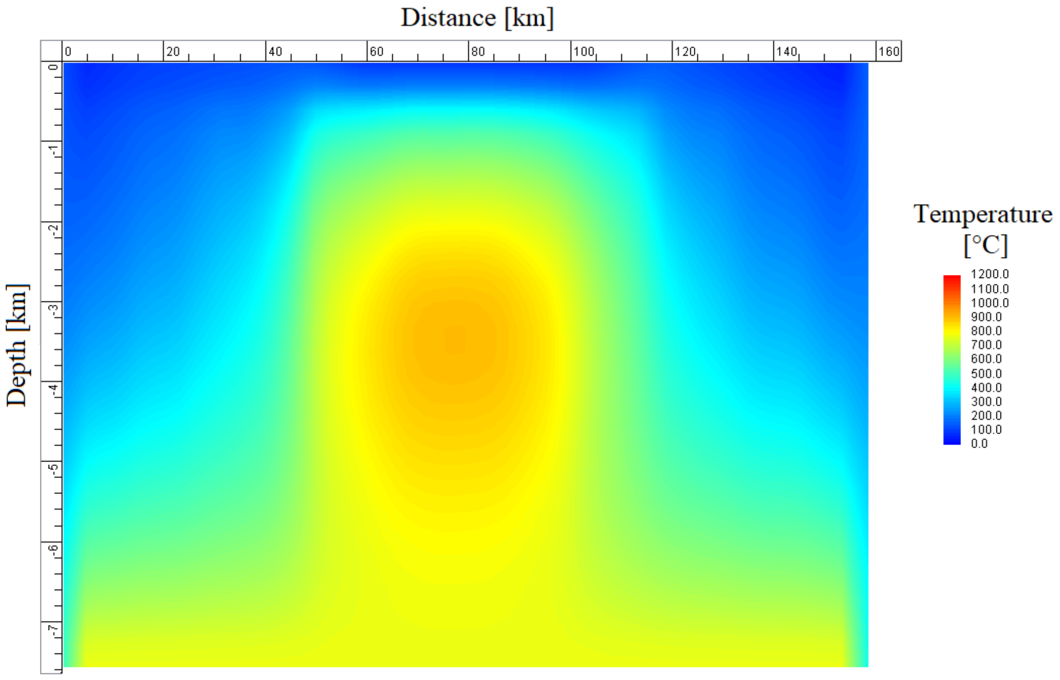

Time: 100.000 years

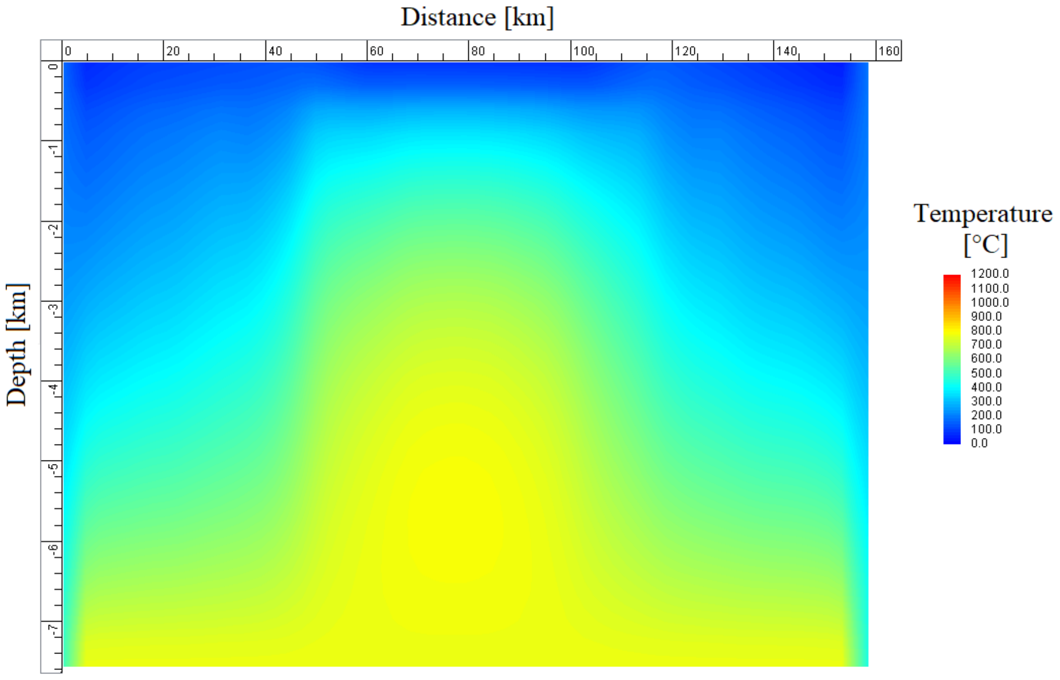

Time: 200.000 years

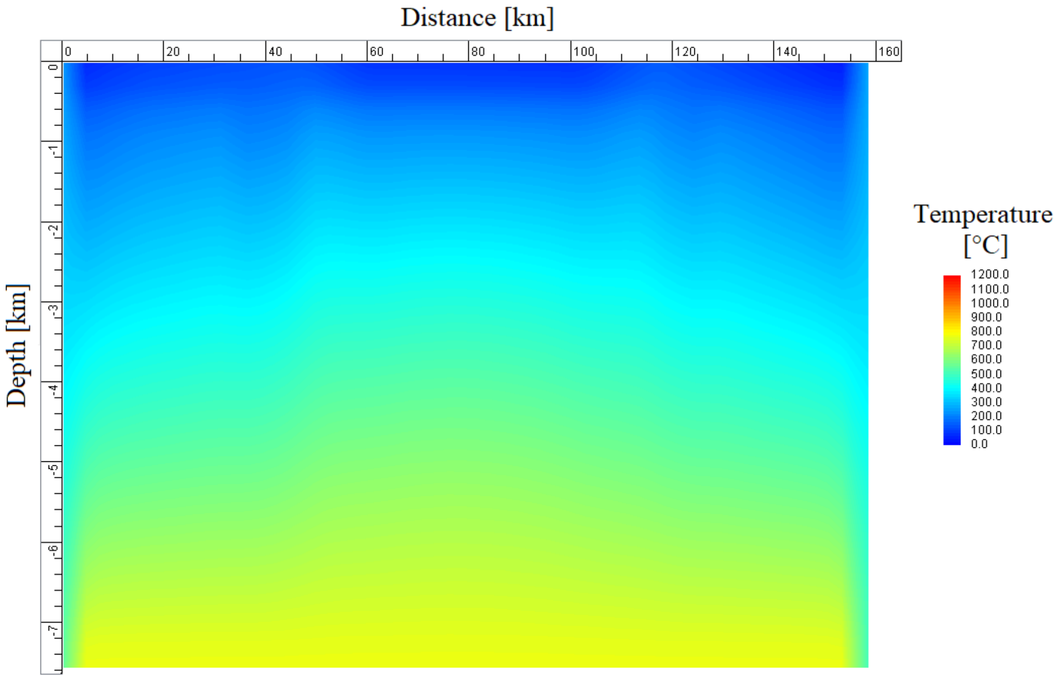

Time: 500.000 years

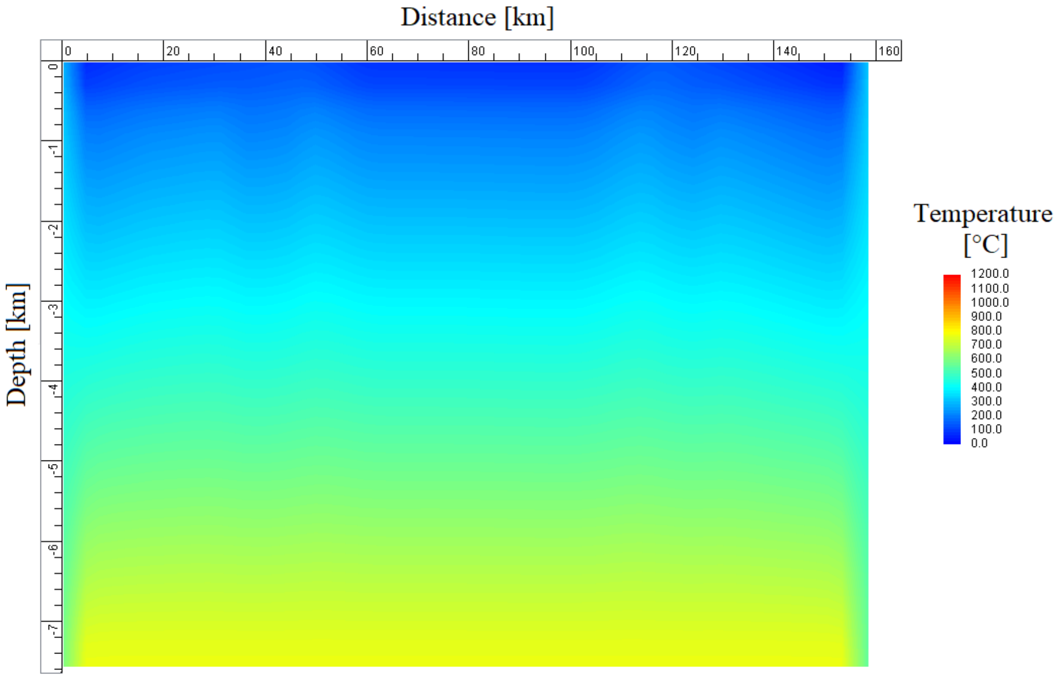

Time: 1.000.000 years

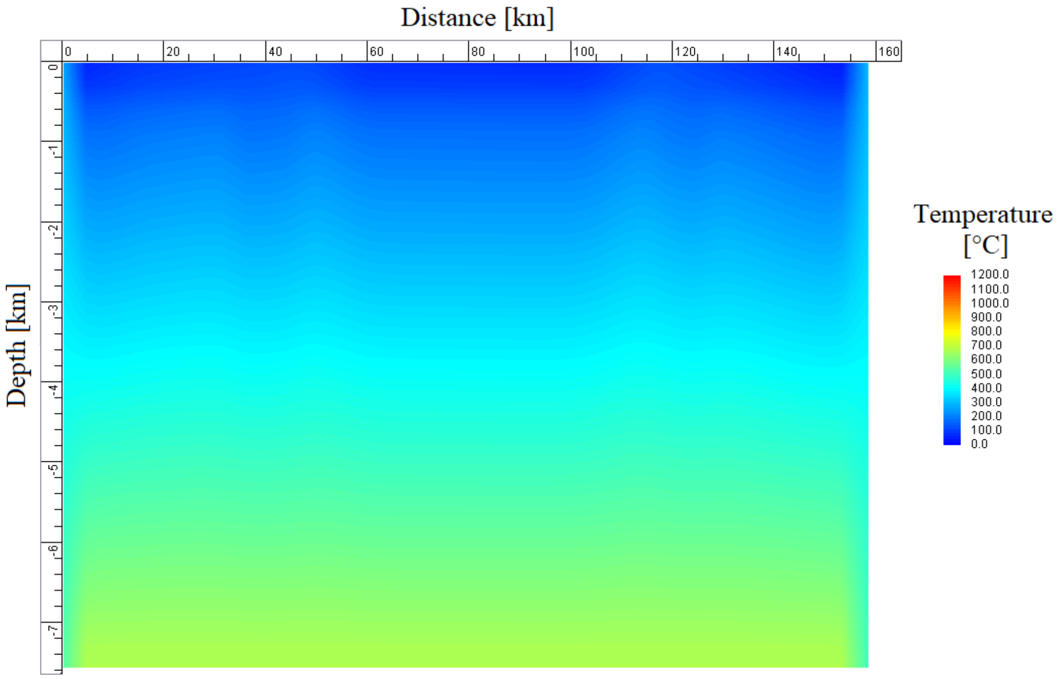

Time: 2.000.000 years

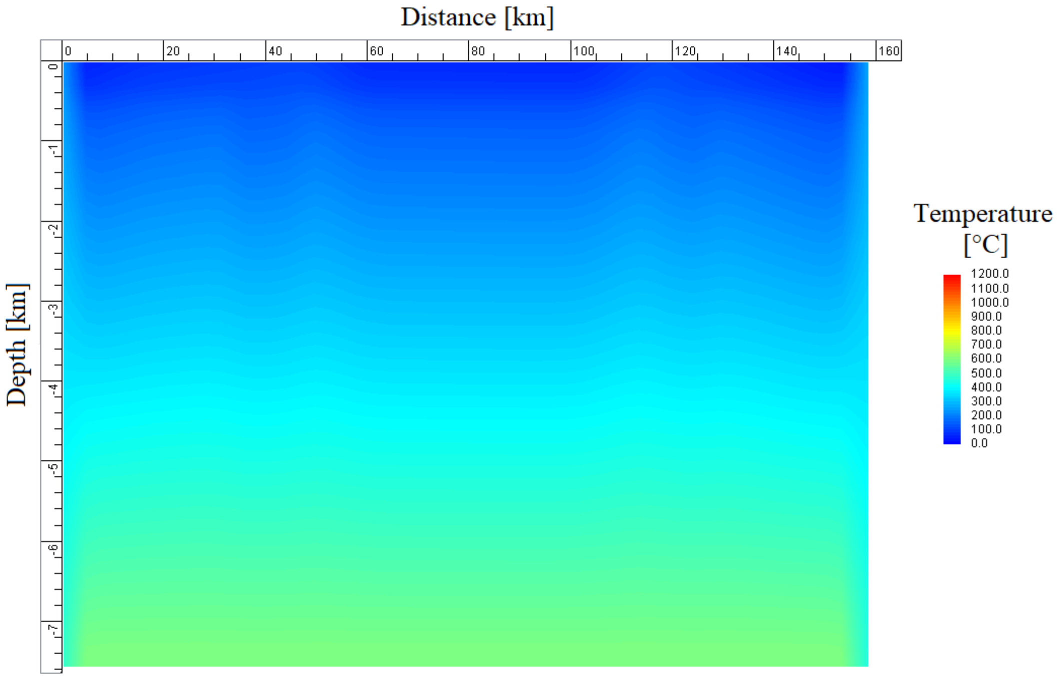

Time: 3.000.000 years

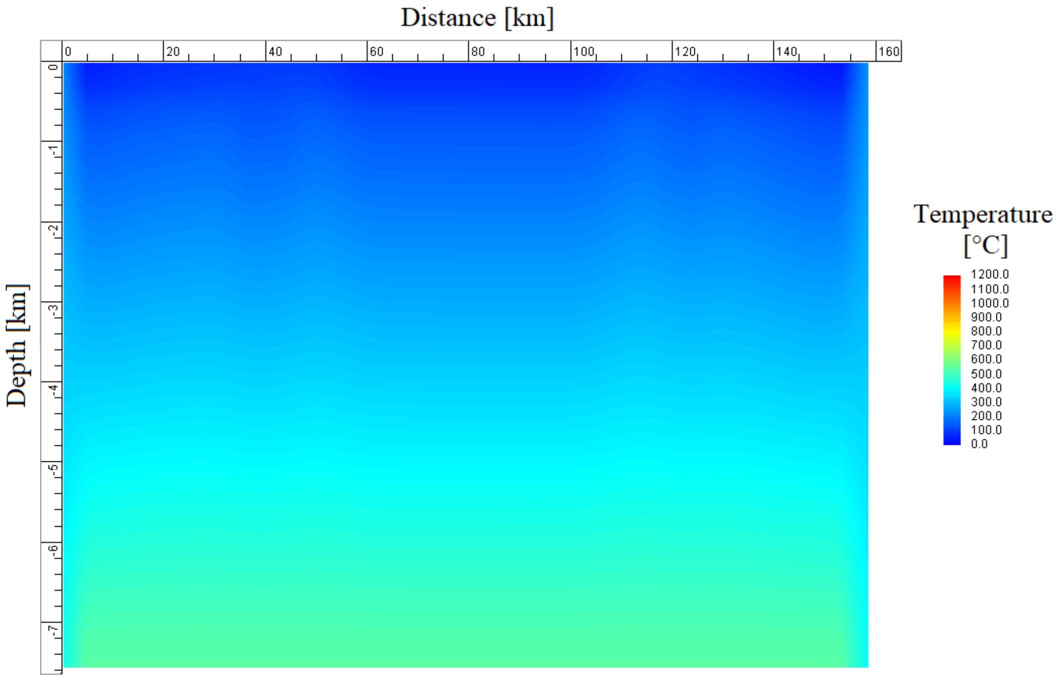

Time: 4.000.000 years

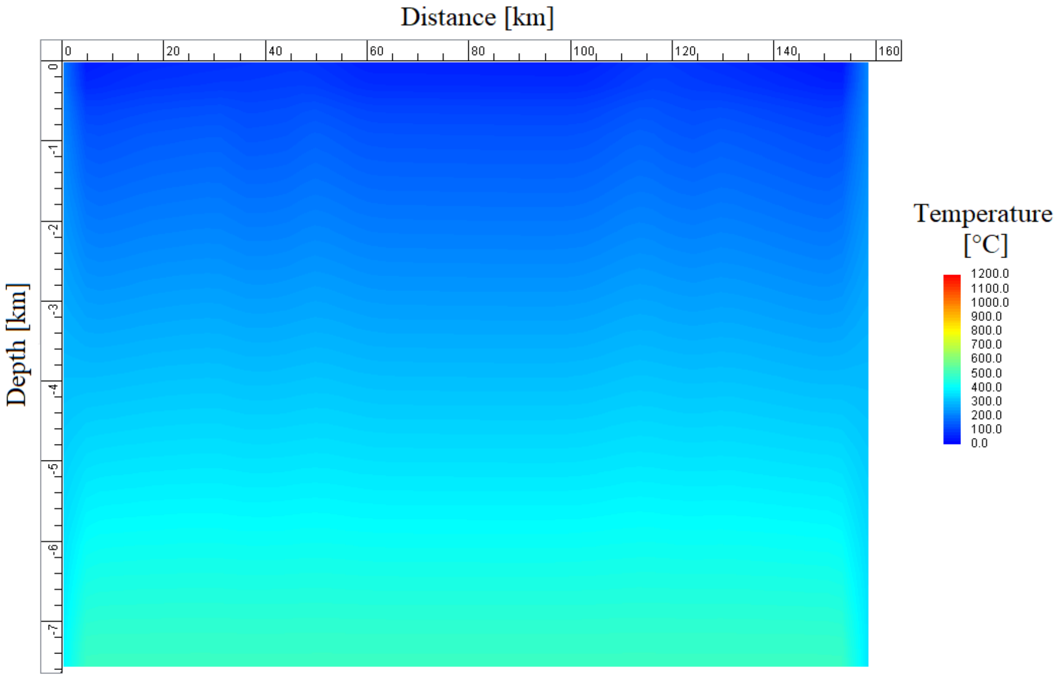

Time: 5.000.000 years

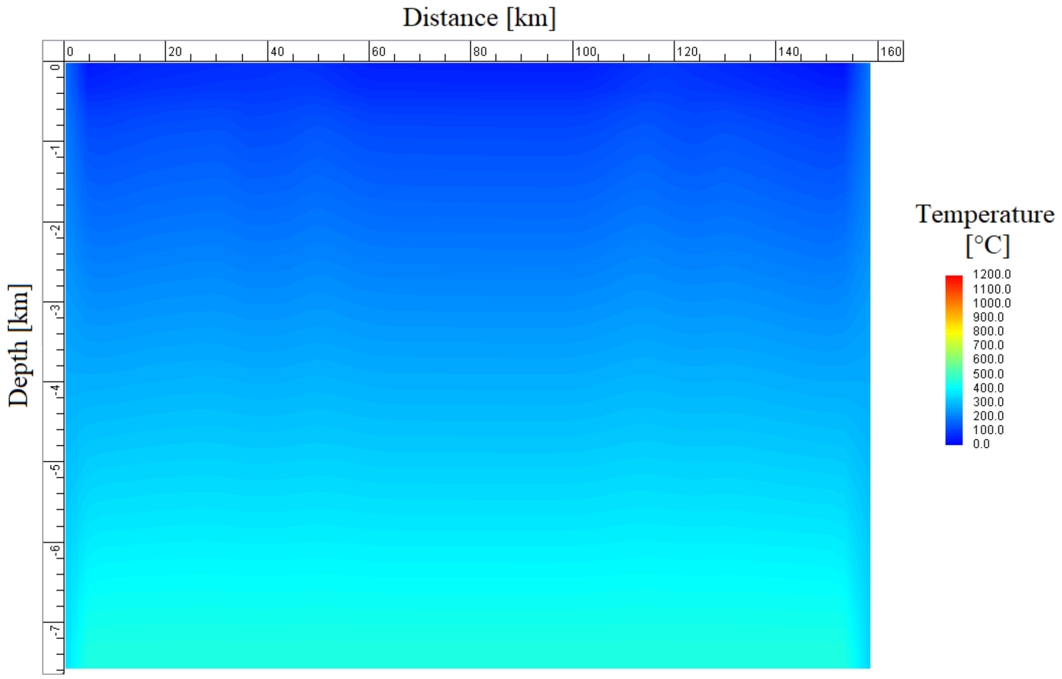

Time: 6.000.000 years

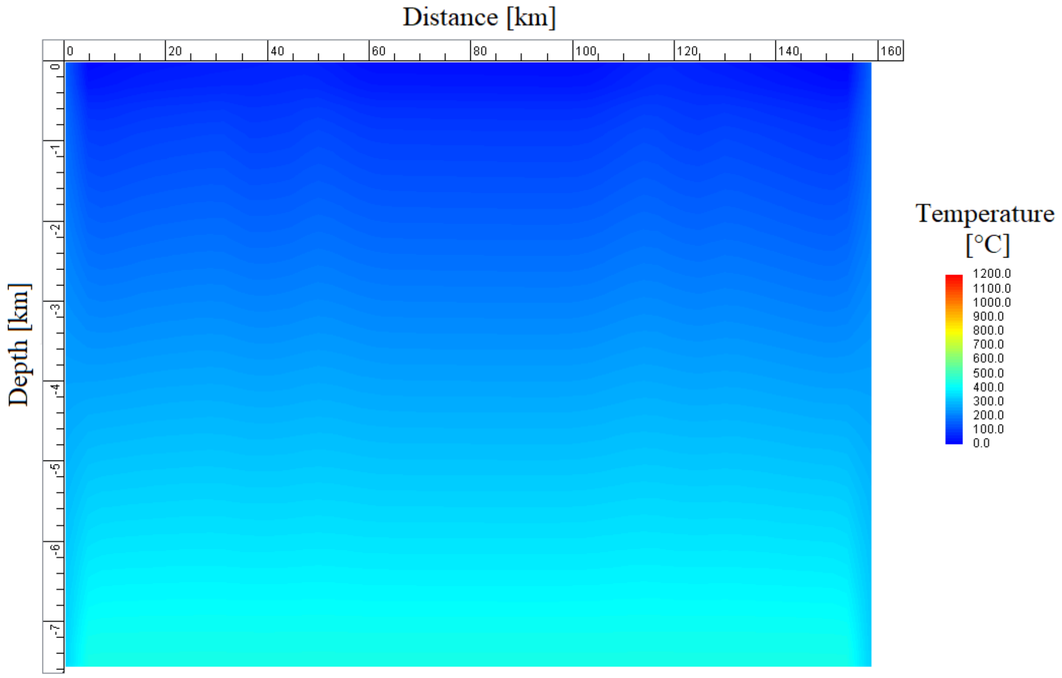

Time: 6.250.000 years

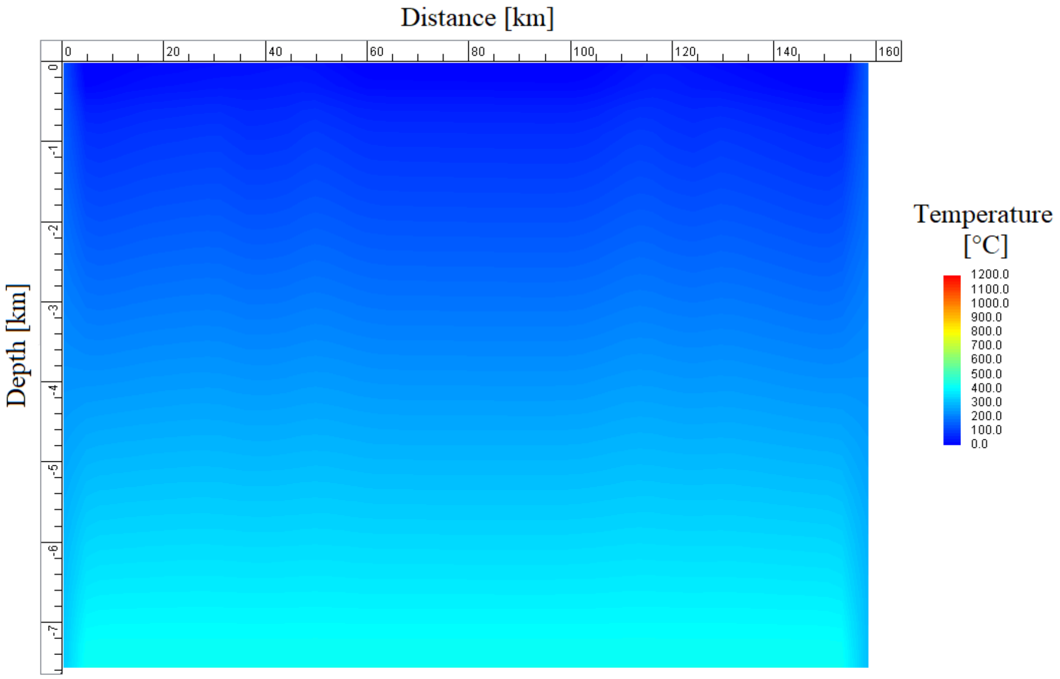

Time: 6.500.000 years

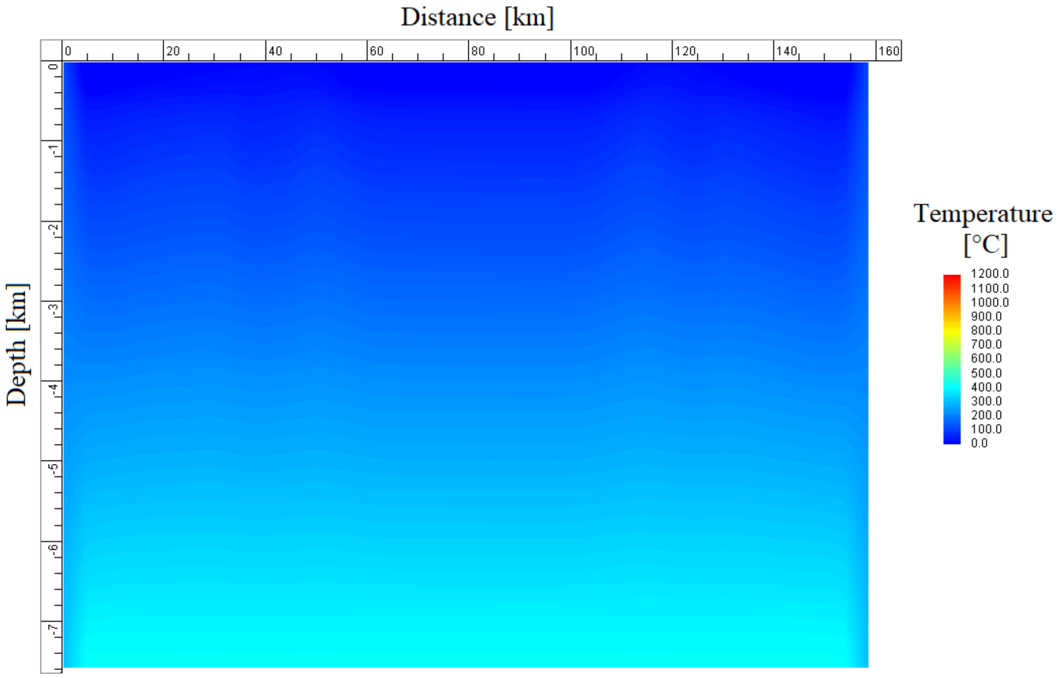

Time: 7.000.000 years

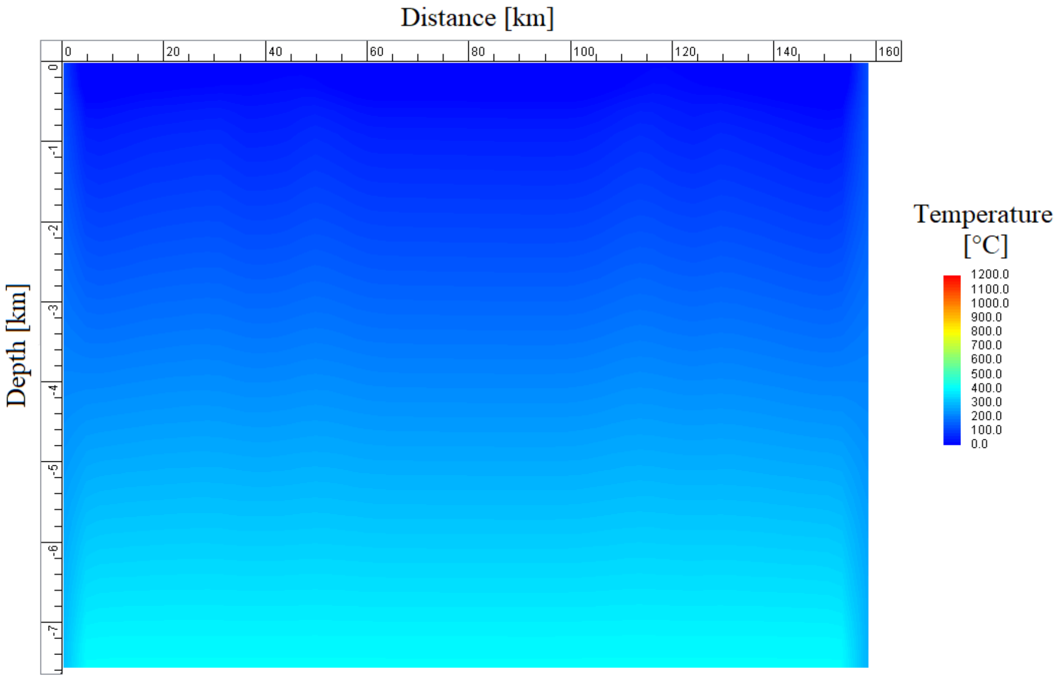

Time: 7.500.000 years

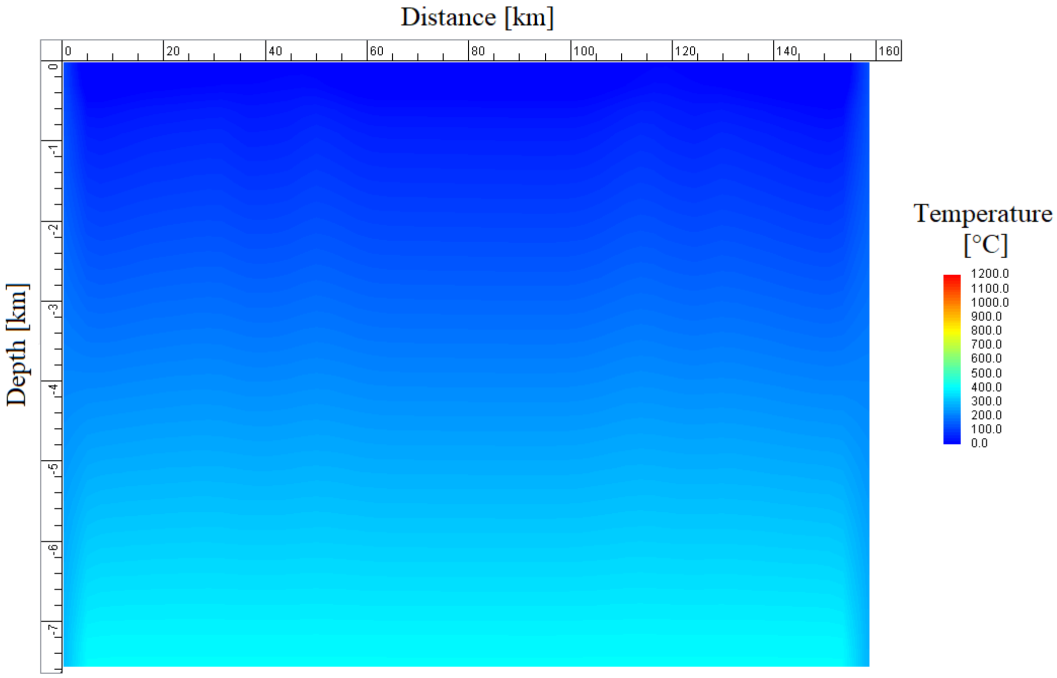

Time: 8.000.000 years

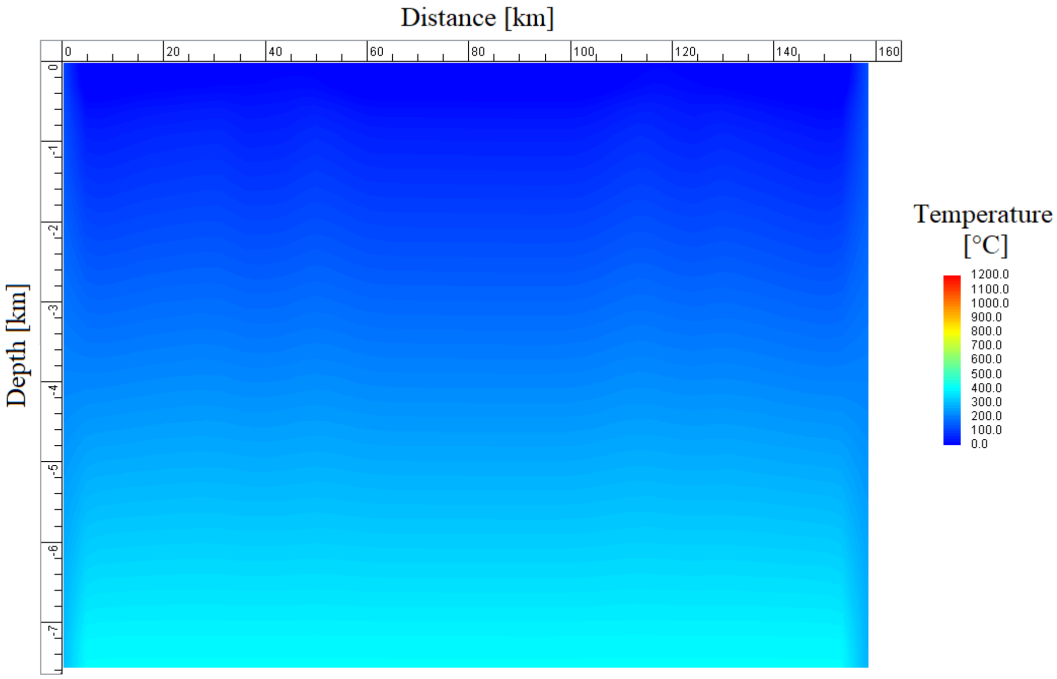

Time: 9.000.000 years

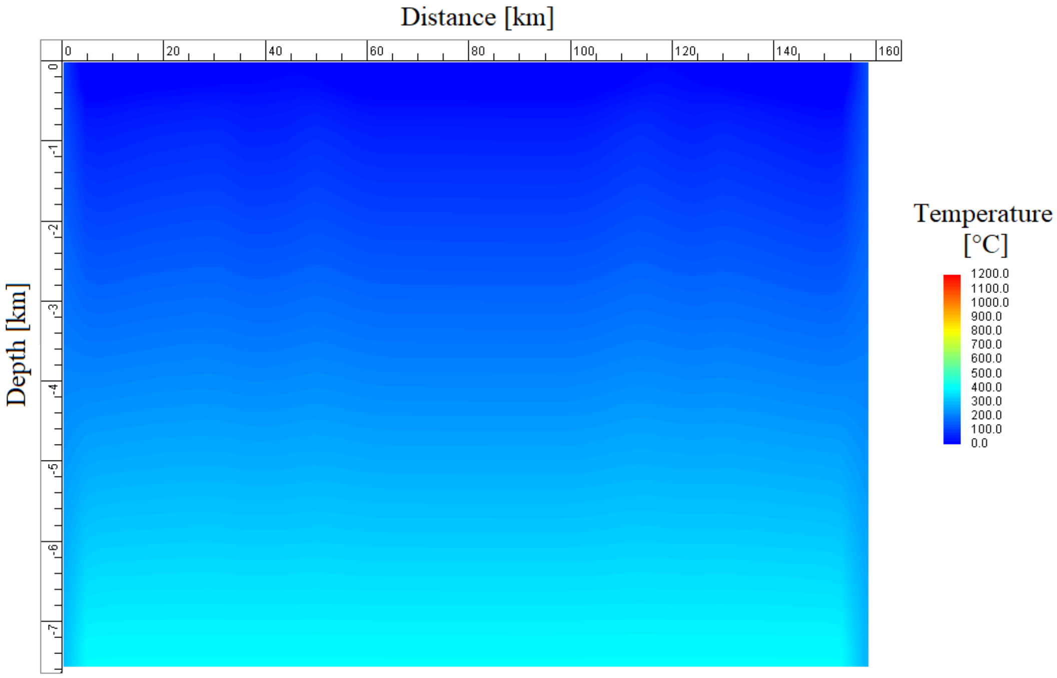

Time: 10.000.000 years

Average water mass flux distributions, steam and (super-critical) fluid mass flux vectors from 0 to 10M years after the impact at the Chicxulub crater:

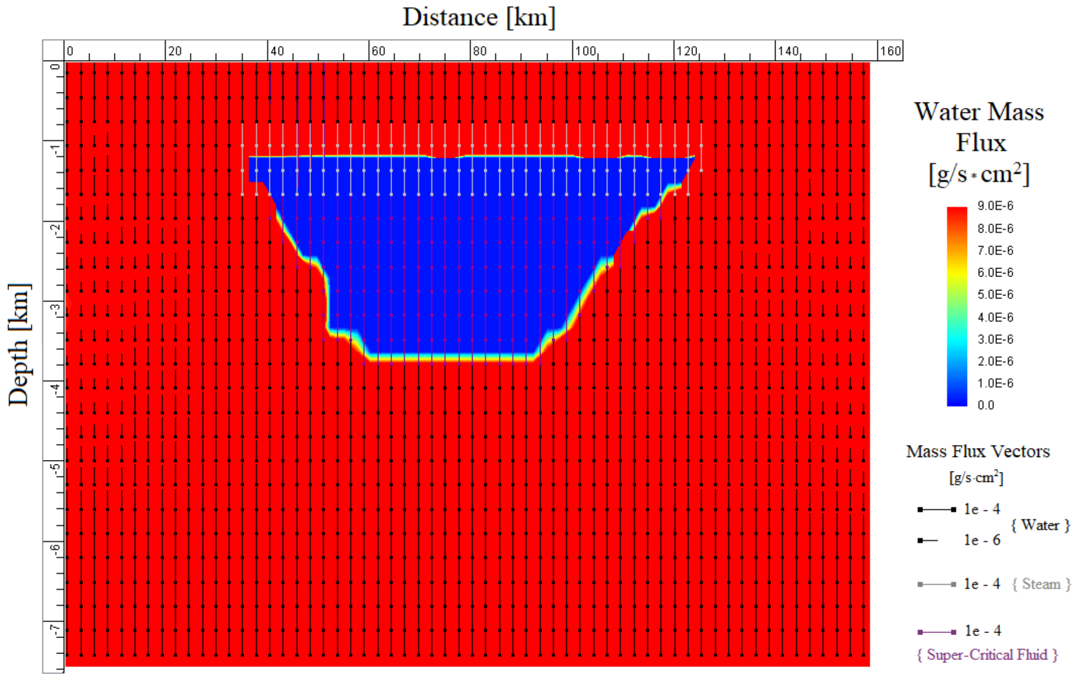

Time: 0 years

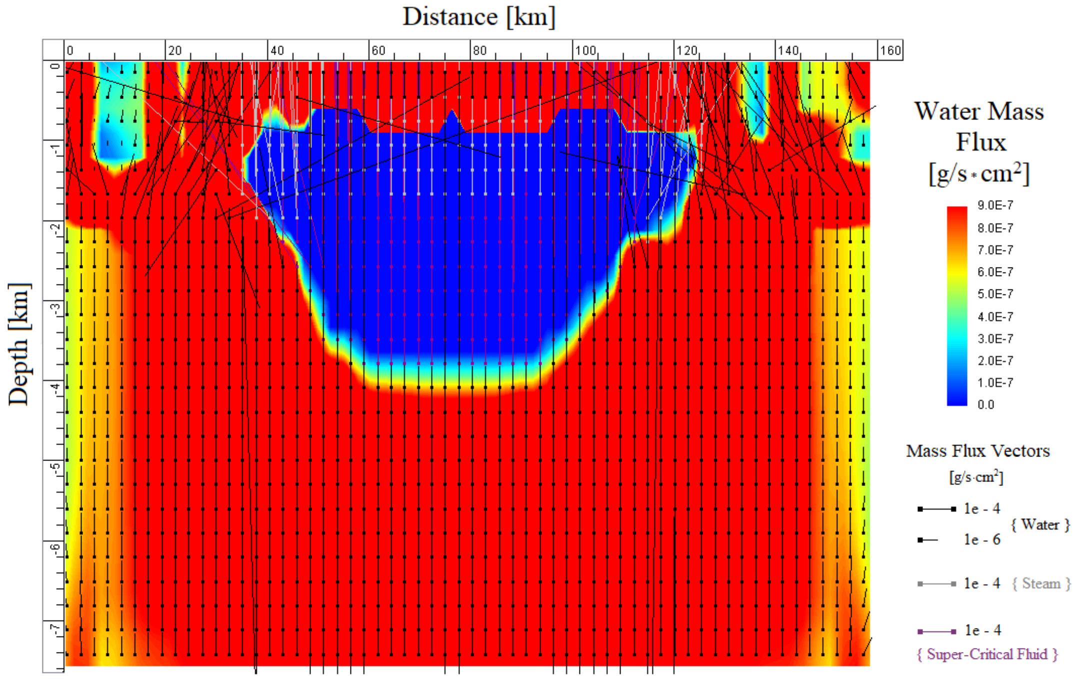

Time: 2.000 years

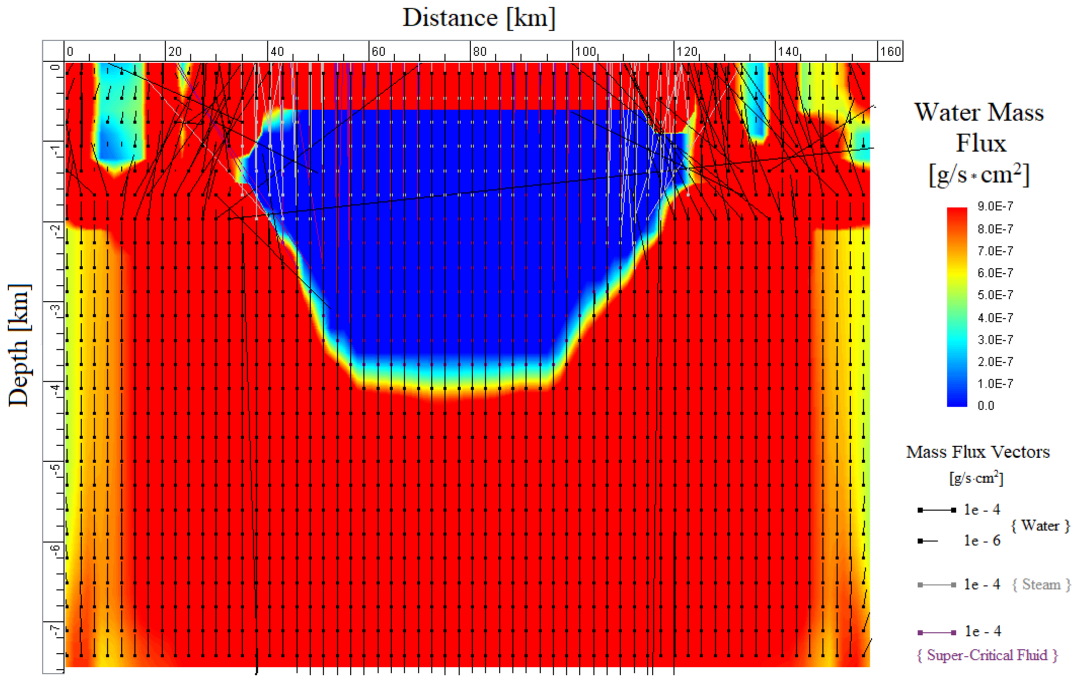

Time: 4.000 years

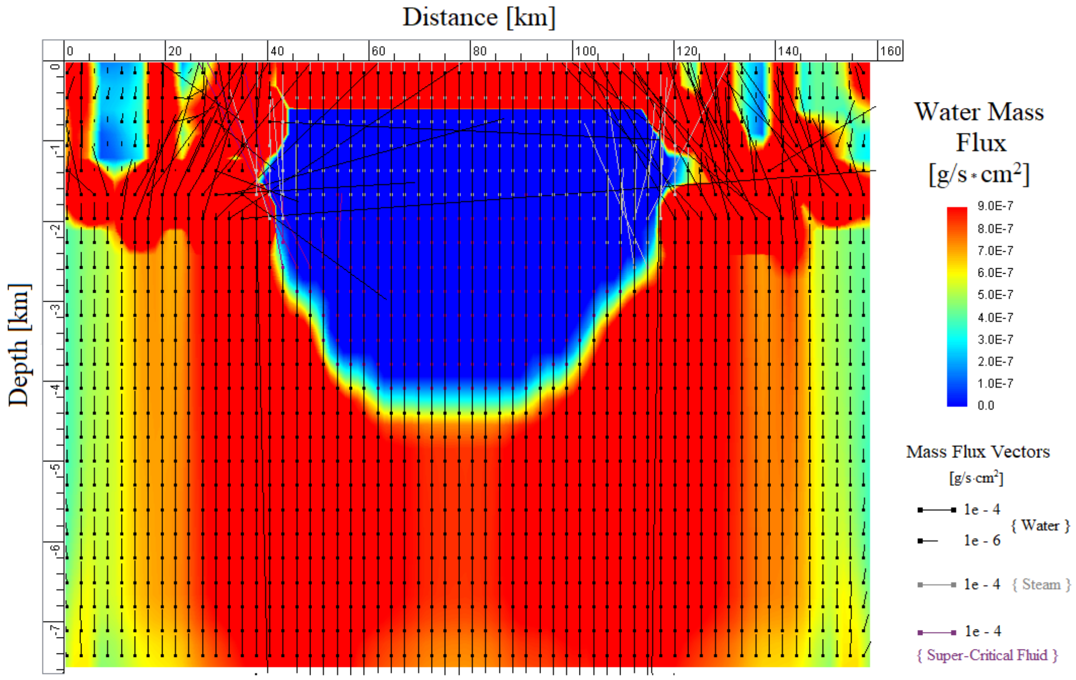

Time: 10.000 years

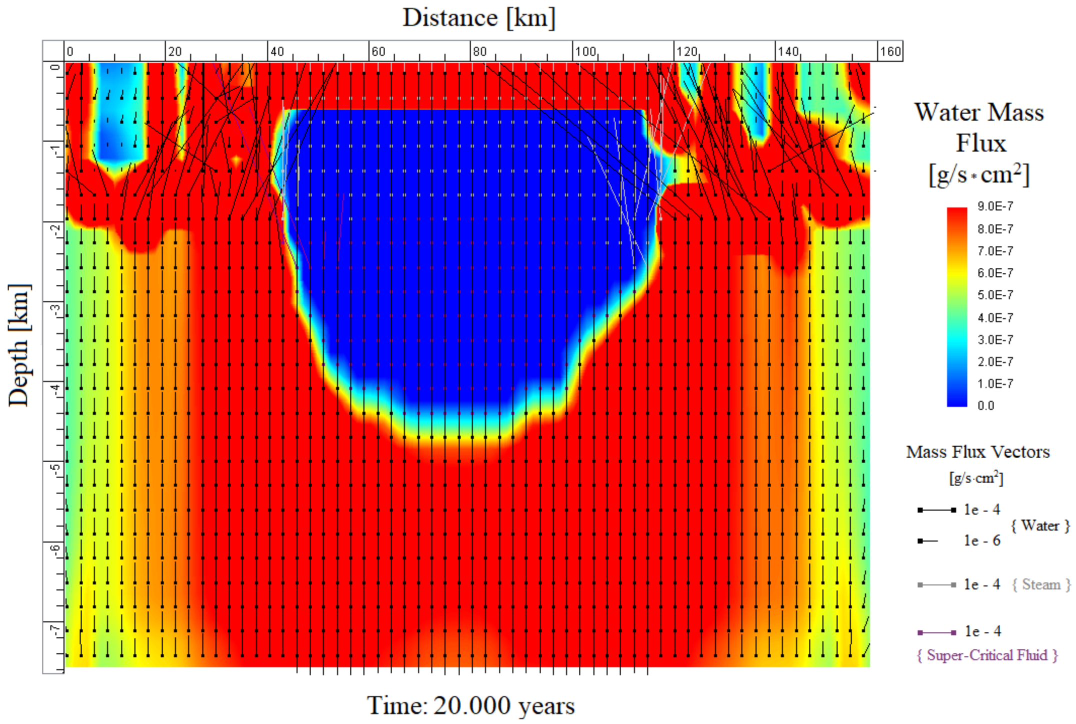
Distance [km]
0
20
40
60
80
100
120
140
160
Depth [km]
Water Mass Flux [g/s*cm2]
9.0E-7
8.0E-7
7.0E-7
6.0E-7
5.0E-7
4.0E-7
3.0E-7
2.0E-7
1.0E-7
0.0
Mass Flux Vectors [g/s·cm2]
1e - 4
1e - 6
{ Water }
1e - 4 { Steam }
1e - 4
{ Super-Critical Fluid }
Time: 20.000 years

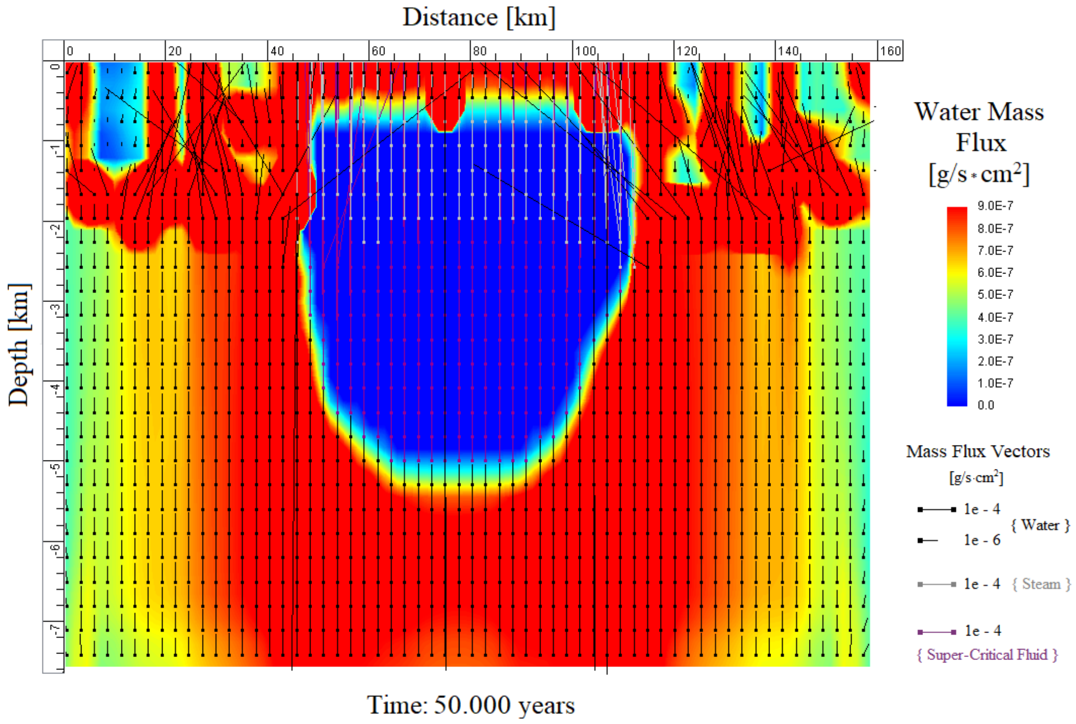
Distance [km]
0
20
40
60
80
100
120
140
160
Depth [km]
Water Mass Flux [g/s*cm2]
9.0E-7
8.0E-7
7.0E-7
6.0E-7
5.0E-7
4.0E-7
3.0E-7
2.0E-7
1.0E-7
0.0
Mass Flux Vectors [g/s·cm2]
1e - 4
1e - 6
{ Water }
1e - 4 { Steam }
1e - 4
{ Super-Critical Fluid }
Time: 50.000 years

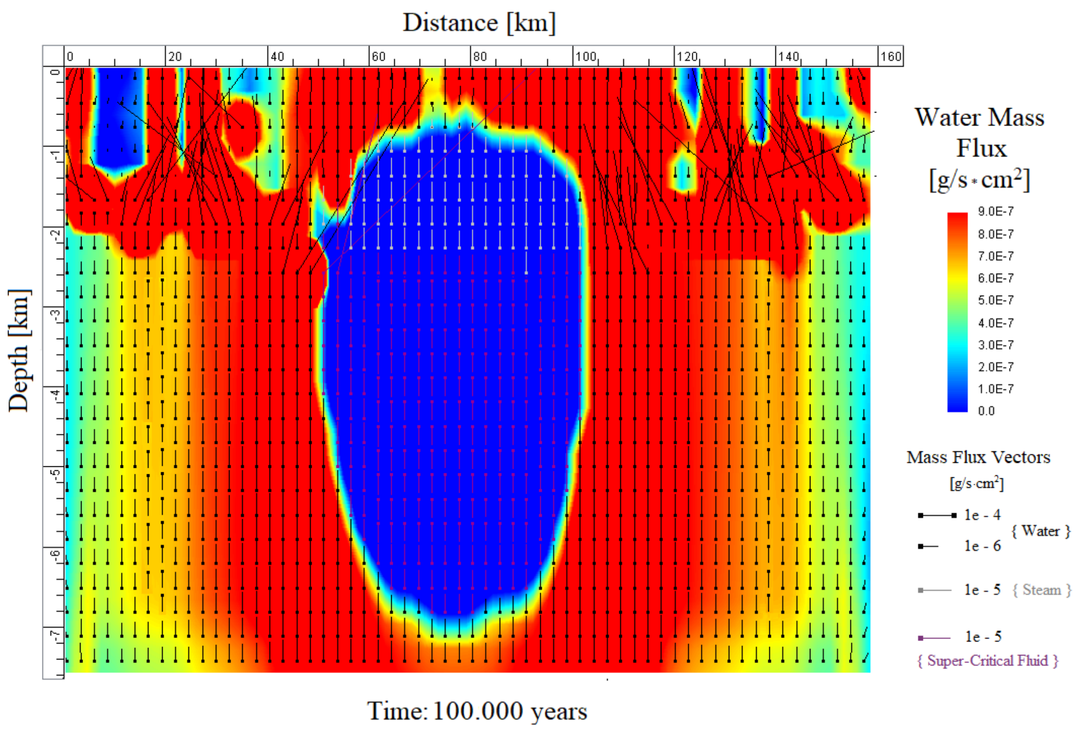

Distance [km]
0
20
40
60
80
100
120
140
160
Depth [km]
Water Mass
Flux
[g/s*cm²]
9.0E-7
8.0E-7
7.0E-7
6.0E-7
5.0E-7
4.0E-7
3.0E-7
2.0E-7
1.0E-7
0.0
Mass Flux Vectors
[g/s·cm²]
1e - 4
1e - 6
{ Water }
1e - 5 { Steam }
1e - 5
{ Super-Critical Fluid }
Time: 100.000 years

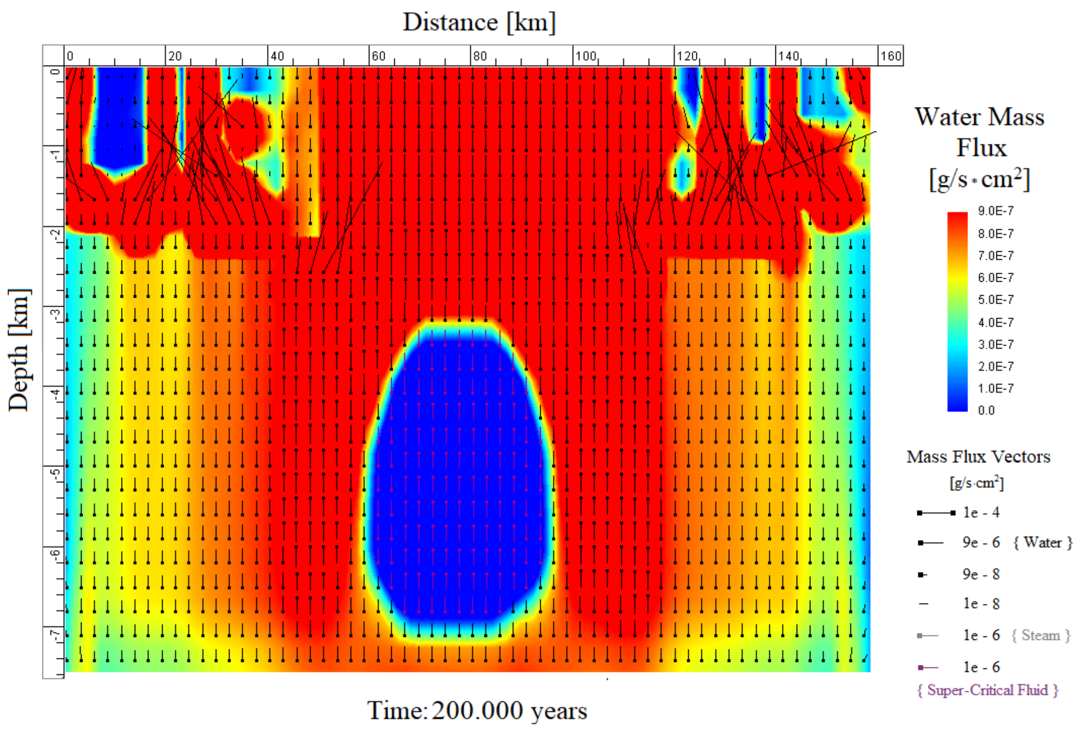

Distance [km]
0
20
40
60
80
100
120
140
160
Depth [km]
Water Mass
Flux
[g/s*cm²]
9.0E-7
8.0E-7
7.0E-7
6.0E-7
5.0E-7
4.0E-7
3.0E-7
2.0E-7
1.0E-7
0.0
Mass Flux Vectors
[g/s·cm²]
1e - 4
9e - 6 { Water }
9e - 8
1e - 8
1e - 6 { Steam }
1e - 6
{ Super-Critical Fluid }
Time: 200.000 years

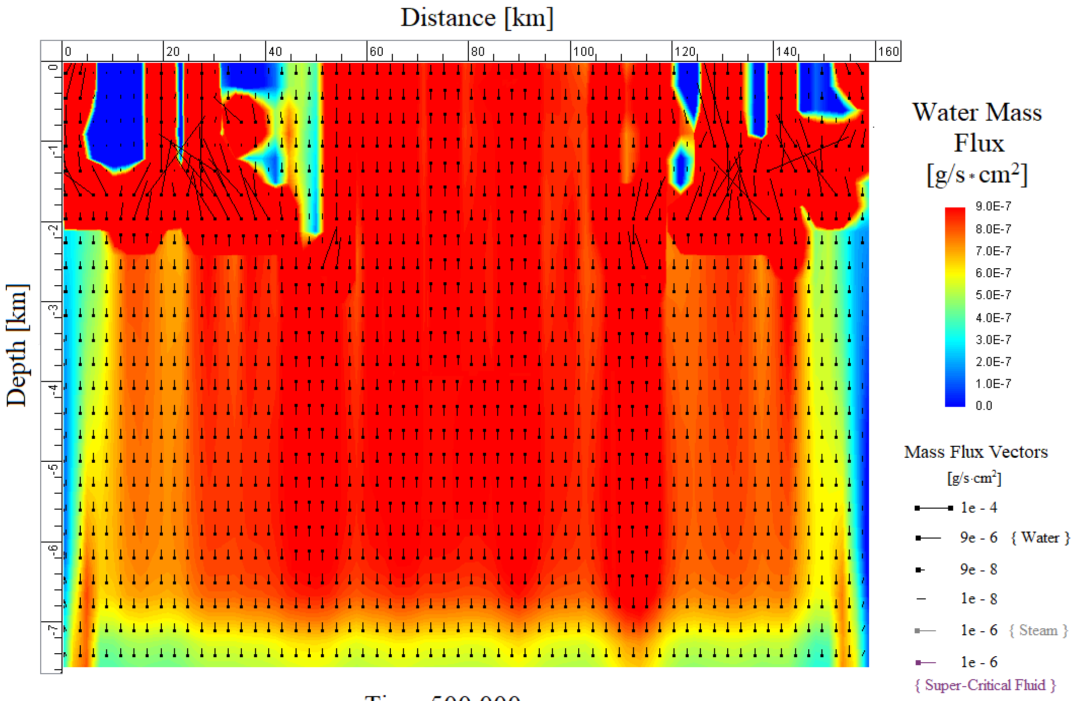

Time: 500.000 years

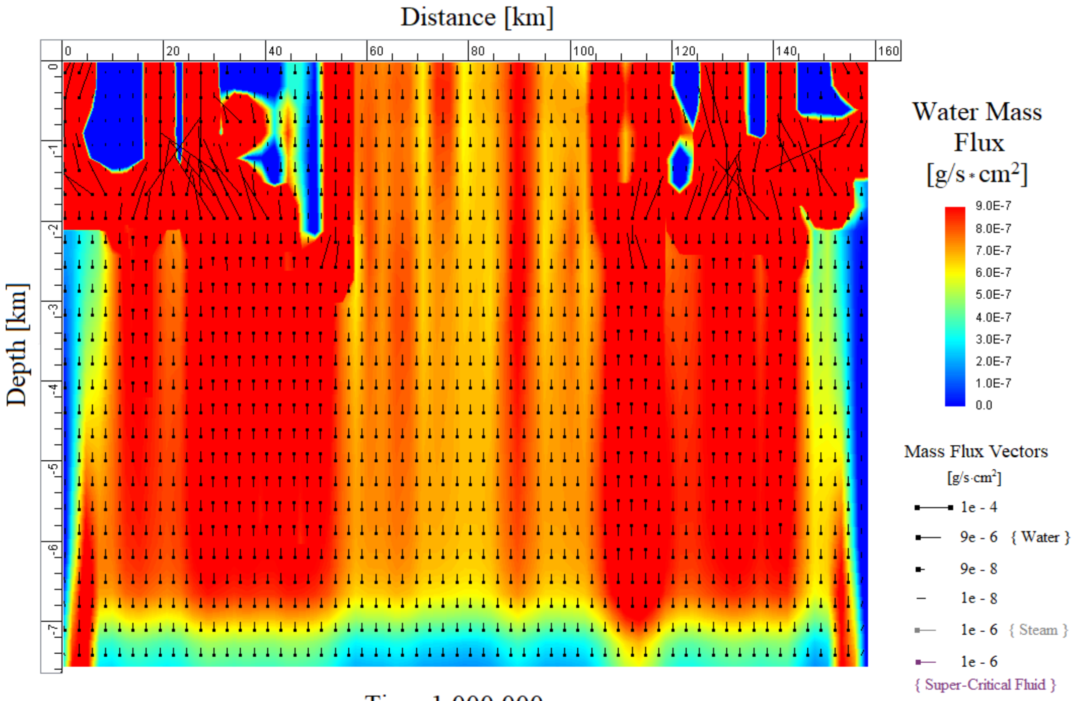

Time: 1.000.000 years

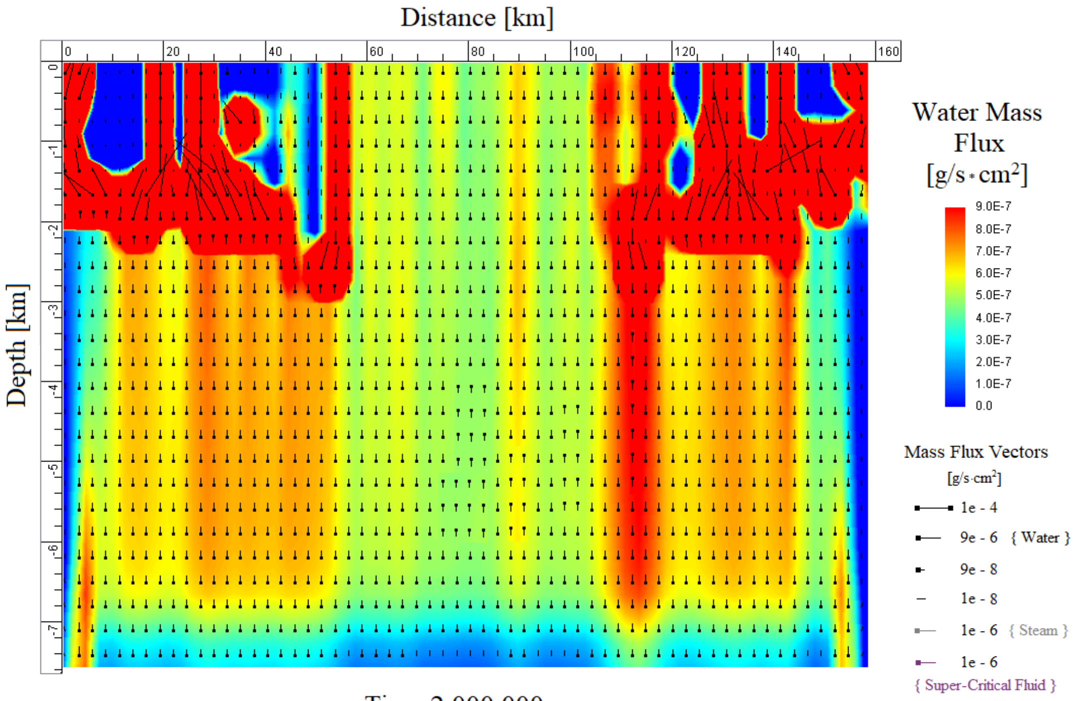

Time: 2.000.000 years

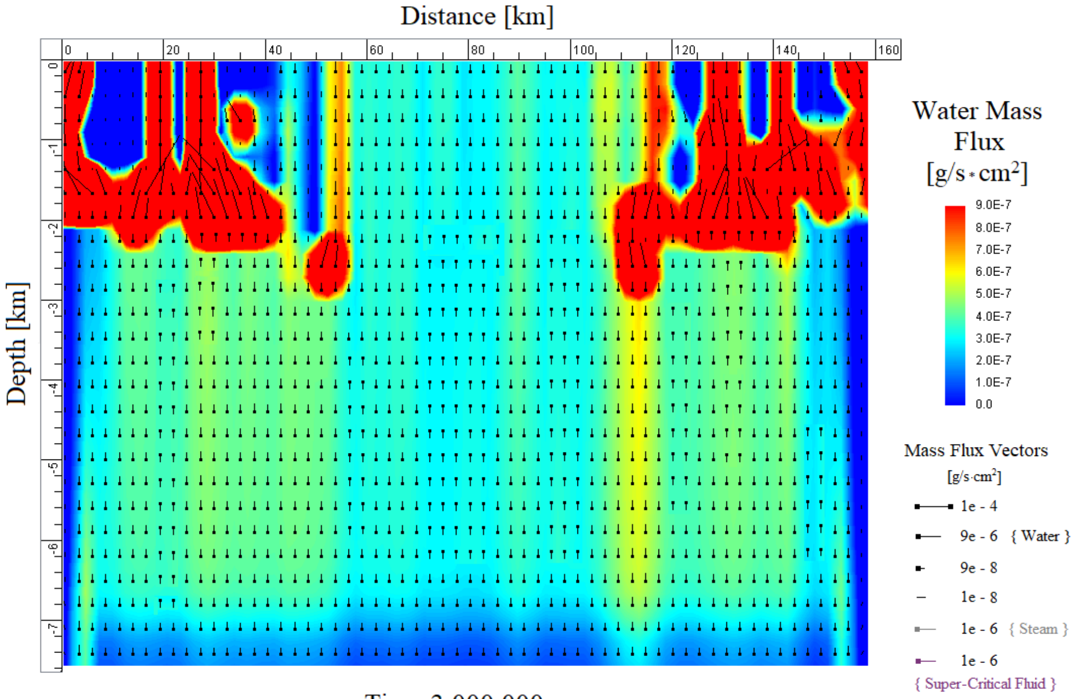

Time: 3.000.000 years

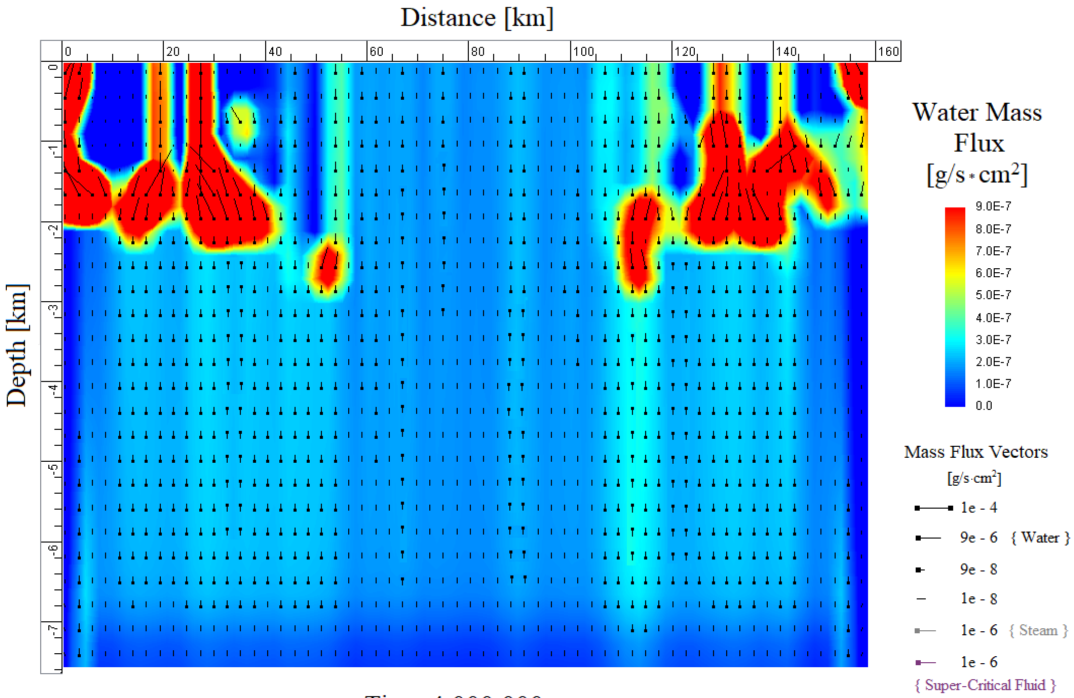

Time: 4.000.000 years

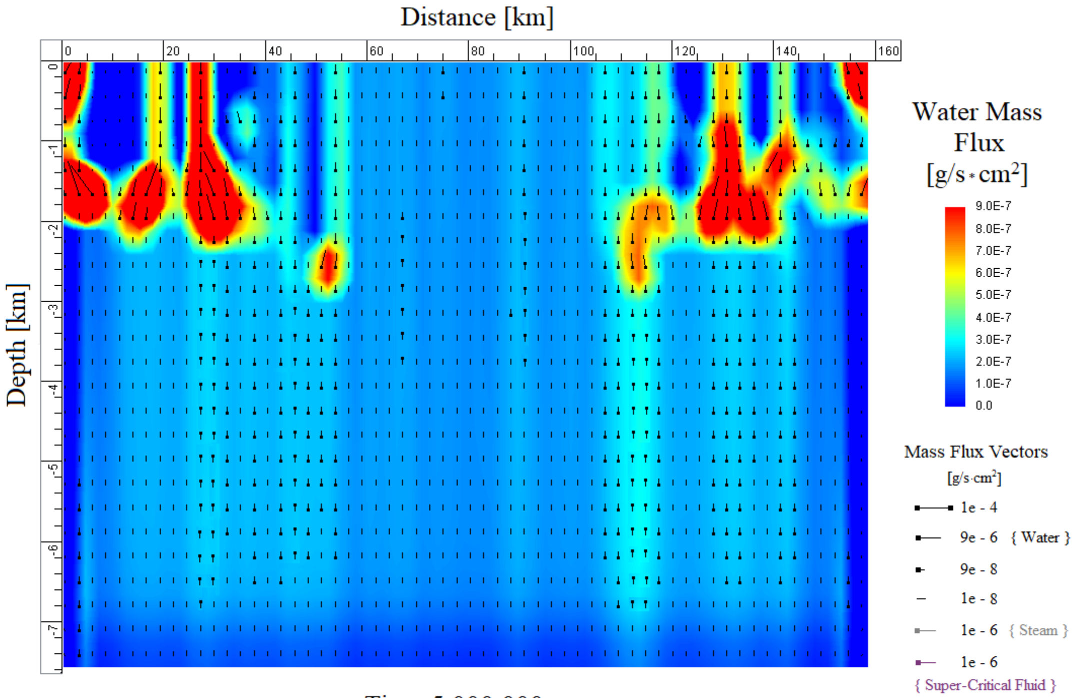

Time: 5.000.000 years

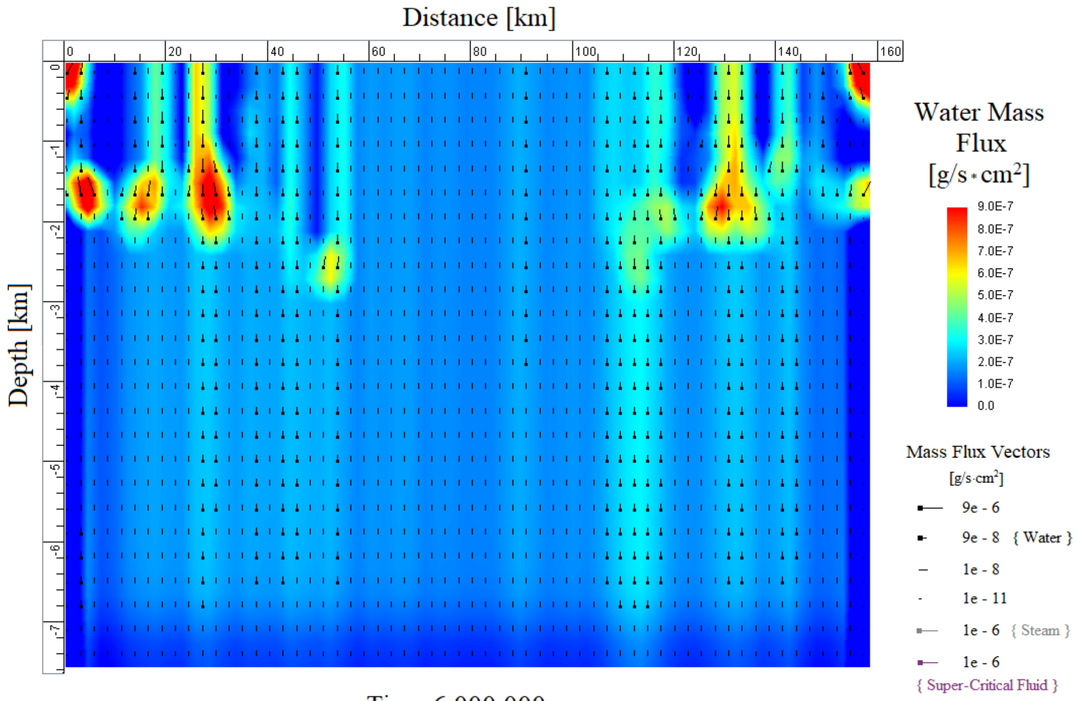

Time: 6.000.000 years

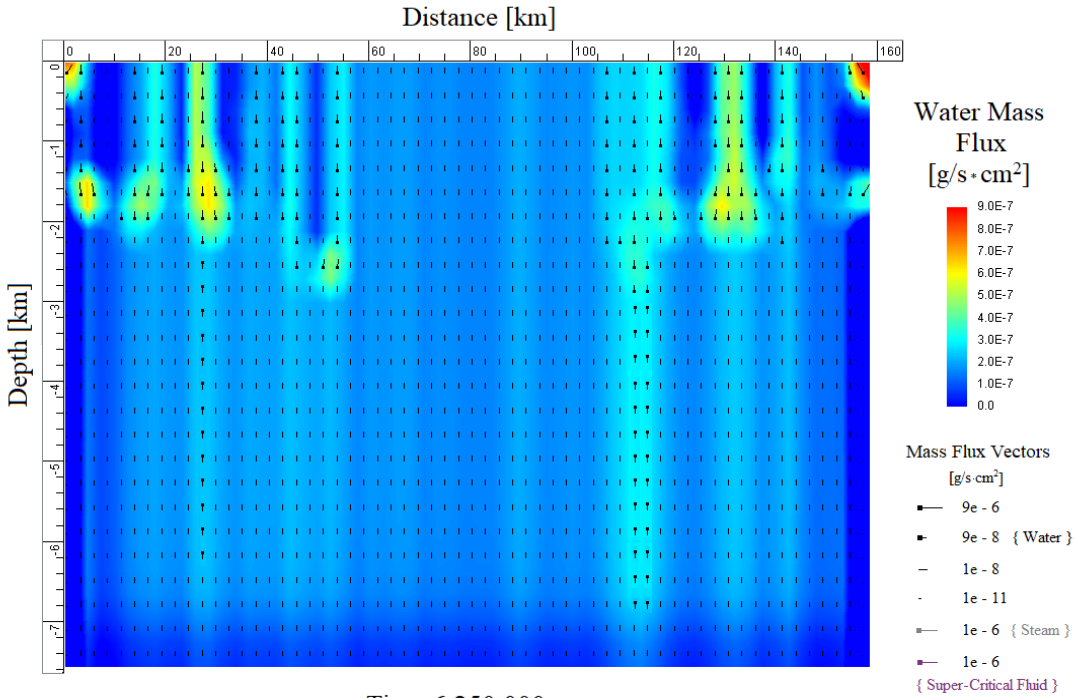

Time: 6.250.000 years

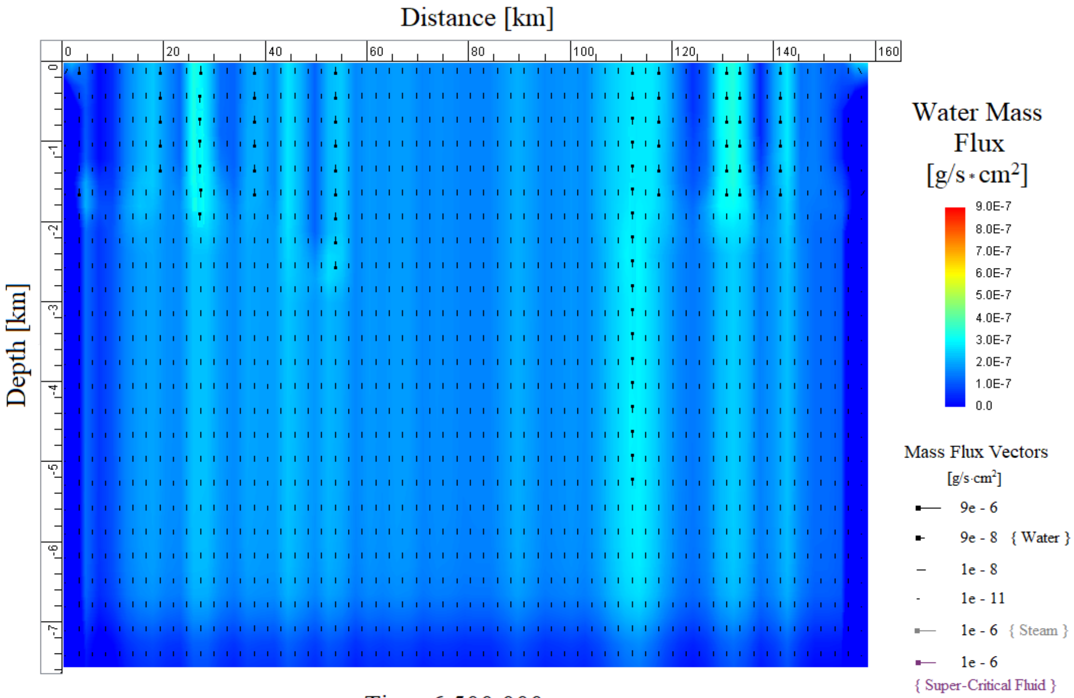

Time: 6.500.000 years

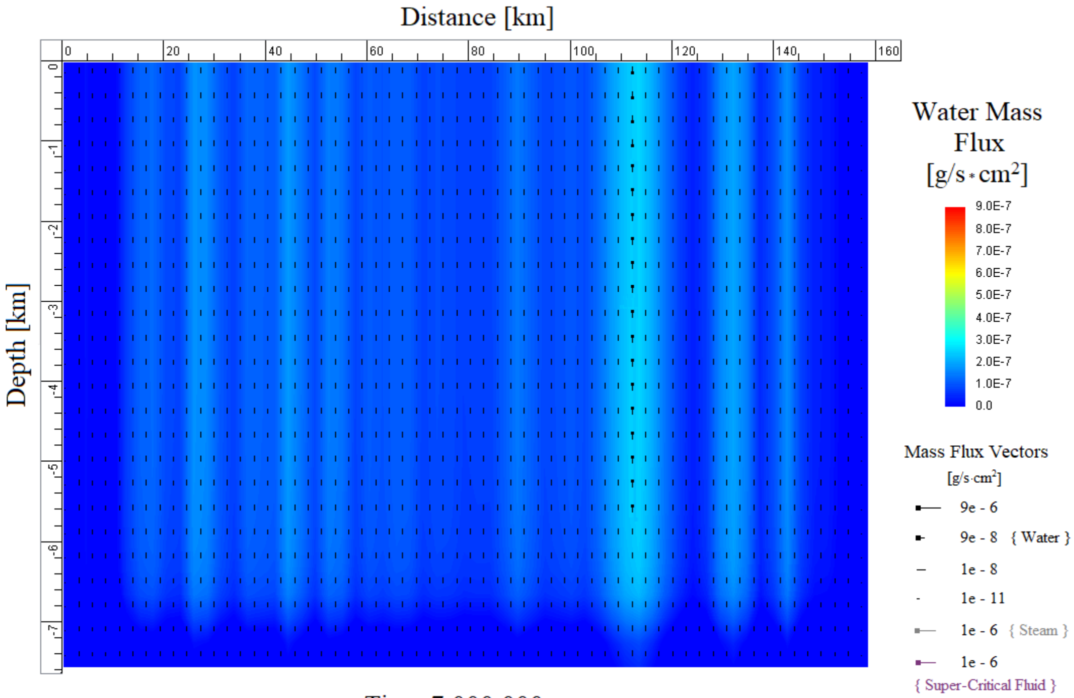

Time: 7.000.000 years

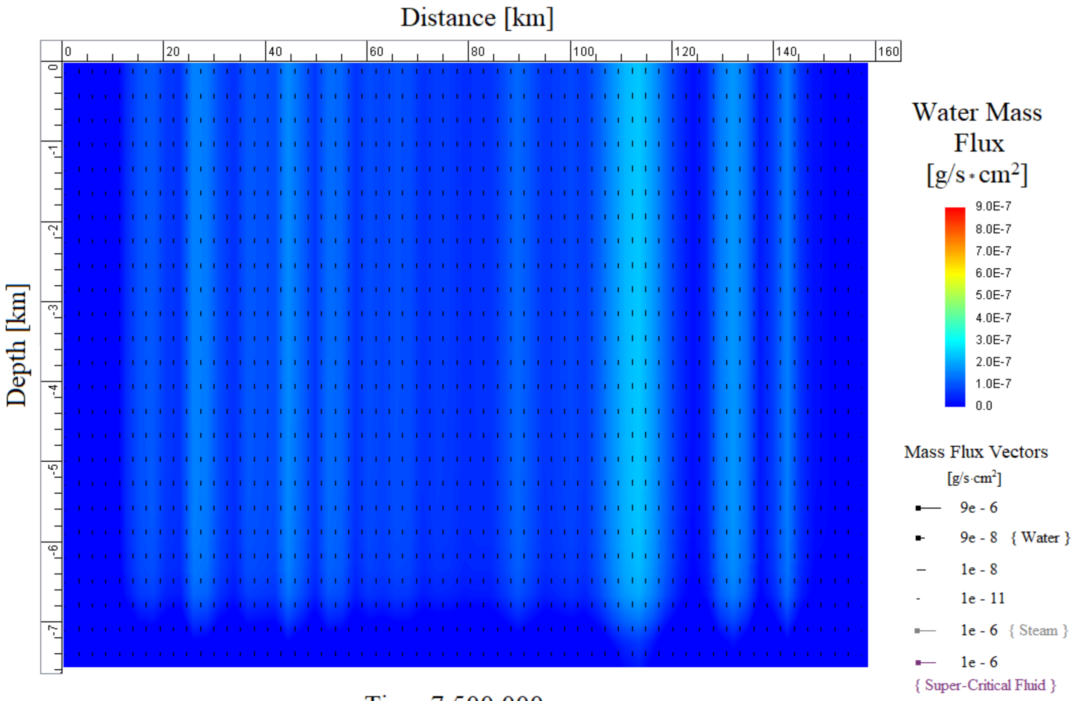

Time: 7.500.000 years

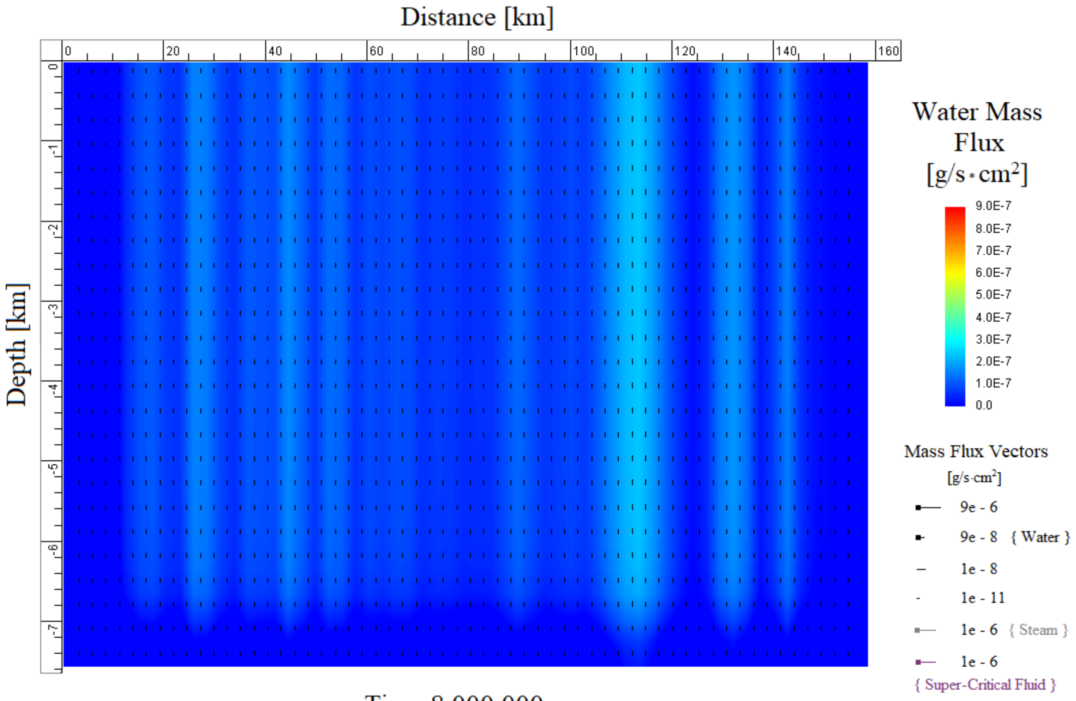

Time: 8.000.000 years

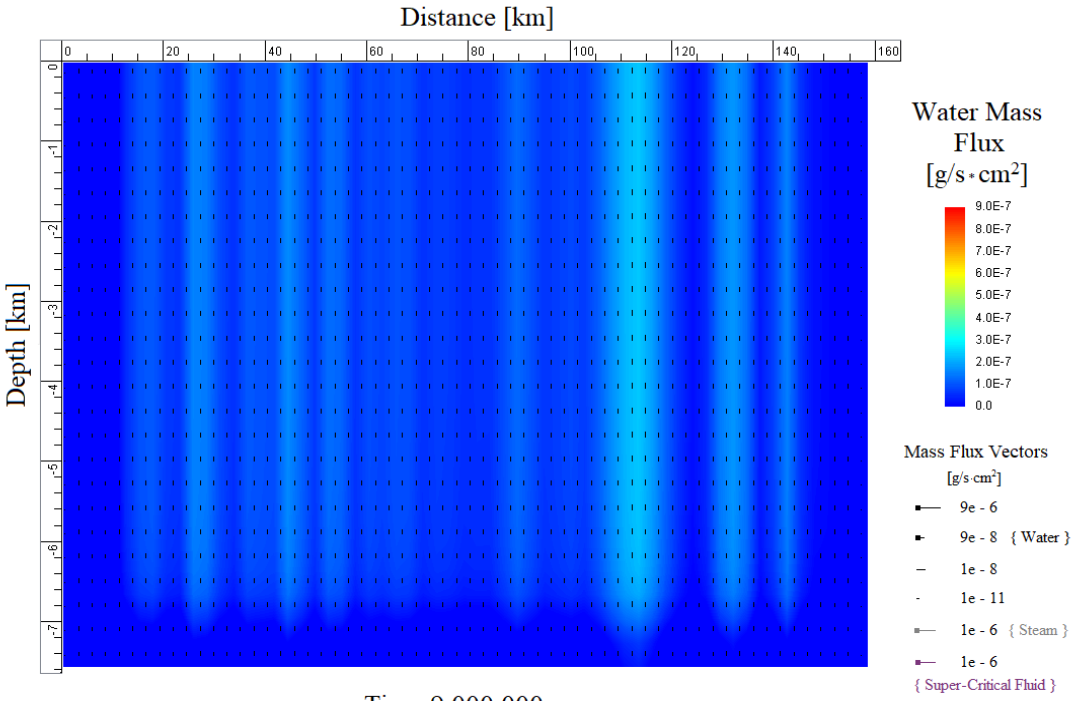

Time: 9.000.000 years

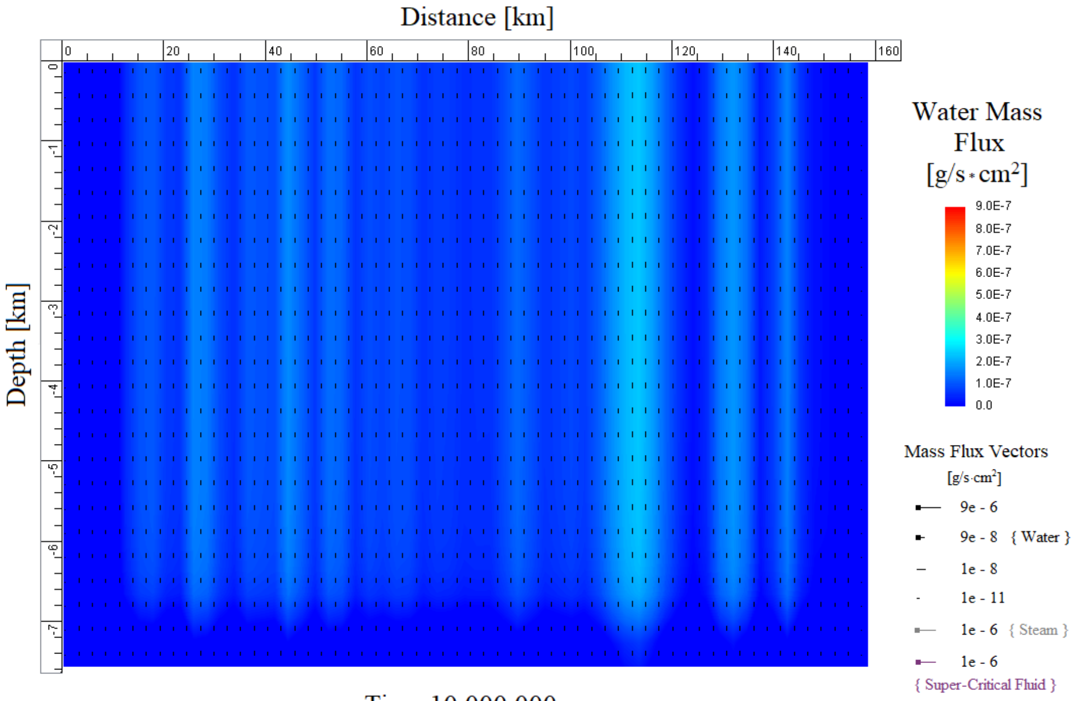

Time: 10.000.000 years

The first time step at 2 Kyr demonstrates that the central melt sheet has a peripheral lower peak temperature T = 1100°C, compared to the initial temperature of 1200°C right after the crater formation. The central melt sheet remains extremely hot though, with a peak temperature at its centre still at 1200°C. This cooling by 100°C suggests that melting of the peripheral rock units should have already occurred within the first 2 Kyr. In terms of hydrothermal circulation, at 2 Kyr there is strong water flux through the entire domain with average fluxes of 5.0e–5 $g/s\cdot cm^2$, except for the central melt sheet that is extremely hot and impermeable. By this time, partial flooding of the deepest portions of the crater cavity by seawater might have taken place. This water quickly infiltrated the permeable breccias over the melt, resulting in peak water fluxes within this rock unit and the broader peripheral area. The peak water fluxes are estimated at value of 1.0e–4 $g/s\cdot cm^2$ and are present mainly within the melt rocks, breccias and suevites above the central melt sheet. The central melt sheet and other rock units that are within a thermal range of 600-1200°C remain impermeable. Stronger water flow is also observed within the brecciated Cretaceous limestones that are in contact with the central melt sheet overheated zone, down to a depth of 4 km in this simulation (which is not meant to represent exact crater structure). The upper boundary donates the replacement fluid to the system and as water flows through the breccia and reaches the deepest peripheral area of the central melt, steam is also generated near the critical point of water at 374°C. At the time-step of 20 Kyr the system is significantly cooler, as the central melt sheet has a peak temperature of 900°C, but it still remains molten. The hydrothermal flow is by an order of magnitude weaker in most of the setting, but the maximum water mass fluxes are preserved within and beneath the peak ring and above the central melt sheet. We can observe that the temperature contours within the breccias, those in the vicinity of the breccia - central melt boundary and the breccia - limestone boundary (along the central crater basin, peak ring and rim) are moving towards the near surface. These are significant deflections in the temperature contours that are controlled by the permeability of the rock units and their thermodynamic state. Permeability decreases exponentially with depth and the central parts of the crater are impermeable due to the high temperatures, relegating most hydrothermal cooling to near-surface regions, while the bulk of the crater is dominated by conduction. Because the numerical code expresses permeability as a function of temperature, this explains the response of the central melt sheet temperature contours and the water flux conditions within the suevite-breccia, which is inconsistent with measured permeabilities of the sorted suevite[14] again suggesting this extended duration model is actually conservative. Moreover, the suevite breccias have a 2-3 orders of magnitude higher permeability than the granitoids and the melt rocks which is also inconsistent at least for the granitoids implying exact patterns of flow are not constrained but a hydrothermal systems extending to at least as long as 6-9 Myr is attainable.

After 200 Kyr the central melt sheet has partially crystallized in our conservative simulation. Most of the hydrothermal activity takes place above the central melt sheet - breccia boundary and close to the

inner slopes of the peak ring. The domain of the central melt sheet after 200 Kyr is now permeable and the melt rocks allow water to flow through it. From 20 to 200 Kyr we observe that the impermeable zone is moving deeper and is spatially reduced as it follows the thermal evolution of the setting (also seen at the temperature figures from 20 to 200 Kyr). The maximum water fluxes at 200 Kyr are by a factor of 2 smaller than those seen at 20 Kyr, but these values nevertheless imply that the hydrothermal fluid - suevite reactions are still ongoing. During the period from 20 to 250 Kyr we observe from the water mass flux output figures that the hydrothermal fluid is recharged, probably by seawater that penetrates and flows through the impactites and down to the breccia – limestone - central melt boundary. Quite interestingly, this process creates other smaller circulation cells within the hydrothermal system. These cells have an amazingly high periodic occurrence, as they are generated every 2 Ky in the simulations. Their lifetime though is no greater than 2-4 Kyr and they have a higher occurrence within the period from 250 Kyr - 4 Myr. Through the output of the water mass flux these phenomena can be recognized as instantaneous flares of cold water recharge. In conclusion, this process suggests that the hydrothermal fluid is constantly recharged with fresh seawater that gradually boosts the hydrothermal cooling process. The hydrothermal cooling and the weakening of the water mass flow are represented quite clearly via the water mass flux from 500 Kyr to 7 Myr. During the period from 2 to 7 Myr the system's maximum water mass flux is almost two orders of magnitude smaller and the hydrothermal circulation is preserved by several small convective cells. At 2-3 Myr, it is quite noticeable that two circulation cells are active within a depth of 1.5 km beneath the peak ring. At 5 Myr, the hydrothermal cells that have endured are easily distinguishable. The major cells appear mainly beneath the peak ring and especially in the outer rim and inner basin areas closer to the peak ring. The simulations suggest a much stronger hydrothermal flow to have taken place within the peak ring regions; a logical explanation is that the hydrothermal flow in the peak ring persisted for a longer period of time therein than in any other region of the crater and hence, this may have resulted in a much more rapid alteration of the melt rocks and the formation of clays in the peak ring. The eastern convective cell appears to expand its activity to a greater depth than the western cell, and this is seen at the water mass flux output from 3 to 10 Myr. This can be initially considered an artefact of deterministic modelling; but in these sets of experimental simulations the mass amount of melt rocks and granitoids that were raised by the impact to a shallower depth is modelled higher to the eastern region beneath the peak ring than the western domain and hence, the thermal properties of the rocks therein allow the hydrothermal fluid to maintain its velocity and vigour for longer. For experimentation purposes, asymmetry on the spatial distribution of the rock units and a higher anisotropy via fracturing were added to the eastern domain beneath the peak ring without an effort to make any given body match geophysical observations. Rather this construct allows us to show and compare the chaotic response of several regions of the same simulated geological setting after minor changes on the spatial distribution of fractures and the geometrical characteristics of some small chunks of the main lithological units. These changes on the spatial distribution of the rock units

and their fractures resulted in a different circulation pattern in some of the eastern domains of the setting, proving that deterministic modelling of such systems is an utterly complex scientific process and suggests more geophysically constraind detailed model construction would be appropriate in future studies. This approach is also consistent in general with the anisotropy observed in all natural systems and in particular with the observations and studies of all of the retrieved drilled cores from the Chicxulub crater.

Finally, after 6 Myr the thermal state of the hydrothermal system is defined by a maximum temperature of the domain no greater than 400°C at a depth of 7 km and the maximum water fluxes are now by an order of magnitude smaller. There is still a long-lived but weak (water mass flux $\leq$ 1.0e-8 $g/s \cdot cm^2$) water flow above the central melt - breccia boundary, the peak ring and within the impact-breccias in general. The system cools completely at 6.5 Myr, with peak temperatures at 170°C, down to a depth of 3.0 km, implying that the temperatures have returned close to the present geothermal gradient. At 7 Myr the system still has a few persistent flows, most notably within the suevite-breccias. This convection pattern may persist for a longer time or may even be non-existent, due to fracture closing by hydrothermal mineralization and clay deposition processes.

References

1. Renne, P. R., Sharp, Z. D. & Heizler, M. T. Cl-derived argon isotope production in the CLICIT facility of OSTR reactor and the effects of the Cl-correction in $^{40}Ar/^{39}Ar$ geochronology. *Chemical Geology* https://doi.org/10.1016/j.chemgeo.2008.07.014 (2008) doi:10.1016/j.chemgeo.2008.07.014.
2. Renne, P. R., Knight, K. B., Nomade, S., Leung, K. N. & Lou, T. P. Application of deuteron-deuteron (D-D) fusion neutrons to $^{40}Ar/^{39}Ar$ geochronology. *Applied Radiation and Isotopes* https://doi.org/10.1016/j.apradiso.2004.06.004 (2005) doi:10.1016/j.apradiso.2004.06.004.
3. Renne, P. R., Balco, G., Ludwig, K. R., Mundil, R. & Min, K. Response to the comment by W.H. Schwarz et al. on 'Joint determination of ^{40}K decay constants and $^{40}Ar^*/^{40}K$ for the Fish Canyon sanidine standard, and improved accuracy for $^{40}Ar/^{39}Ar$ geochronology'. *Geochimica et Cosmochimica Acta* **75**, 5097–5100 (2011).
4. Renne, P. R. & Norman, E. B. Determination of the half-life of ^{37}Ar by mass spectrometry. *Physical Review C - Nuclear Physics* **63**, 473021–473023 (2001).

5. Stoenner, R. W., Schaeffer, O. A. & Katcoff, S. Half-lives of Argon-37, Argon-39, and Argon-42. *Science* **148**, 1325–1328 (1965).
6. Lee, J. Y. *et al.* A redetermination of the isotopic abundances of atmospheric Ar. *Geochimica et Cosmochimica Acta* **70**, 4507–4512 (2006).
7. Steiger, R. H. & Jäger, E. Subcommission on geochronology: convention on the use of decay constants in geo- and cosmochronology. *Earth and Planetary Science Letters* **36**, 359–362 (1977).
8. Tremblay, M. M., Shuster, D. L. & Balco, G. Diffusion kinetics of ^{3}He and ^{21}Ne in quartz and implications for cosmogenic noble gas paleothermometry. *Geochimica et Cosmochimica Acta* **142**, 186–204 (2014).
9. Kipp, K. L., Hsieh, P. A. & Charlton, S. R. *Guide to the Revised Ground-Water Flow and Heat Transport Simulator: HYDROTHERM - Version 3*. *Techniques and Methods* (2008).
10. Hayba, D. O. & Ingebritsen, S. E. *The Computer Model Hydrotherm, a Three-Dimensional Finite-Difference Model to Simulate Ground-Water Flow and Heat Transport in the Temperature Range of 0 to 1,200 Degrees C*. *Water-Resources Investigations Report* (1994).
11. Abramov, O. & Kring, D. A. Numerical modeling of impact-induced hydrothermal activity at the Chicxulub crater. *Meteoritics and Planetary Science* **42**, 93–112 (2007).
12. Yang, J., Latychev, K. & Edwards, R. N. Numerical computation of hydrothermal fluid circulation in fractured Earth structures. *Geophys. J. Int.* **135**, 627–649 (1998).
13. Vujević, K., Graf, T., Simmons, C. T. & Werner, A. D. Impact of fracture network geometry on free convective flow patterns. *Advances in Water Resources* **71**, 65–80 (2014).
14. Le Ber, E. *et al.* Petrophysics of Chicxulub Impact Crater's Peak Ring. *JGR Solid Earth* **127**, e2021JB023801 (2022).
15. Garcia, D. D., Leite, E. P., Vasconcelos, M. A. R., Christou, E. & Crósta, A. P. Distribution of gamma-ray elements in Cerro do Jarau impact structure and a proposal of geothermal modeling. *An. Acad. Bras. Ciênc.* **96**, e20230005 (2024).

16. Alsemgeest, J., Christou, E. & Brouwer, F. M. Evolution of impact-generated hydrothermal systems in basaltic targets on Earth and implications for habitats on Mars. *Icarus* **417**, 116140 (2024).
17. Alexander, A. M., Marchi, S., Johnson, B. C., Wiggins, S. E. & Kring, D. A. Impact-Generated Fragmentation, Porosity, and Permeability Within the Chicxulub Impact Structure. *Earth and Space Science* **11**, e2023EA003383 (2024).
18. Christeson, G. L. *et al.* Extraordinary rocks from the peak ring of the Chicxulub impact crater: P-wave velocity, density, and porosity measurements from IODP/ICDP Expedition 364. *Earth and Planetary Science Letters* **495**, 1–11 (2018).
19. Naumov, M. V. Principal features of impact-generated hydrothermal circulation systems: Mineralogical and geochemical evidence. *Geofluids* **5**, 165–184 (2005).
20. Melosh, H. J. *Impact Cratering: A Geologic Process*. (Oxford University Press, New York, 1989).
21. Morgan, J. V. *et al.* The formation of peak rings in large impact craters. *Science* **354**, 878–882 (2016).
22. Zürcher, L. & Kring, D. A. Hydrothermal alteration in the core of the Yaxcopoil-1 borehole, Chicxulub impact structure, Mexico. *Meteoritics and Planetary Science* **39**, 1199–1221 (2004).
23. Mayr, S. I. *et al.* Integrated interpretation of physical properties of rocks of the borehole Yaxcopoil-1 (Chicxulub impact structure). *J. Geophys. Res.* **113**, 2007JB005420 (2008).
24. Popov, Y. *et al.* Thermal properties of rocks of the borehole Yaxcopoil-1 (Impact Crater Chicxulub, Mexico). *Geophysical Journal International* **184**, 729–745 (2011).
25. Vermeesch, P. M. & Morgan, J. V. Chicxulub central crater structure: Initial results from physical property measurements and combined velocity and gravity modeling. *Meteoritics and Planetary Science* **39**, 1019–1034 (2004).
26. Lamur, A. *et al.* The permeability of fractured rocks in pressurised volcanic and geothermal systems. *Scientific Reports* **7**, 1–9 (2017).

27. Connolly, J. A. D., Schmidt, M. W., Solferino, G. & Bagdassarov, N. Permeability of asthenospheric mantle and melt extraction rates at mid-ocean ridges. *Nature* **462**, 209–212 (2009).

28. Parnell, J., Taylor, C. W., Thackrey, S., Osinski, G. R. & Lee, P. Permeability data for impact breccias. in *40th Lunar and Planetary Science Conference* (2009).

29. Christeson, G. L., Morgan, J. V. & Gulick, S. P. S. Mapping the Chicxulub Impact Stratigraphy and Peak Ring Using Drilling and Seismic Data. *JGR Planets* **126**, e2021JE006938 (2021).

30. Gulick, S. P. S. *et al.* GEOPHYSICAL CHARACTERIZATION OF THE CHICXULUB IMPACT CRATER. *Reviews of Geophysics* **51**, 31–52 (2013).

31. Cockell, C. S. *et al.* Shaping of the Present-Day Deep Biosphere at Chicxulub by the Impact Catastrophe That Ended the Cretaceous. *Front. Microbiol.* **12**, 668240 (2021).

32. Kring, D. A. & Boynton, W. V. Petrogenesis of an augite-bearing melt rock in the Chicxulub structure and its relationship to K/T impact spherules in Haiti. *Nature* **358**, 141–144 (1992).

33. Kring, D. A. Environmental consequences of impact cratering events as a function of ambient conditions on Earth. *Astrobiology* **3**, 133–152 (2003).

34. Kring, D. A. *et al.* Probing the hydrothermal system of the Chicxulub impact crater. *Science Advances* **6**, 1–10 (2020).

35. Onorato, P. I. K., Uhlmann, D. R. & Simonds, C. H. The thermal history of the Manicouagan Impact Melt Sheet, Quebec. *J. Geophys. Res.* **83**, 2789–2798 (1978).

36. Caudill, C. *et al.* Origin of the degassing pipes at the Ries impact structure and implications for impact-induced alteration on Mars and other planetary bodies. *Meteorit & Planetary Scien* **56**, 404–422 (2021).

37. Abramov, O. & Kring, D. A. Numerical modeling of an impact-induced hydrothermal system at the Sudbury crater. *J. Geophys. Res.* **109**, 2003JE002213 (2004).

38. Abramov, O. & Kring, D. A. Impact-induced hydrothermal activity on early Mars. *J. Geophys. Res.* **110**, 2005JE002453 (2005).

39. Fournier, R. O. The transition from hydrostatic to greater than hydrostatic fluid pressure in presently active continental hydrothermal systems in crystalline rock. *Geophys. Res. Lett.* **18**, 955–958 (1991).
