# Supplementary material for: A long-lived impact-generated hydrothermal system at the Chicxulub impact structure
Source: Commun Earth Environ. 2026 Jun 9;7(1):470. doi: 10.1038/s43247-026-03618-5 (PMC13249567; doi:10.1038/s43247-026-03618-5)
Supplement: Supplementary file 1 — Transparent Peer Review file [file 43247_2026_3618_MOESM1_ESM.pdf]

nature portfolio

Peer Review File

A long-lived impact-generated hydrothermal system at the Chicxulub impact structure

Corresponding Author: Dr Annemarie Pickersgill

This file contains all editorial decision letters in order by version, followed by all author rebuttals in order by version.

A version of this paper was originally rejected for publication by Communications Earth & Environment, however that decision was reconsidered after appeal by the authors.

Version 0:

Decision Letter:

Dear Dr Pickersgill,

Please allow us to apologise for the delay in sending a decision on your manuscript titled "Critical constraints on a cradle of life: duration of hydrothermal activity at Chicxulub". It has now been seen by 3 reviewers, whose comments are appended below. In the light of their advice we regret to inform you that we cannot publish your manuscript in Communications Earth & Environment.

Whilst we recognise that the majority of the concerns from Reviewers #1 and #3 could likely be addressed through revision, you will see that Reviewer #2 raises a fundamental concern over the validity of your Ar data which likely invalidates some key results in the paper. Taking these points together with our editorial considerations, we are unable to be confident that your manuscript represents a sufficiently compelling advance over the body of related work in the literature. Unfortunately, these reservations are sufficiently important to preclude publication of this study in Communications Earth & Environment.

We are sorry that we cannot be more positive on this occasion and thank you for the opportunity to consider your work.

Best regards,

Holly Stein, PhD
Editorial Board Member
Communications Earth & Environment
orcid.org/0000-0002-9709-7165

Joe Aslin
Deputy Editor
Communications Earth & Environment

Reviewers' comments:

Reviewer #1 (Remarks to the Author):

This is an interesting generally well-written manuscript. The results are of broad interest and help to understand what happened at the Chicxulub impact structure after the impact event, and as this is one of the largest impact structures preserved on Earth, this is of general interest.

The data are interesting and appear to be of high quality.

Figures and tables are fine.

I have a couple of general requests/comments, and one specific one about the references.

The general comments first: it is not entirely clear from the manuscript which previous data and interpretations led to a much shorter lifetime of a post-impact hydrothermal system - it sounds from some sentences that these estimates are just based on modeling? But there were plenty of earlier petrographic observations, too. And, second, I am missing a more detailed discussion of the possibility that the Ar-ages are influenced by diffusion and other post-mineral-formation alteration effects. How can this be excluded or at least constrained?

And the references are really very full of self-citations, excessively so. There are other publications on hydrothermal systems and alterations at impact structures, not just those by always the same authors (such as 9, 10, 11, 14, 24, 25, 29...etc). Relevant examples of other paper would be:

https://doi.org/10.1016/j.icarus.2024.116140

Rowe et al.: https://doi.org/10.1111/j.1945-5100.2004.tb01138.x

Marchi et al., 2024, doi.org/10.1029/2023EA003065.

Naumov: https://link.springer.com/chapter/10.1007/978-3-662-05010-1_6

Arp et al.: https://doi.org/10.1016/j.icarus.2024.116140
Boer et al. https://doi.org/10.1130/0-8137-2302-7.377
McCarville: https://doi.org/10.1130/0-8137-2302-7.347

and several others...
A bit more diversity in referencing would be welcome...

So in summary I recommend minor revision.
Best regards,
Christian Koeberl (University of Vienna)

Reviewer #2 (Remarks to the Author):

Pickersgill et al present 40Ar/39Ar incremental heating data and numerical simulations from impact melt rocks from three levels within the Chicxulub impact crater. Based on these data, they suggest that the post-impact hydrothermal activity lasted for 8 million years which is 3 times longer than previous estimates.

The beginning of the manuscript skips some basics of the scientific method. There is not real introduction to the problem. Are there conflicting published datasets? What hypotheses are being tested and why? Its almost as if they generated the data and just fit the story to it. What was the motivation for this study in the first place? It is not clear as written. Was it to assess the duration of hydrothermal activity because one study said it was short and other indicated it might be very long? Were they attempting to compare it to hydrothermal activity at other large impacts?

There is one huge fundamental flaw in the manuscript and frankly I'm shocked that the very experienced coauthors did not consider this. The authors suggest that "A key observation from modelling our diffusion experiment results is that the more retentive mineral phase also dominates the budget of radiogenic Ar in our samples….We infer that this retentive phase is the K-rich feldspar overgrowths." Moreover, they suggest that the closure temperature for the K-feldspar overgrowths is 175-210°C. However, these samples were all irradiated at Oregon State CLICIT facility where the temperature during irradiation gets to 230-240 °C. So, all of their data and interpretations related to authigenic K-feldspar cannot be valid because the K-feldspar got above its closure temperature in the reactor and would have been reset. Nothing can be said about the duration of authigenic K-rich feldspar growth and water flux through the system, etc. Unfortunately, any constraints on the duration of hydrothermal activity based on these experiments are also invalid. A more likely explanation is that these data indeed represent subsequent regional geologic/alteration processes that could have induced post-impact argon loss in the plagioclase in these samples, which is exactly what they said is not happening on lines 122-123.

Another point that is mentioned multiple times in the text that is extremely misleading is that the authors suggest that the large Chicxulub impact is an analog for impacts in the Early earth solar system. It also repeatedly says that these early Earth impacts could have created habitable environments for life. Yet they provide no published data or observations (because there aren't any), which indicate that life existed in these early Earth impacts. All this text including the title, which correlates the Chicxulub impact to early Earth life is not warranted.

In sum, due to some major issues, this manuscript should not be considered further for publication in this journal.

Reviewer #3 (Remarks to the Author):

This manuscript presents new constraints on the duration of hydrothermal activity at the Chicxulub impact crater. This is accomplished by presenting new 40Ar/39Ar data for four samples of impact melt rock from the Chicxulub drill core, carrying out diffusion experiments to characterize the Ar diffusivity of the samples, and then performing numerical simulations to evaluate the boundary conditions required for extended hydrothermal activity. The authors conclude that hydrothermal activity continued for at least 6 Myr post-impact, which is three times longer than previously thought.

Overall, the conclusion of extended hydrothermal activity at Chicxulub is solid and is well supported by both the data and the modeling results. I appreciate the integration of different approaches in this study. I provide some suggestions below that I believe would improve the clarity, writing, and presentation of the results.

I am a geochronologist and not a modeler, so I defer to others to provide a rigorous review of the numerical simulations in this manuscript.

Line numbers are missing. Their addition would make it much easier to refer to different parts of the text in my review below.

The manuscript should be more specific in the introductory section and in the discussion about what is already known about the duration of hydrothermal activity in impact settings.
• What duration has been inferred previously at Chicxulub?
• What duration has been inferred previously at other impact craters?
• What data were those previous interpretations based on?
• The opening paragraph states that hydrothermal activity continued for three times longer than previously thought. Three times longer than previously thought at impact craters in general? Or just at Chicxulub? If a 6-8 Myr duration of hydrothermal activity has been inferred at other impact craters previously, then this work is much less significant than if the previously documented duration at other impact craters is only 2 Myr. This is not clear in the current text.

Petrography section.
• The Petrography section is a mix of summarizing the analyzed samples, the petrology, and the methods used to separate material for Ar analysis. “Petrography” is not an accurate header for this section. I suggest using a different header.
• This section would likely flow more easily by first describing the samples and core depths, then the sample petrography, then the relationships between the plagioclase and K-feldspar, and then the attempts to separate different materials. This content is somewhat mixed together in the paragraphs.
• When I finished the Petrography section, the main point was not clear to me. There is a key sentence in the Radio-isotopic analysis section that describes the key conclusion of the petrologic observations. This sentence would be better placed in the Petrography section: “Petrography reveals the order of crystallization of K-bearing phases: plagioclase formed by quenching of the impact melt and later K-rich feldspar overgrowths grew during hydrothermal activity.”
• The third paragraph starts with “The swallow tail and skeletal plagioclase textures indicate…” Please clarify if these textures were present in all samples.
• The final “sentence” in the Petrography section is not actually a complete sentence. “Also veins of K-rich feldspar and albite…”

Radio-isotopic analysis
• For each sample, please be clearer what was thought to be analyzed. Plagioclase? K-feldspar overgrowths? Why? This information is difficult to follow between the petrography and results section.
• “40Ar/39Ar geochronology” would be a more specific header for this section.
• Suggest combining the second and third paragraphs of this section. Both paragraphs describe the 22 plateau ages from the 3 samples.
• In the 6th paragraph of this section, the authors interpret the “two youngest ages to be a result of imperfect separation of sample CHX 756.0 into two distinct lithologies…”. Please explain in the text why the two youngest ages are interpreted to be erroneously young due to imperfect separation. Are these youngest ages included in Figure 2A and 2B? I thought the younger ages were interpreted as due to protracted hydrothermal activity? Why would some young ages be attributed to hydrothermal activity, while others are interpreted as too young to be due to this effect? This section is not clear.

The discussion emphasizes that hydrothermal activity lasted at least 6.0-9.6 Myr.
• Where does 9.6 Myr come from? The Ar dates emphasized in the text and Figure 1 range from 58.3 to 66.2 Myr. This is an 8 Myr duration. Not 9.6 Myr.
• Where does the 6 Myr come from?

An enormous range of geothermal gradients was adopted in the impact simulations, ranging from 13 °C/km to 70 °C/km. Why was 13 °C/km adopted for one of the geothermal gradients? Abramov & Kring is cited here, but some explanation is needed.

Figure 1. The different components of this figure are effective in progressively moving from the crater-scale to the lithologic column-scale to the sample scale. In B) it would be helpful to add dashed lines between the lithologic column on the left and the zoomed in section on the right, to be clearer where in the section that the samples are from.

Figure 2. Caption states that the 66 Ma to 58 Ma age span affects all three samples, but it doesn’t. CHX-756.0A shows a

more limited range.

Data tables
• The Ar data tables and associated information should follow the reporting guidelines of Schaen et al. 2021 (GSAB). That manuscript includes dozens of authors and reaches agreement on reporting recommendations. I think that some of the data that should be reported are missing from the tables? Unless I missed that information in the supplementary files somewhere.

• I also believe that additional information is needed in the diffusion experiment tables C1-C2 to align with recent reporting recommendations. The table should include ln(D/a2) and uncertainties. The references for and all parameters used in the calculation of ln(D/a2) should be explained.

Version 1:

Decision Letter:

** Please ensure you delete the link to your author home page in this e-mail if you wish to forward it to your coauthors **

Dear Dr Pickersgill,

Please allow us to apologise for the delay in sending a decison on your manuscript titled "Critical constraints on a cradle of life: duration of hydrothermal activity at Chicxulub". It has now been seen by our reviewers, whose comments appear below. In light of their advice we are delighted to say that we are happy, in principle, to publish a suitably revised version in Communications Earth & Environment.

We therefore invite you to revise your paper one last time to address the remaining concerns of our reviewers. With respect to the concerns from Reviewer #2, we feel you may retain your interpretations, so long as you are clear where your interpretations are more speculative and where they are based on your direct results. At the same time we ask that you edit your manuscript to comply with our format requirements and to maximise the accessibility and therefore the impact of your work.

EDITORIAL REQUESTS:

Please review our specific editorial comments and requests regarding your manuscript in the attached "Editorial Requests Table".

*****Please take care to match our formatting and policy requirements. We will check revised manuscript and return manuscripts that do not comply. Such requests will lead to delays. *****

Please outline your response to each request in the right hand column. Please upload the completed table with your manuscript files as a Related Manuscript file.

If you have any questions or concerns about any of our requests, please do not hesitate to contact me.

SUBMISSION INFORMATION:

In order to accept your paper, we require the files listed at the end of the Editorial Requests Table; the list of required files is also available at https://www.nature.com/documents/commsj-file-checklist.pdf .

OPEN ACCESS:

Communications Earth & Environment is a fully open access journal. Articles are made freely accessible on publication. For further information about article processing charges, open access funding, and advice and support from Nature Portfolio, please visit https://www.nature.com/commsenv/open-access

At acceptance, you will be provided with instructions for completing the open access licence agreement on behalf of all authors. This grants us the necessary permissions to publish your paper. Additionally, you will be asked to declare that all required third party permissions have been obtained, and to provide billing information in order to pay the article-processing charge (APC).

Please use the following link to submit the above items:
Link Redacted

** This url links to your confidential home page and associated information about manuscripts you may have submitted or be reviewing for us. If you wish to forward this email to co-authors, please delete the link to your homepage first **

We hope to hear from you within two weeks; please let us know if you need more time.

Best regards,

Holly Stein
Editorial Board Member
Communications Earth & Environment

Joe Aslin
Deputy Editor,
Communications Earth & Environment
Consulting Editor,
Communications Sustainability

https://www.nature.com/commsenv/
Twitter: @CommsEarth

REVIEWERS' COMMENTS:

Reviewer #1 (Remarks to the Author):

The revised version is certainly improved compared to the earlier initial version. Most review comments were properly addressed. In my view the manuscript is now ready to be accepted.
Best regards, Christian Koeberl (Univ. Vienna)

Reviewer #2 (Remarks to the Author):

The authors have done a nice job responding to most the three reviewers' criticisms regarding the structure/organization of the manuscript and the questions/issues regarding the geochronologic data and interpretations. The substantial reorganization, especially of the Introduction, now provides much more context for the study. The only issue that now remains outstanding is that the authors continue to insist on correlating the post-Chicxulub impact hydrothermal conditions to those on the early Earth. In their response, the authors have correctly stated that other published papers have already proposed post-impact hydrothermal activity as a mechanism for creating habitable environments for thermophilic and hyperthermophilic microorganisms and generating cradles for prebiotic chemical reactions. Those inferences are exactly that. They are inferences that are not based on data. The continued attempts to correlate Chicxulub hydrothermal activity to that in the early Earth comes across as a desperate attempt to make the manuscript have more curb appeal. It is not needed. The extended duration of hydrothermal activity at Chicxulub is a very interesting finding that can stand on its own and it doesn't need to be stretched to the early Earth. Moreover, the maturity and structure of the Hadean crustal system is still an unknown and it is not clear if hydrothermal activity could be substantiated for a period as long as Chicxulub. Thus, the early Earth correlations on lines 27-33, 270-271, and 355-361 should be removed prior to publication.

Reviewer #3 (Remarks to the Author):

The authors have thoroughly addressed my comments and I consider the manucript ready for publication.

Rebecca Flowers
University of Colorado Boulder

** Visit Nature Portfolio's author and referees' website at www.nature.com/authors for information about policies, services and author benefits**

Open Access This Peer Review File is licensed under a Creative Commons Attribution 4.0 International License, which permits use, sharing, adaptation, distribution and reproduction in any medium or format, as long as you give appropriate credit to the original author(s) and the source, provide a link to the Creative Commons license, and indicate if changes were made.
In cases where reviewers are anonymous, credit should be given to 'Anonymous Referee' and the source.
The images or other third party material in this Peer Review File are included in the article's Creative Commons license, unless indicated otherwise in a credit line to the material. If material is not included in the article's Creative Commons license and your intended use is not permitted by statutory regulation or exceeds the permitted use, you will need to obtain permission directly from the copyright holder.
To view a copy of this license, visit https://creativecommons.org/licenses/by/4.0/

Thank you for your response to our manuscript and for the thoughtful reviews. My coauthors and I have worked to address all reviewer concerns in the attached manuscript with tracked changes, and in the point-by-point responses below.

Reviewer #1 (Remarks to the Author):

This is an interesting generally well-written manuscript. The results are of broad interest and help to understand what happened at the Chicxulub impact structure after the impact event, and as this is one of the largest impact structures preserved on Earth, this is of general interest.
The data are interesting and appear to be of high quality.
Figures and tables are fine.
I have a couple of general requests/comments, and one specific one about the references.
The general comments first: it is not entirely clear from the manuscript which previous data and interpretations led to a much shorter lifetime of a post-impact hydrothermal system - it sounds from some sentences that these estimates are just based on modeling? But there were plenty of earlier petrographic observations, too.

> The previous estimates of duration largely come from numerical simulations, with some constraints provided by palaeomagnetic and petrographic work. We have added an expanded discussion about the previous estimates of duration in the section "*Hydrothermal System at Chicxulub*" (lines 68-94)

And, second, I am missing a more detailed discussion of the possibility that the Ar-ages are influenced by diffusion and other post-mineral-formation alteration effects. How can this be excluded or at least constrained?

> We have considered other possible heat sources than the Chicxulub impact and have moved text discussing the chronologically and geographically nearest events from Supplement E into the main text (see subsection "*Other Geological Events*" – lines 314-343).
>
> Post-formation alteration was addressed in the sample preparation phase during which samples were leached ultrasonically in nitric acid and subsequently rinsed until water ran clean. Samples selected for $^{40}Ar/^{39}Ar$ analysis were handpicked to select the cleanest grains. Details of sample preparation are in the Methods section under "$^{40}Ar/^{39}Ar$ analyses – Sample preparation" (lines 397-433). We were able to confirm that this sample preparation technique had been successful by examining the low-temperature steps in the age spectra plots. Had the younger alteration phases still been present, they would have dominated the low-temperature steps (argon released from low-retentivity sites) and yielded younger ages at the start of the step-heating plot. We have added the following text to address this issue (lines 337-343):

"Additionally, neither diffusion nor step-heating experiments indicate substantial diffusive loss of ^{40}Ar from low-retentivity domains, which would manifest as younger apparent step ages during the initial stages of step degassing, with ages increasing with increasing ^{39}Ar release fraction. Diffusion experiment results indicate quantitative argon retention below 150-225 ºC on geologic timescales (e.g., Figures C-3 and C-4), temperatures which we do not expect the samples to have exceeded during their geologic history after hydrothermal activity at Chicxulub ceased."

And the references are really very full of self-citations, excessively so. There are other publications on hydrothermal systems and alterations at impact structures, not just those by always the same authors (such as 9, 10, 11, 14, 24, 25, 29...etc).
Relevant examples of other paper would be:
Alsemgeest et al 2024: https://doi.org/10.1016/j.icarus.2024.116140
Rowe et al.: https://doi.org/10.1111/j.1945-5100.2004.tb01138.x
Marchi et al., 2024, doi.org/10.1029/2023EA003065.
Naumov: https://link.springer.com/chapter/10.1007/978-3-662-05010-1_6
Arp et al.: **https://doi.org/10.1111/maps.12235**
Boer et al. https://doi.org/10.1130/0-8137-2302-7.377
McCarville: https://doi.org/10.1130/0-8137-2302-7.347
and several others...
A bit more diversity in referencing would be welcome...

We have moved some of the supplementary text into the main text, thereby expanding the discussion and the reference list.

We have expanded discussion of other estimates of hydrothermal duration to more thoroughly acknowledge authors who have worked on this problem before (lines 95-129).

We have expanded discussion of previous hydrothermal work at Chicxulub (lines 68-94).

Reviewer #2 (Remarks to the Author):

Pickersgill et al present 40Ar/39Ar incremental heating data and numerical simulations from impact melt rocks from three levels within the Chicxulub impact crater. Based on these data, they suggest that the post-impact hydrothermal activity lasted for 8 million years which is 3 times longer than previous estimates.
The beginning of the manuscript skips some basics of the scientific method. There is not real introduction to the problem. Are there conflicting published datasets?

There is existing conflict between modelled vs. measured duration of hydrothermal activity at other impact craters (see discussion in *"Hydrothermal duration at other impact structures"* (lines 95-129) subsection of the manuscript, formerly in Supplement E). There are no currently published radio-isotopic datasets which have attempted to measure the duration of activity at Chicxulub, only numerical simulations (Abramov & Kring 2007, Christou MSc), palaeomagnetic, and petrographic constraints (Kring et al 2020, Rowe et al 2004).

We have expanded the introduction to include text from the supplement discussing what is known about hydrothermal duration at other impact structures. We have also added a section to more clearly describe what is already known about hydrothermal duration at Chicxulub (*"The hydrothermal system at Chicxulub"* lines 68-94).

What hypotheses are being tested and why? Its almost as if they generated the data and just fit the story to it. What was the motivation for this study in the first place? It is not clear as written. Was it to assess the duration of hydrothermal activity because one study said it was short and other indicated it might be very long? Were they attempting to compare it to hydrothermal activity at other large impacts?

We have expanded the introduction to more rigorously set up the problem, and have incorporated some of the supplementary text into the manuscript.

Specifically, we added these sentences (lines 79-83):

> *"Numerical simulations of hydrothermal activity at Chicxulub[33] suggested that it took 1.5 to 2.3 Myr to cool to 90 °C at 1 km below the surface, and it would have taken at least twice as long to cool below the thermophilic window. The authors considered this to be a conservative estimate, and one of the main goals of Expedition 364 was to search for evidence which could empirically ground truth those simulations[22]."*

There is one huge fundamental flaw in the manuscript and frankly I'm shocked that the very experienced coauthors did not consider this. The authors suggest that "A key observation from modelling our diffusion experiment results is that the more retentive mineral phase also dominates the budget of radiogenic Ar in our samples….We infer that this retentive phase is the K-rich feldspar overgrowths." Moreover, they suggest that the closure temperature for the K-feldspar overgrowths is 175-210°C. However, these samples were all irradiated at Oregon State CLICIT facility where the temperature during irradiation gets to 230-240 °C. So, all of their data and interpretations related to authigenic K-feldspar cannot be valid because the K-feldspar got above its closure temperature in the reactor and would have been reset.

This critique relies on the relationship between reactor temperature during irradiation and the concept of closure temperature. In order to demonstrate that the above concern about ^{40}Ar loss during irradiation is unwarranted we would like to further explore argon systematics and diffusion kinetics of our samples to demonstrate why

argon loss during irradiation is not of concern, and to clarify the conditions during irradiation as discussed with reactor staff.

Closure temperature can be a useful concept when applied to systems like $^{40}Ar/^{39}Ar$ because it enables comparison of the thermal sensitivities of different minerals and/or samples. However, the mathematical formulation of closure temperature is highly specific to a set of thermal history conditions: it is typically calculated assuming monotonic cooling at 10 ºC/Ma. It is a common misconception that minerals become “reset” or lose substantial proportions of their argon once they exceed a nominal closure temperature, but this is not true and is inconsistent with noble gas diffusion kinetics. That said, because this is not an uncommon misconception, it is worth addressing more completely.

To assess the potential for radiogenic ^{40}Ar loss during neutron irradiation, we need not rely on the closure temperature concept and can instead directly calculate the expected fractional loss of ^{40}Ar given the diffusion kinetics we obtained from the reactor-produced ^{39}Ar. Consider the worst-case scenario in which our samples were held at 240 ºC, the maximum temperature quoted by Reviewer 2, for 50 hours (we describe in detail below why this worst case scenario is far from the true irradiation conditions and durations). For these conditions, the authigenic K-feldspar in CHX-735.0 would lose only 0.13% of its radiogenic ^{40}Ar from its least retentive diffusion domain; the more retentive domains of the K-feldspar in CHX-735.0, which constitute the majority of the ^{40}Ar budget, would lose even less radiogenic ^{40}Ar. Similarly, for the authigenic K-feldspar in CHX-756.0A, the least retentive diffusion domain will only lose 0.04% of its radiogenic ^{40}Ar if the sample were held at 240 ºC for 50 hours, with other domains (constituting the majority of the ^{40}Ar budget) losing even less. Thus, both CHX-735.0 and CHX-756.0A are expected to lose $<<$ 1% of their radiogenic ^{40}Ar during neutron irradiation in this worst-case scenario, which is, as discussed below, not realistic for the actual irradiation conditions experienced by these samples.

We have communicated directly with staff at the OSU TRIGA Reactor where our samples were irradiated to confirm that temperatures for irradiation setups with the aluminium discs used by SUERC have been measured to reach a maximum of 225 ºC after 3 hours of operation. This temperature was confirmed with measurements in 2009 and 2024, bracketing the time of our irradiations. Though our irradiations were 50 hours in total duration, this duration was broken up into 9 separate irradiation periods of varying duration (4-6 hours, full schedule now included in Appendix B). Each irradiation session will ramp up to full temperature slowly resulting in a timeframe of up to 3 hours a day at an absolute maximum of 225 °C, such that the amount of time spent at 225 ºC would be substantially shorter than the overall irradiation duration – we estimate a maximum of 30 hours total at a maximum of 225 °C. If we do the same calculation as above and instead consider a 30-hour long heating event to 225 ºC (still a worst-case scenario, as the cooling between the 9 irradiation periods would further inhibit ^{40}Ar loss), we would predict 0.06% loss from

the least retentive K-feldspar domain in CHX-735.0 and 0.01% loss from the least retentive K-feldspar domain in CHX-756.0A. As these are the least retentive domains and constitute a minority of the authigenic K-feldspar ^{40}Ar budget, the total potential fractional loss of ^{40}Ar is substantially lower. Therefore, loss of ^{40}Ar during the neutron irradiation is insignificant and cannot explain the observed young apparent $^{40}Ar/^{39}Ar$ dates that we interpret to reflect continued hydrothermal activity for several million years after the Chicxulub impact.

Finally, it is worth noting that the concerns raised by Reviewer 2 about the reactor conditions and the potential for radiogenic ^{40}Ar loss would invalidate all low temperature $^{40}Ar/^{39}Ar$ geochronology. Given the large body of literature validating this method across a large range of geologic settings and minerals, we are confident that this is a valid technique.

In the case of our samples, we can add that they are demonstrably not reset in that they still record ages of ~58-66 Ma. If we consider that substantial argon loss occurred as a result of temperature in the reactor then we would expect to see it as disruption of the age spectra plots themselves in addition to disruption in the diffusion data (we would not get plateaus as low retentivity sites would be degassed during irradiation).

In order to clarify the above issues we have added part of the above discussion to the manuscript at lines 414-431:

> *"The 50-hour irradiation took place over 9 separate irradiation sessions, with each session lasting between 4 and 6 hours (see Supplement B for full irradiation schedule).*
>
> *Samples were heated to a maximum of 225 ºC during irradiation due to interaction with gamma rays. This temperature was confirmed with measurements conducted in 2009 and 2024 by OSU TRIGA staff, bracketing the time of our irradiations. Though our irradiations were 50 hours in total duration, this was broken up into 9 separate irradiation periods of varying lengths, such that the amount of time spent at 225 ºC would be substantially shorter – we estimate a maximum of 30 hours. To assess the potential for radiogenic ^{40}Ar loss during neutron irradiation, we calculated the expected fractional loss of ^{40}Ar given the diffusion kinetics we obtained from the reactor-produced ^{39}Ar. If we consider a 30-hour long heating event to 225 ºC (a worst-case scenario, as the cooling between the 9 irradiation periods would further inhibit ^{40}Ar loss), we predict 0.06% loss from the least retentive K-*

feldspar domain in CHX-7350 and 0.01% loss from the least retentive K-feldspar domain in CHX-7560A. As these are the least retentive domains and constitute a minority of the authigenic K-feldspar ^{40}Ar budget, the total potential fractional loss of ^{40}Ar across all domains is substantially lower. Therefore, loss of ^{40}Ar during the neutron irradiation is insignificant and cannot explain the observed young apparent ^{40}Ar/^{39}Ar dates that we interpret to reflect continued hydrothermal activity for several million years after the Chicxulub impact."

Nothing can be said about the duration of authigenic K-rich feldspar growth and water flux through the system, etc. Unfortunately, any constraints on the duration of hydrothermal activity based on these experiments are also invalid. A more likely explanation is that these data indeed represent subsequent regional geologic/alteration processes that could have induced post-impact argon loss in the plagioclase in these samples, which is exactly what they said is not happening on lines 122-123.

We are confident, as described above, of having justified why nontrivial radiogenic ^{40}Ar loss during irradiation is not plausible.

Regarding geologic processes/events after hydrothermal activity ceased that could have caused diffusive loss of radiogenic ^{40}Ar, we have moved the discussion of "*Other Nearby Geological Events*" from Supplement E into the main text, and expanded discussion about alteration and potential diffusive loss from subsequent geologic events in the main text (lines 314-343).

Another point that is mentioned multiple times in the text that is extremely misleading is that the authors suggest that the large Chicxulub impact is an analog for impacts in the Early earth solar system.

Chicxulub generated initial physical conditions which are expected to be virtually identical to Hadean impacts. Although there may be differences in target rock, it makes sense to start with the best example preserved on Earth of what Hadean impacts would have looked like, i.e., a structure with the same physical properties (controlled by impact) of fracturing and modified porosity and permeability.

The Chicxulub impact is the best preserved peak-ring basin on Earth, which would have been a common structure on early Earth. The impact-induced fracturing and modifications in porosity and permeability are, within the constraints of the model, suitable representations of Hadean impact sites. That is, Chicxulub is a good physical model of that environment. We acknowledge that the effects of target lithologies remain open to discussion. While Hadean zircon implies there were granitic crustal components – like that at Chicxulub – far more mafic crust also existed. Thus, hydrothermal chemical reactions and mineral products will have differed in some

Hadean impact sites, but those differences would have been less consequential for the duration of a hydrothermal system, as modelled here, than the physical properties observed and inherent in a structure like Chicxulub.

The following text was added to lines 52-63:

> *"However, we can investigate some of the key attributes of these structures by studying younger analogues of those very large craters – termed peak-ring basins - that are preserved. The 66 Ma Chicxulub impact structure, Yucatán, México (e.g.,(Kring et al. 2020)) is the best preserved peak-ring basin on Earth, making it a good physical model of Hadean impact sites. The target rocks on early Earth are more likely to have been mafic, so post-impact hydrothermal chemical reactions and mineral products will have differed from those observed at Chicxulub. However, those differences are expected to be less consequential for hydrothermal system duration than the overall physical properties of the structure such as impact-induced fracturing and modifications to porosity and permeability observed at Chicxulub. Additionally, Chicxulub provides a unique sample suite recovered through drill cores, and has not undergone significant post-impact heating or modification unrelated to the impact event."*

It also repeatedly says that these early Earth impacts could have created habitable environments for life. Yet they provide no published data or observations (because there aren't any), which indicate that life existed in these early Earth impacts. All this text including the title, which correlates the Chicxulub impact to early Earth life is not warranted.

Previous work, as well as our current manuscript, demonstrate that impact-generated hydrothermal systems provide *environmental conditions suitable for life*. Moreover, evidence of microbial ecosystems in the geologic past has been noted in previous work. For example, sulfate-sulfide isotopic fractionation indicative of microbial metabolism has been documented in the impact-generated hydrothermal systems of Haughton crater (Parnell et al. 2010), Rochechouart crater (France) (Simpson et al. 2017), and Chicxulub (Kring et al. 2021), the focus of this study. Not only did a microbial ecosystem exist in the Chicxulub hydrothermal system, microbial activity still persists in the subsurface of the crater today (Cockell et al. 2021; Quraish et al. 2024). The metabolic processes in Hadean craters may have been different, but that metabolism and thriving communities *can exist* in such environments has been demonstrated by previous work.

We have expanded the Introduction to more thoroughly cover previous research linking early life on Earth to hydrothermal systems and impact structures. We have attempted to explain the difference between discussing habitability (that a site meets the requirements for supporting microorganisms) and claiming that we have proved inhabitation of early Earth impacts. As discussed in the manuscript, these early Earth impacts have been erased, necessitating that we address questions about them by

studying analogous sites. That is why we study the large impacts from more relatively recent times such as Chicxulub, to understand the processes that were occurring further back in Earth's history.

The hypothesis that impact craters on early Earth and on other planets were habitable environments has been discussed for decades [e.g., (Cockell 2006; Cockell and Lee 2002; Cockell et al. 2003)]. We herein discuss habitability not inhabitation, making no claim that micro-organisms were confirmed to have been living in Hadean impact structures). We acknowledge having no evidence that these early craters were inhabited because these early craters no longer exist, and if they did any biosignatures would most likely have been destroyed over the billions of years since their formation. However, we have substantial evidence that everywhere on Earth in the present day where we have heat and water interacting we have abundant and diverse life (e.g., black smokers, Yellowstone, etc. (e.g. (Maher and Stevenson 1988) (Sleep et al. 1989) (Russell and Hall 1997) (Holm 1992) (Wächtershäuser 1988)).

We have added the following text on lines 15-34:

> *"The impact of asteroids and comets with planetary bodies is one of the most fundamental and widespread geological processes in the solar system(*Melosh 1989; Osinski and Pierazzo 2013*). Once thought of as purely destructive events, interest in large ancient meteorite impact structures has risen due to their potential to have created habitable environments in the first few hundred million years of solar system history(*Abramov and Kring 2004, 2005; Cockell 2006; Cockell and Lee 2002; Farmer 2000; Kring 2000, 2003; Naumov 2005; Osinski et al. 2013, 2020; Rathbun and Squyres 2002; Schwenzer and Kring 2009, 2013*). Hydrothermal systems form anywhere that heat and aqueous fluids interact(*Pirajno 1992*), including within cooling hypervelocity impact craters(*Kring 2003; Kring and Boynton 1992; Naumov 2005; Osinski et al. 2013*). This has been proposed as a mechanism for creating habitable environments for thermophilic and hyperthermophilic micro-organisms(*Cockell and Lee 2002*) and generating cradles for prebiotic chemical reactions(*Cockell 2006; Osinski et al. 2020*).*
>
> *Impact-generated hydrothermal systems are widespread, with evidence of hydrothermal activity found at over 70 of the ~200 impact structures on Earth(*Osinski et al. 2022*). While it has been demonstrated that these environments are habitable via increased porosity, chemistry, and nutrient*

*availability, explicit evidence of microbial colonization in impact craters is harder to find (only 8 of the ~200 impact structures on Earth), and even harder to chronologically link with specific impact-generated processes(*Gustafsson et al. 2025*). We therefore approach the question of whether ancient impacts on Earth could have contributed to the origin of life by examining the windows of opportunity for habitability. While we cannot say with certainty that these early impact environments were inhabited, we can assess whether they provided the right conditions — and for long enough durations — for life to emerge. In this work we address the critical constraint of longevity of the impact generated hydrothermal system in the Chicxulub peak-ring basin as an analogue for early Earth impacts."*

In sum, due to some major issues, this manuscript should not be considered further for publication in this journal.

We hope that we have sufficiently addressed the reviewer's concerns regarding the work presented, thereby allowing for publication.

Reviewer #3 (Remarks to the Author):

This manuscript presents new constraints on the duration of hydrothermal activity at the Chicxulub impact crater. This is accomplished by presenting new 40Ar/39Ar data for four samples of impact melt rock from the Chicxulub drill core, carrying out diffusion experiments to characterize the Ar diffusivity of the samples, and then performing numerical simulations to evaluate the boundary conditions required for extended hydrothermal activity. The authors conclude that hydrothermal activity continued for at least 6 Myr post-impact, which is three times longer than previously thought.
Overall, the conclusion of extended hydrothermal activity at Chicxulub is solid and is well supported by both the data and the modeling results. I appreciate the integration of different approaches in this study. I provide some suggestions below that I believe would improve the clarity, writing, and presentation of the results.
I am a geochronologist and not a modeler, so I defer to others to provide a rigorous review of the numerical simulations in this manuscript.
Line numbers are missing. Their addition would make it much easier to refer to different parts of the text in my review below.

Done

The manuscript should be more specific in the introductory section and in the discussion about what is already known about the duration of hydrothermal activity in impact settings.
• What duration has been inferred previously at Chicxulub?

• What duration has been inferred previously at other impact craters?
• What data were those previous interpretations based on?
• The opening paragraph states that hydrothermal activity continued for three times longer than previously thought. Three times longer than previously thought at impact craters in general? Or just at Chicxulub? If a 6-8 Myr duration of hydrothermal activity has been inferred at other impact craters previously, then this work is much less significant than if the previously documented duration at other impact craters is only 2 Myr. This is not clear in the current text.

What was the opening paragraph is now the abstract. We have added this line to the end (lines137-140):

> *"...*which is approximately four times longer than previously estimated by numerical simulations, palaeomagnetism, and petrographic interpretations at Chicxulub, and is the longest documented duration for an impact structure on Earth*."*

We have additionally moved text from Supplement E into the main text to better describe what is known about hydrothermal duration at other impact structures. See subsection "*Hydrothermal duration at other impact structures*" (lines 91-125).

And have added a section to more thoroughly describe what is already known about the duration of hydrothermal activity at Chicxulub "*Hydrothermal System at Chicxulub"* (lines 64-90).

Petrography section.
• The Petrography section is a mix of summarizing the analyzed samples, the petrology, and the methods used to separate material for Ar analysis. "Petrography" is not an accurate header for this section. I suggest using a different header.

Changed to "Sample description"

• This section would likely flow more easily by first describing the samples and core depths, then the sample petrography, then the relationships between the plagioclase and K-feldspar, and then the attempts to separate different materials. This content is somewhat mixed together in the paragraphs.

Done

• When I finished the Petrography section, the main point was not clear to me. There is a key sentence in the Radio-isotopic analysis section that describes the key conclusion of the petrologic observations. This sentence would be better placed in the Petrography section: "Petrography reveals the order of crystallization of K-bearing phases: plagioclase formed by quenching of the impact melt and later K-rich feldspar overgrowths grew during hydrothermal activity."

Have moved that sentence into the petrography section

• The third paragraph starts with "The swallow tail and skeletal plagioclase textures indicate…" Please clarify if these textures were present in all samples.

Amended this sentence to: "***Swallow tail and skeletal textures were visible in all plagioclase*** *indicating rapid crystal formation*" (line 154)

• The final "sentence" in the Petrography section is not actually a complete sentence. "Also veins of K-rich feldspar and albite…"

Amended that sentence to: "***Additionally,*** *veins of K-rich feldspar and albite w**ere reported** from the M0077A core indicating temperatures of >250°C(Kring et al. 2020) and relatively high temperature K-metasomatism in the form of veins with biotite, chlorite, and quartz **has been described** in samples from Yaxcopoil-1*(Zürcher and Kring 2004)." (lines 166-169)

Radio-isotopic analysis

• For each sample, please be clearer what was thought to be analyzed. Plagioclase? K-feldspar overgrowths? Why? This information is difficult to follow between the petrography and results section.

Have added this paragraph to the $^{40}Ar/^{39}Ar$ section (lines 171-177):

> *"Due to the small crystal size and complex texture of intergrown plagioclase and K-rich feldspar, separating the two phases during sample preparation was not possible, such that all analysed samples were mixed phases of varying proportions of plagioclase and K-feldspar. Based on visual inspection we interpret the argon budget of samples CHX 706.4, 735.0, and 756.0A to be dominated by the authigenic K-feldspar overgrowths. The argon budget of sample CHX 756.0B is dominated by plagioclase feldspar, though we cannot exclude the possibility that some authigenic feldspar was not included."*

• "40Ar/39Ar geochronology" would be a more specific header for this section.

Done

• Suggest combining the second and third paragraphs of this section. Both paragraphs describe the 22 plateau ages from the 3 samples.

Done

• In the 6th paragraph of this section, the authors interpret the “two youngest ages to be a result of imperfect separation of sample CHX 756.0 into two distinct lithologies…”. Please explain in the text why the two youngest ages are interpreted to be erroneously young due to imperfect separation. Are these youngest ages included in Figure 2A and 2B? I thought the younger ages were interpreted as due to protracted hydrothermal activity? Why would some young ages be attributed to hydrothermal activity, while others are interpreted as too young to be due to this effect? This section is not clear.

We have reworked this paragraph as follows (lines 200-209):

> *“Sample CHX 756.0B defined the oldest and two of the youngest ages in this study (Fig. 2). Due to the uneven boundary between samples CHX 756.0A and B, and challenges in visually differentiating them in hand sample we could not attain perfect physical separation during sample preparation. As a result of that challenge and in light of the data, we interpret the two youngest ages from 756.0B to contain more authigenic K-feldspar than the rest of sample 756.0B and therefore to have their 40Ar* budget dominated by younger hydrothermal phases rather than impact-melt generated plagioclase. The imperfect separation implies mixing between the age groups, and we can see mixing in the overlap between the two youngest CHX 756.0B ages and the CHX 735.0 and CHX 756.0A ages. This observation is unsurprising given the complex intermixing of textures and phases observed in these samples.”*

The discussion emphasizes that hydrothermal activity lasted at least 6.0-9.6 Myr.

• Where does 9.6 Myr come from? The Ar dates emphasized in the text and Figure 1 range from 58.3 to 66.2 Myr. This is an 8 Myr duration. Not 9.6 Myr.

• Where does the 6 Myr come from?

This range comes from subtracting the age of the youngest analysis (93303-10: 58.3 ± 1.3 Ma) from the age of the impact and propagating the uncertainty to yield 7.8 ± 1.8 Myr, which was then stated as a range to avoid being overly precise. We recognise that this value range is slightly different from that reported in the original text, which is attributable to rounding errors. We have clarified this on lines

> *“To establish a minimum duration of hydrothermal activity we calculate the difference between the age of the impact (66.052±0.086 Ma) and the youngest analysis (Run ID 93303-10: 58.3 ± 1.3 Ma), yielding 7.8 ± 1.3 Myr, which we report as ~8 Myr.”*

An enormous range of geothermal gradients was adopted in the impact simulations, ranging from 13 °C/km to 70 °C/km. Why was 13 °C/km adopted for one of the geothermal gradients? Abramov & Kring is cited here, but some explanation is needed.

We have added the following to clarify the purpose of using 13 °C/km (lines 271-278).

> *"Those initial simulations by Abramov and Kring used, as input, the post-impact thermal state generated by a previously published impact simulation(Ivanov 2004). Abramov and Kring(Abramov and Kring 2007) noted that a gradient of 13 ºC/km was a low value, producing a conservative estimate for the duration of hydrothermal activity, and that, in practice, hydrothermal activity may have persisted longer. We considered it important to replicate those results in this study in order to bracket our interpretations. As in the previous work, the duration(s) based on current modelling can be considered conservative; Hadean post-impact hydrothermal systems may have persisted longer."*

Figure 1. The different components of this figure are effective in progressively moving from the crater-scale to the lithologic column-scale to the sample scale. In B) it would be helpful to add dashed lines between the lithologic column on the left and the zoomed in section on the right, to be clearer where in the section that the samples are from.

Done

Figure 2. Caption states that the 66 Ma to 58 Ma age span affects all three samples, but it doesn't. CHX-756.0A shows a more limited range.

Changed this to: "*The age span…**comprises** all three samples…*"

Data tables

• The Ar data tables and associated information should follow the reporting guidelines of Schaen et al. 2021 (GSAB). That manuscript includes dozens of authors and reaches agreement on reporting recommendations. I think that some of the data that should be reported are missing from the tables? Unless I missed that information in the supplementary files somewhere.

Some of the required information is not in the tables but is included in the Methods section.

A note stating when samples were irradiated and when they were analysed has been added, and the data are now provided as a csv file as well as a word doc. These were the only missing requirements that we could identify.

• I also believe that additional information is needed in the diffusion experiment tables C1-C2 to align with recent reporting recommendations. The table should include ln(D/a2) and uncertainties. The references for and all parameters used in the calculation of ln(D/a2) should be explained.

We added values of (D/a2) and their uncertainties to tables C1-C2.

Thank you for your response to our manuscript and for the thoughtful reviews. My coauthors and I have worked to address the remaining reviewer concerns in the attached manuscript with tracked changes, and in the point-by-point responses below.

Reviewer #1 (Remarks to the Author):

The revised version is certainly improved compared to the earlier initial version. Most review comments were properly addressed. In my view the manuscript is now ready to be accepted.
Best regards, Christian Koeberl (Univ. Vienna)

> *Thank you for your feedback, and support to publish.*

Reviewer #2 (Remarks to the Author):

The authors have done a nice job responding to most the three reviewers' criticisms regarding the structure/organization of the manuscript and the questions/issues regarding the geochronologic data and interpretations. The substantial reorganization, especially of the Introduction, now provides much more context for the study. The only issue that now remains outstanding is that the authors continue to insist on correlating the post-Chicxulub impact hydrothermal conditions to those on the early Earth. In their response, the authors have correctly stated that other published papers have already proposed post-impact hydrothermal activity as a mechanism for creating habitable environments for thermophilic and hyperthermophilic microorganisms and generating cradles for prebiotic chemical reactions. Those inferences are exactly that. They are inferences that are not based on data. The continued attempts to correlate Chicxulub hydrothermal activity to that in the early Earth comes across as a desperate attempt to make the manuscript have more curb appeal. It is not needed. The extended duration of hydrothermal activity at Chicxulub is a very interesting finding that can stand on its own and it doesn't need to be stretched to the early Earth. Moreover, the maturity and structure of the Hadean crustal system is still an unknown and it is not clear if hydrothermal activity could be substantiated for a period as long as Chicxulub. Thus, the early Earth correlations on lines 27-33, 270-271, and 355-361 should be removed prior to publication.

> ***Lines 27-33 (now lines 39-46):*** *We have adjusted to make clear that Chicxulub is not a perfect analogue, but that we are examining the temperature and fluid flux conditions in a post-impact hydrothermal system as an analogue for large impact basins in general.*

"We cannot say with certainty that these early impact environments were inhabited, because so little of the rock record from Early Earth still exists and therefore the physical properties of the early Earth's crust are poorly constrained. We can however examine whether impact craters may have provided the right temperature and fluid flux conditions, for sufficiently long durations, for life to emerge. In this work we address the constraint of longevity by examining duration of the impact generated hydrothermal system in the Chicxulub peak-ring basin as an analogue for large impact basins in general.

Lines 270-271 (now lines 312-314): we have rephrased to remove reference to the Hadean.

"As in the previous work, the duration(s) based on current modelling can be considered conservative; natural post-impact hydrothermal systems may have persisted for longer."

Lines 355 to 361 (now lines 412-417): we have rephrased to be explicit that any relation to early Earth is a possibility, not a certainty.

"Although Chicxulub is still relatively small (200 km) compared to the impact basins expected on early Earth and observed on other planetary bodies (1000s km), it is possible that these larger impacts could have created even longer-lived hydrothermal systems and, therefore, could have been able to maintain the temperatures and fluid flux required for habitable environments for a minimum of several millions of years."

Reviewer #3 (Remarks to the Author):

The authors have thoroughly addressed my comments and I consider the manucript ready for publication.

Rebecca Flowers
University of Colorado Boulder

Thank you for your feedback, and support to publish.
